# Photoswitchable Catalysis by a Self-Assembled Molecular Cage

**DOI:** 10.1021/jacs.4c04846

**Published:** 2024-07-25

**Authors:** Ray G. DiNardi, Samina Rasheed, Simona S. Capomolla, Man Him Chak, Isis A. Middleton, Lauren K. Macreadie, Jake P. Violi, William A. Donald, Paul J. Lusby, Jonathon E. Beves

**Affiliations:** † School of Chemistry, UNSW Sydney, Sydney, New South Wales 2052, Australia; ‡ EaStCHEM School of Chemistry, University of Edinburgh, Joseph Black Building, David Brewster Road, Edinburgh, Scotland EH9 3FJ, U.K.

## Abstract

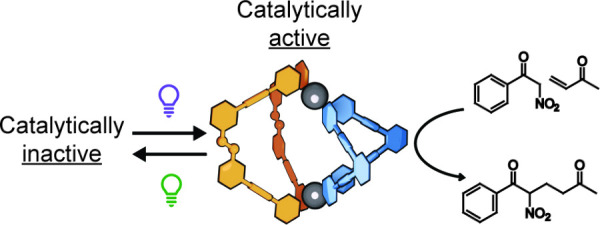

A heteroleptic [Pd_2_L_2_L’_2_]^4+^ coordination cage containing a photoswitchable azobenzene-derived ligand catalyzes the Michael addition reaction between methyl vinyl ketone and benzoyl nitromethane within its cavity. The corresponding homoleptic cages are catalytically inactive. The heteroleptic cage can be reversibly disassembled and reassembled using 530 and 405 nm light, respectively, allowing catalysis within the cage to be switched *OFF* and *ON* at will.

Over the past three decades, supramolecular cages have evolved from simple hosts to systems with impressive catalytic functions.^1^ Inspired by how enzymes accelerate reactions, catalytic cages can leverage their activity in multiple ways. These include methods that rely on reducing the entropy of activation, for example, using binding to limit conformational freedom^2^ or by encapsulating more than one substrate to increase effective concentration.^3^ Cages can also use electrostatic forces to drive catalysis, for example leading to either enhanced basicity^4^ or acidity^5^ of the substrate compared to the nonbound species, and/or stabilizing any subsequent higher energy species.^6^ Other methods of accelerating reactions include binding substrates in higher energy conformational states,^7^ or using the local high concentration of ions around the cage portals.^8^ When more than one of these mechanisms is used simultaneously, very high activity can be observed.^9^ Molecular cages have also been used to control regioselectivity, although these reactions are not typically catalytic.^10^

A key feature of enzyme catalysis is the regulated activity. This area of cage catalysis remains significantly underdeveloped and invariably relies on either the endo or exo binding of a guest that is not a substrate.^11^ One way to mediate cage catalysis would be using light irradiation. Light-responsive molecular cages have been prepared using photoswitchable ligands and metal ions.^12^ These include those based on diarylethene photoswitches that form cages with different geometries and cavity sizes, allowing selective guest binding.^13^ Diazocine-based cages are unique as they can be switched from a thermodynamically stable *Z*-isomer to the *E*-isomer using UV light, forming metastable cages that encapsulate guests.^14^ A related example uses two diazocine ligands that allow light to selectively disassemble one cage and assemble another.^15^ We have recently reported an azobenzene-based molecular cage that reversibly responds to visible light to change its composition from a [Pd_2_L_4_]^4+^ lantern-like structure to a [PdL_2_]^2+^ monomeric product.^16^ However, guest molecules do not readily bind within the cavity of the lantern-like cage,^16^ instead preferentially binding on the exterior. Molecular cages can also act as photosensitizers^17^ and have been used to drive photochemical reactions away from equilibrium.^18^

Photoswitchable catalysis^19^ has progressed significantly since the earliest reports,^20^ with examples of enantioselective catalysis,^21^ polymerization^22^ and cooperative catalysis.^23^ In these examples, the catalysis is switched by changing the electronic properties of a donor atom,^24^ blocking an active site with steric bulk,^20^ bringing together cooperative organocatalytic groups,^21a^ or forming a more reactive functional group.^25^ While there are reports of switchable catalysis within macrocycles,^26^ on surfaces of nanoparticles,^27^ and using rotaxanes,^28^ to the best of our knowledge, there are no reports of photoswitchable catalysis using discrete self-assembled species. Herein we report light-regulated catalysis using a heteroleptic [Pd_2_L_2_L′_2_]^4+^ cage system (Figure 1).

**Figure 1:**
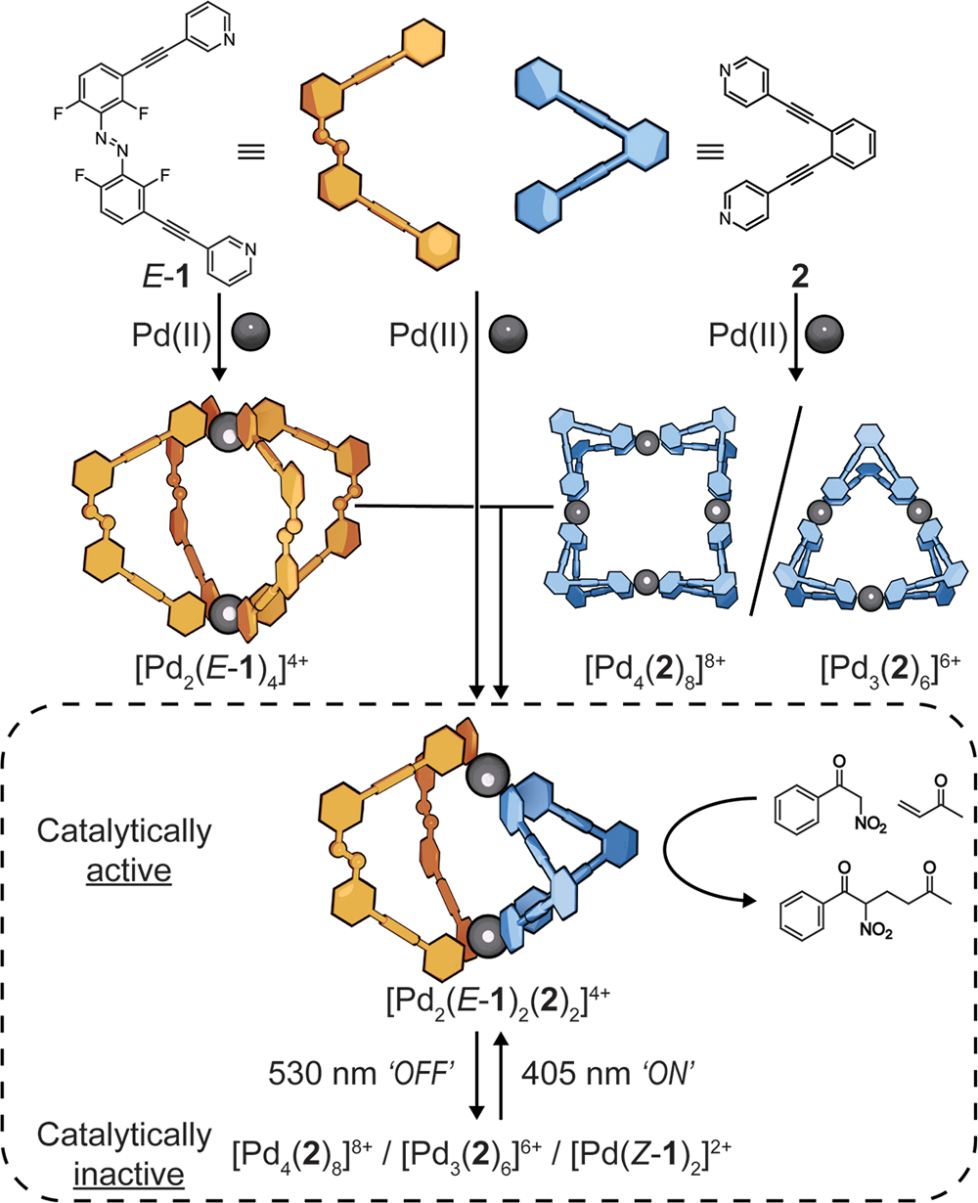
Self-assembly of homoleptic cages [Pd_2_(*E*-**1**)_4_]^4+^, [Pd_4_(**2**)_8_]^8+^, [Pd_3_(**2**)_6_]^6+^ and photoswitchable heteroleptic cage catalyst [Pd_2_(*E*-**1**)_2_(**2**)_2_]^4+^.

Photoswitchable ligand **1** was synthesized via Sonogashira cross-coupling (see Supporting Information S2) of 3-bromo-2,6-difluoroaniline and 3-ethynylpyridine to give 2,6-difluoro-3-(pyridin-3-ylethynyl)aniline in 80% yield. The reaction of two equivalents of this aniline with *N*-chlorosuccinimide (NCS) and 1,8-diazabicyclo[5.4.0]undec-7-ene (DBU)^29^ gave photoswitchable ligand **1** in 33% yield. A single-crystal X-ray structure of *E*-**1** (CCDC: 2343887, see Supporting Information S2.4) confirmed the rings are almost coplanar, like related ligands we have reported.^16,30^

The photoswitching properties of ligand **1** were investigated using NMR and UV-vis absorption spectroscopies (see Supporting Information S3). Photostationary states (PSS) were generated by irradiating a sample of *E*-**1** in DMSO-*d*_6_ with a 530 nm LED for 10 min (PSS_530_ = 88% *Z*-**1**) or 405 nm LED for 20 min (PSS_405_ = 86% *E*-**1**). The metastable isomer, *Z*-**1**, has a thermal half-life of around a month at room temperature in DMSO (see Supporting Information S3.3), in line with that of related switches.^16^

Reaction of ligand *E*-**1** (4.6 mM, 1 equiv.) and [Pd(MeCN)_4_](BF_4_)_2_ (2.3 mM, 0.5 equiv.) in DMSO-*d*_6_ gives the homoleptic lantern-like complex [Pd_2_(*E*-**1**)_4_](BF_4_)_4_, as shown by the combination of NMR spectroscopy (Figure 2a) and electrospray ionization mass spectrometry (ESI-MS) (see Supporting Information S6). When [Pd_2_(*E*-**1**)_4_](BF_4_)_4_ in DMSO-*d*_6_ is irradiated with 530 nm light, the monomeric complex [Pd(*Z*-**1**)_2_](BF_4_)_2_ is formed, as confirmed by NMR and ESI-MS experiments (see Supporting Information S7). This behavior is analogous to that of a related [Pd_2_L_4_](BF_4_)_4_ cage.^16^ With this favorable photochemical switching, the host-guest chemistry of [Pd_2_(*E*-**1**)_4_]^4+^ was then explored using the analogous tetrakis[3,5-bis(trifluoromethyl)phenyl]borate (BAr_F_) salt.^31^ The use of these large, “greasy” counteranions facilitates both host-guest chemistry^31^ and catalysis^1e^ with simple Pd_2_L_4_ cages. This is because they maximize polar and electrostatically activating interactions in less polar solvents by removing competitive counteranion binding.

**Figure 2:**
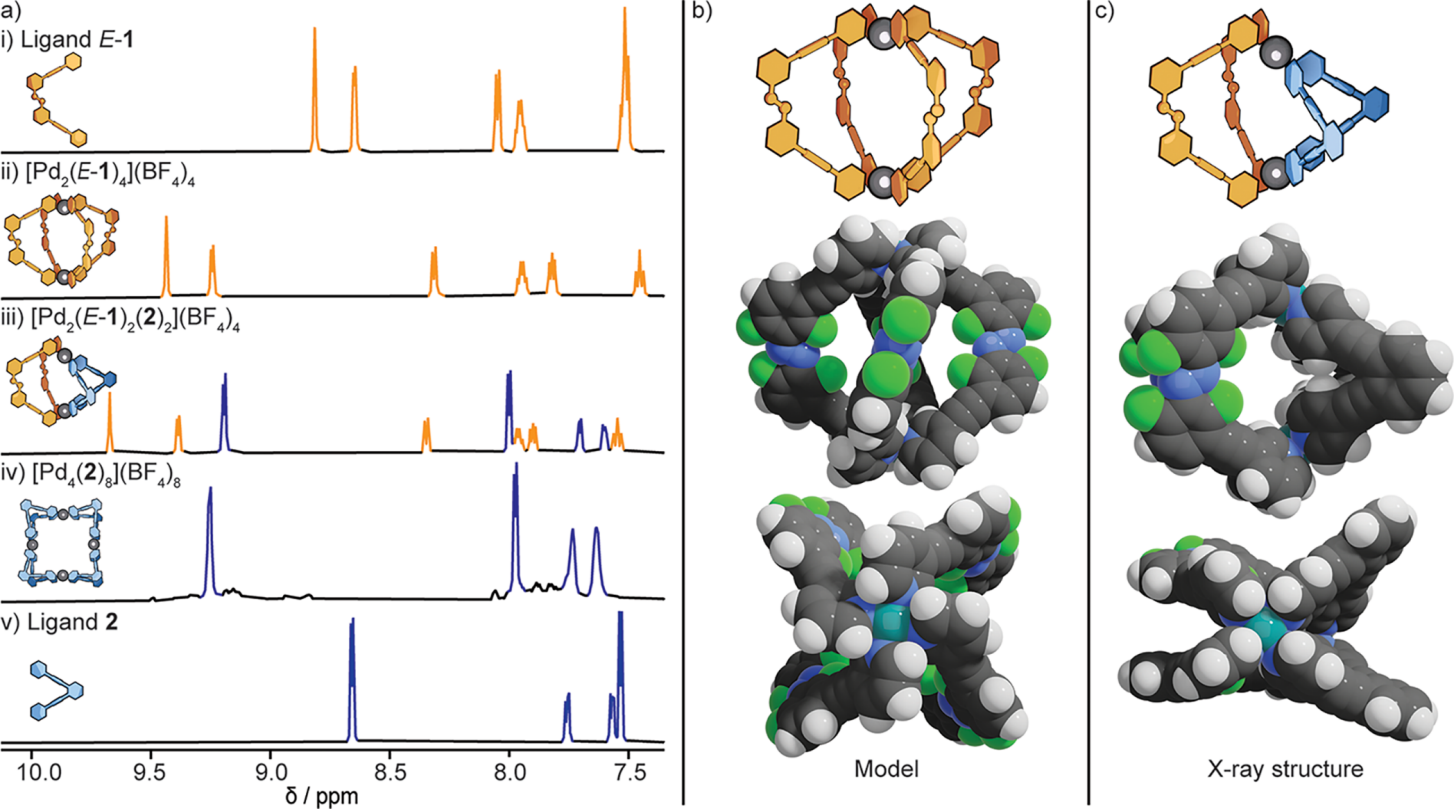
Characterization of homoleptic and heteroleptic cages. a) ^1^H NMR spectra (600 MHz, DMSO-*d*_6_) of (i) photoswitchable ligand *E*-**1**; (ii) homoleptic [Pd_2_(*E*-**1**)_4_](BF_4_)_4_; (iii) heteroleptic [Pd_2_(*E*-**1**)_2_(**2**)_2_](BF_4_)_4_; (iv) a mixture of homoleptic [Pd_4_(**2**)_8_](BF_4_)_8_ and [Pd_3_(**2**)_6_](BF_4_)_6_, as reported;^34^ (v) ligand **2**. b) Molecular mechanics model of homoleptic [Pd_2_(*E*-**1**)_4_]^4+^. c) Single crystal X-ray structure of heteroleptic [Pd_2_(*E*-**1**)_2_(**2**)_2_](BF_4_)_4_, CCDC: 2343886. Color codes: gray: carbon; white: hydrogen; blue: nitrogen; green: fluorine; teal: palladium. Anions and solvent molecules are omitted for the sake of clarity.

To prepare the BAr_F_ salt, *E*-**1** was reacted with [Pd(Py*)_4_](BAr_F_)_2_ (where Py* = 3-chloropyridine, see Supporting Information S4)^32^ in CD_3_CN to give [Pd_2_(*E*-**1**)_4_](BAr_F_)_4_, which was characterized by NMR and ESI-MS data (see Supporting Information S6.4, S6.5). Disappointingly, [Pd_2_(*E*-**1**)_4_](BAr_F_)_4_ showed little evidence of guest binding, nor was it able to catalyze the representative Michael addition reaction of methyl vinyl ketone and benzoyl nitromethane in the presence of 18-crown-6, similar to the behavior of a related cage^16^ (Supporting Information S5.2, S5.3, S18.4.3).

There are several possible reasons for this lack of host-guest chemistry and reactivity. It could be that the relative size and shape of the cavity of [Pd_2_(*E*-**1**)_4_](BAr_F_)_4_ are unsuitable for encapsulating the substrates. A molecular model of the homoleptic cage also reveals another potential problem; [Pd_2_(*E*-**1**)_4_]^4+^ likely has a pronounced twisted conformation (Figure 2b), a consequence of the nonparallel coordination vectors of *E*-**1**, similar to a related cage.^16^ It has previously been shown that guest binding inside non-twisted, *D*_4*h*_ symmetric Pd_2_L_4_ cages is facilitated by the formation of hydrogen bonds with the two sets of four polarized *ortho*-pyridyl protons that point directly into the cavity.^31^ These interactions also drive Michael addition catalysis by stabilizing the deprotonated nucleophile and co-binding the electrophile to reduce the entropy of activation.^33^ In the case of [Pd_2_(*E*-**1**)_4_]^4+^, we attribute the lack of host-guest chemistry and catalysis to the twisted, propeller-like conformation of the Pd(pyridyl)_4_ units. This twisting perturbs the convergent cavity-directed hydrogen-bond donor atoms, hindering the formation of favorable electrostatic interactions that infer catalytic activity. Looking at an alternative cage design and to address the twisted conformation that could hinder catalysis, we targeted a heteroleptic system combining rigid ligand **2** with *E*-**1**. This combination of ligands was selected for their shape complementarity.^35^ On its own, ligand **2** is reported^34^ to react with palladium(II) ions to form a [Pd_3_(**2**)_6_]^6+^ double-walled triangle in acetonitrile and a [Pd_4_(**2**)_8_]^8+^ double-walled square in DMSO and never a [Pd_2_(**2**)_4_]^4+^ dimer, which is also our observation (Figure 2aiv, Supporting Information S8 and S9).^34^

When one equivalent of each of ligand *E*-**1**, ligand **2**, and [Pd(MeCN)_4_](BF_4_)_2_ are combined in DMSO-*d*_6_ a single new species is formed within 10 min at room temperature (Figure 2aiii, Supporting Information S10). An identical result is obtained if the homoleptic cages [Pd_2_(*E*-**1**)_4_](BF_4_)_4_ and [Pd_4_(**2**)_8_](BF_4_)_8_ are combined in DMSO-*d*_6_ and heated with a heat gun for 5 min (see Supporting Information S10.1), indicating that the heteroleptic cage is the thermodynamic product. Multinuclear NMR experiments (Supporting Information S10.2) confirm that the heteroleptic cage is symmetrical with a single environment for each of the ligands, *E*-**1** and **2**. Interligand ROESY interactions indicate that both ligands are coordinated to the same metal ion (Supporting Information S10.2). Diffusion NMR experiments in DMSO-*d*_6_ (see Supporting Information S13) gave hydrodynamic radii in line with expectations: [Pd_2_(*E*-**1**)_2_(**2**)_2_]^4+^ (9.1 nm), smaller than that of the homoleptic [Pd_2_(*E*-**1**)_4_]^4+^ (10.5 nm) and the macrocyclic [Pd_4_(**2**)_8_]^8+^ (9.9 nm). ESI-MS confirmed the composition as [Pd_2_(**1**)_2_(**2**)_2_]^4+^ with a series of cations with isotope patterns corresponding to sequential loss of BF_4_ anions (Supporting Information S10.3).

Finally, a single crystal suitable for X-ray diffraction unambiguously confirmed the heteroleptic species as *cis*-[Pd_2_(*E*-**1**)_2_(**2**)_2_](BF_4_)_4_ (Figure 2c, CCDC: 2343886, Supporting Information S10.4), in line with expectations from shape complementarity prediction.^35^ The cage has a Pd···Pd separation of 9.92 Å and has a BF_4_ anion inside the cavity, confirming its ability to bind guests. The pyridyl units are not significantly twisted (angles between the *trans* pyridyl rings range from 16 to 22°), and therefore the *ortho*-pyridyl hydrogen-bond donors project into the cavity for optimal guest binding and catalysis.

The heteroleptic cage [Pd_2_(*E*-**1**)_2_(**2**)_2_](BF_4_)_4_ in DMSO-*d*_6_ is disassembled when irradiated with 530 nm light, and the ^1^H NMR spectrum (Figure 3ii) shows a mixture of products is formed. The mixture includes [Pd(*Z*-**1**)_2_]^2+^ and [Pd_4_(**2**)_8_]^8+^, and possibly [Pd_2_(*Z*-**1**)_2_(**2**)_2_]^4+^ (Supporting Information S11). Upon irradiating with 405 nm light the heteroleptic cage [Pd_2_(*E*-**1**)_2_(**2**)_2_](BF_4_)_4_ is reformed near quantitatively (Figure 3iii, Supporting Information S11.3). This data confirm that heteroleptic cage [Pd_2_(*E*-**1**)_2_(**2**)_2_](BF_4_)_4_ can be reversibly assembled and disassembled using visible light.

**Figure 3:**
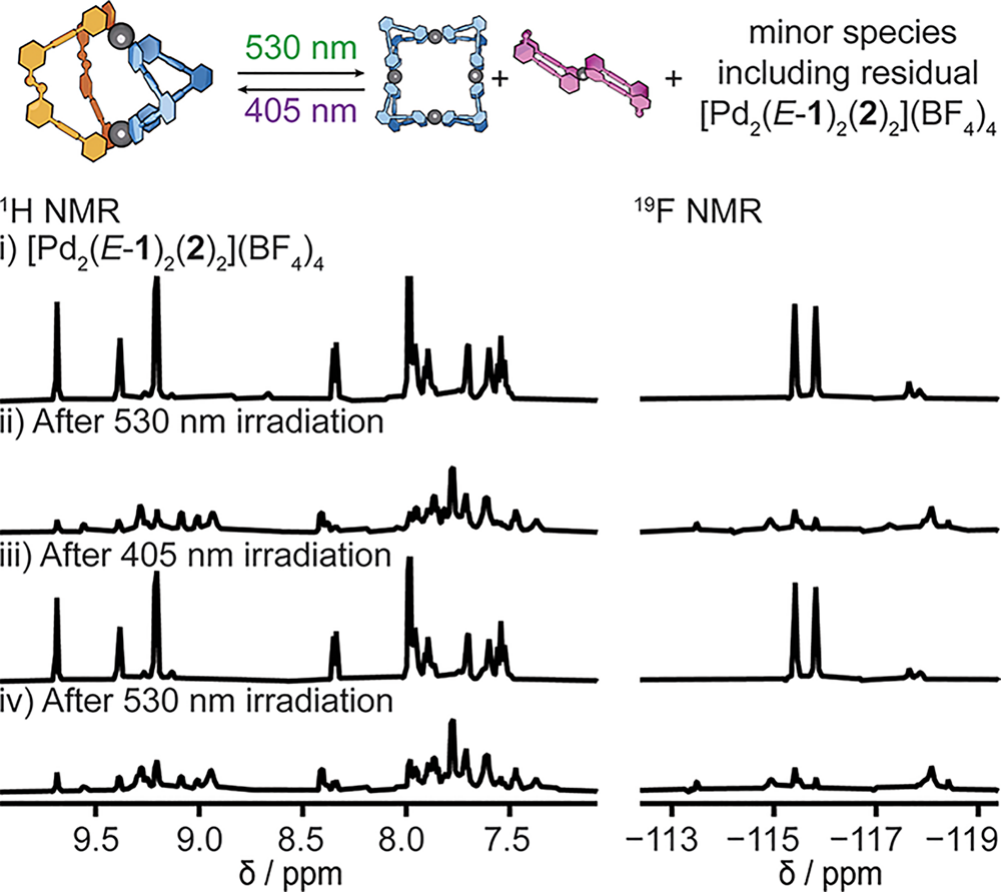
Partial ^1^H (600 MHz) and ^19^F (565 MHz) NMR spectra in DMSO-*d*_6_ showing photoswitching of heteroleptic cage [Pd_2_(*E*-**1**)_2_(**2**)_2_](BF_4_)_4_ (i) before irradiation; (ii) after 530 nm 10 min; (iii) 405 nm 5 min; (iv) 530 nm 10 min irradiation. The PSS composition (PSS_530_ = 88% *Z*-**1**, the same as that for free ligand **1**) was confirmed by adding *N*,*N*-dimethyl-4-aminopyridine (DMAP) to displace the ligands. See Supporting Information S11.3 for details.

Turning to the equivalent BAr_F_ salt, we then tested whether the heteroleptic cage [Pd_2_(*E*-**1**)_2_(**2**)_2_](BAr_F_)_4_ could be formed and, in particular, whether it could be generated from the rearrangement of the two homoleptic structures. Homoleptic structures [Pd_2_(*E*-**1**)_4_](BAr_F_)_4_ and [Pd_3_(**2**)_6_](BAr_F_)_6_/[Pd_4_(**2**)_8_](BAr_F_)_8_ can be assembled with [Pd(Py*)_4_](BAr_F_)_2_ in CD_3_CN, and were characterized by NMR (Supporting Information S6.4, S9.4) and ESI-MS data (Supporting Information S6.5, S9.5). When a 1:1 mixture of these two homoleptic cages was combined in CD_3_CN and heated at 50 °C for 30 min, the heteroleptic cage was formed quantitatively (Supporting Information S10.5). This indicates that swapping from BF_4_ to BAr_F_ counteranions does not lead to problems with kinetic trapping. However, the solvent that is optimal for catalysis—dichloromethane—is poorly coordinating and therefore does not promote the rapid ligand exchange required for cage switching. We found that a solvent mixture of 11:1 CD_2_Cl_2_/CD_3_CN, was a good compromise to maximize the host-guest chemistry while providing some coordinating properties to facilitate cage rearrangement (see Supporting Information S14, S15). Using these mixed solvent conditions, we investigated the binding of methyl vinyl ketone and benzoyl nitromethane within [Pd_2_(*E*-**1**)_2_(**2**)_2_](BAr_F_)_4_ (Supporting Information S17) as well as the ability of [Pd_2_(*E*-**1**)_2_(**2**)_2_](BAr_F_)_4_ to catalyze the Michael addition reaction between these two substrates (Supporting Information S18).^36^ When methyl vinyl ketone is added to a sample of the heteroleptic cage, only minor shifts were observed in the ^1^H NMR peaks of the cage, with no substantial changes when 18-crown-6 is also added (see Supporting Information 17.1). By contrast when benzoyl nitromethane is added a new set of deshielded signals are observed that correspond to the cage with bound deprotonated benzoyl nitromethane. These signals increase in intensity once 18-crown-6 is added (see Supporting Information 17.2). The signals that show the biggest difference in chemical shift compared to the “empty cage” correspond to the protons adjacent to the pyridyl nitrogen of the nonswitchable ligand **2**, and of the inwardly directed *ortho*-pyridyl CH of the photoswitchable ligand **1**. These chemical shift differences are in line with those observed from simple Pd_2_L_4_ cages^1e,5,31^ and are consistent with the binding of the substrates inside the cage through CH···O hydrogen bonds (Figure 4a).^33^ We have also found that triflate binds tightly inside the cage, similarly evidenced by the appearance of a second set of cage signals with deshielded inward-facing proton resonances (Supporting Information S17.4).

**Figure 4:**
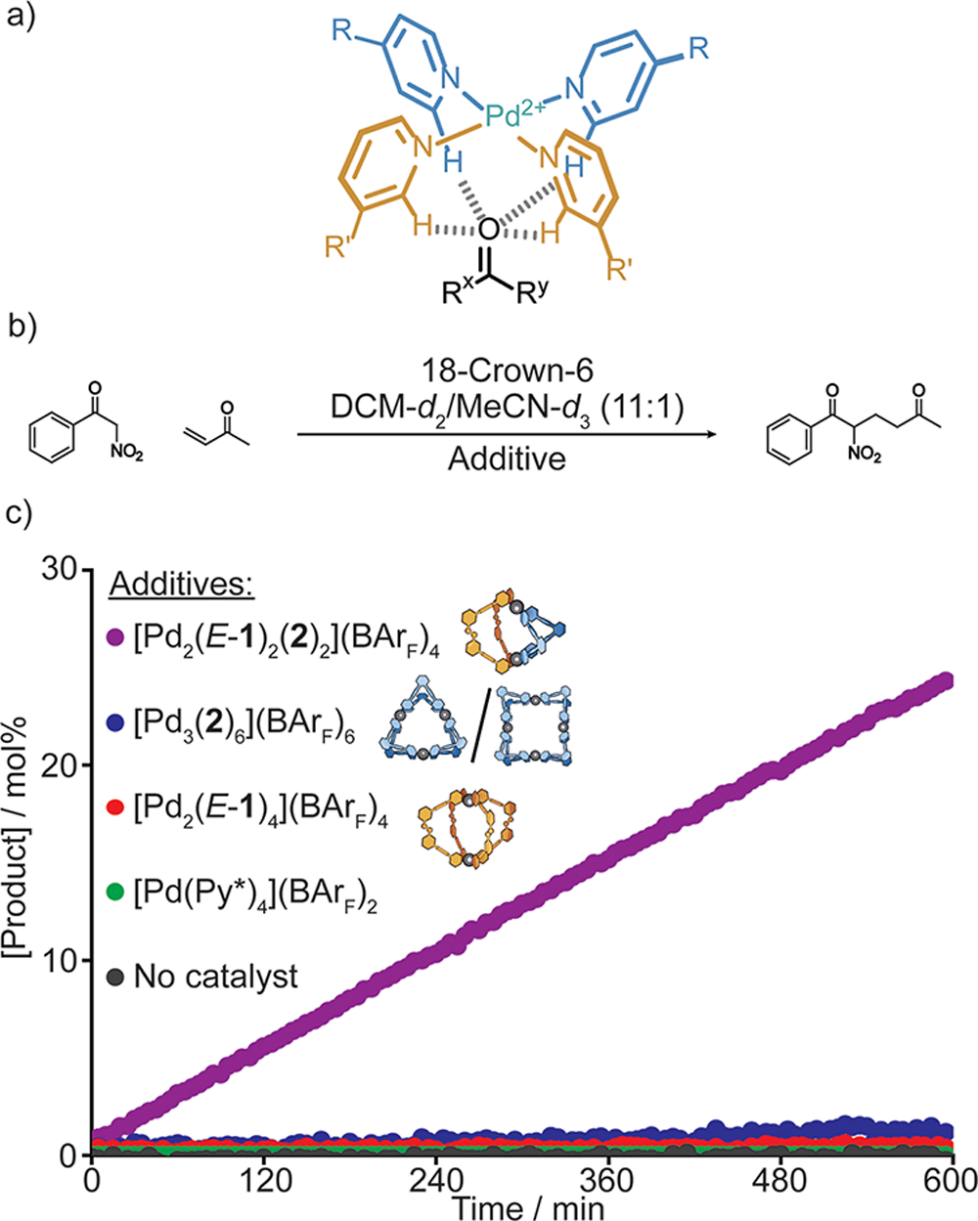
Catalysis of a Michael addition reaction by self-assembled cages. a) Substrate binding inside the heteroleptic cage via CH···O hydrogen bonds. b) Michael addition reaction between benzoyl nitromethane and methyl vinyl ketone. c) Michael addition reaction with different additives. Reaction conditions: CD_2_Cl_2_/CD_3_CN (11:1), benzoyl nitromethane (14 mM), methyl vinyl ketone (27 mM), and 18-crown-6 (11 mM). All cases with palladium [Pd] = 1.6 mM. Product formation was measured by ^1^H NMR spectroscopy. See Supporting Information S18.4.

When both substrates are present, this clear indication of substrate binding is accompanied by substrate consumption and the generation of the Michael addition product, as monitored using ^1^H NMR spectroscopy (Figure 4, Supporting Information S18). An 11% catalyst loading of heteroleptic species [Pd_2_(*E*-**1**)_2_(**2**)_2_](BAr_F_)_4_ converted 24% of the benzoyl nitromethane to the Michael addition product in 10 h.

Having already found that the homoleptic photoswitchable cage [Pd_2_(*E*-**1**)_4_](BAr_F_)_4_ is not a catalyst (see above), we also tested whether [Pd_3_(**2**)_6_](BAr_F_)_6_/[Pd_4_(**2**)_8_](BAr_F_)_8_ would show any reactivity. These homoleptic species showed virtually no catalysis, with <1% benzoyl nitromethane going to the product over 10 h. To ensure that [Pd_2_(*E*-**1**)_2_(**2**)_2_](BAr_F_)_4_ was responsible for the catalysis, control experiments were performed using no catalyst and with [Pd(Py*)_4_](BAr_F_)_2_. For both control experiments, no product formation was observed. Crucially, we have also found that the addition of one equivalent of the strongly binding guest triflate to [Pd_2_(*E*-**1**)_2_(**2**)_2_](BAr_F_)_4_ effectively halts catalytic activity (Supporting Information S19.5). This observation provides further strong support for a mechanism that involves substrate encapsulation.

Having shown that we can reversibly switch the heteroleptic cage with light and that it is also an active catalyst, it was time for photoswitchable catalysis! The [Pd_2_(*E*-**1**)_2_(**2**)_2_](BAr_F_)_4_ cage was successfully disassembled (530 nm light for 10 min) and reassembled (405 nm light for 5 min) in 11:1 CD_2_Cl_2_/CD_3_CN (see Supporting Information S19), showing similar behavior to the BF_4_ salt in DMSO-*d*_6_ (Figure 3).^31^ Benzoyl nitromethane, methyl vinyl ketone, and 18-crown-6 were added to the sample, and the reaction was monitored using ^1^H NMR spectroscopy (Figure 5a), showing the cage was catalytically active. Next, the sample was irradiated with a 530 nm LED for 10 min, which resulted in a 10-fold decrease in the rate of product formation as the cage was disassembled. The reaction was then reactivated by irradiating with a 405 nm LED for 5 min. Following this reactivation, the rate of product formation was almost identical to that prior to 530 nm irradiation, showing that the photoswitching is completely reversible.

**Figure 5:**
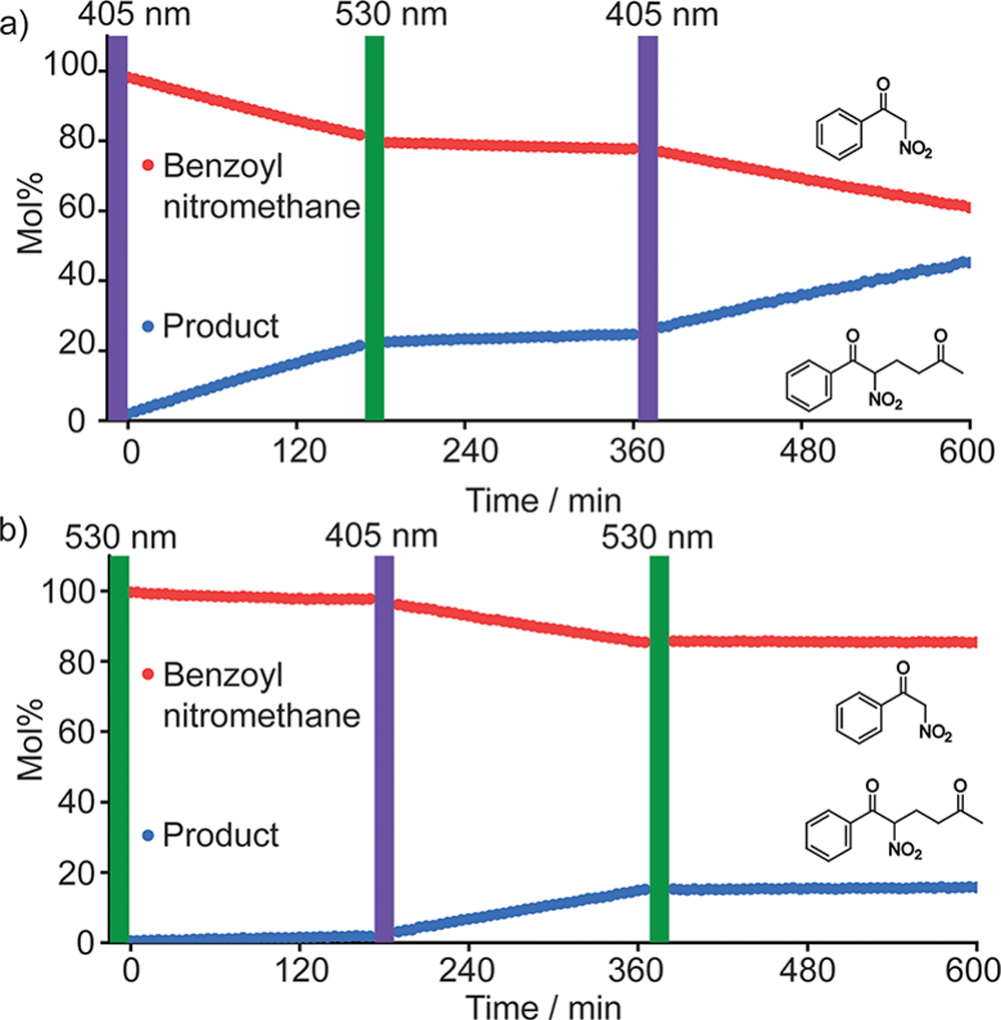
Photoswitchable catalysis by heteroleptic cage [Pd_2_(*E*-**1**)_2_(**2**)_2_](BAr_F_)_4_ in CD_2_Cl_2_:CD_3_CN 11:1 monitored by ^1^H NMR spectroscopy. a) ON/OFF/ON cycle and b) OFF/ON/OFF cycle. Reaction conditions: benzoyl nitromethane (17 mM), methyl vinyl ketone (33 mM), and 18-crown-6 (12 mM), [Pd] = 2.0 mM. Irradiation by 405 nm (5 min) and 530 nm (10 min) outside of the NMR instrument, with colored bars representing the time between NMR measurements; see Supporting Information S19 for details. The system can also be kept dormant by first irradiating [Pd_2_(*E*-**1**)_2_(**2**)_2_](BAr_F_)_4_ with a 530 nm LED before the substrates are added (Figure 5b, Supporting Information S19.3). The reaction can then be activated at will by irradiating the sample with a 405 nm LED. The long thermal half-life of the photoswitch ensures that the cage remains in the state it is programmed after the irradiation is stopped. The responsiveness of the system to visible light demonstrates that using a molecular photoswitch to control self-assembly can lead to excellent control of the chemical reactivity. We also show that the system can be subjected to at least five cycles of photoswitching without any effect on catalytic performance (Supporting Information S19.4).

In conclusion, we have shown the first example of photoswitchable catalysis within a self-assembled molecular cage. The mechanism of catalysis relies on electrostatic interactions within the cavity, which is possible only in the heteroleptic cage with a cavity preorganized for guest binding. The catalysis can be switched ON and OFF with visible light (405 nm and 530 nm, respectively) and is entirely reversible. Controlling the catalytic activity of self-assembled cavities with nondestructive visible light is a new method for directing chemical reactions. Combining photoswitchable ligands with facile ligand-exchange reactions allows a system to be driven toward assemblies composed of different components, with programmable stoichiometries, shapes, affinities, and now catalytic functions. We anticipate future examples could include different self-assembled cages each capable of catalyzing different reactions, allowing more complex multistep reactions to be performed simply by using visible light.

## Supporting Information Available

Experimental data, spectra and other data (PDF)
A preprint of this work, including all raw data, is available at ChemRxiv, DOI: 10.26434/chemrxiv-2024-vdkfj
