# Supplementary material for: Photoswitchable Catalysis by a Self-Assembled Molecular Cage
Source: J Am Chem Soc. 2024 Jul 25;146(31):21196–202. doi: 10.1021/jacs.4c04846 (PMC11311219; doi:10.1021/jacs.4c04846)
Supplement: Supplementary file 1 — ja4c04846_si_001.pdf [file ja4c04846_si_001.pdf]

Electronic Supporting Information

Photoswitchable catalysis by a self-assembled molecular cage

Ray G. DiNardi†, Samina Rasheed†, Simona S. Capomolla†, Man Him Chak†, Isis A. Middleton†, Lauren K. Macreadie,† Jake P. Violi†, William A. Donald†, Paul J. Lusby‡*, and Jonathon E. Beves†*

†School of Chemistry, UNSW Sydney, Sydney, New South Wales 2052, Australia
‡EaStCHEM School of Chemistry, University of Edinburgh, Joseph Black Building, David Brewster Road, Edinburgh, Scotland, EH9 3FJ, U.K

Table of Contents

S1. General Experimental **5**
S1.1 General Experimental 5

S2. Synthesis of photoswitchable ligand 1 ..... **6**
S2.1 Synthetic overview 6
S2.2 Synthesis and characterization of intermediate **3** 7
S2.2.1 Synthesis of intermediate **3** 7
S2.2.2 1D and 2D NMR spectra of **3** in DMSO-d_6 8
S2.3 Synthesis and characterization of photoswitchable ligand **1** 11
S2.3.1 Synthesis of ligand **1** 11
S2.3.2 1D and 2D NMR spectra of **1** in DMSO-d_6 12
S2.4 Single crystal X-ray structure of *E*-**1** (CCDC: 2343887) 16

S3. Photoswitching properties of ligand 1 ..... **18**
S3.1 Determining the PSS distributions of ligand **1** in DMSO-d_6 using ^{1}H and ^{19}F NMR spectroscopy. 18
S3.2 Measuring UV-vis spectra of ligand **1** at different PSS in DMSO 21
S3.3 Determining the thermal stability of ligand *Z*-**1** in DMSO using UV-vis spectroscopy 23

S4. Synthesis of palladium(II) complexes **25**
S4.1 Synthesis of $[Pd(Py^*)_4](OTf)_2$ 25
S4.2 Synthesis of $[Pd(Py^*)_4](BAr_F)_2$ 26

S5. Preliminary guest binding studies between $[Pd_2L_4](BArF)_4$ in DCM **27**
S5.1 Preparing $[Pd_2(L)_4](BAr_F)_4$ in DCM-d_2 27
S5.2 Screening guest binding between $[Pd_2(L)_4](BAr_F)_4$ and different Michael addition acceptors and donors 28
S5.3 Catalysing the Michael addition between vinyl methyl ketone and benzoyl nitromethane using $[Pd_2(L)_4](BAr_F)_4$ 29

S6. Synthesis and characterization of homoleptic cage $[Pd_2(E\text{-}1)_4]^{4+}$ **30**
S6.1 Synthesis of $[Pd_2(E\text{-}\mathbf{1})_4](BF_4)_4$ in DMSO-d_6 30
S6.2 1D and 2D NMR spectra of $[Pd_2(E\text{-}\mathbf{1})_4](BF_4)_4$ in DMSO-d_6 32
S6.3 ESI-MS spectra of $[Pd_2(E\text{-}\mathbf{1})_4](BF_4)_4$ in DMSO 37
S6.4 Synthesis of $[Pd_2(E\text{-}\mathbf{1})_4](BAr_F)_4$ in MeCN-d_3 38
S6.5 ESI-MS spectra of $[Pd_2(E\text{-}\mathbf{1})_4](BAr_F)_4$ in MeCN 39

S7. Photoswitching of $[Pd_2(E\text{-}1)_4]^{4+}$ **40**
S7.1 Synthesis of $[Pd(Z\text{-}\mathbf{1})_2](BF_4)_2$ in DMSO-d_6 40
S7.2 1D and 2D NMR spectra of $[Pd(Z\text{-}\mathbf{1})_2](BF_4)_2$ in DMSO-d_6 41
S7.3 Measuring PSS distribution in DMSO 46
S7.4 ESI-MS spectra of $[Pd(Z\text{-}\mathbf{1})_2](BF_4)_2$ in DMSO-d_6 48
S7.5 ESI-MS spectra of $[Pd(Z\text{-}\mathbf{1})_2](BAr_F)_2$ in MeCN 49

S8. Synthesis and characterization of ligand 2 ..... **50**
S8.1 Synthesis of ligand **2** 50
S8.2 1D and 2D NMR spectra of **2** in DMSO-d_6 51

S9. Synthesis and characterization of homoleptic species $[Pd_4(\mathbf{2})_8]^{8+}$ and $[Pd_3(\mathbf{2})_6]^{6+}$ 54
S9.1 Synthesis of $[Pd_4(\mathbf{2})_8](BF_4)_8$ in DMSO 54
S9.2 1D and 2D NMR spectra of $[Pd_4(\mathbf{2})_8](BF_4)_8$ in DMSO-d_6 55
S9.3 ESI-MS spectrum of $[Pd_4(\mathbf{2})_8](BF_4)_8$ in DMSO 58
S9.4 Synthesis of $[Pd_3(\mathbf{2})_6](BAr_F)_6$ in MeCN 59
S9.5 ESI-MS spectrum of $[Pd_3(\mathbf{2})_6](BAr_F)_6$ and $[Pd_4(\mathbf{2})_6](BAr_F)_8$ in MeCN 60

S10. Synthesis and characterization of heteroleptic cage $[Pd_2(E\text{-}\mathbf{1})_2(\mathbf{2})_2]^{4+}$ 62
S10.1 Synthesis of $[Pd_2(E\text{-}\mathbf{1})_2(\mathbf{2})_2](BF_4)_4$ in DMSO-d_6 62
S10.2 1D and 2D NMR spectra of $[Pd_2(E\text{-}\mathbf{1})_2(\mathbf{2})_2](BF_4)_4$ in DMSO-d_6 64
S10.3 ESI-MS spectra of $[Pd_2(E\text{-}\mathbf{1})_2(\mathbf{2})_2](BF_4)_4$ in DMSO 68
S10.4 Single crystal X-ray structure of $[Pd_2(E\text{-}\mathbf{1})_2(\mathbf{2})_2](BF_4)_4$ (CCDC: 2343886) 69
S10.5 Synthesis of $[Pd_2(E\text{-}\mathbf{1})_2(\mathbf{2})_2](BAr_F)_4$ in CD_3CN 71
S10.6 ESI-MS spectra of $[Pd_2(E\text{-}\mathbf{1})_2(\mathbf{2})_2](BAr_F)_4$ in MeCN 72

S11. Photoswitching of $[Pd_2(E\text{-}\mathbf{1})_2(\mathbf{2})_2](BF_4)_4$ 73
S11.1 Irradiating $[Pd_2(E\text{-}\mathbf{1})_2(\mathbf{2})_2](BF_4)_4$ with 530 nm light in DMSO-d_6 73
S11.2 2D NMR spectra of the heteroleptic mixture in DMSO-d_6 after irradiation with 530 nm light 75
S11.3 Measuring PSS distribution for $[Pd_2(E\text{-}\mathbf{1})_2(\mathbf{2})_2](BF_4)_4$ in DMSO-d_6 after irradiation with 530 nm light 77
S11.4 ESI-MS spectra of $[Pd_2(E\text{-}\mathbf{1})_2(\mathbf{2})_2](BF_4)_4$ in DMSO before and after irradiation with 530 nm light 79
S11.5 ESI-MS spectra of $[Pd_2(E\text{-}\mathbf{1})_2(\mathbf{2})_2](BAr_F)_4$ in MeCN before and after irradiation with 530 nm light 80

S12. Spartan models of $[Pd_2(E\text{-}\mathbf{1})_4]^{4+}$, $[Pd_2(E\text{-}\mathbf{1})_2(\mathbf{2})_2]^{4+}$ and $[Pd(Z\text{-}\mathbf{1})_2]^{2+}$ 81

S13. Diffusion NMR data for $[Pd_2(E\text{-}\mathbf{1})_4](BF_4)_4$, $[Pd_4(\mathbf{2})_8](BF_4)_8$ and $[Pd_2(E\text{-}\mathbf{1})_2(\mathbf{2})_2](BF_4)_4$ in DMSO-d_6 83
S13.1 Gradient calibration 83
S13.2 Diffusion coefficient of the homoleptic switchable cage $[Pd_2(E\text{-}\mathbf{1})_4](BF_4)_4$ in DMSO-d_6 85
S13.3 Diffusion coefficient of the heteroleptic cage $[Pd_2(E\text{-}\mathbf{1})_2(\mathbf{2})_2](BF_4)_4$ in DMSO-d_6 87
S13.4 Diffusion coefficient of the homoleptic cage $[Pd_4(\mathbf{2})_8](BF_4)_4$ in DMSO-d_6 89

S14. Controlling catalysis with light using $[Pd_2(E\text{-}\mathbf{1})_2(\mathbf{2})_2](BAr_F)_4$ in DCM/$MeNO_2$ (9:1) 91

S15. The effect of solvent on self-assembly 93
S15.1 The effects of solvents on the assembly of $[Pd_2(E\text{-}\mathbf{1})_2(\mathbf{2})_2](BAr_F)_4$ 93
S15.2 The effects of solvents on the assembly of $[Pd_2(E\text{-}\mathbf{1})_4](BArF)_4$ 94
S15.3 The effects of solvents on the assembly of $[Pd_3(\mathbf{2})_6](BAr_F)_6$ 95

S16. The effect of 3-chloropyridine (Py*) on the self-assembly of $[Pd_2(E\text{-}\mathbf{1})_2(\mathbf{2})_2](BAr_F)_4$ in DCM:MeCN (10:1) 96
S16.1 Photoswitching of $[Pd_2(E\text{-}\mathbf{1})_2(\mathbf{2})_2](BAr_F)_4$ in the presence of 3-chloropyridine in DCM:MeCN (10:1) 96
S16.2 Photoswitching of $[Pd_2(E\text{-}\mathbf{1})_2(\mathbf{2})_2](BAr_F)_4$ without Py* in DCM:MeCN (10:1) 97

S17. Guest binding studies in heteroleptic cage $[Pd_2(E\text{-}\mathbf{1})_2(\mathbf{2})_2](BAr_F)_4$ in 11:1 DCM:MeCN 98

S17.1 Binding of methyl vinyl ketone by $[Pd_2(E\text{-}\mathbf{1})_2(\mathbf{2})_2](BAr_F)_4$ 99
S17.2 Binding of benzoyl nitromethane by $[Pd_2(E\text{-}\mathbf{1})_2(\mathbf{2})_2](BAr_F)_4$ 100
S17.3 Binding of 18-crown-6 by $[Pd_2(E\text{-}\mathbf{1})_2(\mathbf{2})_2](BAr_F)_4$ 101
S17.4 Binding of triflate (OTf^-) by $[Pd_2(E\text{-}\mathbf{1})_2(\mathbf{2})_2](BAr_F)_4$ 102

S18. Catalyzing the Michael addition between methyl vinyl ketone and benzoyl nitromethane 103
S18.1 Preparation of stock solutions for catalysis samples 103
S18.2 Preparation of self-assembled species samples for catalysis 103
S18.3 Monitoring conversion of benzoyl nitromethane to the Michael addition product using ^1H NMR spectroscopy 106
S18.3.1 Determining T_1 values for benzoyl nitromethane and the Michael addition product in DCM:MeCN (11:1) 106
S18.4 Monitoring the catalysis of the Michael addition between methyl vinyl ketone and benzoyl nitromethane in DCM:MeCN (11:1) using ^1H NMR spectroscopy 109
S18.4.1 Calculating conversion of benzoyl nitromethane to Michael addition product 109
S18.4.2 Using $[Pd_2(E\text{-}\mathbf{1})_2(\mathbf{2})_2](BAr_F)_4$ to catalyse the Michael addition between methyl vinyl ketone and benzoyl nitromethane 111
S18.4.3 Using $[Pd_2(E\text{-}\mathbf{1})_4](BAr_F)_4$ to catalyse the Michael addition between methyl vinyl ketone and benzoyl nitromethane 112
S18.4.4 Using $[Pd_3(\mathbf{2})_6](BAr_F)_6$ to catalyse the Michael addition between methyl vinyl ketone and benzoyl nitromethane 114
S18.4.5 Using $[Pd(Py^*)_4](BAr_F)_4$ to catalyse the Michael addition between methyl vinyl ketone and benzoyl nitromethane 116
S18.4.6 Michael addition between methyl vinyl ketone and benzoyl nitromethane with no catalyst 117

S19. Controlling catalysis with light using $[Pd_2(E\text{-}1)_2(2)_2](BAr_F)_4$ in DCM/MeCN (11:1) 118
S19.1 Preparation of stock solutions for catalysis 118
S19.2 Deactivating catalysis using 530 nm light 118
S19.3 Activating catalysis using 405 nm light 120
S19.4 Multiple switching cycles, followed by catalysis 121
S19.5 Deactivating catalysis by binding OTf in the cavity of the cage 123

S20. References 124

S1. General Experimental

S1.1 General Experimental

Reagents and solvents were purchased from Sigma-Aldrich, Merck, Chem Supply, Combi-Blocks or Alfa Aeser, and were used without purification unless stated otherwise.

Anhydrous solvents were dispensed from a Inertcorp solvent purification system (model number: PS-MD-7).

Flash chromatography was performed on the Biotage® Selekt System using Biotage® Sfär high-capacity silica columns.

NMR spectroscopy was performed using a Bruker Avance III 400 with a Prodigy CryoProbe, a Bruker Avance III 500, a Bruker Avance III 600 or a Bruker Avance III HD 600 with a TCI CryoProbe. All chemical shifts were calibrated against residual solvent signals. All coupling constants (*J*) are reported in Hertz. Signals in the NMR spectra are reported as broad (br), singlet (s), doublets (d), triplets (t), quartets (q), quintets (qu), sextets (sx), septets (sept), or unclear multiplets (m). NMR spectra were processed with MestReNova 14.1.2 software. All NMR data are assigned unambiguously, except where specified. The broad peak observed in ^{19}F spectra between −150 and −210 ppm corresponds to a Teflon matrix, which is a standard component of all Bruker probes.

UV-vis experiments were performed on an Agilent Cary 60 Bio UV-visible Spectrophotometer equipped with a customized Cary Single Cell Peltier Accessory, keeping the samples at 25 °C unless stated otherwise. The cell holder was modified to allow for irradiation perpendicular to the direction of measurement. A Luxeon Rebel LED was mounted on a heat sink 4 cm from the cell and driven using a 1000 mA LuxDrive PowerPuck. The sample was analyzed in a quartz cuvette with a path length of 1 cm. All samples were stirred to ensure homogeneity Recording UV-vis absorption spectra of cage samples proved difficult as high concentrations (mM) are required for cage assembly in coordinating solvents such as DMSO or acetonitrile. Such conditions are not compatible with even short (2 mm) pathlengths cuvettes used for UV-vis as the absorption is too high to accurately measure, and if the sample is diluted the cage composition can no longer be verified by NMR spectroscopy.

High-resolution mass spectrometry (HR-MS) experiments were performed on a hybrid linear quadrupole ion trap mass spectrometer (Thermo LTQ Orbitrap XL) equipped with an external nanospray ionisation (NSI) source.

Micheal addition substrates were purified before use: methyl vinyl ketone was dried over K_2CO_3 and $CaCl_2$ and distilled at 30 °C under reduced pressure; benzoyl nitromethane was recrystallized from isopropanol.

Table S1 Specifications for the LEDs used in this work.

LED Part Number (supplier)	Dominant wavelength / nm
LCS-0405-12-xx (Mightex)	405
LXML-PB01-0040 (LumiLEDs)	470
LXML-PM01-0100 (LumiLEDs)	530

S2. Synthesis of photoswitchable ligand 1

S2.1 Synthetic overview

Photoswitchable ligand **1** was synthesized over 2 steps from commercially available 3–bromo–2,6–difluoroaniline. 3-Ethynyl pyridine was coupled with 3–bromo–2,6–difluoroaniline via Sonogashira coupling to afford intermediate product **3** as a white solid in 80% yield. Ligand **1** was synthesized by coupling 2 equivalents of **3** using *N*-chlorosuccinimide (NCS) and 1,8-diazabicyclo[5.4.0]undec-7-ene (DBU).[1] This afforded ligand **1** as a mixture of E/Z isomers in 33% yield.

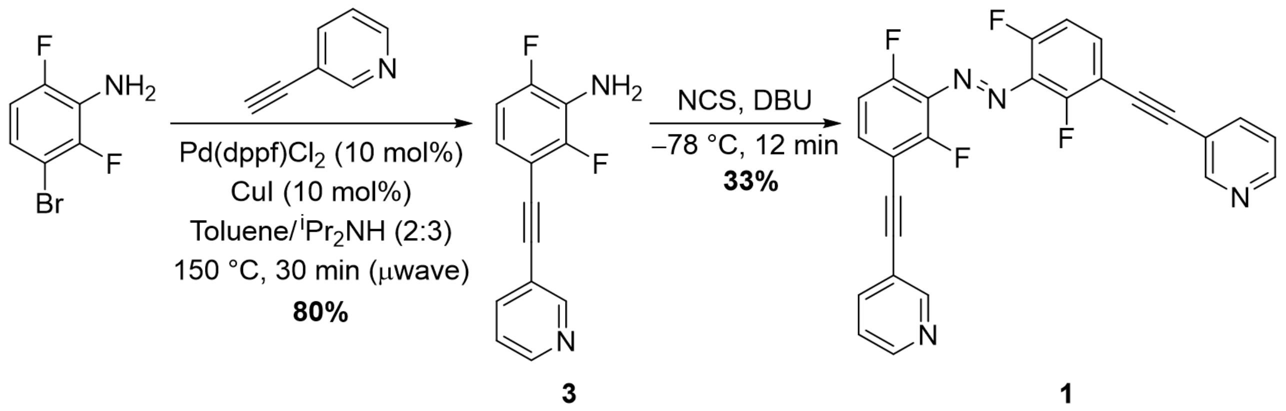

Scheme S1. Synthetic pathway for photoswitchable ligand **1**.

S2.2 Synthesis and characterization of intermediate 3

S2.2.1 Synthesis of intermediate 3

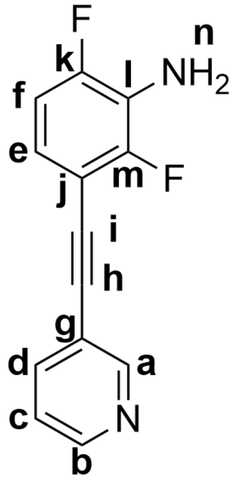

3

3-Bromo-2,6-difluoroaniline (500 mg, 2.4 mmol, 1 equiv.) and 3-ethynlpyridine (745 mg, 7.2 mmol, 3 equiv.) were dissolved in toluene (8 mL) and diisopropylamine (12 mL) in a 30 ml microwave sample vial. The solution was degassed with argon for 10 min, then CuI (45 mg, 240 µmol, 0.1 equiv.), and Pd(dppf)Cl_2 (175 mg, 240 µmol, 0.1 equiv.) were added. The reaction mixture was heated at 150 °C for 30 min in a microwave reactor. The reaction mixture was filtered through Celite. The Celite was washed with DCM (100 mL), then the organic phase was collected and washed with water (3 × 100 mL). The organic phase was dried over $MgSO_4$ and filtered through a filter paper. The solvent was removed under reduced pressure and the residue was purified using flash column chromatography (silica, 0-1% methanol in DCM) to afford the product as a white solid. (443 mg, 1.92 mmol, 80 %)

^{1}H NMR (600 MHz, DMSO-d_6) δ 8.74 (dd, J = 2.1, 0.7 Hz, 1H, H^a), 8.60 (dd, J = 4.8, 1.6 Hz, 1H, H^b), 7.97 (dt, J = 7.9, 1.9 Hz, 1H, H^d), 7.47 (ddd, J = 7.9, 4.9, 0.7 Hz, 1H, H^c), 6.99 (ddd, J = 10.2, 8.5, 1.2 Hz, 1H, H^f), 6.83 – 6.77 (m, 1H, H^e), 5.48 (s, 2H, H^n).

^{19}F NMR (565 MHz, DMSO-d_6) δ -126.97 (ddd, J = 17.0, 10.6, 5.9 Hz, F^m), -127.34 (dd, J = 17.6, 7.5 Hz, H^k).

^{13}C NMR (151 MHz, DMSO-d_6) δ 152.5 (dd, $J_{C\text{-}F}$ = 95.8, 10.2 Hz, C^k), 152.0 (C^a), 150.9 (dd, $J_{C\text{-}F}$ = 94.1, 8.8 Hz, C^m), 149.7 (C^b), 139.0 (C^d), 126.7 (dd, $J_{C\text{-}F}$ = 16.5 Hz, C^l), 124.2 (C^c), 119.6 (C^g), 118.4 (d, $J_{C\text{-}F}$ = 8.3 Hz, C^e), 111.9 (d, $J_{C\text{-}F}$ = 17.0 Hz, C^f), 106.6 (d, $J_{C\text{-}F}$ = 14.8 Hz, (C^j), 90.2 (C^h), 86.2 (C^i).

ESI-MS (m/z): 231.0731 $[M + H]^+$ (calculated: 231.0734)

S2.2.2 1D and 2D NMR spectra of 3 in DMSO-d_6

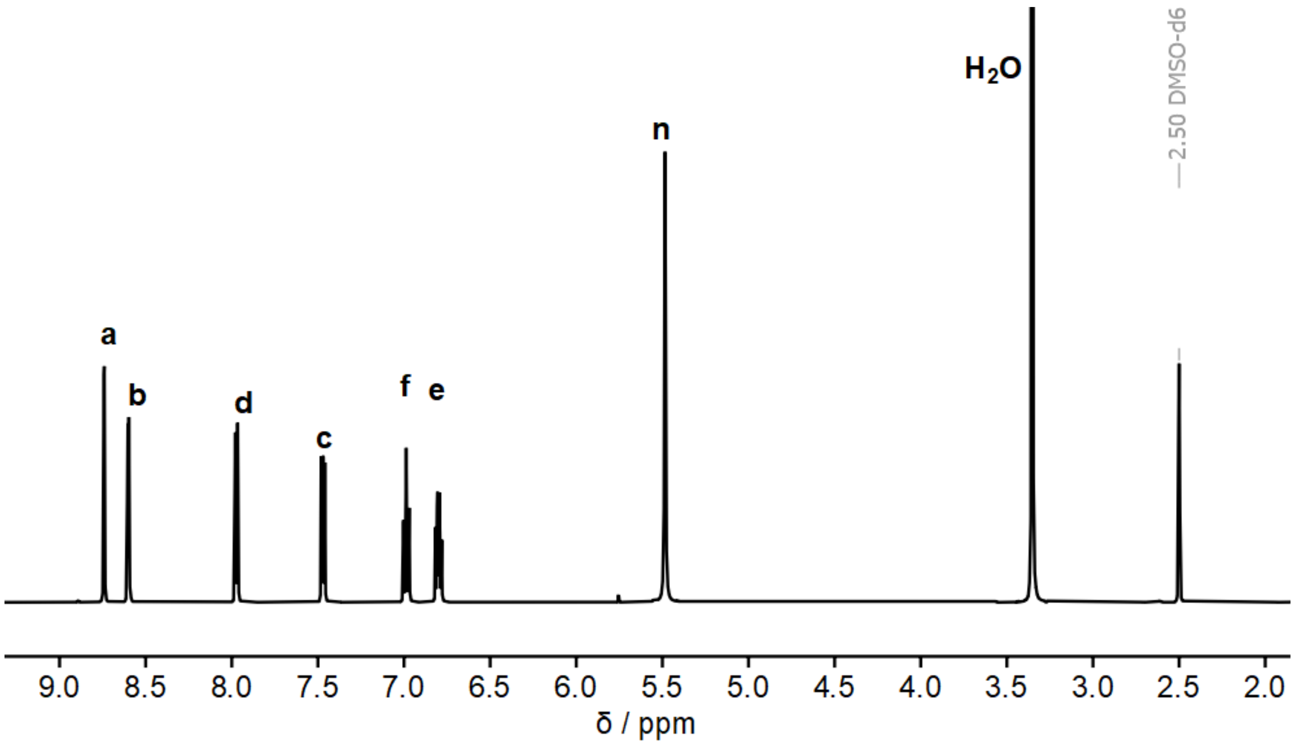

Figure S1. ^{1}H NMR (600 MHz, 298 K, DMSO-d_6) spectrum of intermediate **3**.

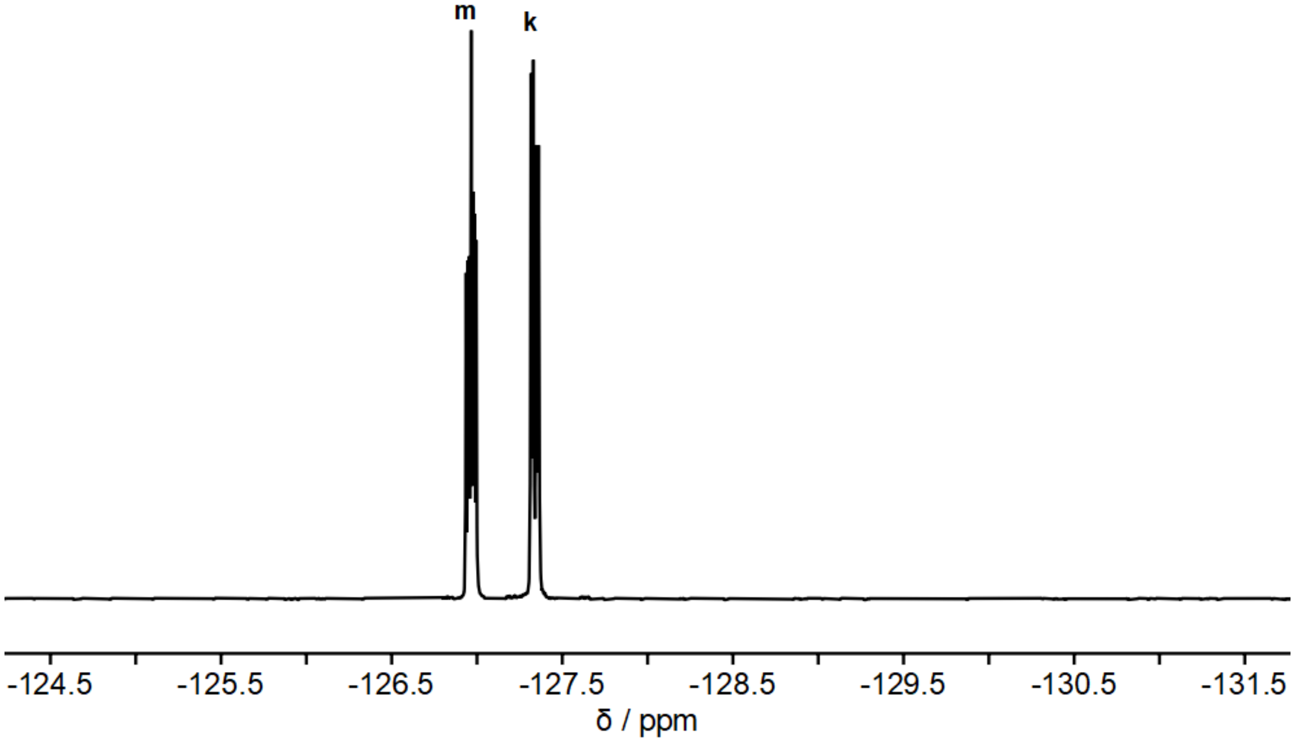

Figure S2. Partial ^{19}F NMR (565 MHz, 298 K, DMSO-d_6) spectrum of intermediate **3**.

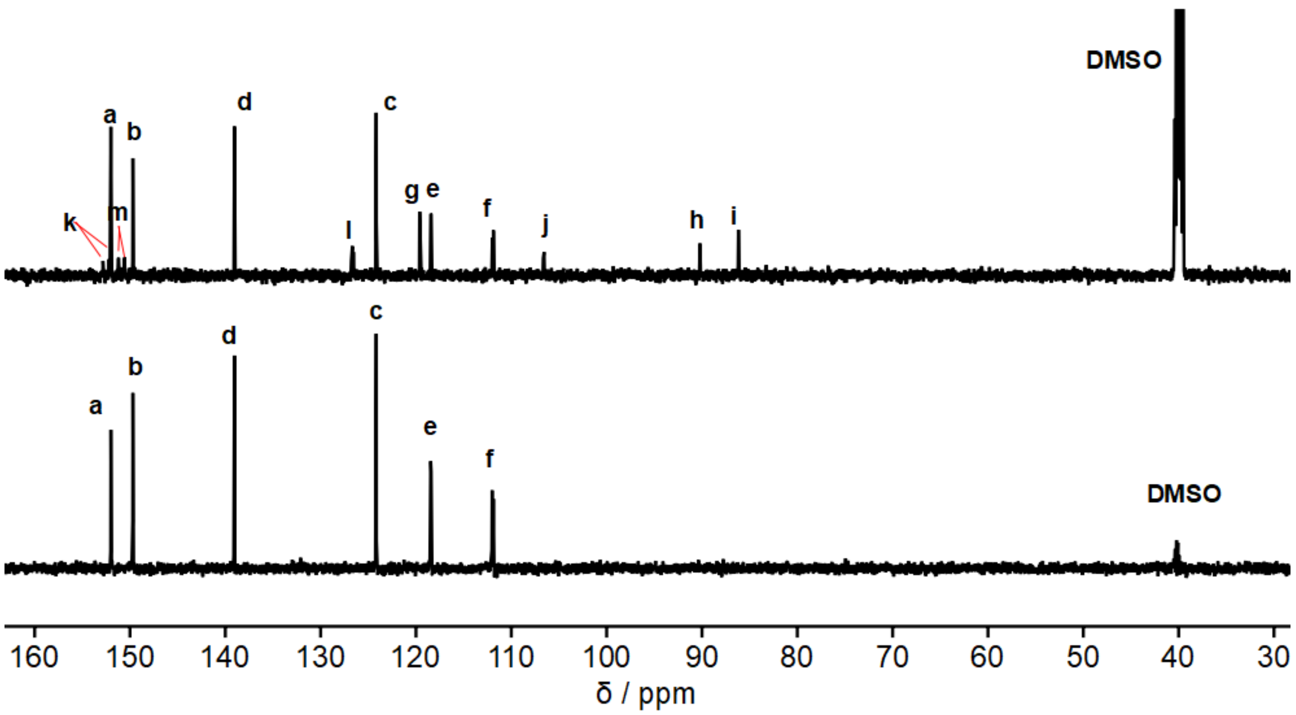

Figure S3. $^{13}C\{^{1}H\}$ (top) and DEPT-135 (bottom) NMR (151 MHz, 298 K, DMSO-d_6) spectrum of intermediate **3**.

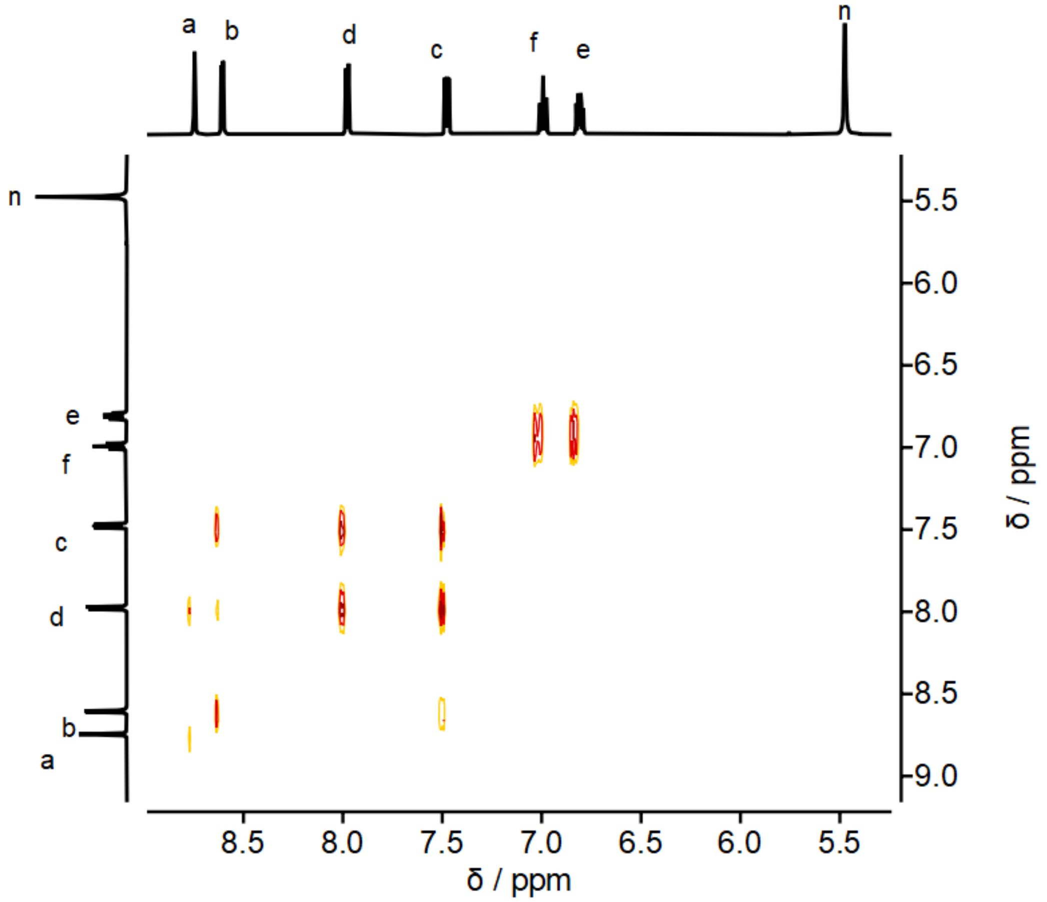

Figure S4. ^{1}H-^{1}H COSY NMR (600 MHz, 298 K, DMSO-d_6) spectrum of intermediate **3**.

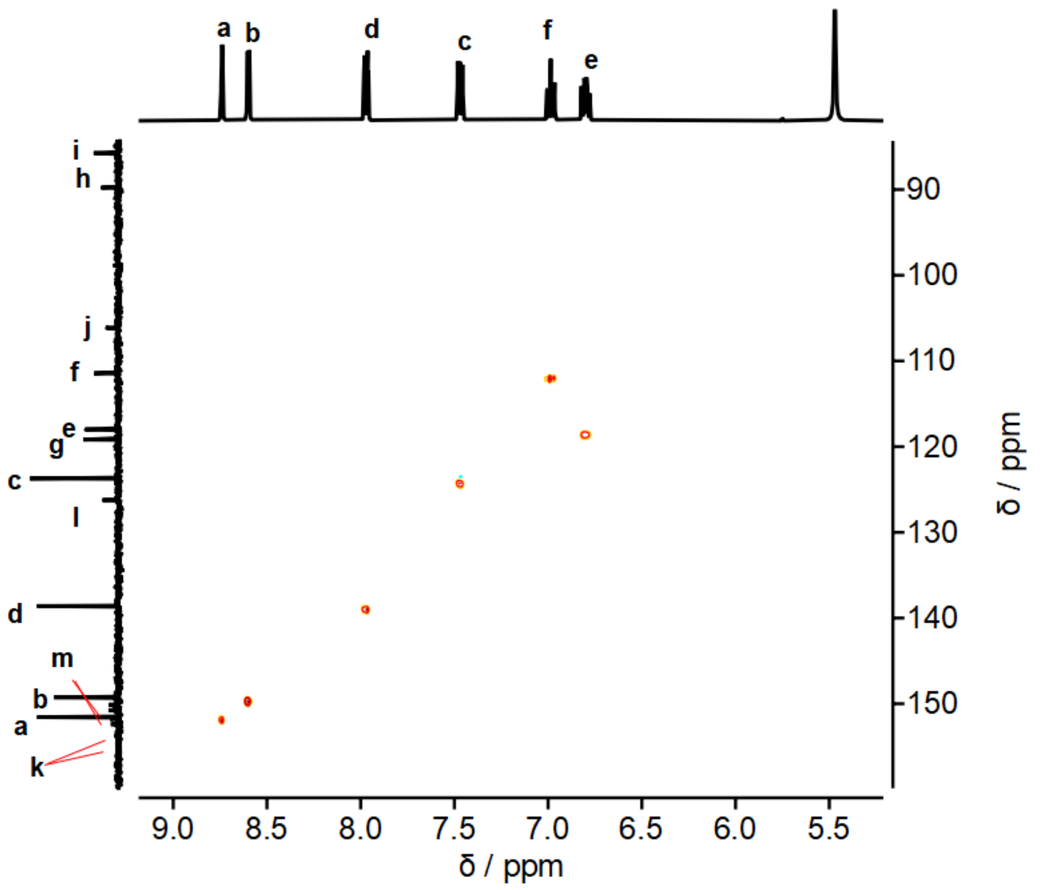

Figure S5. ^{1}H-^{13}C HSQC NMR (500 MHz, 126 MHz, 298 K, DMSO-d_6) spectrum of intermediate **3.**

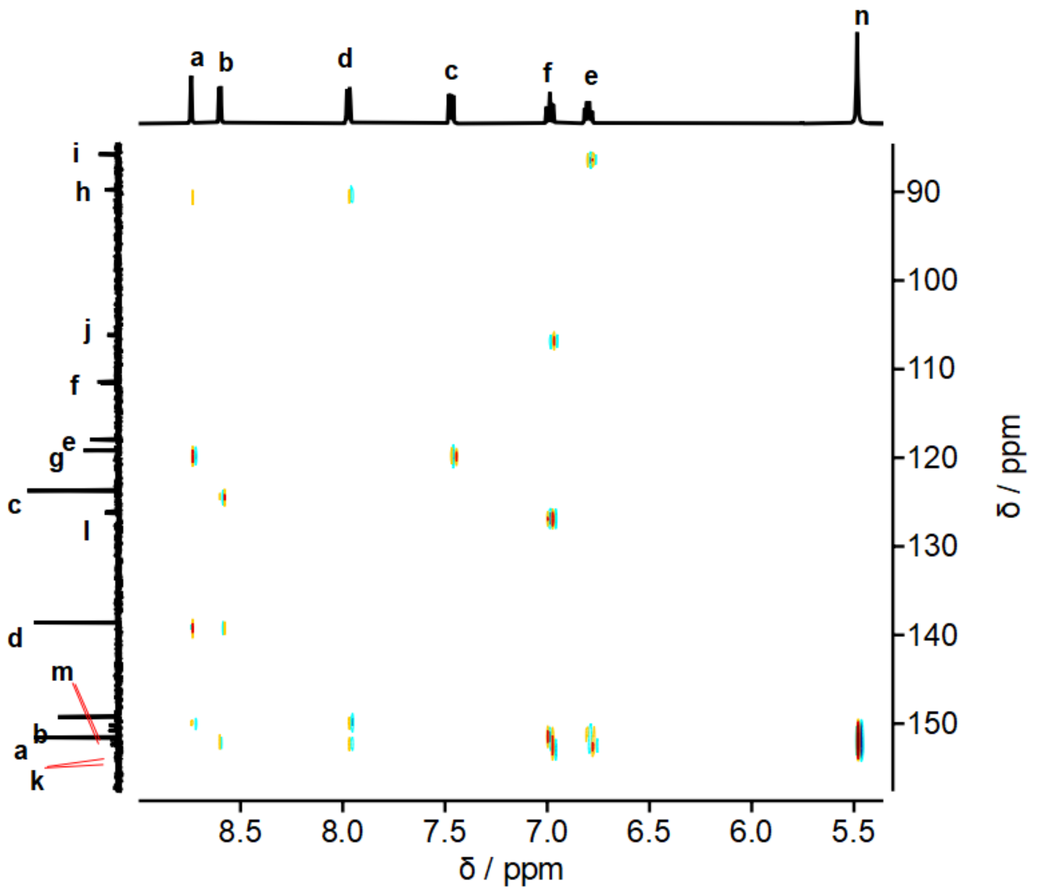

Figure S6. ^{1}H-^{13}C HMBC NMR (600 MHz, 151 MHz, 298 K, DMSO-d_6) spectrum of intermediate **3.**

S2.3 Synthesis and characterization of photoswitchable ligand 1

S2.3.1 Synthesis of ligand 1

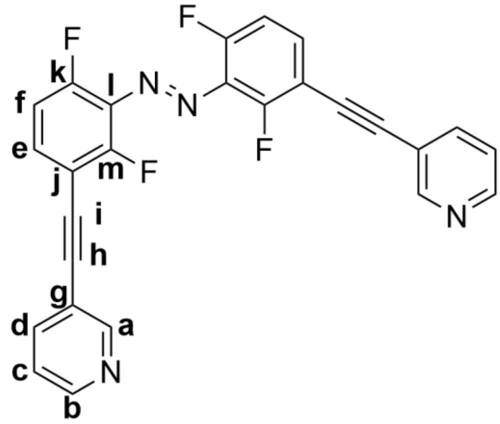

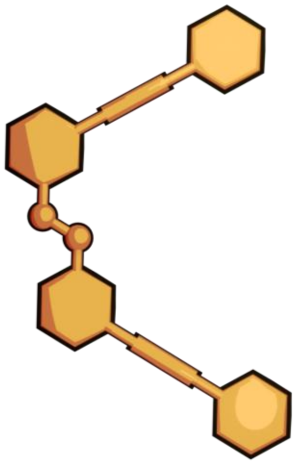

1

Ligand **1** was synthesised following a modified procedure[1].

Product **3** (100 mg, 0.43 mmol, 2 equiv.) was dissolved in DCM (6.5 mL). DBU (135 µL, 0.87 mmol, 4 equiv.) was added and the solution was stirred at rt for 5 min. The mixture was cooled to −78 °C. NCS (116 mg, 0.87 mmol, 4 equiv.) was added as a solid at once. The reaction mixture was stirred at −78 °C for 12 min before being removed from the dry ice/acetone bath. Saturated solution of aqueous $NaHCO_3$ was added to quench the reaction. The organic layer was collected and washed with saturated aqueous $NaHCO_3$ solution and with water. The organic phase was collected, dried over $MgSO_4$, filtered through Kim tech tissue, then the solvent was removed. The product was purified by flash chromatography (SiO_2, 2% MeOH/DCM) to afford the product as a red solid (33 mg, 0.07 mmol, 33%).

^{1}H NMR (600 MHz, DMSO-d_6) δ 8.81 (dd, J = 2.2, 0.9 Hz, 2H, H^a), 8.64 (dd, J = 4.8, 1.7 Hz, 2H, H^b), 8.04 (d, J = 8.0 Hz, 2H, H^d), 7.95 (dt, J = 10.0, 6.3 Hz, 2H, H^e), 7.60 – 7.38 (m, 4H, H^c+H^f).

^{19}F NMR (565 MHz, DMSO-d_6) δ −117.66 (d, J = 7.3 Hz, 2F, F^m), −117.83 – −117.99 (m, 2F, F^k)

^{13}C NMR (151 MHz, DMSO-d_6) δ 155.6 (d, $J_{C\text{-}F}$ = 36.9 Hz, C^k), 153.9 (d, $J_{C\text{-}FC}$ = 39.3 Hz, C^m), 151.6 (C^a), 149.6 (C^b), 138.7 (C^d), 136.3 (C^e), 130.7 (C^l), 123.7 (C^c), 118.5 (C^g), 113.9 (d, J = 22 Hz, C^F), 108.4 (d, J = 15 Hz, C^j), 91.9 (C^h), 83.7 (C^i).

ESI-MS (m/z): 457.1072 $[M + H]^+$ (calculated: 457.1076)

S2.3.2 1D and 2D NMR spectra of 1 in DMSO-d_6

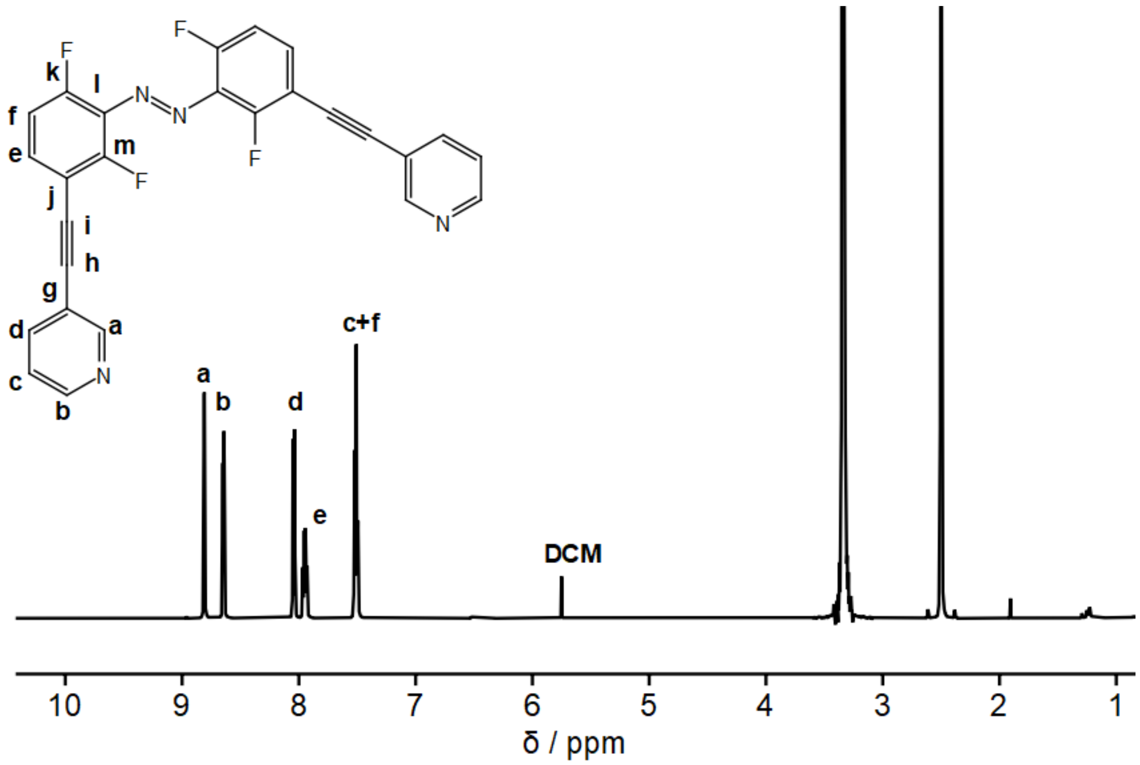

Figure S7. ^{1}H NMR (600 MHz, 298 K, DMSO-d_6) spectrum of ligand **1**.

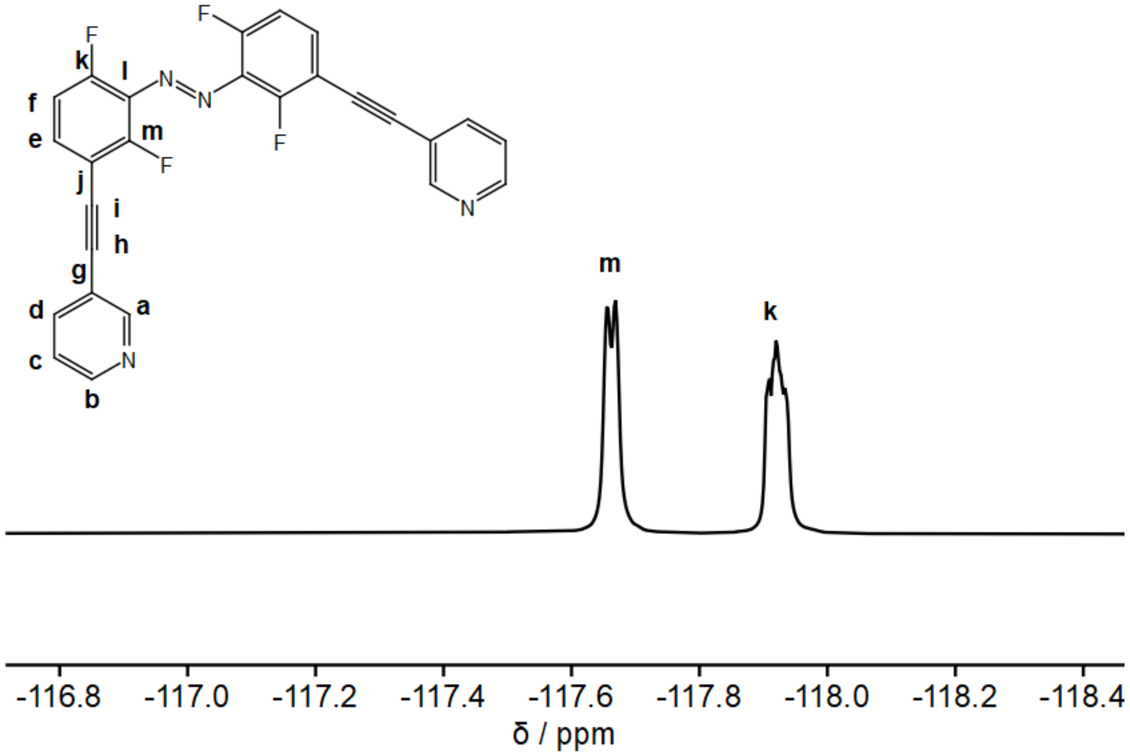

Figure S8. Partial ^{19}F NMR (565 MHz, 298 K, DMSO-d_6) spectrum of ligand **1**.

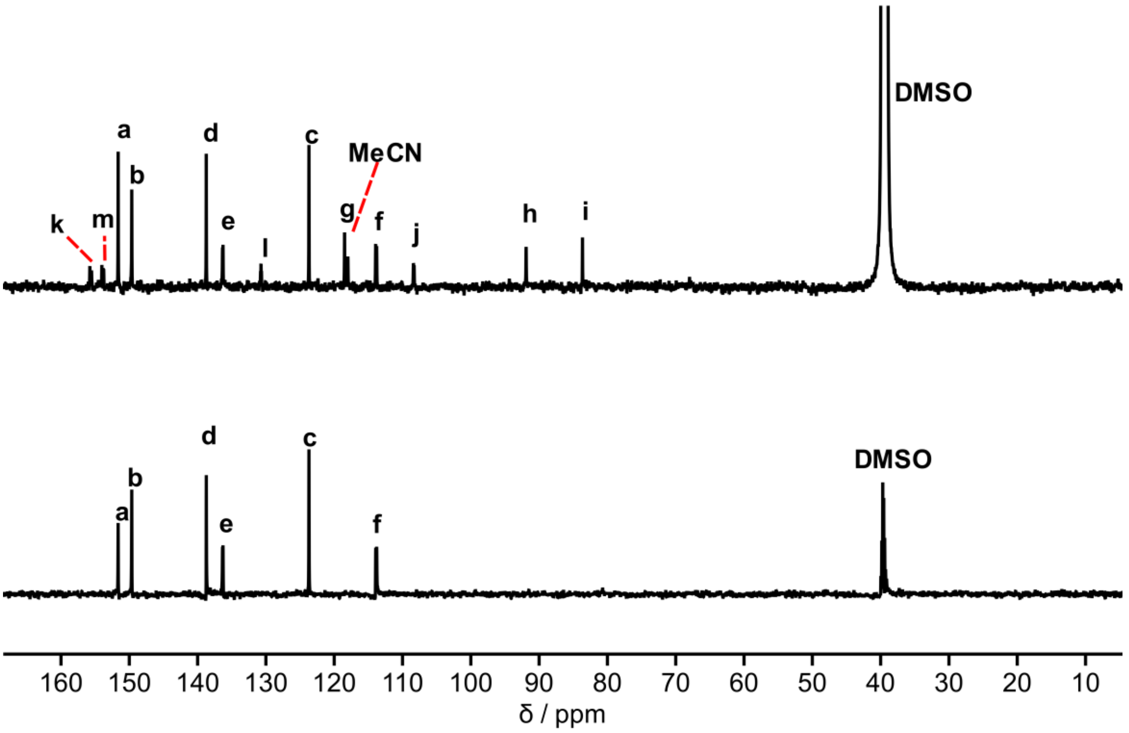

Figure S9. $^{13}C\{^1H\}$ and 135-DEPT NMR (151 MHz, 298 K, DMSO-d_6) spectrum of ligand **1**.

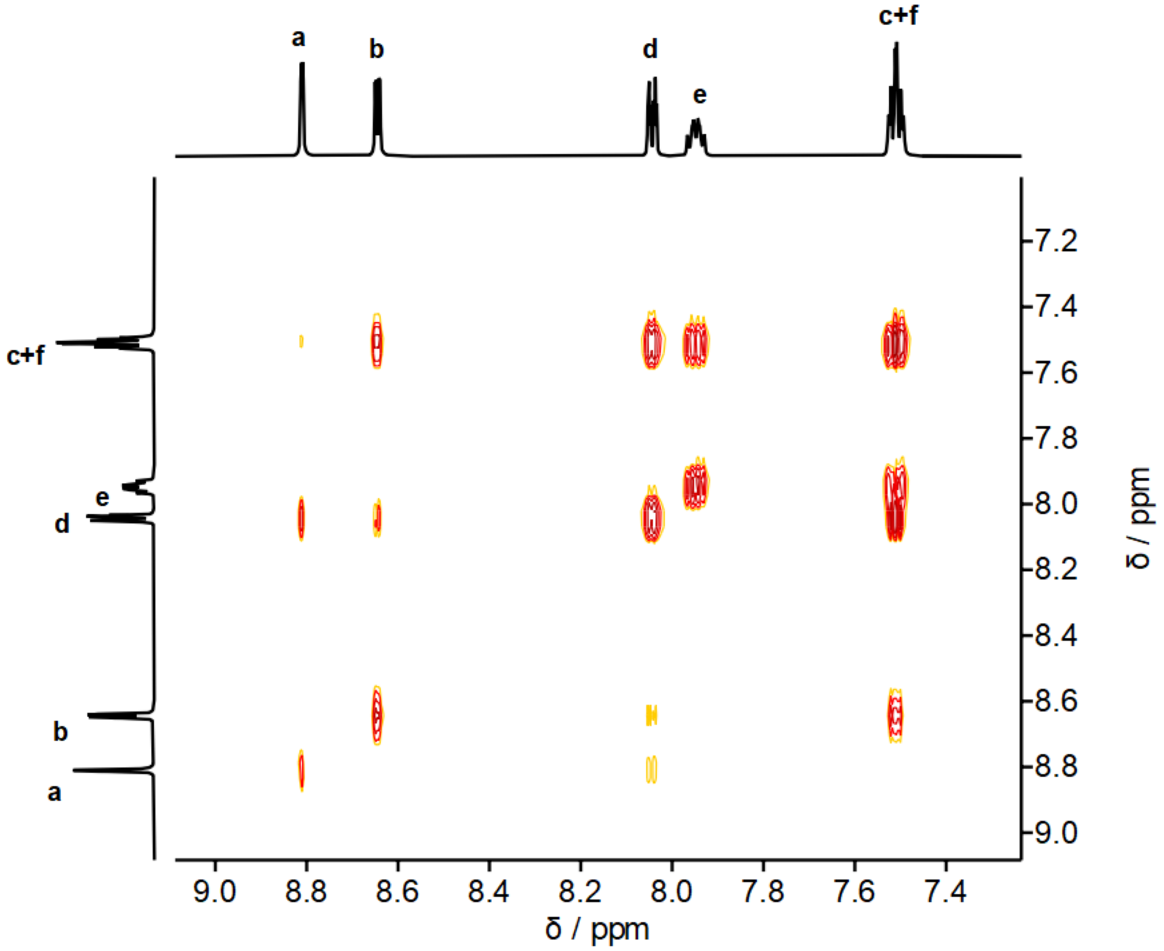

Figure S10. ^1H-^1H COSY NMR (600 MHz, 298 K, DMSO-d_6) spectrum of ligand **1**.

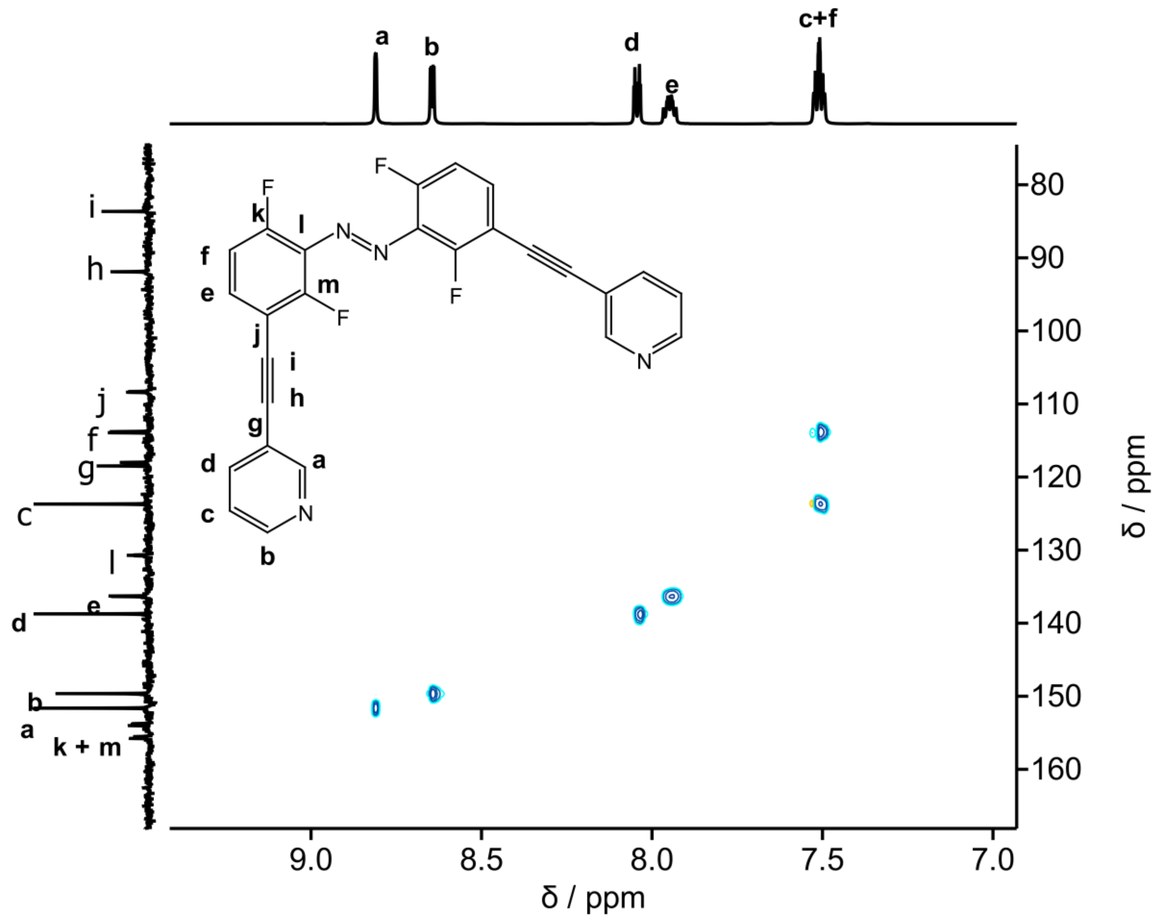

Figure S11. ^{1}H-^{13}C HSQC NMR (600 MHz, 151 MHz, 298 K, DMSO-d_6) spectrum of ligand **1**.

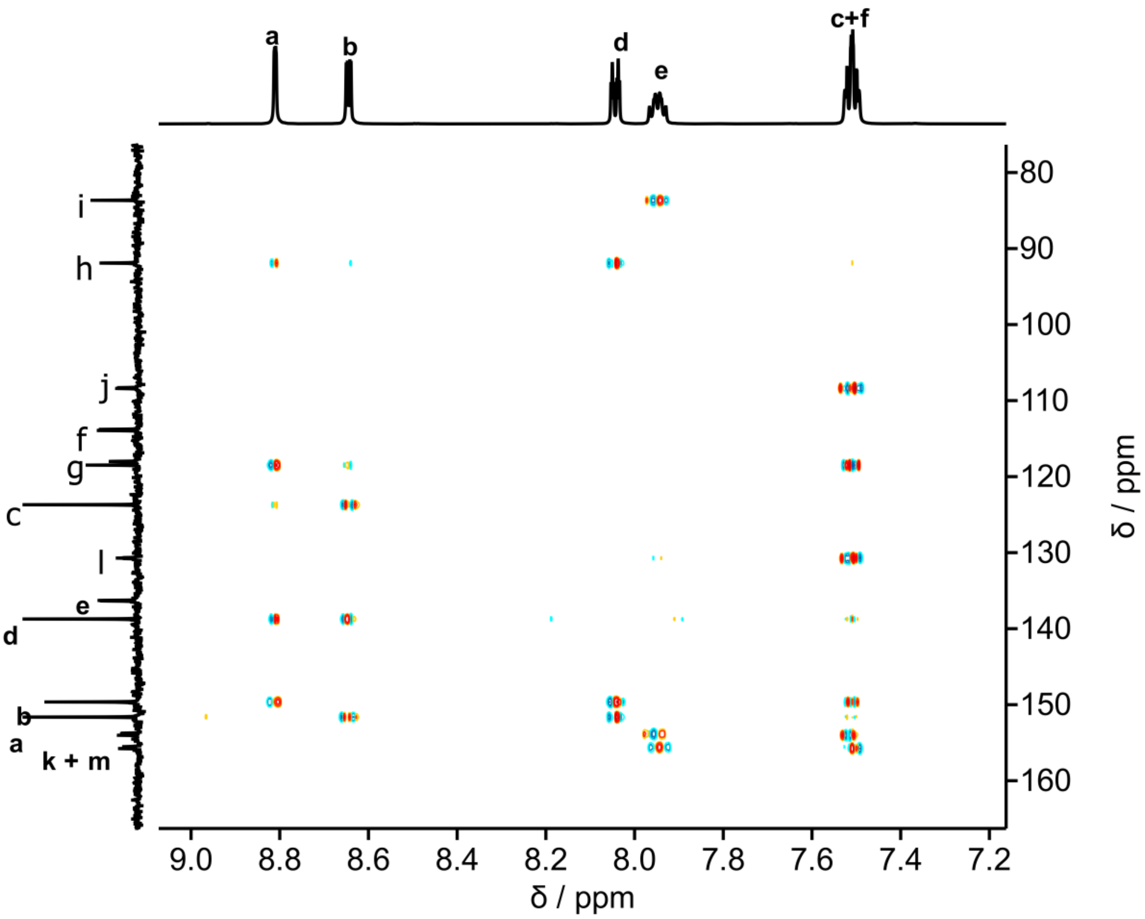

Figure S12. ^{1}H-^{13}C HMBC NMR (600 MHz, 151 MHz, 298 K, DMSO-d_6) spectrum of ligand **1**.

S2.4 Single crystal X-ray structure of *E*-1 (CCDC: 2343887)

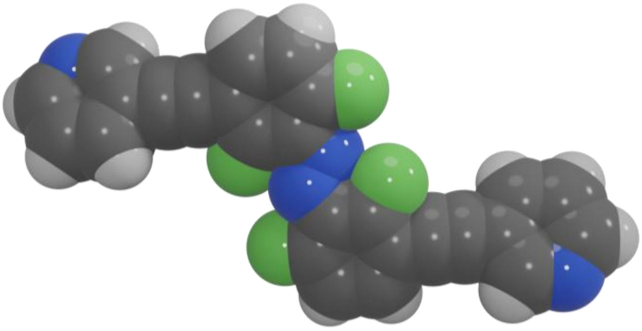

Figure S13. Single crystal X-ray structure of ligand *E*-**1.** Color codes: grey: carbon; white: hydrogen; blue: nitrogen; and green: fluorine.

Crystals suitable for X-ray crystallography were grown by the slow evaporation of a solution of *E*-**1** in acetonitrile. A needle-like single crystal with dimensions of 0.012 × 0.015 × 0.08 mm was selected under the polarizing microscope, and it was then mounted on a MicroMount, consisting of a thin polymer tip with a wicking aperture. X-ray diffraction data were collected at 100 K on the MX1[2] Macromolecular Crystallography beamline at the Australian Synchrotron at a wavelength of 0.71073 Å. The data collection and integration were performed within the AS QEGUI and XDS[3] software programs. The solutions were obtained by intrinsic phasing using SHELXT[4] followed by successive refinements using full matrix least squares method against F^2 using SHELXL-2018/3.[5] The program OLEX2[6] was used was used as a graphical SHELX interface. All non-hydrogen atoms were refined with anisotropic thermal parameters, with hydrogen atoms being added geometrically and refined using riding thermal parameters. Due to the software set up at Australian Synchrotron, absorption correction and transmission factors are not reported.

PLAT058_ALERT_1_A Maximum Transmission Factor Missing.

- Value not reported by XDS.

PLAT059_ALERT_1_A Minimum Transmission Factor Missing.

- Value not reported by XDS.

The above alerts are present as an absorption correction was not done. This is specified in the CheckCIF procedure for PLAT058 and PLAT059 "The Maximum transmission factor should be specified in the case a correction for absorption was done."[7]

Table S2. Crystal data and structure refinement for *E*-**1**.

Crystallographic details	*E*-**1**
Identification code	Ligand_1
Empirical formula	$C_{26}H_{12}F_4N_4$
Formula weight	456.40
Temperature/K	100.00
Crystal system	monoclinic
Space group	$P2_1/c$
a/Å	10.910(2)
b/Å	4.6300(9)
c/Å	19.610(4)
α/°	90
β/°	90.22(3)
γ/°	90
Volume/Å^3	990.6(3)
Z	2
ρ_{calc}g/cm^3	1.530
μ/mm^{-1}	0.119
F(000)	464.0
Crystal size/mm^3	0.08 × 0.015 × 0.012
Radiation	Synchrotron (λ = 0.71073)
2Θ range for data collection/°	1.867 to 28.661
Index ranges	-14 ≤ h ≤ 14, -6 ≤ k ≤ 6, -25 ≤ l ≤ 26
Reflections collected	33314
Independent reflections	2276 [R_{int} = 0.0422, R_{sigma} = 0.0180]
Data/restraints/parameters	2276/0/154
Goodness-of-fit on F^2	1.046
Final R indexes [I>=2σ (I)]	R_1 = 0.0494, wR_2 = 0.1411
Final R indexes [all data]	R_1 = 0.0519, wR_2 = 0.1445
Largest diff. peak/hole / e Å^{-3}	0.27/-0.32

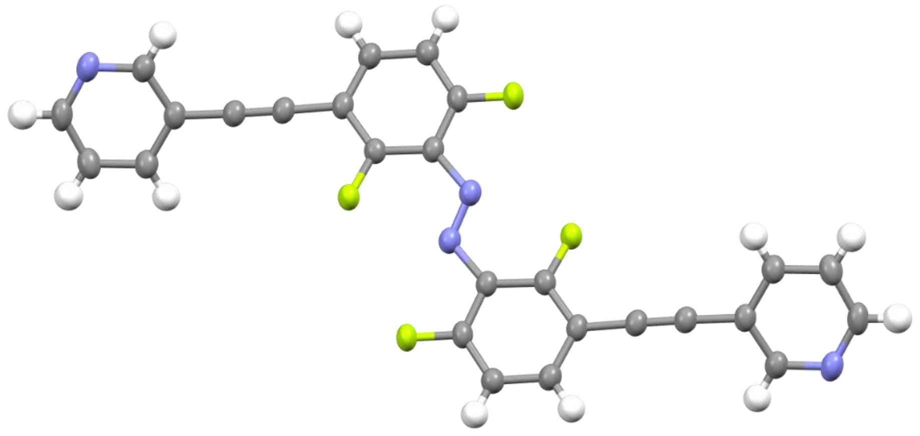

Figure S14. ORTEP representation of ligand *E*-**1**. Ellipsoids are drawn at 50% probability.

S3. Photoswitching properties of ligand 1

S3.1 Determining the PSS distributions of ligand 1 in DMSO-d_6 using ^{1}H and ^{19}F NMR spectroscopy

^{1}H and ^{19}F NMR spectroscopy was used to determine the distribution of *E*-**1** and *Z*-**1** after generating PSSs with different irradiation wavelengths. A sample of ligand **1** in DMSO-d_6 was heated with a heat gun at 150 °C for 3 min to thermally equilibrate the sample so that only *E*-**1** was present (Figure S15i). The sample was irradiated with a 530 nm LED for one 10 min period (Figure S15ii), then another 10 min period (Figure S15iii). The signal intensities in the NMR spectra did not change after the second irradiation period, indicating that a PSS was generated within 10 min. The sample was irradiated with a 405 nm LED for one 10 min period (Figure S15iv), then another 10 minutes period (Figure S15v). After the second 405 nm irradiation period, the signal intensities for *E*-**1** increased by 2-4% and the signal intensities for *Z*-**1** decreased by 1-4%. This change in signal intensity indicates that the PSS was not generated within the first irradiation period. The sample was irradiated with a 470 nm LED for a 10 min period (Figure S15vi), then irradiated for another 5 min period (Figure S15vii). The signal intensities in the NMR spectra did not change after the second irradiation period, indicating that a PSS was generated within 10 min.

To determine the distribution of *E*-**1** and *Z*-**1** at each PSS, we measured the NMR signal intensities for environments H^a, H^e, F^k, and F^m. These environments were selected as the signals for these environments do not overlap with other signals. For each environment, after each irradiation period, the signal intensities for *E*-**1** and *Z*-**1** were measured and the percentage of each isomer was calculated. For clarity, the signal intensities and the isomer percentages are tabulated in Table S3. The isomer percentages were averaged to determine the PSS distribution at PSS_{405} (86:14 *E*:*Z*), PSS_{530} (12:88 *E*:*Z*), and PSS_{470} (43:57 *E*:*Z*) (Table S4). The percentage values after irradiating with 405 nm light for 10 min were not used to determine the isomer distribution at PSS_{405}, as the sample had not reached a PSS.

We demonstrated that selective PSS can be generated for *E*-**1** and *Z*-**1**. The PSS selectivity of ligand **1** is higher than previously reported *o*-fluoroazobenzene derived ligands.[8, 9]

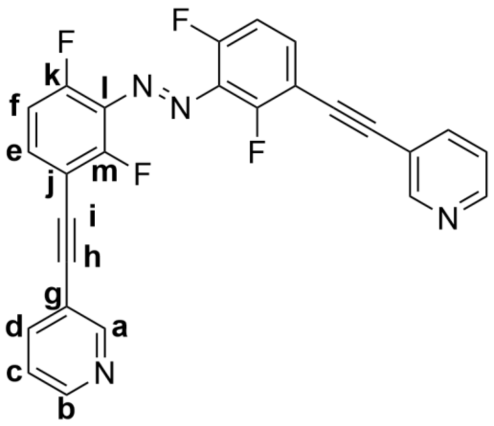

1

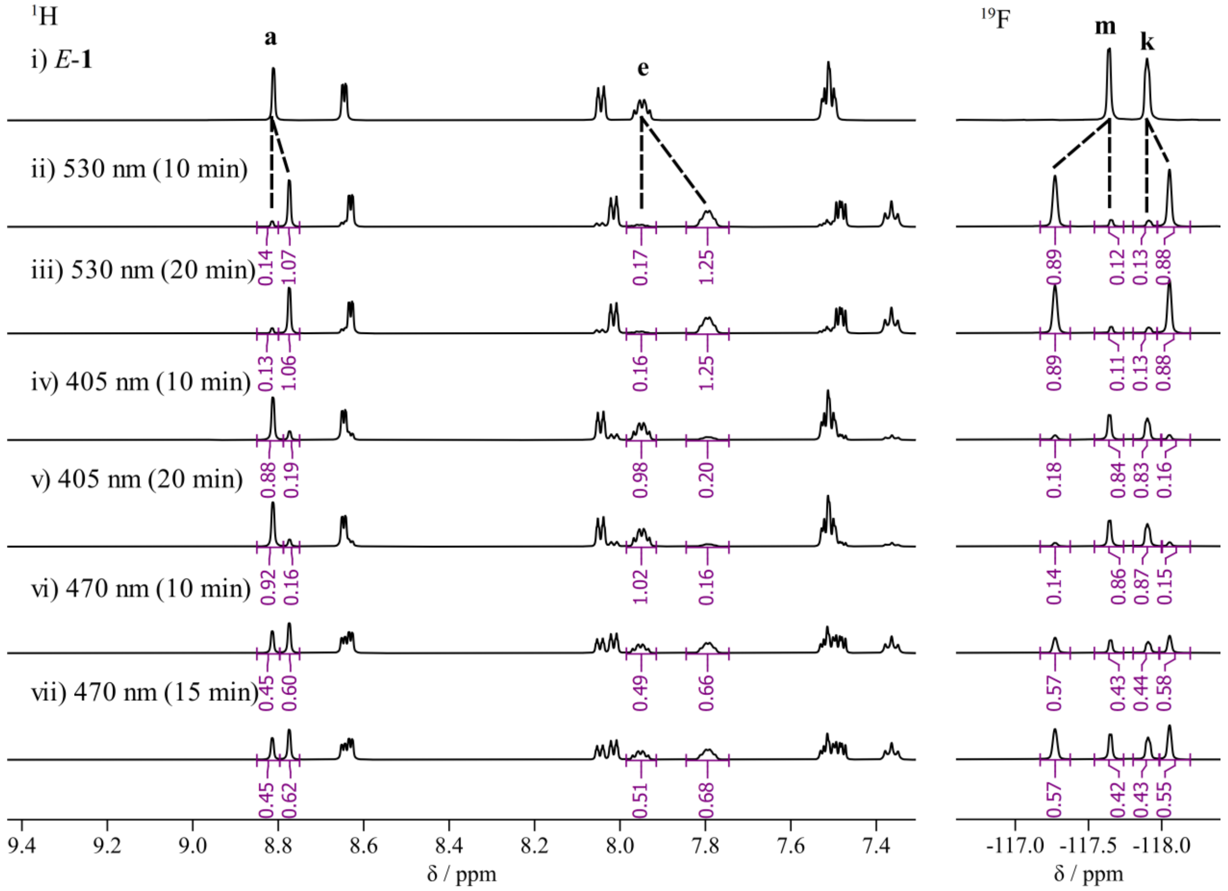

Figure S15. ^{1}H (600 MHz, 298 K, DMSO-d_6) and ^{19}F (575 MHz, 298 K, DMSO-d_6) NMR spectra of ligand **1** after i) thermal equilibration, ii) irradiation with a 530 nm LED for 10 min, iii) irradiation with a 530 nm LED for 20 min, iv) irradiation with a 405 nm LED for 10 min, v) irradiation with a 405 nm LED for 20 min, vi) irradiation with a 470 nm LED for 10 min, vii) irradiation with a 470 nm LED for 15 min.

Table S3. ^{1}H (600 MHz, 298 K, DMSO-d_6) and ^{19}F (575 MHz, 298 K, DMSO-d_6) NMR signal intensities and calculated isomer percentages for ligand **1** in DMSO after irradiating with different wavelengths.

**Environment H[a]**				
Irradiation	*E*-**1** (8.81 ppm)	*Z*-**1** (8.77 ppm)	%*E*	%*Z*
530 nm (10 min)	0.14	1.07	12%	88%
530 nm (20min)	0.13	1.06	11%	89%
405 nm (10min)	0.88	0.19	82%	18%
405 nm (20 min)	0.92	0.16	85%	15%
470 nm (10 min)	0.45	0.6	43%	57%
470 nm (15 min)	0.45	0.62	42%	58%
**Environment H[e]**				
Irradiation	*E*-**1** (7.95 ppm)	*Z*-**1** (7.79 ppm)	%E	%Z
530 nm (10 min)	0.17	1.25	12%	88%
530 nm (20min)	0.16	1.25	11%	89%
405 nm (10min)	0.98	0.2	83%	17%
405 nm (20 min)	1.02	0.16	86%	14%
470 nm (10 min)	0.49	0.66	43%	57%
470 nm (15 min)	0.51	0.68	43%	57%
**Environment F[k]**				
Irradiation	*E*-**1** (−117.91 ppm)	*Z*-**1** (−118.05 ppm)	%E	%Z
530 nm (10 min)	0.13	0.88	13%	87%
530 nm (20min)	0.13	0.88	13%	87%
405 nm (10min)	0.83	0.16	84%	16%
405 nm (20 min)	0.87	0.15	85%	15%
470 nm (10 min)	0.44	0.58	43%	57%
470 nm (15 min)	0.43	0.55	44%	56%
**Environment F[m]**				
Irradiation	*E*-**1** (−117.65 ppm)	*Z*-**1** (−117.27)	%E	%Z
530 nm (10 min)	0.13	0.88	13%	87%
530 nm (20min)	0.13	0.88	13%	87%
405 nm (10min)	0.83	0.16	84%	16%
405 nm (20 min)	0.87	0.15	85%	15%
470 nm (10 min)	0.44	0.58	43%	57%
470 nm (15 min)	0.43	0.55	44%	56%

Table S4. Average PSS distributions of ligand **1** using different irradiation wavelengths.[a]

Irradiation wavelength / nm	%*E*-**1**	%*Z*-**1**
530	12%	88%
405	86%	14%
470	43%	57%

[a]PSS distributions determined from ^{1}H (600 MHz, 298 K, DMSO-d_6) and ^{19}F (575 MHz, 298 K, DMSO-d_6) NMR signal intensities for ligand **1** environments H[a], H[e], F[k], and F[m].

S3.2 Measuring UV-vis spectra of ligand 1 at different PSS in DMSO

To measure the UV-vis absorbance spectra of ligand **1**, we prepared a sample of ligand **1** in DMSO-d_6 and heated it with a heat gun at 150 °C for 3 min. ^{1}H and ^{19}F NMR spectroscopy was used to ensure the sample contained only E-**1**. The sample of E-**1** was added dropwise to a quartz cuvette filled with 2.5 mL of DMSO until the maximum absorbance of the sample was ~0.65 (Figure S16). To measure UV-vis absorbance spectra at different PSS, we irradiated the sample with a 405 nm LED, a 470 nm LED, then a 530 nm LED. For each irradiation wavelength we irradiated the sample until the absorbance stopped changing, which indicated that the PSS had been reached. For all irradiation wavelengths, a PSS was generated in <20 s.

We calculated the UV-vis absorbance spectrum of Z-**1** using the UV-vis absorbance spectra of ligand **1** and the PSS distributions of E-**1** and Z-**1** determined using ^{1}H and ^{19}F NMR spectroscopy (see section S3.1 for details). The absorbance of Z-**1** was calculated using the equation:

$$Abs_Z = \frac{Abs_{PSS} - (E \times Abs_E)}{Z}$$

Abs_Z = absorbance of the Z-isomer

Abs_{PSS} = absorbance at the relative PSS

E = relative abundance of the E-isomer

Abs_E = absorbance of the sample containing all E-isomer

Z = relative abundance of the Z-isomer

The absorbance of Z-**1** was calculated using the absorption spectra for PSS_{405}, PSS_{470}, and PSS_{530}. The calculated spectra were averaged, and the standard deviation was calculated and used to determine errors.

The π-π* excitation band for both E-**1** and Z-**1** overlap with another excitation band centred at 300 nm. The n-π* excitation band is centred at 459 nm for E-**1** and centred at 418 nm for Z-**1** (Table S5). A large separation between n-π* bands contribute to generating PSS distributions selective for the Z-isomer, as this introduces a region of wavelengths where only the E-isomer absorbs allowing the selective excitation the E-isomer. The separation between n-π* band between each isomer is significantly larger for **1** ($\Delta_{n\text{-}\pi^*}$ = 42 nm, PSS_{530} = 89% Z, PSS_{410} = 11% E) than for the related *ortho*-fluoro-azobenzene ligands **S1** ($\Delta_{n\text{-}\pi^*}$ = 35 nm, PSS_{530} = 80%, PSS_{410} = 15%)[8] and **S2** ($\Delta_{n\text{-}\pi^*}$ = 34 nm, PSS_{530} = 80% Z, PSS_{405} = 80% E)[9] (Figure S17), which is reflected in the improved PSS selectivity of Z-isomer generated by 530 nm irradiation.

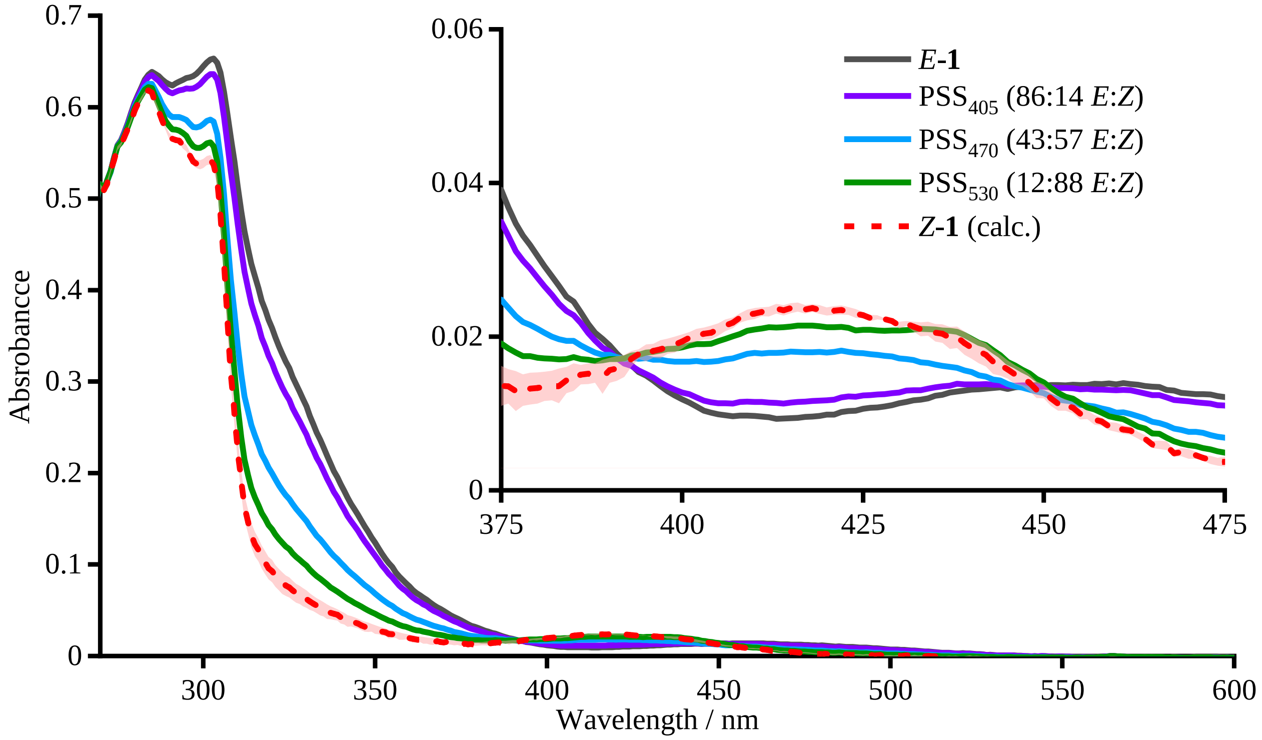

Figure S16. The UV-visible absorption (DMSO, 298 K) spectra of photoswitch **1** at PSS generated using LEDs with emission centred at various wavelengths. The spectrum for *Z*-**1** was calculated using the ^{1}H and ^{19}F NMR data together with absorption spectra solutions at PSS when irradiated with 405, 470, 530 nm.

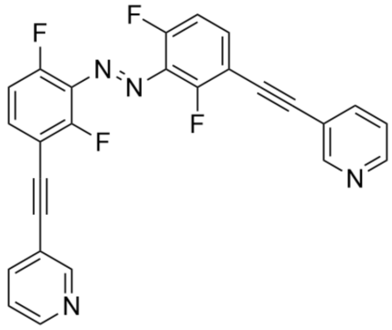

1

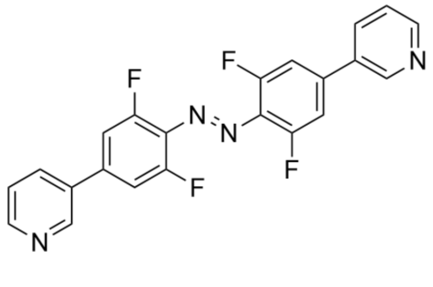

S1

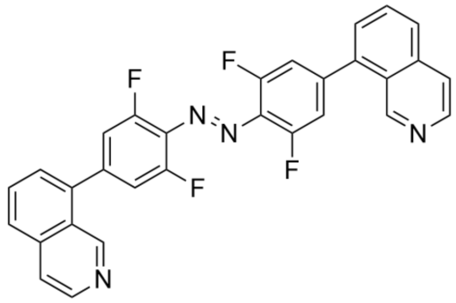

S2

Figure S17. Photoswitchable ligands **1**, **S1**, and **S2**

Table S5. Spectroscopic data[a] for photoswitchable ligand **1** and related photoswitchable ligands.

Ligand	*E*-isomer $\lambda_{n\text{-}\pi^*}$ / nm	*Z*-isomer $\lambda_{n\text{-}\pi^*}$ / nm	$\Delta\lambda_{n\text{-}\pi^*}$ / nm
**1**	459	418	41
**S1**[8]	462	427	35
**S2**[9]	459	425	34

[a]DMSO, 298 K

S3.3 Determining the thermal stability of ligand *Z*-1 in DMSO using UV-vis spectroscopy

We used UV-vis absorption spectroscopy to determine the thermodynamic parameters for the thermal isomerization of *Z*-**1** to *E*-**1**. The thermal recovery of *E*-**1** was monitored at 353, 363, and 373 K. For each temperature, a sample of ligand **1** (10 μM, DMSO) was irradiated with a 530 nm LED to enrich the sample with *Z*-**1** and the absorbance at 330 nm was monitored over 4 hours. The absorbance for each experiment was plot against time (Figure S18) and the data were fit to a mono-exponential to determine the first-order rate constant (Table S6).

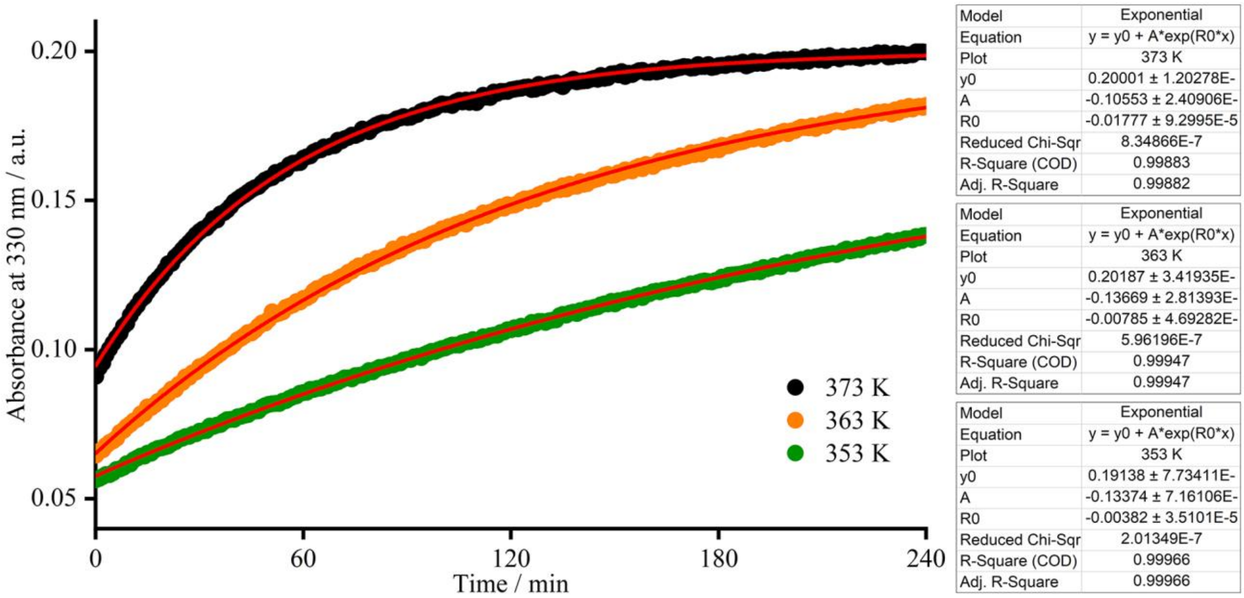

Figure S18. Thermal isomerization of *Z*-**1** to *E*-**1** in DMSO at 353, 363, and 373 K. Monitored by UV-vis spectroscopy.

Table S6. First-order rate constants for the thermal isomerization of *Z*-**1** to *E*-**1** at different temperatures.

Temperature / K	373	363	353
k / $min^{-1} \times 10^{-3}$	17.8 ± 0.01	7.85 ± 0.01	3.82 ± 0.01
k / $s^{-1} \times 10^{-4}$	2.96 ± 0.02	1.31 ± 0.01	0.637 ± 0.006
ln(k/T)	−14.1 ± 0.1	−14.8 ± 0.1	−15.5 ± 0.1
1/T / $K^{-1} \times 10^{-3}$	2.68 ± 0.004	2.75 ± 0.004	2.83 ± 0.004

First-order rate constants were determined in DMSO by fitting absorbance data to a mono-exponential.

The determined rate constants in Table S6, ln(k/T) was plotted against 1/T to make an Eyring plot (Figure S19). Data were fit to a straight line and the linear form of the Eyring equation was used to determine the entropy of activation ($\Delta S^{\ddagger} = -93.4 \pm 13.0$ $J\cdot mol^{-1}\cdot K^{-1}$), and the enthalpy of activation ($\Delta H^{\ddagger} = 82.5 \pm 4.7$ $kJ\cdot mol^{-1}$). Using these parameters, the change in Gibbs free energy of activation, $\Delta G^{\ddagger}$, for thermal isomerization was calculated for each temperature (Table S7). The values determined using the Eyring plot were extrapolated to determine $\Delta G^{\ddagger}$ and the half-life at room temperature (298 K) for the thermal isomerization from *E*-**1** to *Z*-**1** (Table S8). Extrapolating from the Eyring plot assumes that $\Delta H^{\ddagger}$ and $\Delta S^{\ddagger}$ are the same at all temperatures. That is, that the mechanism does not change with temperature, which is likely accurate for these intramolecular isomerisation reactions.

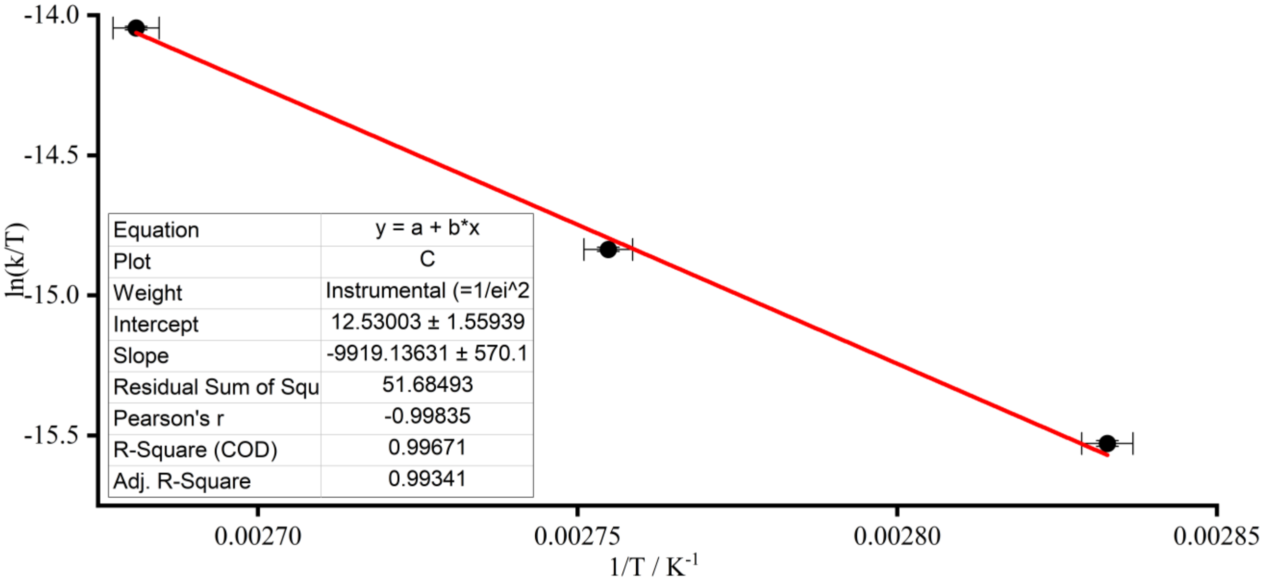

Figure S19. Eyring plot for the thermal isomerization of *Z*-**1** to *E*-**1** in DMSO. Rate constants (Table S6) were determined using UV-vis spectroscopy at 353, 363, 373 K (Figure S18).

Table S7. ΔG‡ for the thermal isomerization of *Z*-**1** to *E*-**1** in DMSO at different temperatures.

Temperature / K	373	363	353
ΔG‡ / kJ·mol^{-1}	117.3 ± 9.5	116.3 ± 9.4	115.3 ± 9.3

Table S8. Thermodynamic parameters and half-life for the thermal isomerisation of *Z*-**1** to *E*-**1** in DMSO at 298 K.

ΔG‡ / kJ·mol^{-1}	ΔH‡ / kJ·mol^{-1}	TΔS‡ / kJ·mol^{-1}	$t_{1/2}$ / days
110.2 ± 8.6	82.5 ± 4.7	-27.8 ± 3.9	27

Thermodynamic parameters determined using Eyring plot (Figure S19) and extrapolating to 298 K.

S4. Synthesis of palladium(II) complexes

S4.1 Synthesis of $[Pd(Py^*)_4](OTf)_2$

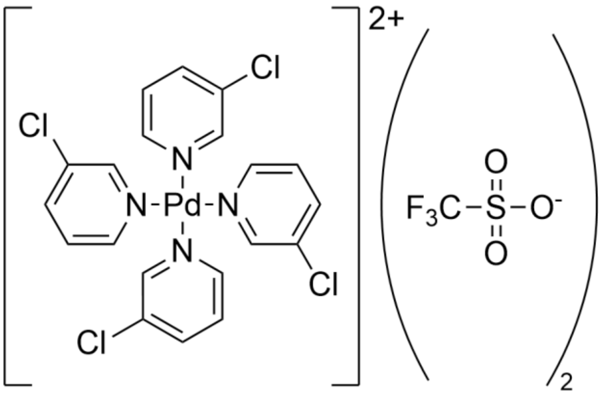

$[Pd(Py^*)_4](OTf)_2$ (Py* = 3-chloropyridine) was synthesized following a modified literature procedure. [10]

$Pd(OAc)_2$ (336 mg, 1.50 mmol, 1.0 equiv.) was dissolved in anhydrous acetonitrile (28 mL). The solution was degassed by bubbling argon through for 20 min. Triflic acid (0.39 mL, 4.4 mmol, 2.9 equiv.) was added dropwise over 5 min at room temperature and the solution changed from brown to yellow. The reaction mixture was stirred for 30 minutes at room temperature under an argon atmosphere. The solution was diluted with anhydrous diethyl ether (100 mL) yielding immediate precipitation of a pale-yellow solid. The mixture was stirred for 5 minutes before the solid was allowed to settle. The liquid was then decanted under a flow of argon. Anhydrous diethyl ether (50 mL) was added again, and the mixture was stirred for 5 minutes. After the precipitate has settled, the supernatant was decanted under a flow of argon. This procedure was repeated in total three times. The residual pale-yellow solid was dried under a flow of argon for 30 minutes and then dried under reduced pressure for 30 minutes. The resulting $Pd(OTf)_2$ was used in the next step without purification or characterisation due to its hygroscopic nature. Nitromethane (4 mL) was added to the residue to give a yellow solution. 3-Chloropyridine (Py*, 1.50 mL, 16.0 mmol, 10.7 equiv.) was added and the mixture was stirred at 80 °C for one hour. After cooling to room temperature, the solvent was removed *in vacuo*. The residue was triturated with chloroform (30 mL) to remove residual 3-chloropyridine and the solid was collected by filtration. The resulting pale-yellow crystalline solid was washed with chloroform (10 mL) and dried *in vacuo* yielding $[Pd(Py^*)_4](OTf)_2$ (866 mg, 1.01 mmol, 67%). The NMR spectroscopic data agreed with previously reported values.[10]

^1H NMR (400 MHz, CD_3NO_2) δ 9.14 (dt, *J* = 2.2, 0.6 Hz, 4H), 9.04 (ddd, *J* = 5.7, 1.3, 0.6 Hz, 4H), 8.04 (ddd, *J* = 8.4, 2.2, 1.2 Hz, 4H), 7.59 (ddd, *J* = 8.4, 5.7, 0.6 Hz, 4H).

^{19}F NMR (376 MHz, CD_3NO_2) δ -79.5.

S4.2 Synthesis of [Pd(Py*)$_4$](BAr$_F$)$_2$

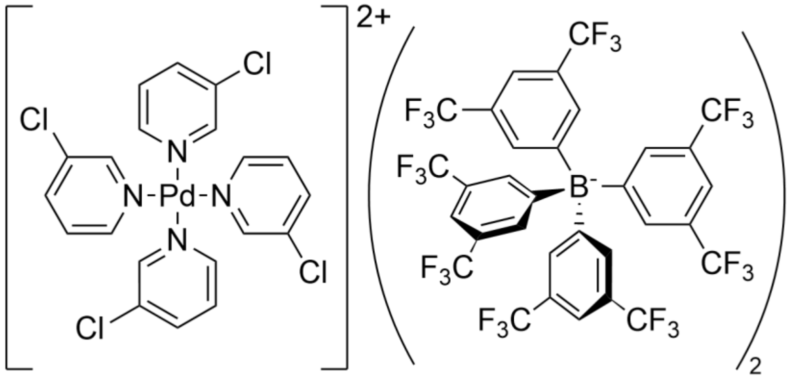

Title complex was prepared following literature procedure.[11] Sodium tetrakis[3,5-bis(trifluoromethyl)phenyl]borate) (NaBAr$_F$) was synthesized following literature procedure.[12]

[Pd(Py*)$_4$](OTf)$_2$ (150 mg, 175 µmol, 1.0 equiv.) and NaBAr$_F$ (310 mg, 350 µmol, 2.0 equiv.) were combined in a Schlenk flask and anhydrous CH_2Cl_2 (30 mL) was added. The reaction mixture was sonicated for 15 min at rt, then filtered to remove insoluble NaOTf. The filter was washed with anhydrous CH_2Cl_2 (2 × 30 mL) and the filtrate was evaporated under reduced pressure to obtain [Pd(Py*)$_4$](BAr$_F$)$_2$ as an off-white foam (328 mg, 143 µmol, 82%). Spectral data was identical to that previously reported.[11]

^{1}H NMR (400 MHz, CD_3NO_2) δ 8.89 (d, *J* = 2.2 Hz, 4H), 8.83 (dt, *J* = 5.7, 0.8 Hz, 4H), 8.07 (ddd, *J* = 8.4, 2.2, 1.2 Hz, 4H), 7.84 (br m, 16H), 7.67 (br s, 8H), 7.59 (dd, *J* = 8.4, 5.7 Hz, 4H).

^{19}F NMR (376 MHz, CD_3NO_2) δ -63.6.

S5. Preliminary guest binding studies between $[Pd_2L_4](BArF)_4$ in DCM

S5.1 Preparing $[Pd_2(L)_4](BAr_F)_4$ in DCM-d_2

Ligand **L** was prepared following a previously reported procedure.[8]

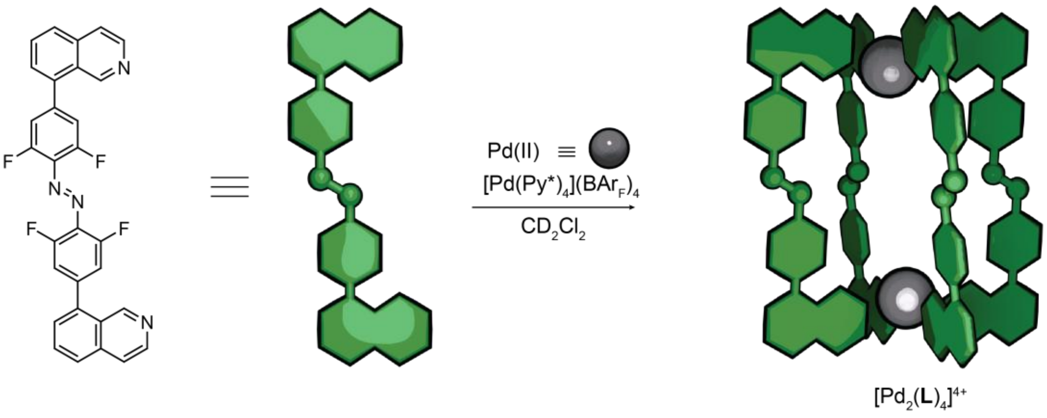

Scheme S2. Synthesis of homoleptic cage $[Pd_2(\mathbf{L})_4](BAr_F)_4$ in CD_2Cl_2.

Self-assembled species $[Pd_2(\mathbf{L})_4](BAr_F)_4$ was prepared by reacting a solution of ligand **L** (4.0 mL, 2.5 mM, 9.8 μmol, 1 equiv.) in DCM-d_2 (Figure S20i) with a solution of $[Pd(Py^*)_4](BAr_F)_4$ (75 μL, 67 mM, 4.9 μmol, 0.5 equiv.) in DCM-d_2. The sample was kept at 30°C for 20 min to equilibrate. This afforded a solution of $[Pd_2(\mathbf{L})_4](BAr_F)_4$ ([Pd] = 1.2 mM) and free Py* (6.0 mM) in DCM-d_2 (Figure S20iii).

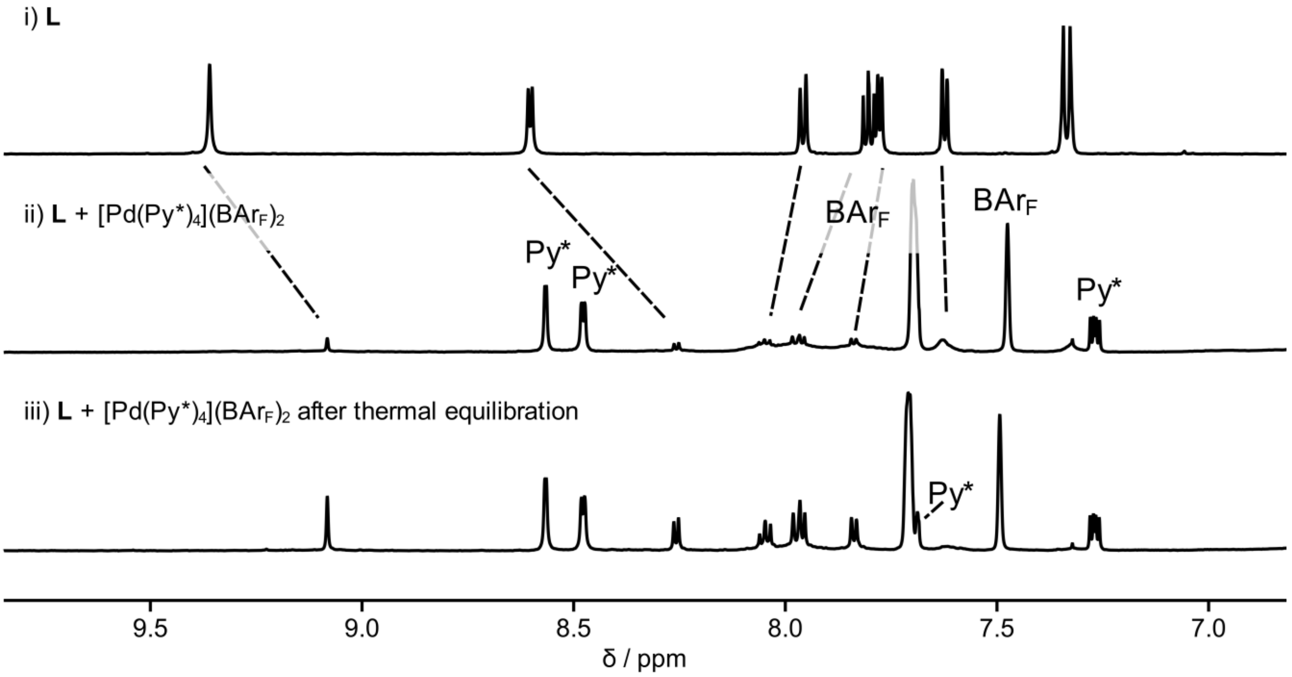

Figure S20. ^{1}H NMR (600 MHz, 298 K, DCM-d_2) spectra of i) ligand **L**, ii) **L** (2.5 mM, 1 equiv.) after immediately after adding $[Pd(Py^*)_4](BAr_F)_4$, and iii) the sample of **L** (2.5 mM, 1 equiv.) and $[Pd(Py^*)_4](BAr_F)_4$ after heating at 30°C for 20 min.

S5.2 Screening guest binding between $[Pd_2(L)_4](BAr_F)_4$ and different Michael addition acceptors and donors

^{1}H NMR spectroscopy was used to monitor the change in chemical shift of $[Pd_2(\mathbf{L})_4](BAr_F)_4$ environments after adding different Michael addition donors and acceptors. Solution in DCM-d_2 of methyl vinyl ketone (25 µL, 0.71 M), nitromethane (25 µL, 0.82 M), malononitrile (25 µL, 0.76 M), methyl acrylate (30 µL, 0.58 M), and benzoyl nitromethane (25 µL, 0.61 M) were individually added to different samples of $[Pd_2(\mathbf{L})_4](BAr_F)_4$ ([Pd] = 1.2 mM).

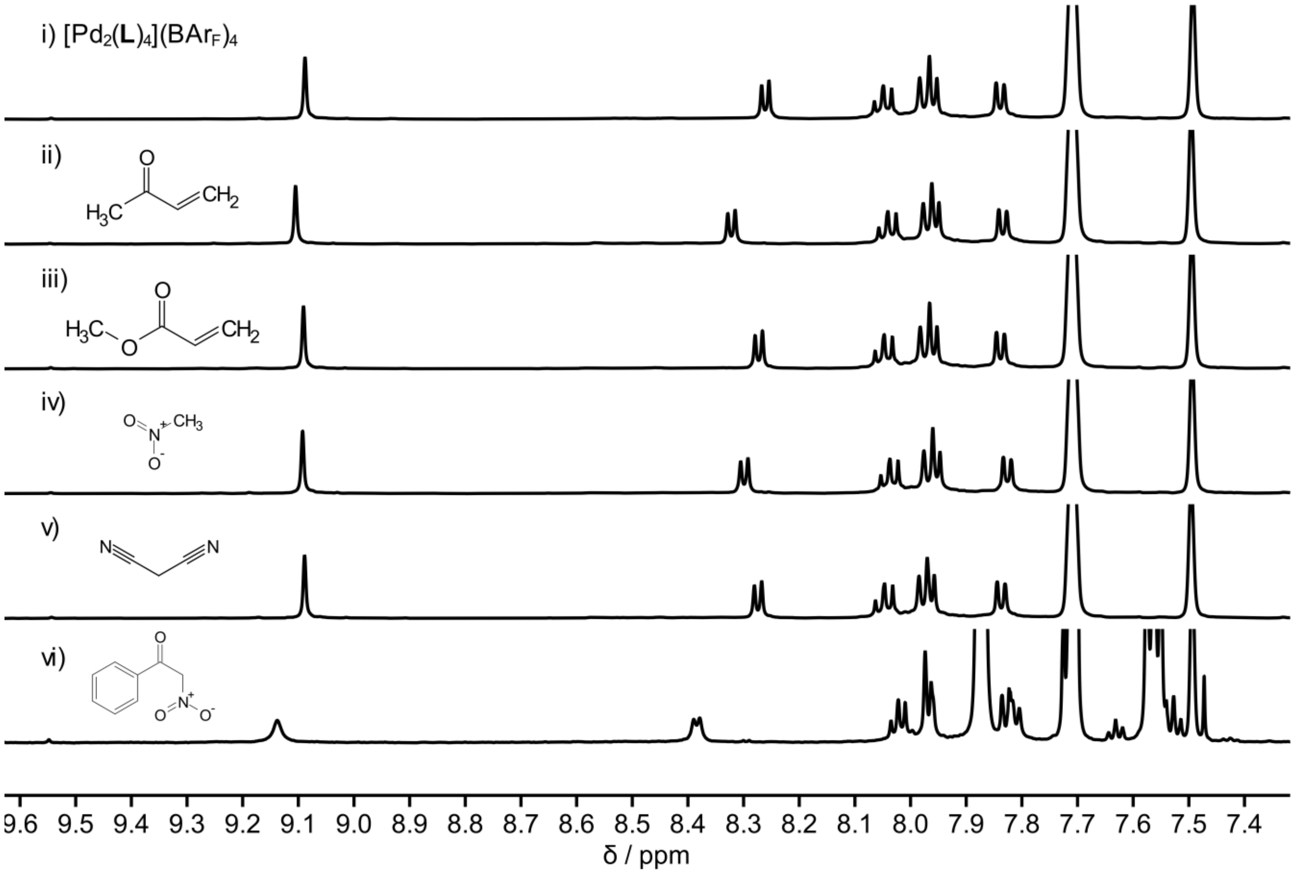

Figure S21. ^{1}H NMR (500 MHz, 298 K, DCM-d_2) spectra of i) $[Pd_2(\mathbf{L})_4](BAr_F)_4$ ([Pd] = 1.2 mM), and $[Pd_2(\mathbf{L})_4](BAr_F)_4$ ([Pd] = 1.2 mM) with ii) methyl vinyl ketone (34 mM), iii) nitromethane (39 mM), iv) malononitrile (36 mM), v) methyl acrylate (33 mM), and vi) benzoyl nitromethane (29 mM).

S5.3 Catalysing the Michael addition between vinyl methyl ketone and benzoyl nitromethane using $[Pd_2(L)_4](BAr_F)_4$

^1H NMR spectroscopy was used to determine whether $[Pd_2(\mathbf{L})_4](BAr_F)_4$ could catalysis the Michael addition reaction between benzoyl nitromethane and methyl vinyl ketone. These substrates were selected as their addition to $[Pd_2(\mathbf{L})_4](BAr_F)_4$ resulted in the largest change in chemical shift compared to the other Michael donors and acceptors.

A solution of $[Pd_2(\mathbf{L})_4](BAr_F)_4$ ([Pd] = 1.2 mM, 500 µL) in CD_2Cl_2 was prepared. Solutions of benzoyl nitromethane (25 µL, 0.61 M), methyl vinyl ketone (25 µL, 0.71 M), and 18-crown-6 (10 µL, 0.41 M) in CD_2Cl_2 were added. This afforded a sample of $[Pd_2(\mathbf{L})_4](BAr_F)_4$ ([Pd] = 1.1 mM, 1 equiv.), benzoyl nitromethane (29 mM, 25 equiv.), methyl vinyl ketone (34 mM, 29 equiv.), and 18-crown-6 (2.2 mM, 2.0 equiv.) in CD_2Cl_2. The sample was monitored at room temperature for 10 hours. The signal intensities of the signal at 5.97 ppm were used to monitor the abundance of benzoyl nitromethane and the integral of the signal at 2.66 ppm was used to monitor the abundance of the product Michael addition product (Figure S22). After 10 hours, no product formation could be observed.

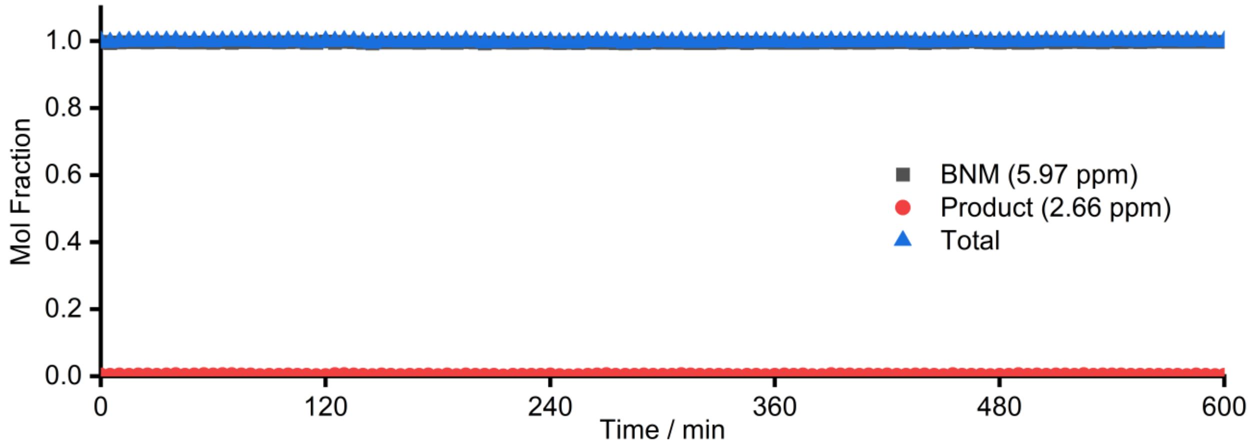

Figure S22. Monitoring catalysis within homoleptic cage $[Pd_2(\mathbf{L})_4](BAr_F)_4$. ^1H NMR (600 MHz, 298 K, DCM-d_2) signal intensities of benzoyl nitromethane and Michael addition product following the reaction between benzoyl nitromethane (29 mM, 25 equiv.) and methyl vinyl ketone (34 mM, 29 equiv.) in the presence of 18-crown-6 (2.2 mM, 2.0 equiv.) and $[Pd_2(\mathbf{L})_4](BAr_F)_4$ ([Pd] = 1.1 mM, 1 equiv.). The integral of the signal at 5.97 ppm was used to monitor the abundance of benzoyl nitromethane and the integral of the signal at 2.66 ppm was used to monitor the abundance of the product Michael addition product. Relaxation delay (Bruker setting D1) was set to 5 seconds.

S6. Synthesis and characterization of homoleptic cage $[Pd_2(E\text{-}1)_4]^{4+}$

S6.1 Synthesis of $[Pd_2(E\text{-}1)_4](BF_4)_4$ in DMSO-d_6

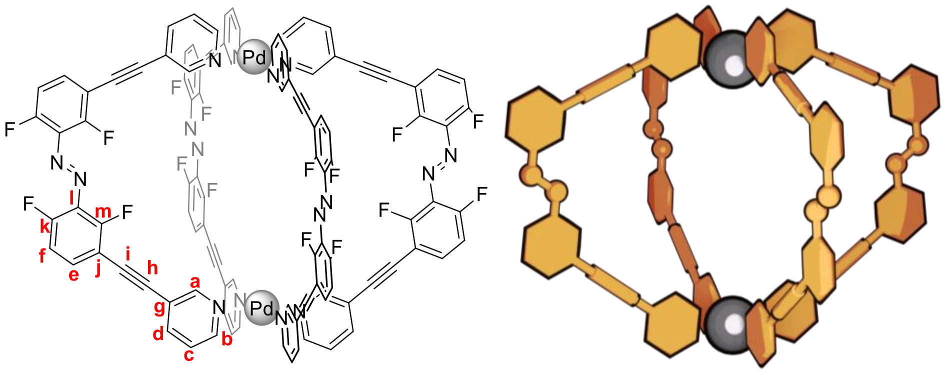

Self-assembly between palladium(II) and ligand **1** was characterized using NMR spectroscopy and ESI-MS. To assemble the homoleptic cage $[Pd_2(E\text{-}\mathbf{1})_4](BF_4)_4$, a solution of photoswitchable ligand **1** (1 equiv., 500 µL, 4.8 mM, DMSO-d_6) was thermally equilibrated by heating with heat gun set to 150 °C for 3 minutes until only *E*-**1** was present (monitored by ^1H NMR spectroscopy). A solution of $[Pd(MeCN)_4](BF_4)_2$ (0.5 equiv., 23 µL, 52 mM, DMSO-d_6) was added to the solution of *E*-**1** and the sample was equilibrated at room temperature for 10 min. The ^1H and ^{19}F NMR spectra revealed that a single self-assembled species was generated near-quantitatively. Using ESI-MS, we determined the self-assembled product to be $[Pd_2(E\text{-}\mathbf{1})_4](BF_4)_4$. Formation of a $[Pd_2L_4]^{4+}$ structure is consistent with self-assembly between a similar previously reported ligand and palladium(II) ions.[9]

^1H NMR (600 MHz, 298 K, DMSO-d_6) δ 9.44 (d, J = 2.0 Hz, 8H, H^a), 9.24 (dd, J = 5.9, 1.4 Hz, 8H, H^b), 8.31 (dt, J = 8.1, 1.6 Hz, 8H, H^d), 7.95 (td, J = 8.3, 5.7 Hz, 8H, H^e), 7.82 (dd, J = 7.9, 5.9 Hz, 8H, H^c), 7.45 (ddd, J = 10.1, 8.6, 1.4 Hz, 8H, H^f) ppm.

^{19}F NMR (565 MHz, 298 K, DMSO-d_6) δ −118.38 (br, F^m), −118.55 (br, F^k) ppm.

^{13}C NMR (151 MHz, 298 K, DMSO-d_6) δ 155.5(C^k), 153.7(C^m), 152.5 (C^a), 151.1 (C^b), 143.1 (C^d), 136.8 (C^e), 130.9 (C^l), 127.3 (C^c), 121.5 (C^g), 114.0 (C^f), 107.4 (C^j), 89.2 (C^h), 86.0 (C^i) ppm.

Table S9. Comparison of NMR environments between ligand *E*-**1** and $[Pd_2(E\text{-}\mathbf{1})_4](BF_4)_4$.

	$^{1}H^{a}$		$^{13}C^{b}$		$^{19}F^{c}$	
Environment	*E*-**1**	$[Pd_2(E\text{-}\mathbf{1})_4](BF_4)_2$	*E*-**1**	$[Pd_2(E\text{-}\mathbf{1})_4](BF_4)_2$	*E*-**1**	$[Pd_2(E\text{-}\mathbf{1})_4](BF_4)_2$
**a**	8.81	9.44	151.6	152.5	-	-
**b**	8.64	9.24	149.6	151.1	-	-
**c**	7.51	7.82	123.7	129.3	-	-
**d**	8.04	8.31	138.7	143.1	-	-
**e**	7.95	7.95	136.3	136.8	-	-
**f**	7.51	7.45	113.9	114.0	-	-
**g**	-	-	118.5	121.5	-	-
**h**	-	-	91.9	89.2	-	-
**i**	-	-	83.7	86.0	-	-
**j**	-	-	108.4	107.4	-	-
**k**	-	-	154.9	154.5	−117.92	−118.55
**l**	-	-	130.7	130.9	-	-
**m**	-	-	154.6	154.5	−117.67	−118.38

[a](600 MHz, 298 K, DMSO-d_6), [b](151 MHz, 298 K, DMSO-d_6), [c](565 MHz, 298 K, DMSO-d_6).

S6.2 1D and 2D NMR spectra of [Pd_2(*E*-1)$_4$](BF_4)$_4$ in DMSO-d_6

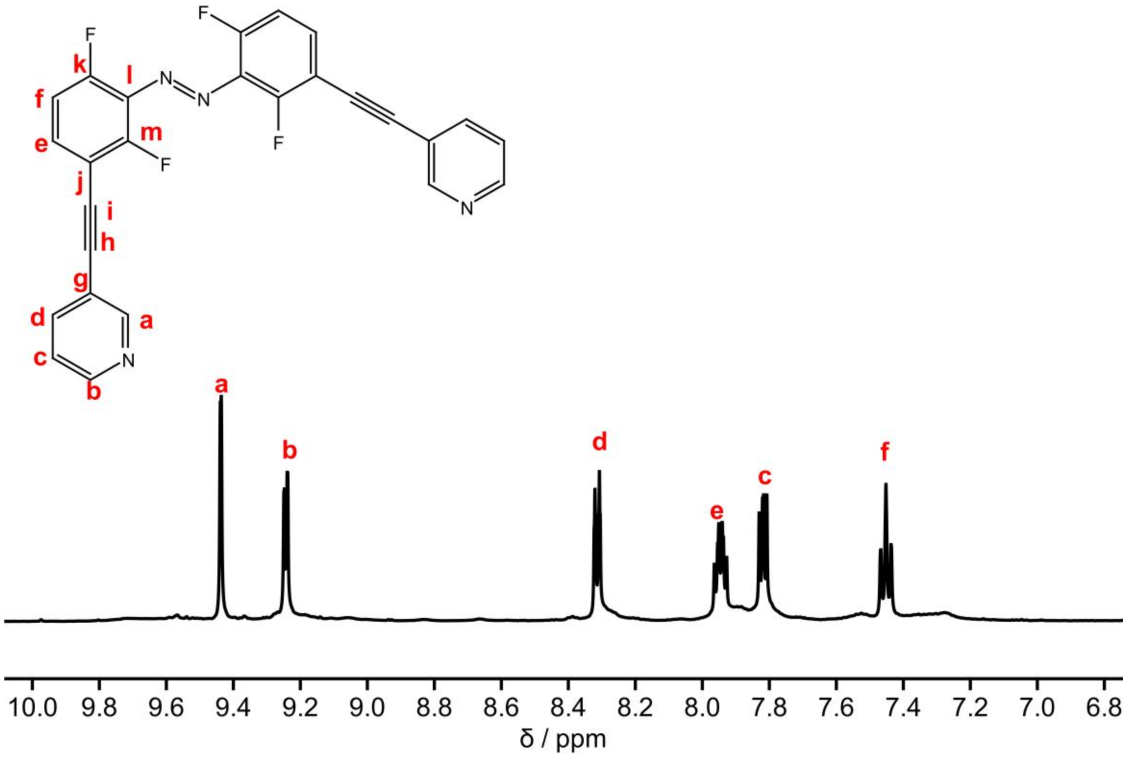

Figure S23. ^{1}H NMR (600 MHz, 298 K, DMSO-d_6) spectrum of [Pd_2(*E*-**1**)$_4$](BF_4)$_4$ ([Pd] = 2.3 mM).

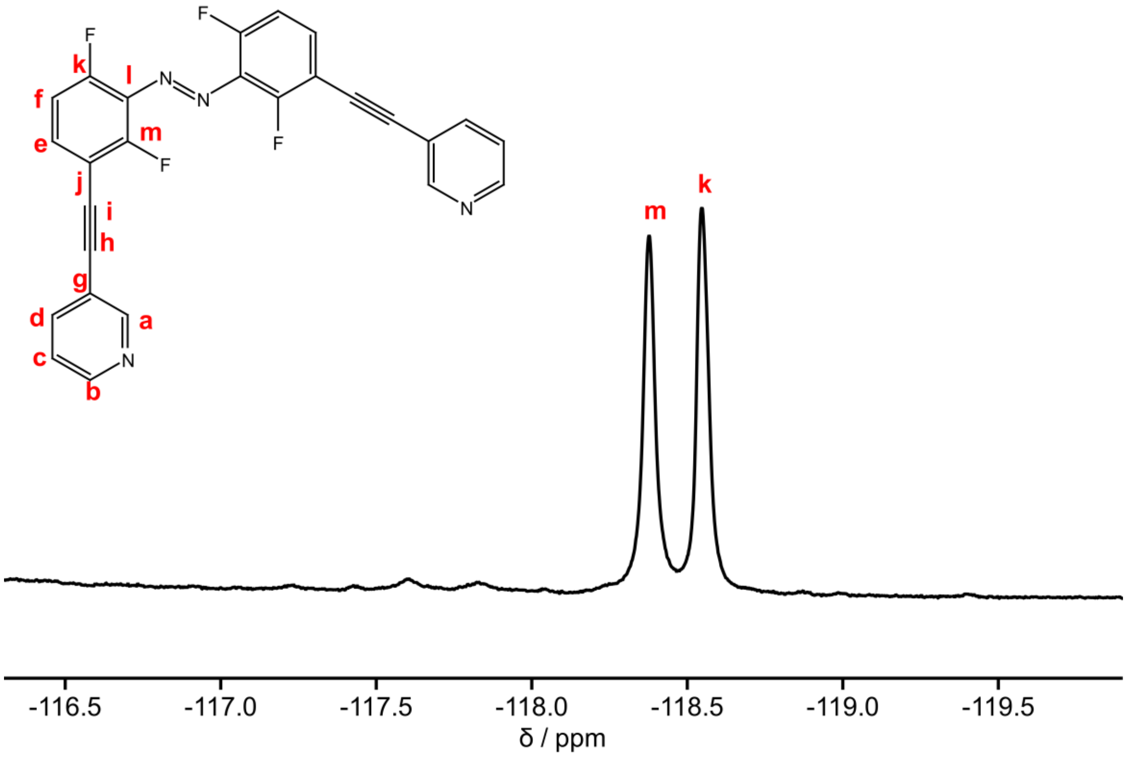

Figure S24. ^{19}F NMR (565 MHz, 298 K, DMSO-d_6) spectrum of [Pd_2(*E*-**1**)$_4$](BF_4)$_4$ ([Pd] = 2.3 mM).

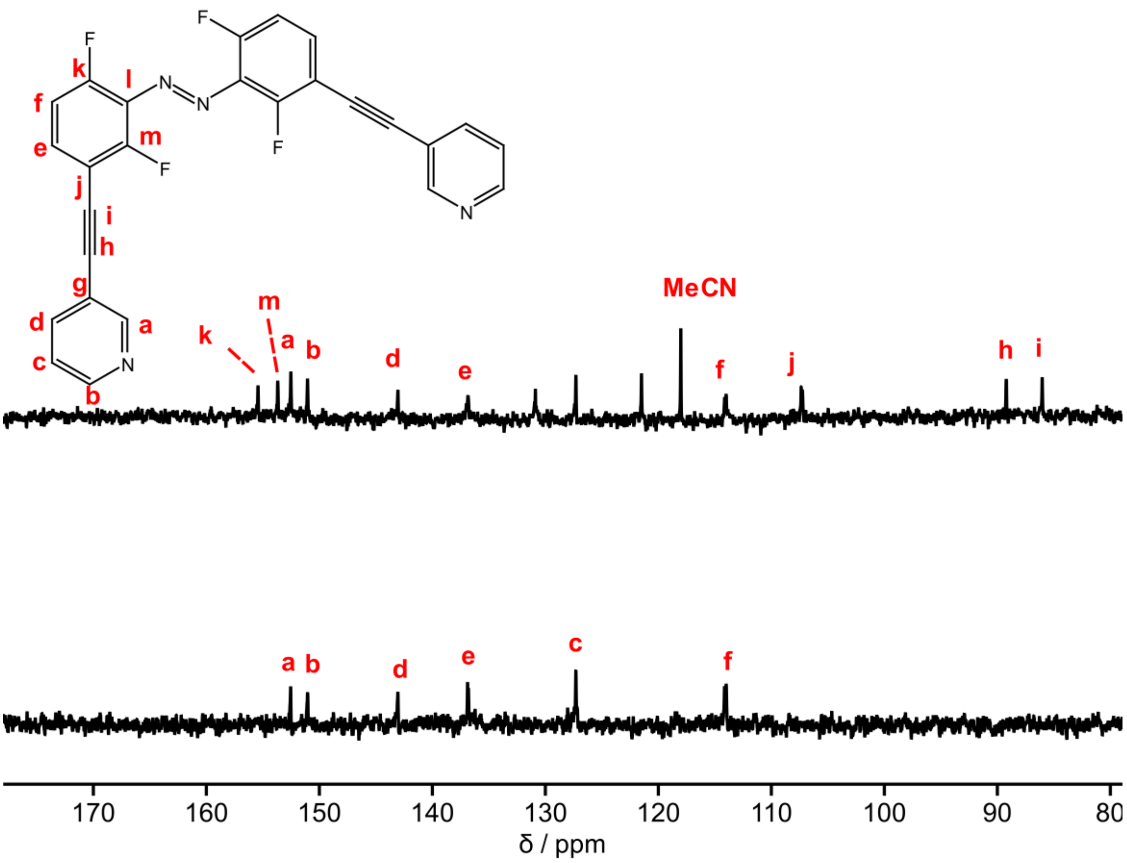

Figure S25. $^{13}C\{^1H\}$ (top) and DEPT-135 (bottom) NMR (151 MHz, 298 K, DMSO-d_6) spectrum of $[Pd_2(E\text{-}\mathbf{1})_4](BF_4)_4$ ([Pd] = 2.3 mM).

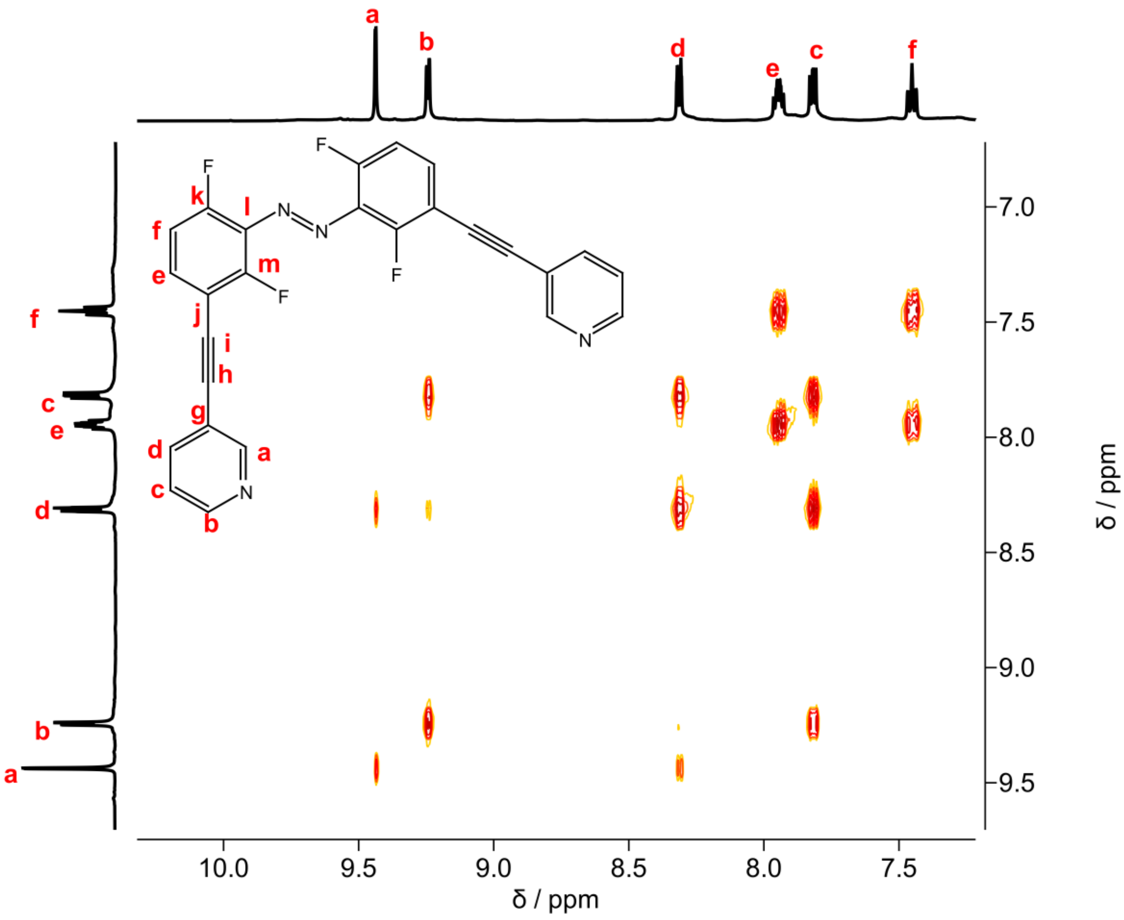

Figure S26. ^1H-^1H COSY NMR (600 MHz, 298 K, DMSO-d_6) spectrum of $[Pd_2(E\text{-}\mathbf{1})_4](BF_4)_4$ ([Pd] = 2.3 mM).

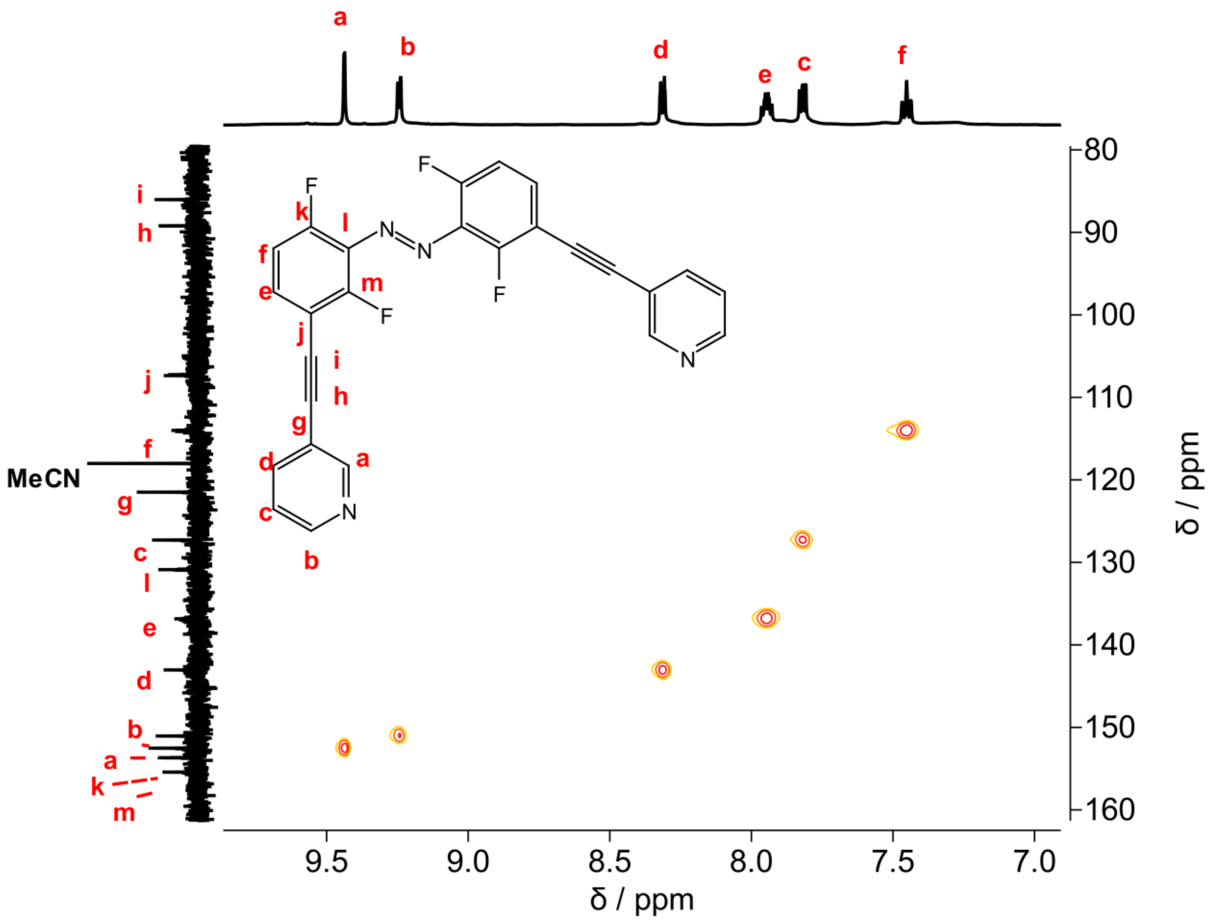

Figure S27. ^1H-^{13}C HSQC NMR (600 MHz, 151 MHz, 298 K, DMSO-d_6) spectrum of $[Pd_2(E\text{-}\mathbf{1})_4](BF_4)_4$ ([Pd] = 2.3 mM).

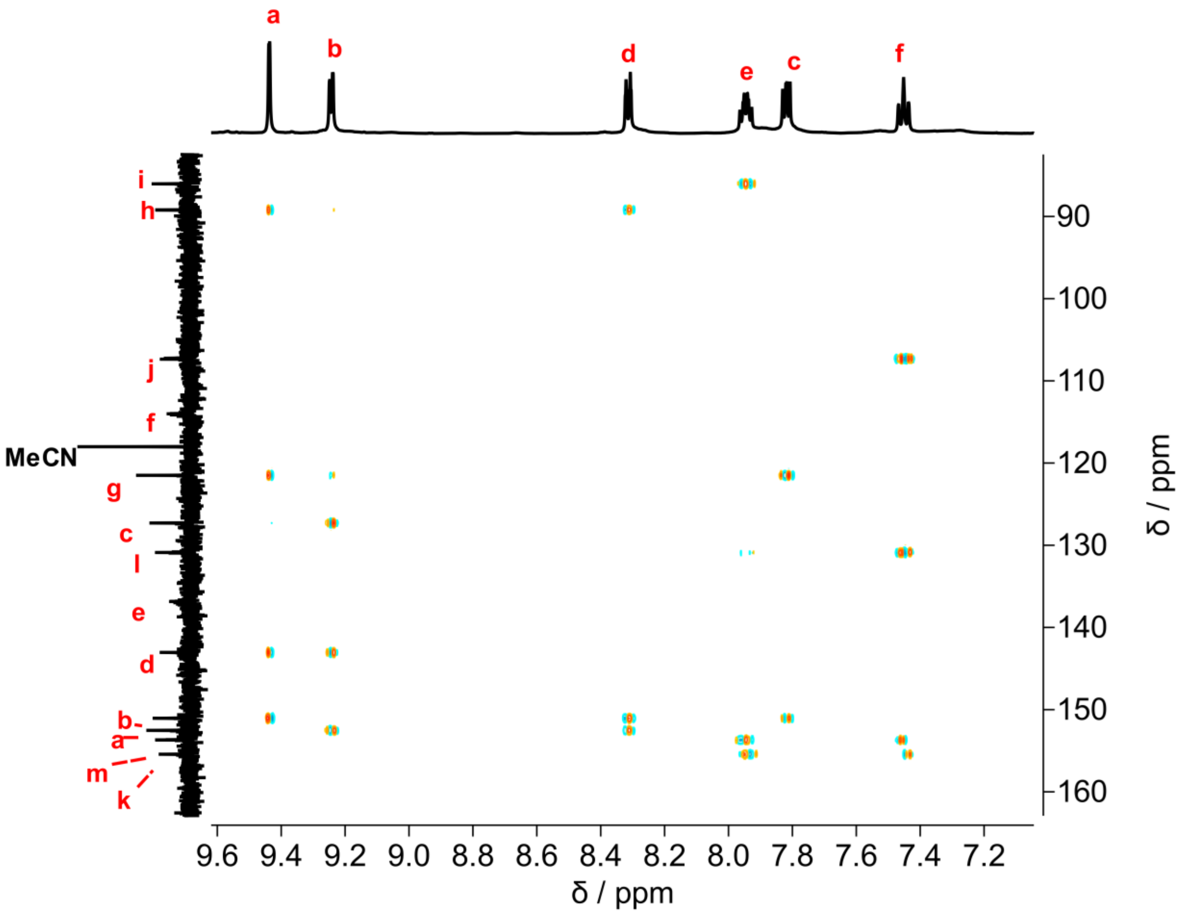

Figure S28. ^{1}H-^{13}C HMBC NMR (600 MHz, 151 MHz, 298 K, DMSO-d_6) spectrum of [Pd_2(*E*-**1**)$_4$](BF_4)$_4$ ([Pd] = 2.3 mM).

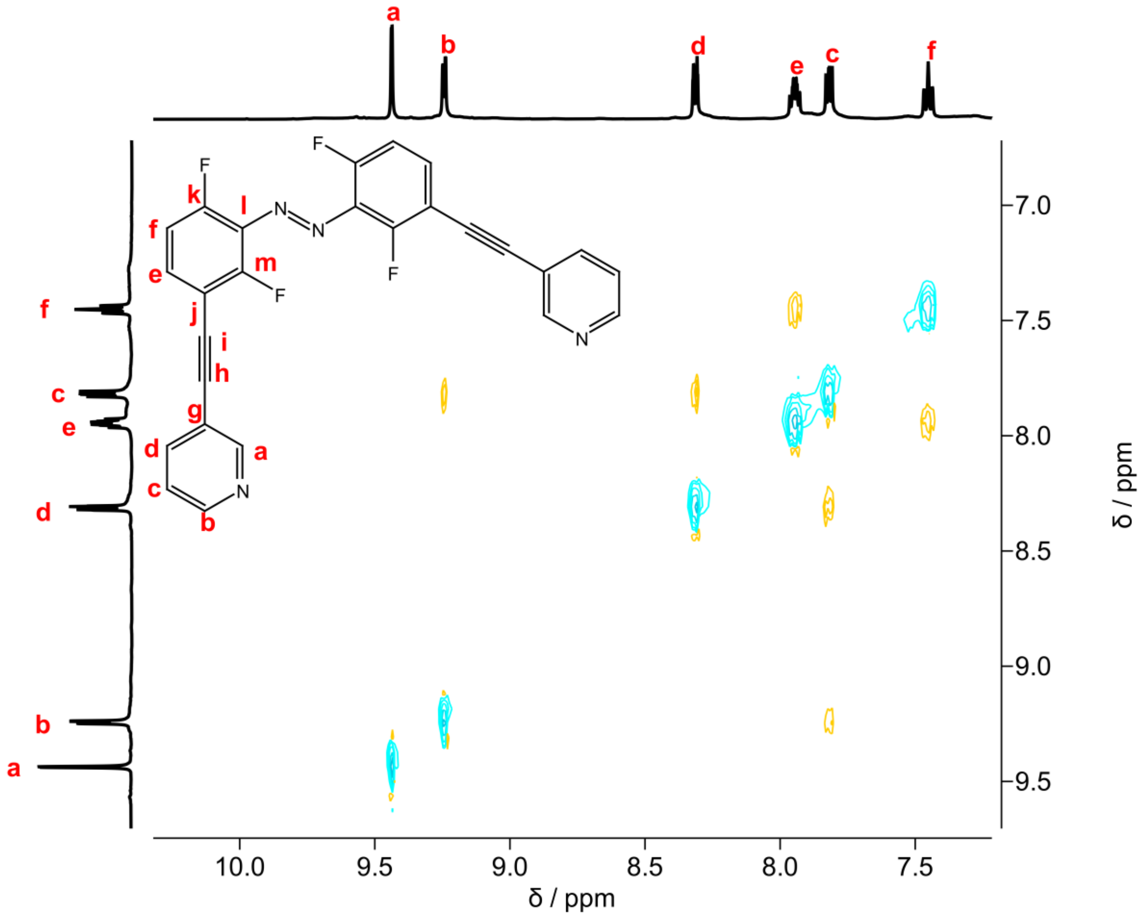

Figure S29. ^{1}H-^{1}H ROESY NMR (600 MHz, 298 K, DMSO-d_6) spectrum of $[Pd_2(E\text{-}\mathbf{1})_4](BF_4)_4$ ([Pd] = 2.3 mM).

S6.3 ESI-MS spectra of [Pd$_2$(*E*-**1**)$_4$](BF$_4$)$_4$ in DMSO

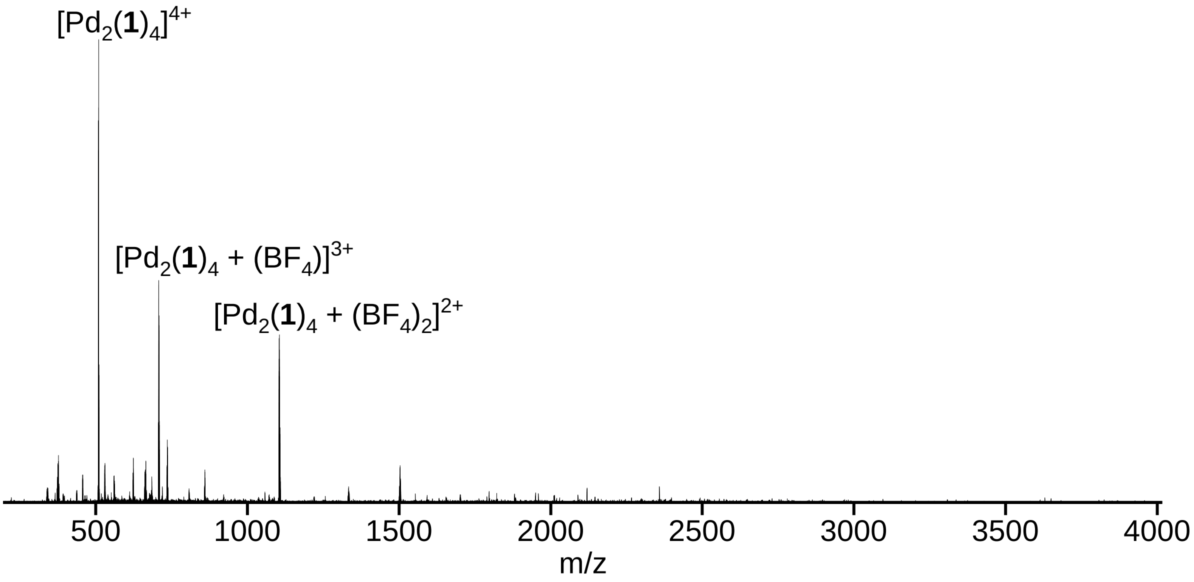

Figure S30. ESI-MS spectrum of [Pd$_2$(*E*-**1**)$_4$](BF$_4$)$_4$ ([Pd] = 2.3 mM) in DMSO.

Table S10. Zoom scans of select ESI-MS peaks, with simulated isotope patterns ESI-MS of [Pd$_2$(*E*-**1**)$_4$](BF$_4$)$_4$ ([Pd] = 2.3 mM) in DMSO-*d*$_6$.

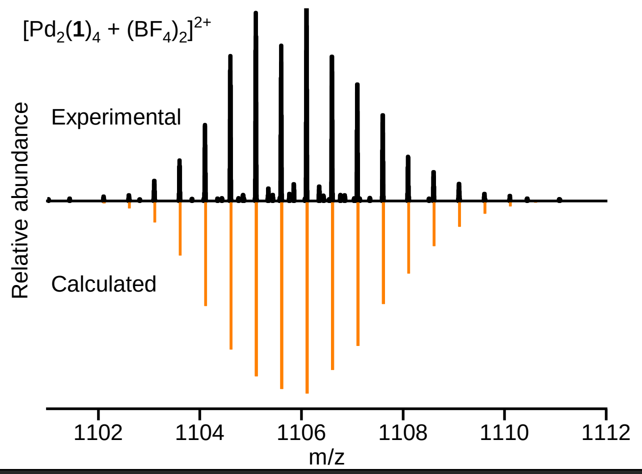	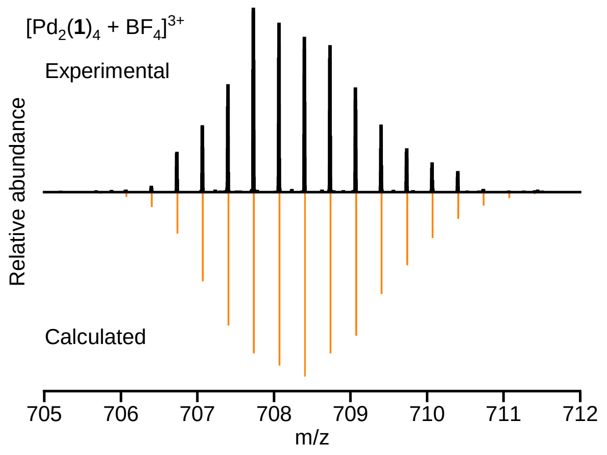
[Pd$_2$(**1**)$_4$ + (BF$_4$)$_2$]$^{2+}$	[Pd$_2$(**1**)$_4$ + BF$_4$]$^{3+}$
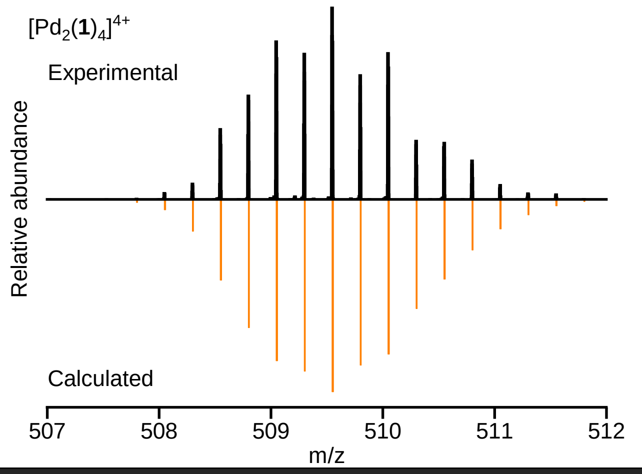	
[Pd$_2$(**1**)$_4$]$^{4+}$	

S6.4 Synthesis of $[Pd_2(E\text{-}\mathbf{1})_4](BAr_F)_4$ in MeCN-d_3

To prepare an isomerically pure sample of *E*-**1**, a sample containing a mixture of *E*-**1** and *Z*-**1** was dissolved in anhydrous acetonitrile. The solution was heated in a microwave reactor at 130 °C for 15 min, then the solvent was removed under reduced pressure. Ligand *E*-**1** (6.25 mg, 13.7 µmol, 2.0 equiv.) was dissolved in MeCN-d_3 (3.2 mL) and a solution of $[Pd(Py^*)_4](BAr_F)_2$ (280 µL, 23.9 mM, 1.0 equiv.) was added. The solution was prepared in the dark and was equilibrated at room temperature for 30 minutes to afford a stock solution of $[Pd_2(E\text{-}\mathbf{1})_4](BAr_F)_4$ ([Pd] = 1.96 mM) and free 3-chloropyridine (Py*).

An NMR sample tube was charged with the stock solution of $[Pd_2(E\text{-}\mathbf{1})_4](BAr_F)_4$ (500 µL, [Pd] = 1.96 mM) and free Py*. The sample was heated at 50 °C for 20 min to equilibrate the sample. After the addition of $[Pd(Py^*)_4](BAr_F)_2$, one ligand **1** environment is observed in the ^{1}H NMR spectrum (Figure S31), indicating that the self-assembled product has a high order of symmetry. The composition of the sample was determined using ESI-MS spectroscopy as $[Pd_2(E\text{-}1)_4 + (BAr_F)_{4-n}]^{n+}$ (n = 2-4). The self-assembled product $[Pd_2(E\text{-}\mathbf{1})_4]^{4+}$, is the same as when ligand **1** is reacted with $[Pd(MeCN)_4](BF_4)_2$ in DMSO-d_6, indicating that the solvent and anion does not change the outcome of self-assemble, as is observed in the case of ligand **2**.[13]

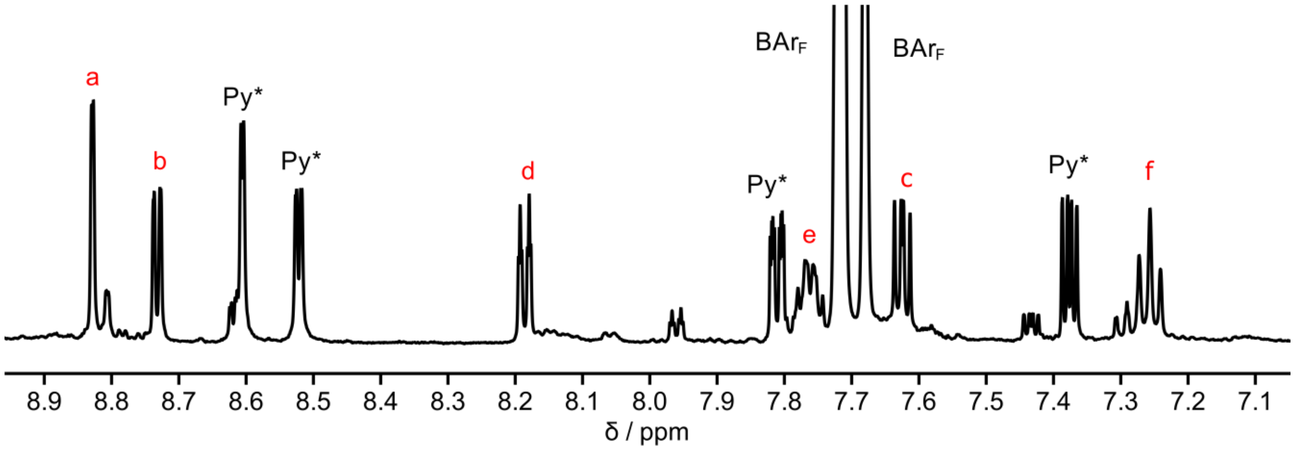

Figure S31. ^{1}H NMR (600 MHz, 298 K, MeCN-d_3) spectra of $[Pd_2(E\text{-}\mathbf{1})_4](BAr_F)_4$ ([Pd] = 1.96 mM).

S6.5 ESI-MS spectra of $[Pd_2(E\text{-}\mathbf{1})_4](BAr_F)_4$ in MeCN

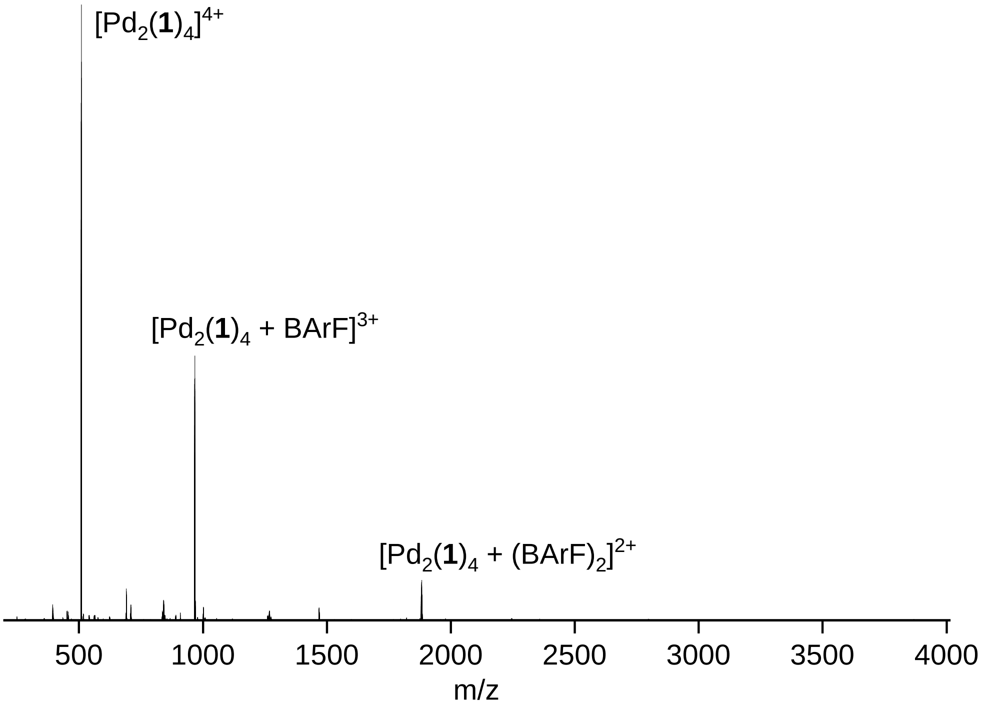

Figure S32. ESI-MS spectrum of $[Pd_2(E\text{-}\mathbf{1})_4](BAr_F)_4$ ([Pd] = 2.1 mM) in MeCN.

Table S11. Zoom scans of select ESI-MS peaks, with simulated isotope patterns ESI-MS of $[Pd_2(E\text{-}\mathbf{1})_4](BAr_F)_4$ ([Pd] = 2.1 mM) in MeCN.

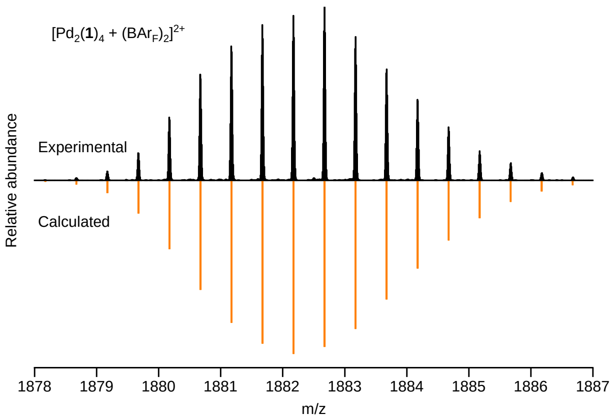	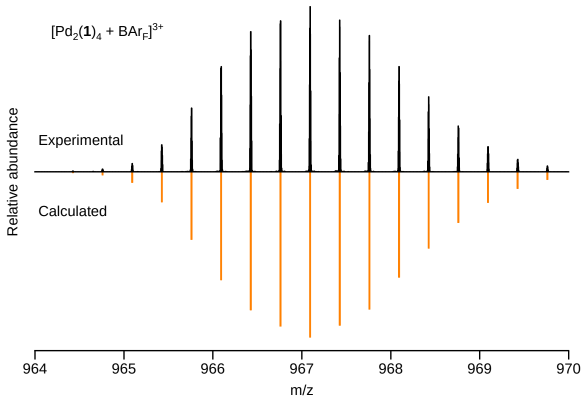
$[Pd_2(\mathbf{1})_4 + (BAr_F)_2]^{2+}$	$[Pd_2(\mathbf{1})_4 + BAr_F]^{3+}$
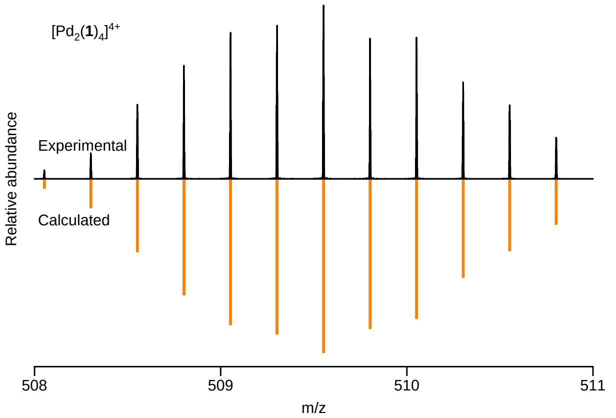	
$[Pd_2(\mathbf{1})_4]^{4+}$	

S7. Photoswitching of $[Pd_2(E\text{-}1)_4]^{4+}$

S7.1 Synthesis of $[Pd(Z\text{-}1)_2](BF_4)_2$ in DMSO-d_6

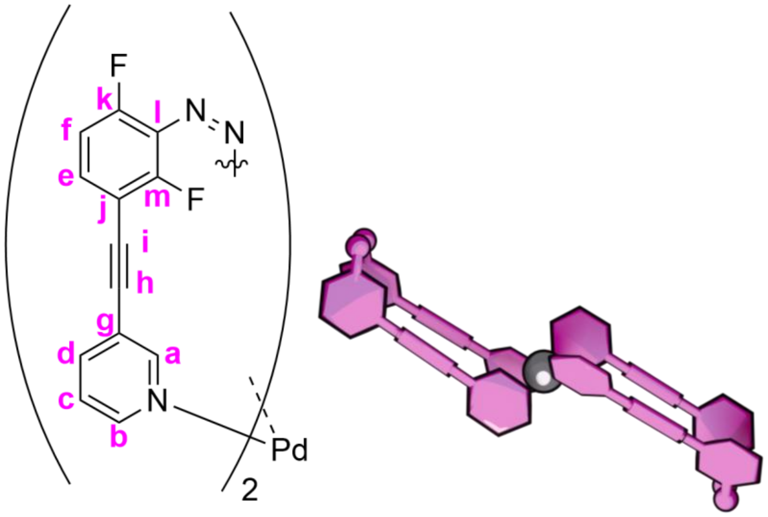

To prepare a sample of $[Pd(Z\text{-}\mathbf{1})_2](BF_4)_2$, the sample of $[Pd_2(E\text{-}\mathbf{1})_4](BF_4)_4$ in DMSO-d_6 from Supporting Information section S6.1 was irradiated with a 530 nm LED for 10 minutes. The sample was kept at room temperature for 10 min to equilibrate. ^1H NMR spectroscopy, showed the quantitative disassembly of $[Pd_2(E\text{-}\mathbf{1})_4](BF_4)_4$ and the concomitant formation of one major self-assembled product and multiple minor products. Using ESI-MS we assigned the major product to be $[Pd(Z\text{-}\mathbf{1})_2](BF_4)_2$. Formation of a $[PdL_2]^{2+}$ structure is consistent with self-assembly between a similar previously reported ligand and palladium(II) ions.[9]

^1H NMR (600 MHz, DMSO-d_6) δ 9.55 (s, 4H, H^a), 8.99 (s, 4H, H^b), 8.20 (dt, J = 8.06, 1.59 Hz, 4H, H^d), 7.72 – 7.66 (m, 8H, H^{c+e}), 7.37 (dt, J = 8.87, 4.35 Hz, 4H, H^f).

^{19}F NMR (565 MHz, DMSO-d_6) δ -115.5 (F^m), -118.4 (F^k).

^{13}C NMR (151 MHz, DMSO-d_6) δ 152.6 (C^a), 152.0 ($C^{k/m}$), 151.4 (C^b), 150.3 ($C^{k/m}$), 141.7 (C^d), 133.7 (C^e), 131.6 (C^l), 127.1 (C^c), 121.6 (C^g), 113.6 (C^f), 106.9 (C^j), 90.3 (C^h), 86.0 (C^i).

S7.2 1D and 2D NMR spectra of $[Pd(Z\text{-}\mathbf{1})_2](BF_4)_2$ in DMSO-d_6

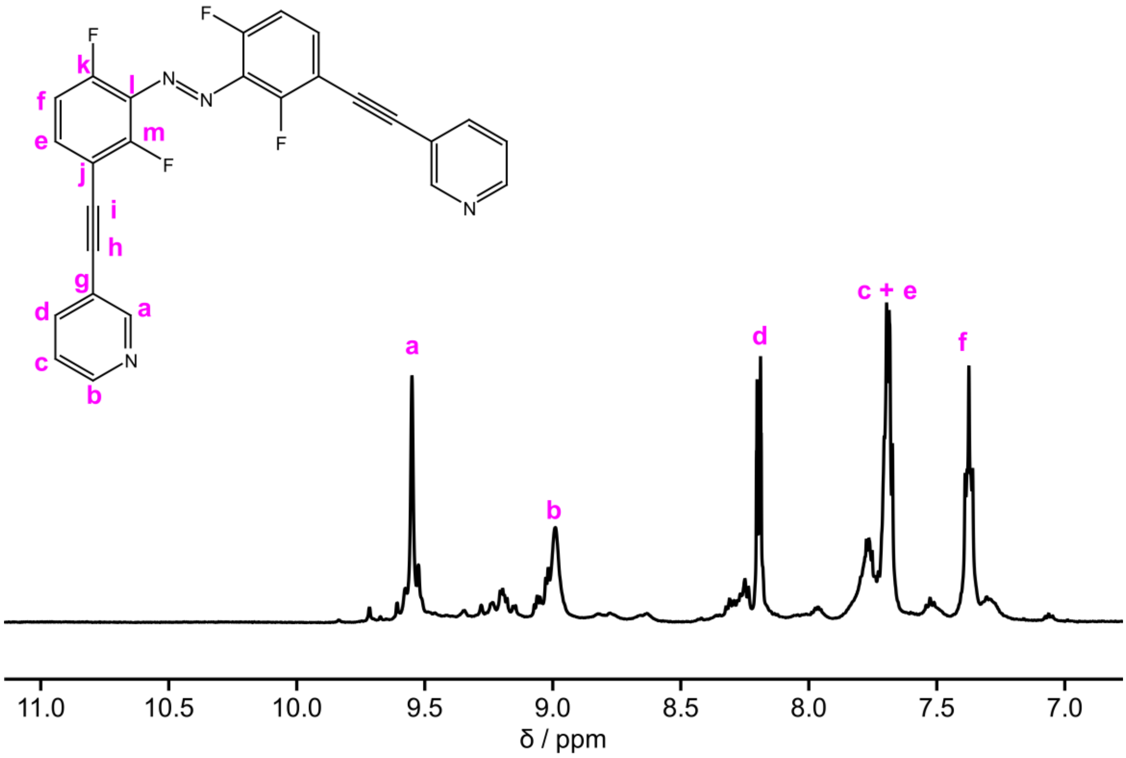

Figure S33. ^{1}H NMR (600 MHz, 298 K, DMSO-d_6) spectrum of $[Pd(Z\text{-}\mathbf{1})_2](BF_4)_2$ ([Pd] = 2.3 mM).

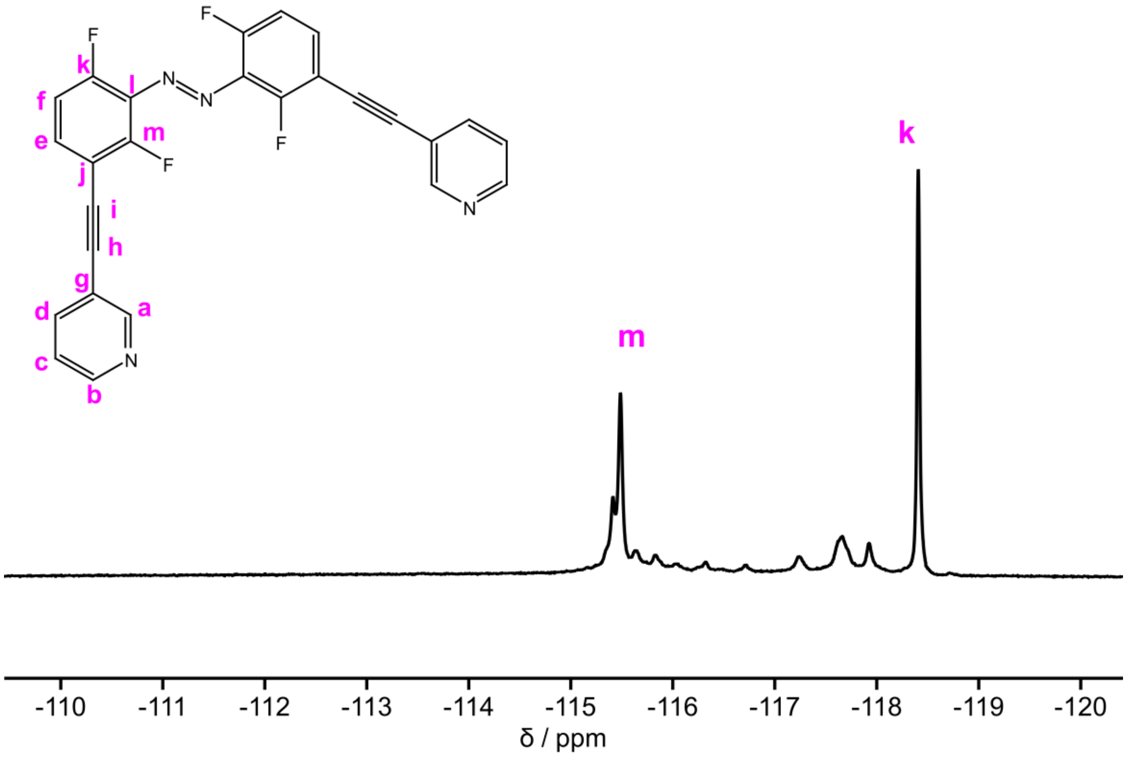

Figure S34. ^{19}F NMR (565 MHz, 298 K, DMSO-d_6) spectrum of $[Pd(Z\text{-}\mathbf{1})_2](BF_4)_2$ ([Pd] = 2.3 mM).

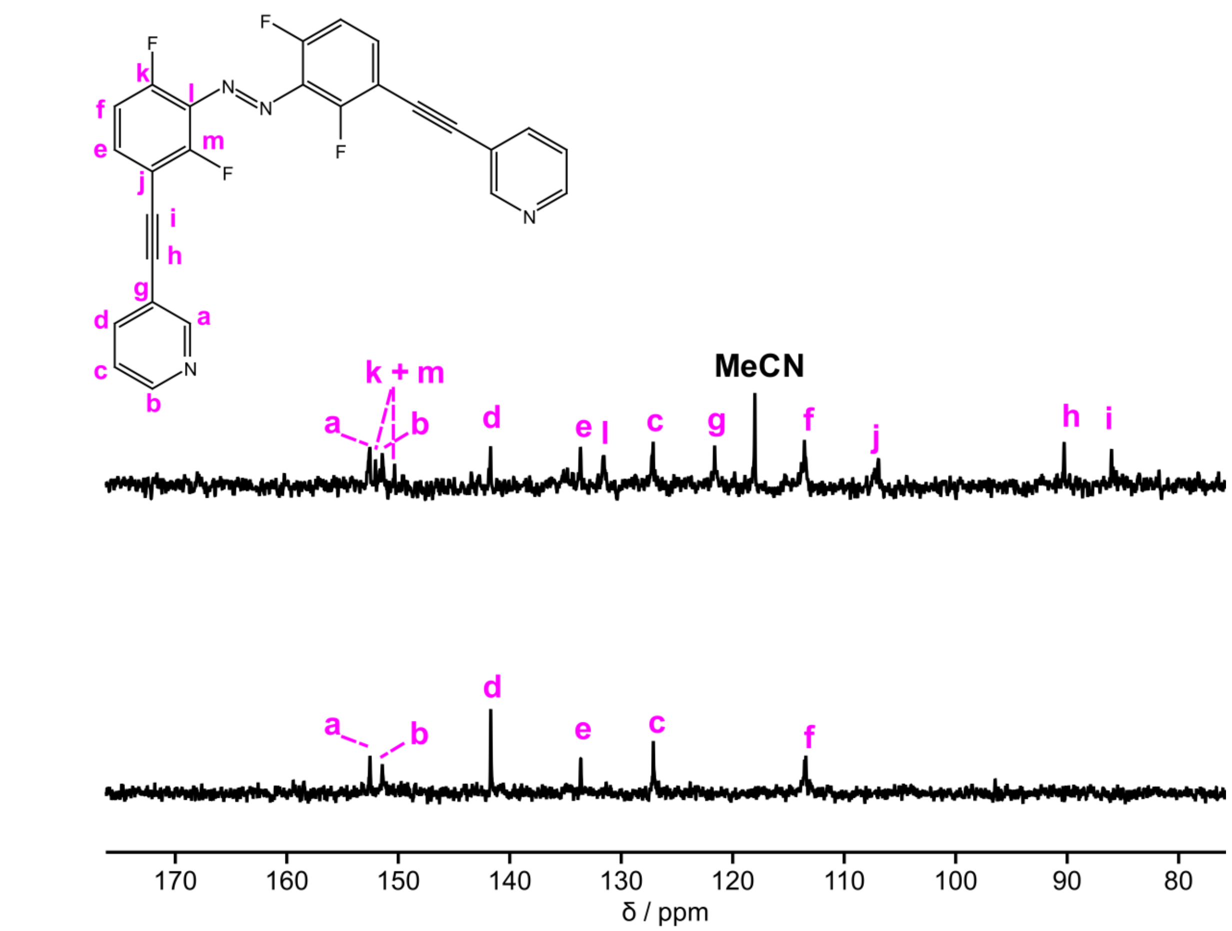

Figure S35. $^{13}C\{^1H\}$ (top) and DEPT-135 (bottom) NMR (151 MHz, 298 K, DMSO-d_6) spectrum of [Pd(*Z*-**1**)$_2$](BF$_4$)$_2$ ([Pd] = 2.3 mM).

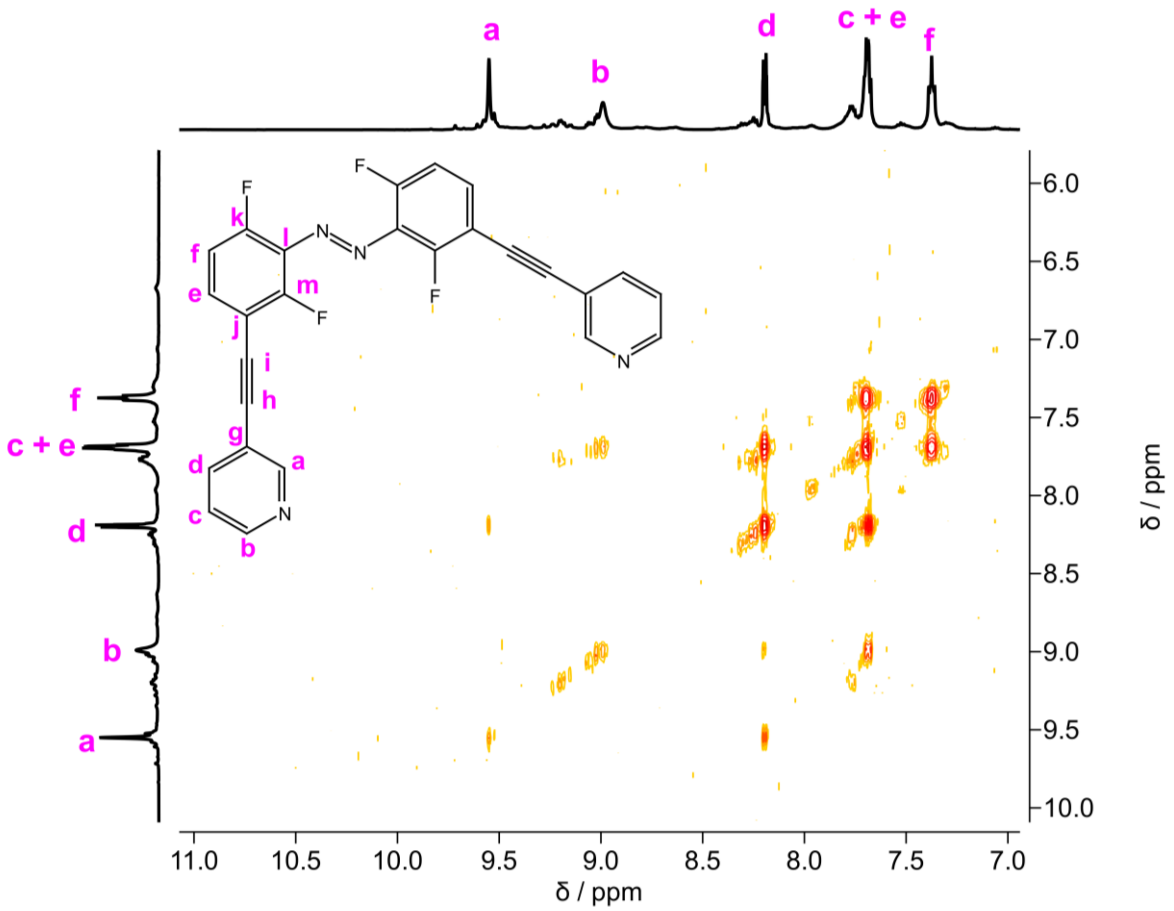

Figure S36. ^{1}H-^{1}H COSY NMR (600 MHz, 298 K, DMSO-d_6) spectrum of $[Pd(Z\text{-}\mathbf{1})_2](BF_4)_2$ ([Pd] = 2.3 mM).

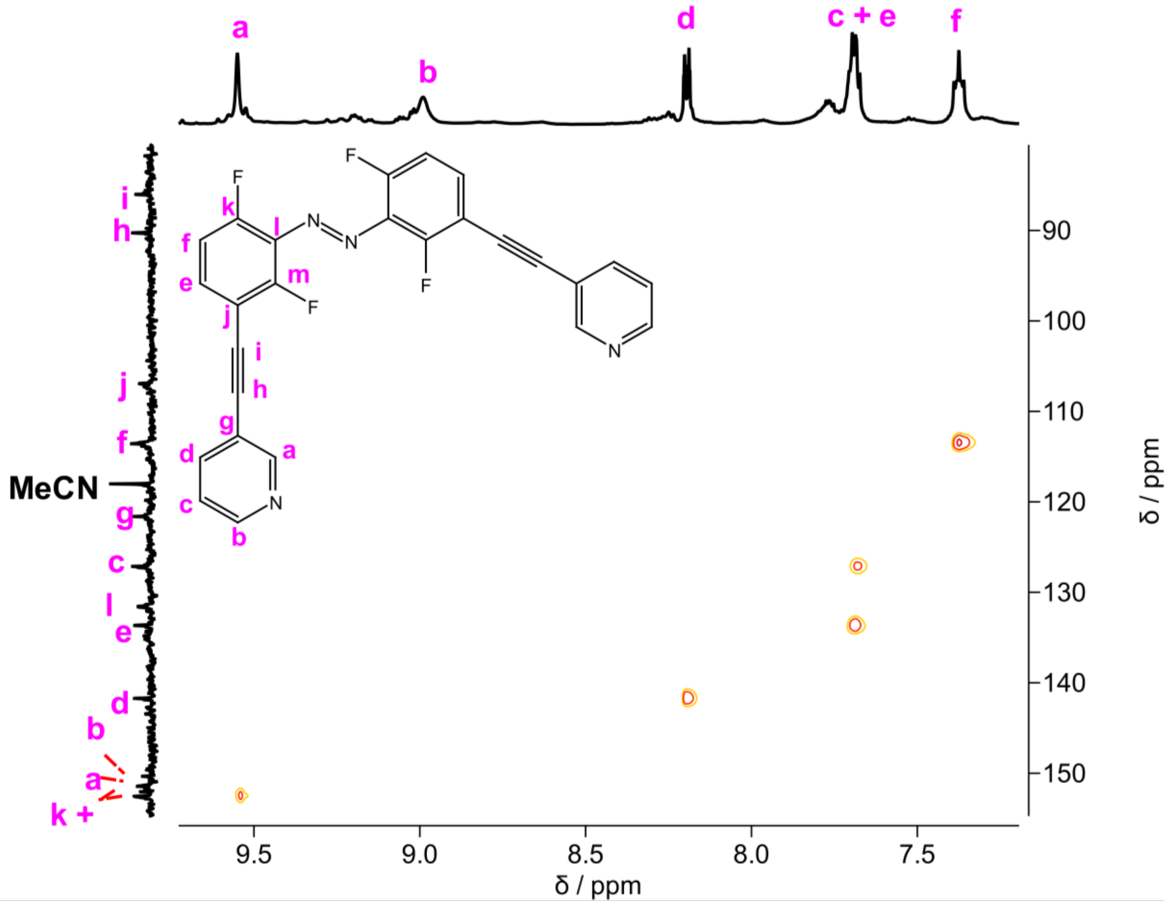

Figure S37. ^{1}H-^{13}C HSQC NMR (600 MHz, 151 MHz, 298 K, DMSO-d_6) spectrum of $[Pd(Z\text{-}\mathbf{1})_2](BF_4)_2$ ([Pd] = 2.3 mM).

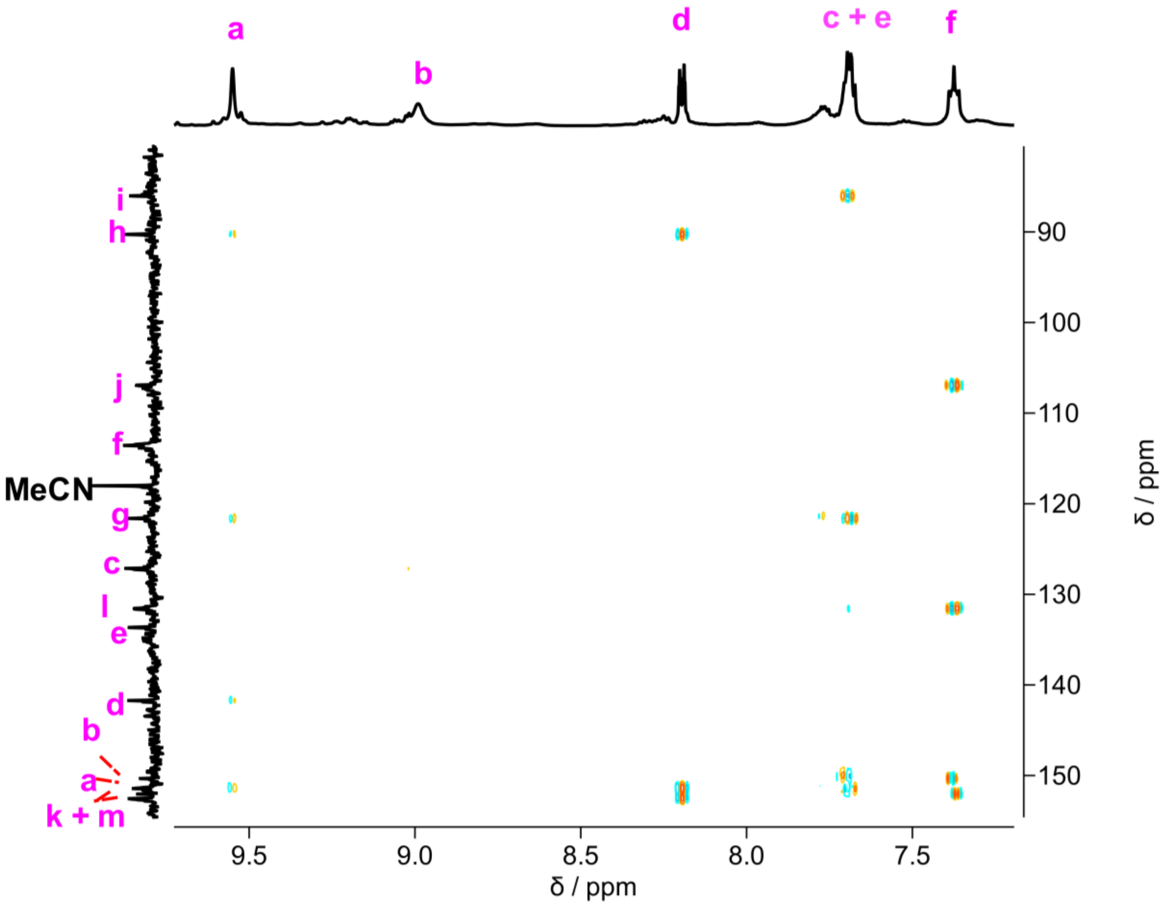

Figure S38. ^{1}H-^{13}C HMBC NMR (600 MHz, 151 MHz, 298 K, DMSO-d_6) spectrum of [Pd(Z-**1**)$_2$](BF$_4$)$_2$ ([Pd] = 2.3 mM).

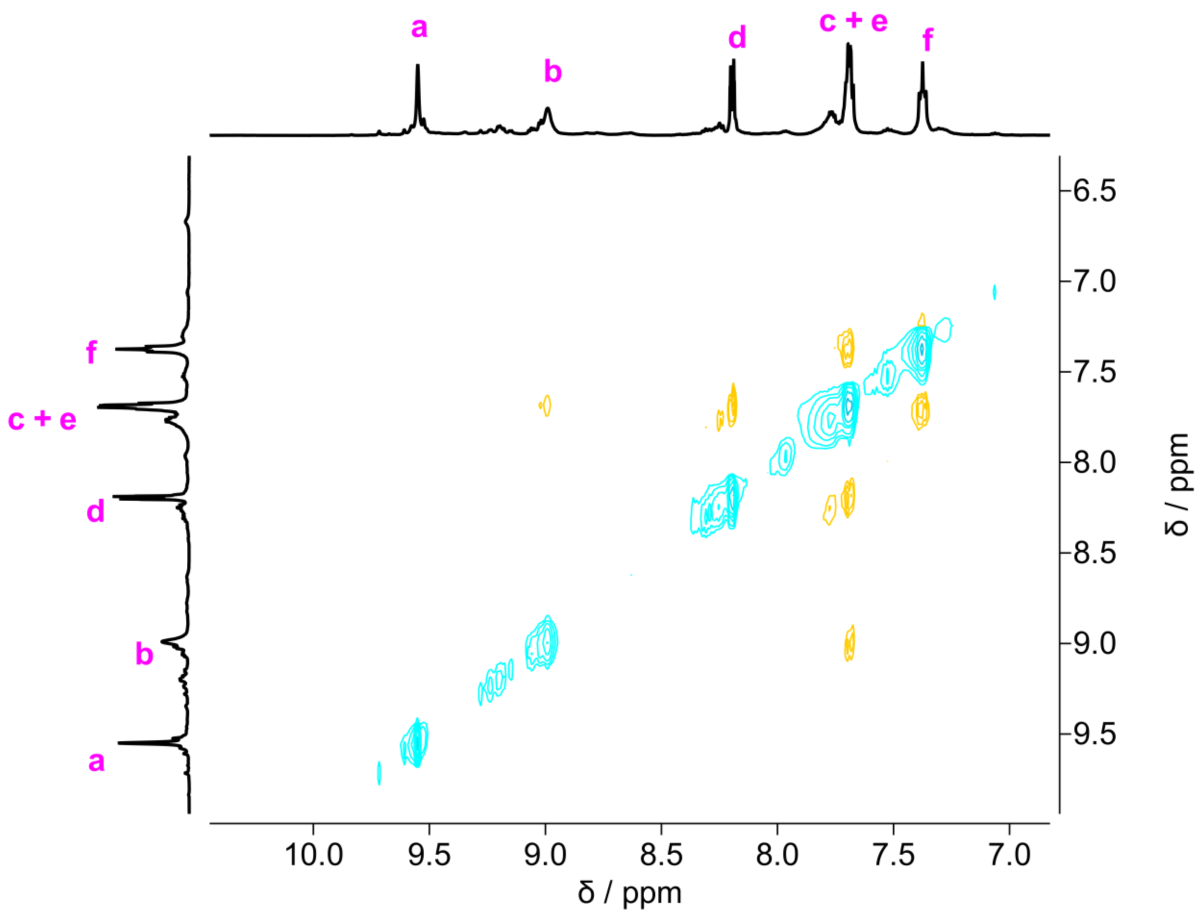

Figure S39. ^{1}H-^{13}C ROESY NMR (600 MHz, 151 MHz, 298 K, DMSO-d_6) spectrum of $[Pd(Z\text{-}\mathbf{1})_2](BF_4)_2$ ([Pd] = 2.3 mM).

S7.3 Measuring PSS distribution in DMSO

A sample of [Pd_2(*E*-**1**)$_4$](BF_4)$_4$ ([Pd] = 2.1 mM, 500 µL in DMSO-*d*$_6$, 1.06 µmol, 1.0 equiv.) was prepared in the dark and heated to 50 °C for 30 minutes to thermally equilibrate the sample.

^{1}H (Figure S40) and ^{19}F NMR (Figure S41) spectroscopy were used to monitor how irradiation affects [Pd_2(*E*-**1**)$_4$](BF_4)$_4$. The sample of [Pd_2(*E*-**1**)$_4$](BF_4)$_4$ (Figure S40i, Figure S41i) was irradiated with 530 nm light for 20 min (Figure S40ii, Figure S41ii) which resulted in the formation of [Pd(*Z*-**1**)$_2$](BF_4)$_2$. Then 4-dimethylaminopyridine (DMAP, 164 mM, 128 µL in DMSO-*d*$_6$, 21.0 µmol, 20.0 equiv.) was added to the sample (Figure S40iii, Figure S41iii). This afforded a solution containing free ligand **1**, free DMAP, and [Pd(DMAP)$_4$](BF_4)$_2$. The ^{1}H NMR spectrum does not have any cleanly resolved signals for the two isomers, so we used ^{19}F NMR to quantify the PSS.

We measured the ^{19}F NMR signal intensities of *E*-**1** and *Z*-**1** after DMAP was added to calculate the distribution of the *E*/*Z* isomers of **1**. Irradiating [Pd_2(*E*-**1**)$_4$](BF_4)$_4$ generated a PSS containing 96% *Z*-**1** and 4% *E*-**1**, which is a more favoured towards *Z*-**1** than for free ligand **1** when irradiated with 530nm light (S3.1).

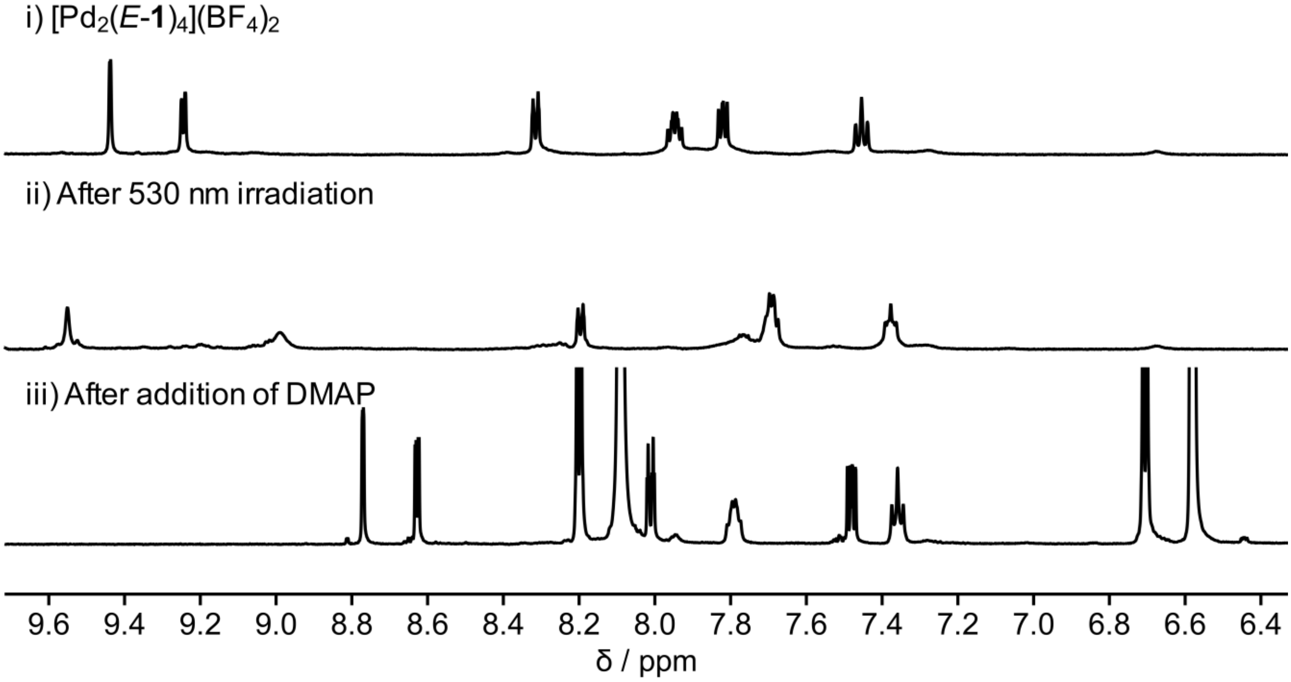

Figure S40. ^{1}H NMR (600 MHz, 298 K, DMSO-*d*$_6$) spectra of a sample of [Pd_2(*E*-**1**)$_4$](BF_4)$_4$ ([Pd] = 2.1 mM, 1 equiv.) i) before irradiation, ii) after irradiating with a 530 nm LED for 20 min, and iii) after adding DMAP (42 mM, 20 equiv.).

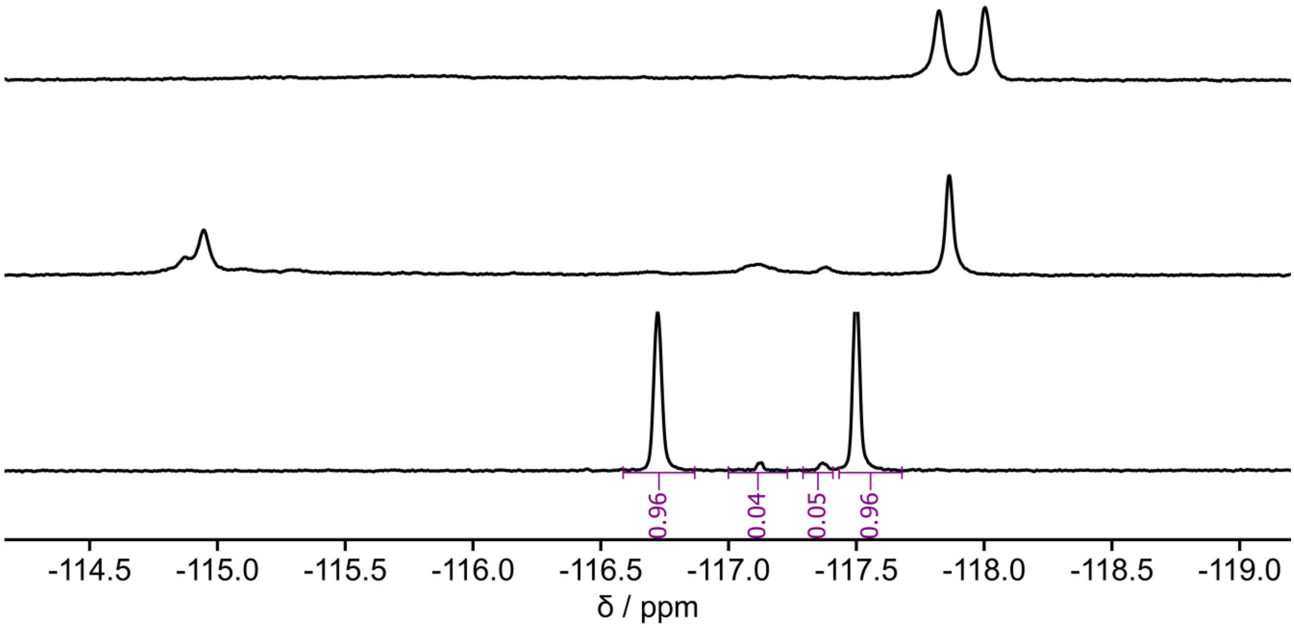

Figure S41. ^{19}F NMR (565 MHz, 298 K, DMSO-d_6) spectra of a sample of $[Pd_2(E\text{-}1)_4](BF_4)_4$ ([Pd] = 2.1 mM, 1 equiv.) i) before irradiation, ii) after irradiating with a 530 nm LED for 20 min, and iii) after adding DMAP (42 mM, 20 equiv.).

S7.4 ESI-MS spectra of $[Pd(Z\text{-}\mathbf{1})_2](BF_4)_2$ in DMSO-d_6

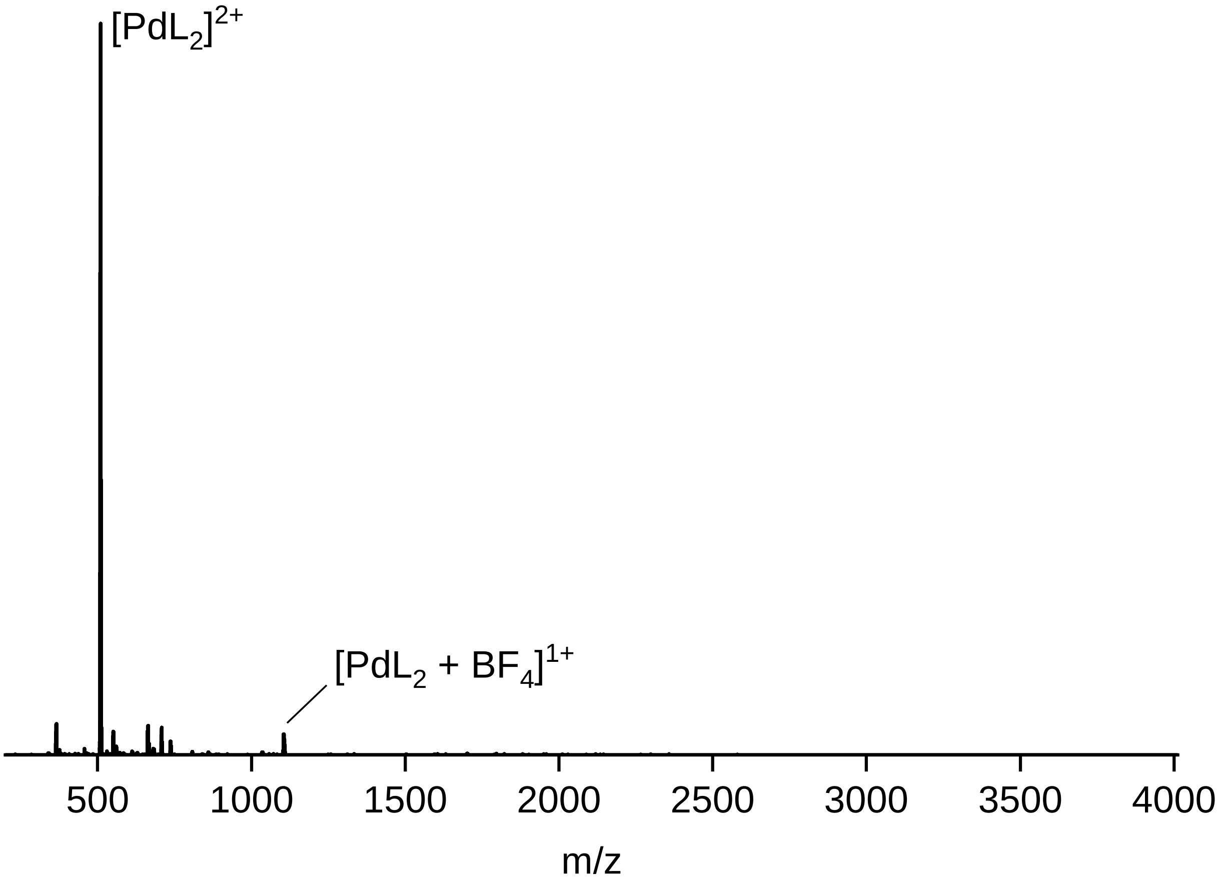

Figure S42. ESI-MS spectra of $[Pd(Z\text{-}\mathbf{1})_2](BF_4)_2$ ([Pd] = 2.3 mM) in DMSO-d_6.

Table S12. Zoom scans of select ESI-MS peaks, with simulated ESI-MS isotope patterns of $[Pd(Z\text{-}\mathbf{1})_2](BF_4)_2$ ([Pd] = 2.3 mM).

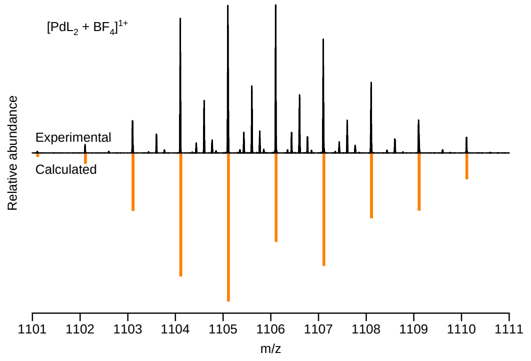	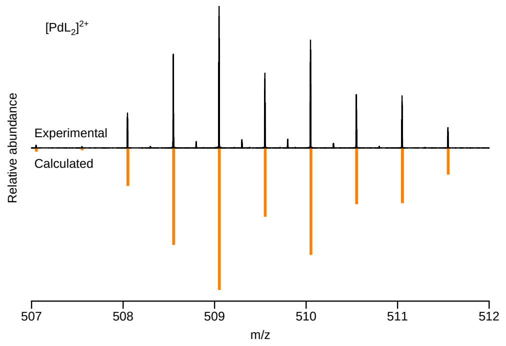
$[Pd(\mathbf{1})_2 + BF_4]^{1+}$ (major) + $[Pd_2(\mathbf{1})_4 + (BF_4)_2]^{2+}$ (minor)	$[Pd(\mathbf{1})_2]^{2+}$ (major) + $[Pd_2(\mathbf{1})_4]^{4+}$ (minor)

S7.5 ESI-MS spectra of [Pd(*Z*-1)$_2$](BAr$_F$)$_2$ in MeCN

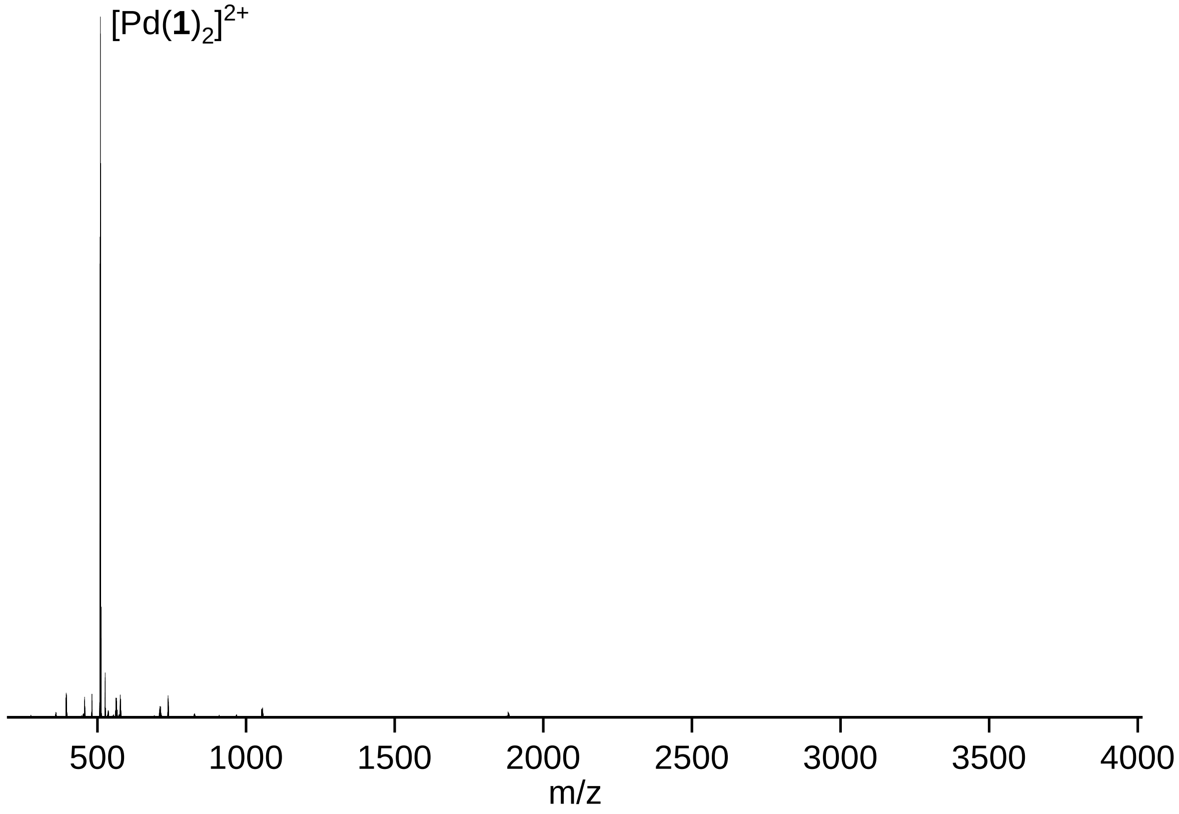

Figure S43. ESI-MS spectra of [Pd(*Z*-**1**)$_2$](BAr$_F$)$_2$ ([Pd] = 2.1 mM) in MeCN.

Table S13. Zoom scans of select ESI-MS peaks, with simulated ESI-MS isotope patterns of [Pd(*Z*-**1**)$_2$](BAr$_F$)$_2$ ([Pd] = 2.1 mM).

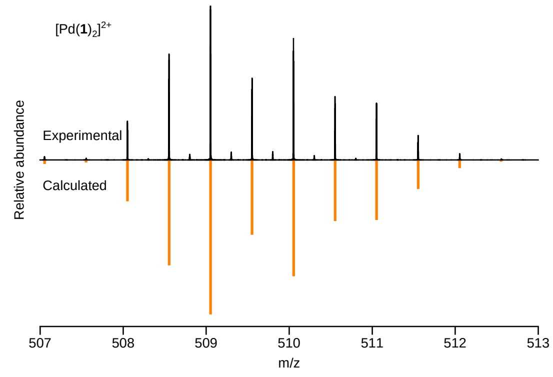	
[Pd(**1**)$_2$]$^{2+}$ (major) + [Pd$_2$(**1**)$_4$]$^{4+}$ (minor)	

S8. Synthesis and characterization of ligand 2

S8.1 Synthesis of ligand 2

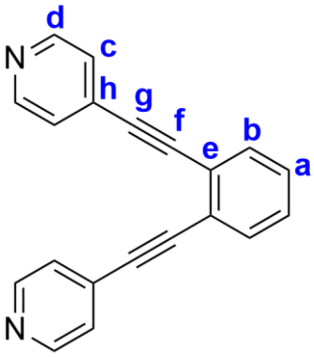

2

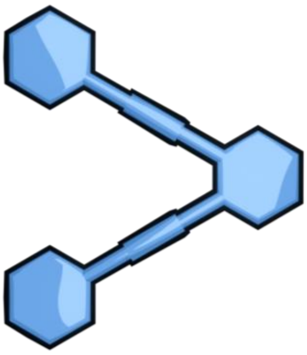

Ligand **2** was synthesised following modified literature procedure.[13]

1,2-Dibromobenzene (0.4 mL, 3.4 mmol, 1 equiv.) and 4-ethynylpyridine (1 g, 9.8 mmol, 2.9 equiv.) were dissolved in toluene (10 mL) and diisopropylamine (20 mL). The reaction mixture was degassed with argon for 20 minutes. CuI (80 mg, 0.4 mmol, 12 mol%) and $Pd(PPh_3)_2Cl_2$ (360 mg, 0.5 mmol, 10 mol%) were added, and the sample was stirred at 80 °C for 1 day. The reaction mixture was filtered through Celite. The Celite was washed DCM (50 mL), organic phases were washed with water (3 × 50 mL), saturated aqueous NaCl solution (50 mL), then dried over $MgSO_4$ and filtered through a Kim wipe and the solvent was removed under reduced pressure. The product was isolated using flash chromatography (SiO_2, 0-100% acetone in DCM), affording the product as an off-white solid (220 mg, 0.8 mmol, 23%). Spectroscopic data matches that reported.[13]

^{1}H NMR (600 MHz, DMSO-d_6) δ 8.66 (dd, J = 4.5, 1.6 Hz, 4H, H^{d}), 7.75 (dd, J = 5.8, 3.3 Hz, 2H, H^{b}), 7.57 (dd, J = 5.8, 3.4 Hz, 2H, H^{a}), 7.53 (dd, J = 4.4, 1.6 Hz, 4H, H^{c}).

^{13}C NMR (151 MHz, DMSO-d_6) δ 150.1 (C^{d}), 132.4 (C^{b}), 129.9 (C^{a}), 129.7 (C^{h}), 125.2 (C^{c}), 123.9 (C^{e}), 91.5 (C^{f}), 90.8 (C^{g}).

ESI-MS (m/z): 281.1071 $[M + H]^+$ (calculated: 281.1079)

S8.2 1D and 2D NMR spectra of 2 in DMSO-d_6

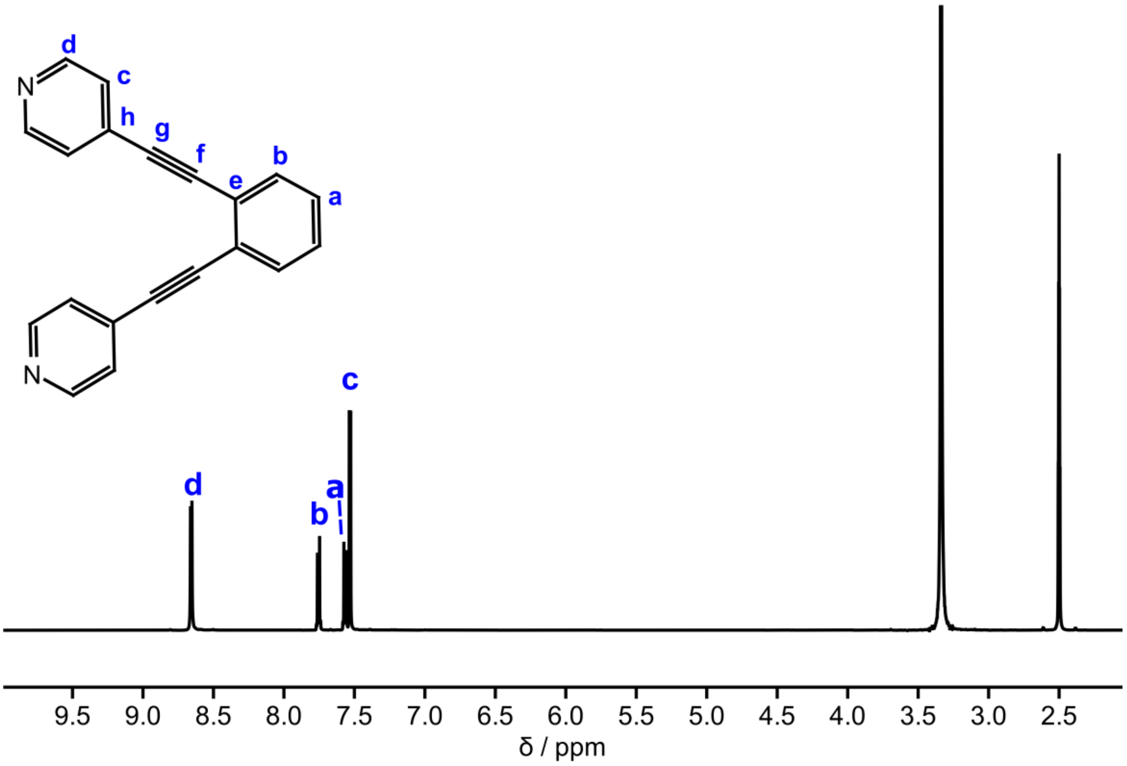

Figure S44. ^{1}H NMR (600 MHz, 298 K, DMSO-d_6) spectrum of ligand **2**.

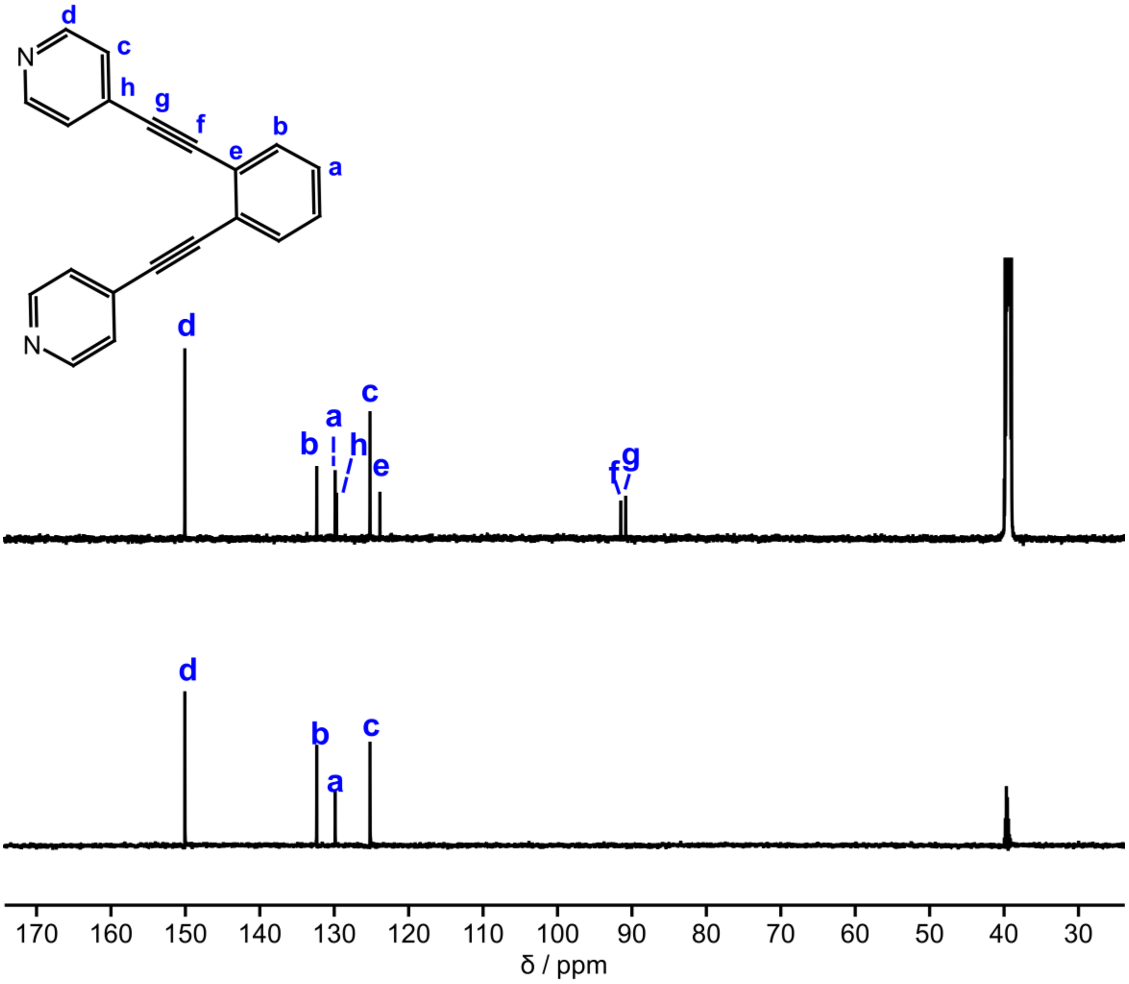

Figure S45. $^{13}C\{^{1}H\}$ (top) and DEPT-135 (bottom) NMR (151 MHz, 298 K, DMSO-d_6) spectrum of ligand **2.**

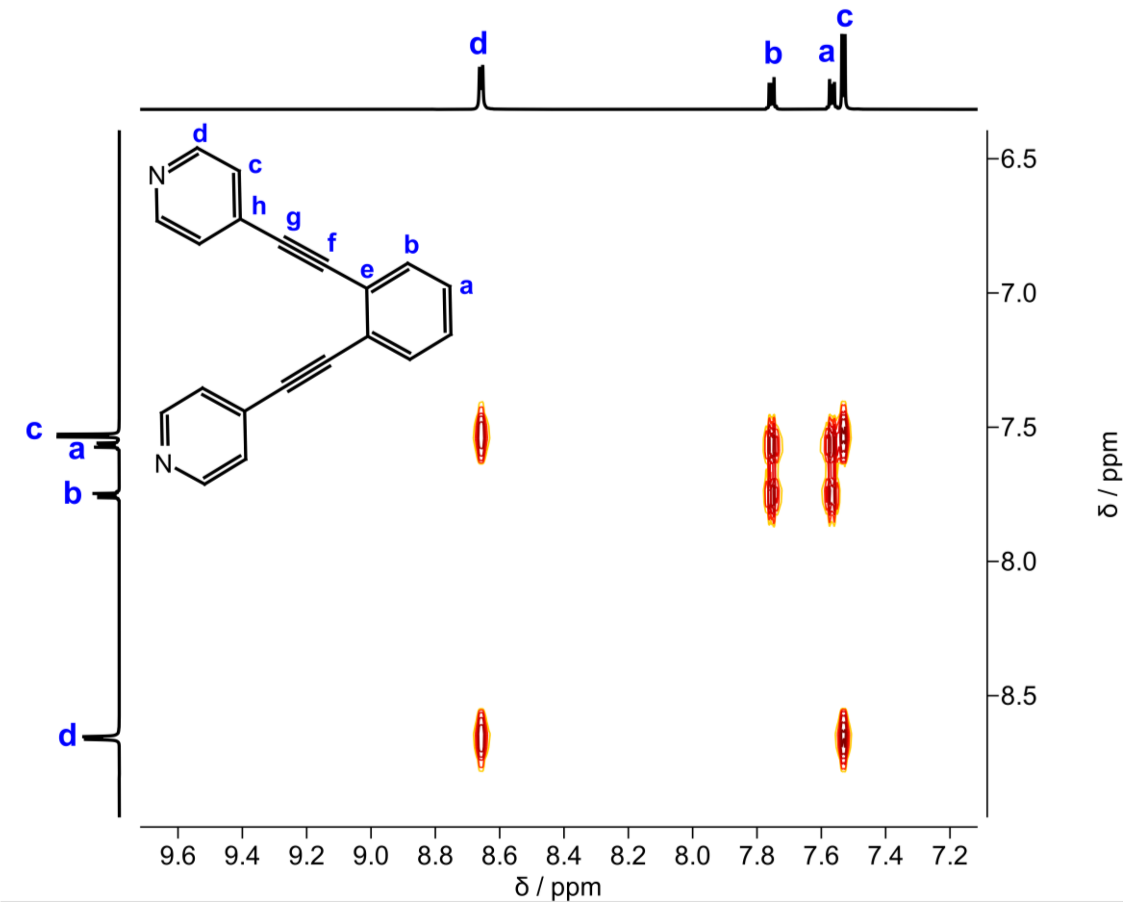

Figure S46. ^{1}H-^{1}H COSY NMR (600 MHz, 298 K, DMSO-d_6) spectrum of ligand **2.**

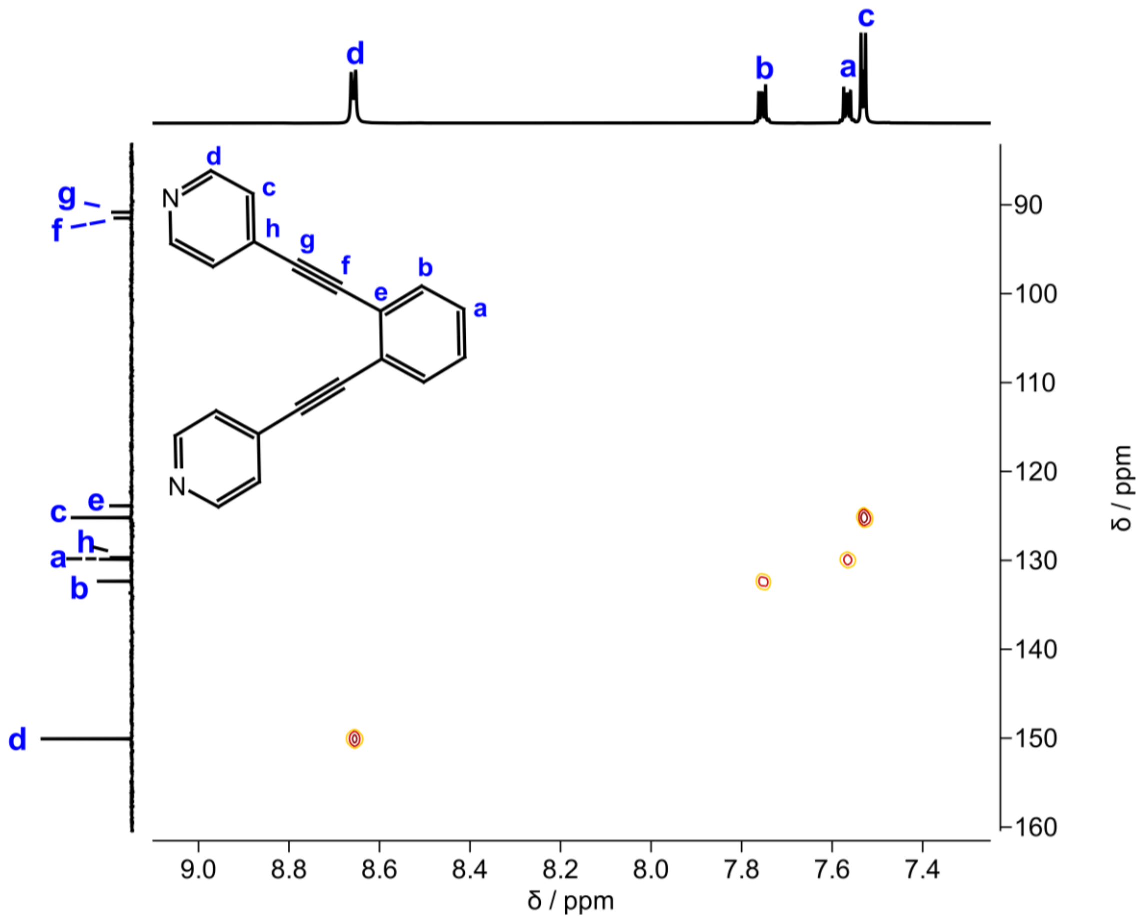

Figure S47. ^{1}H-^{13}C HSQC NMR (600 MHz, 151 MHz, 298 K, DMSO-d_6) spectrum of ligand **2.**

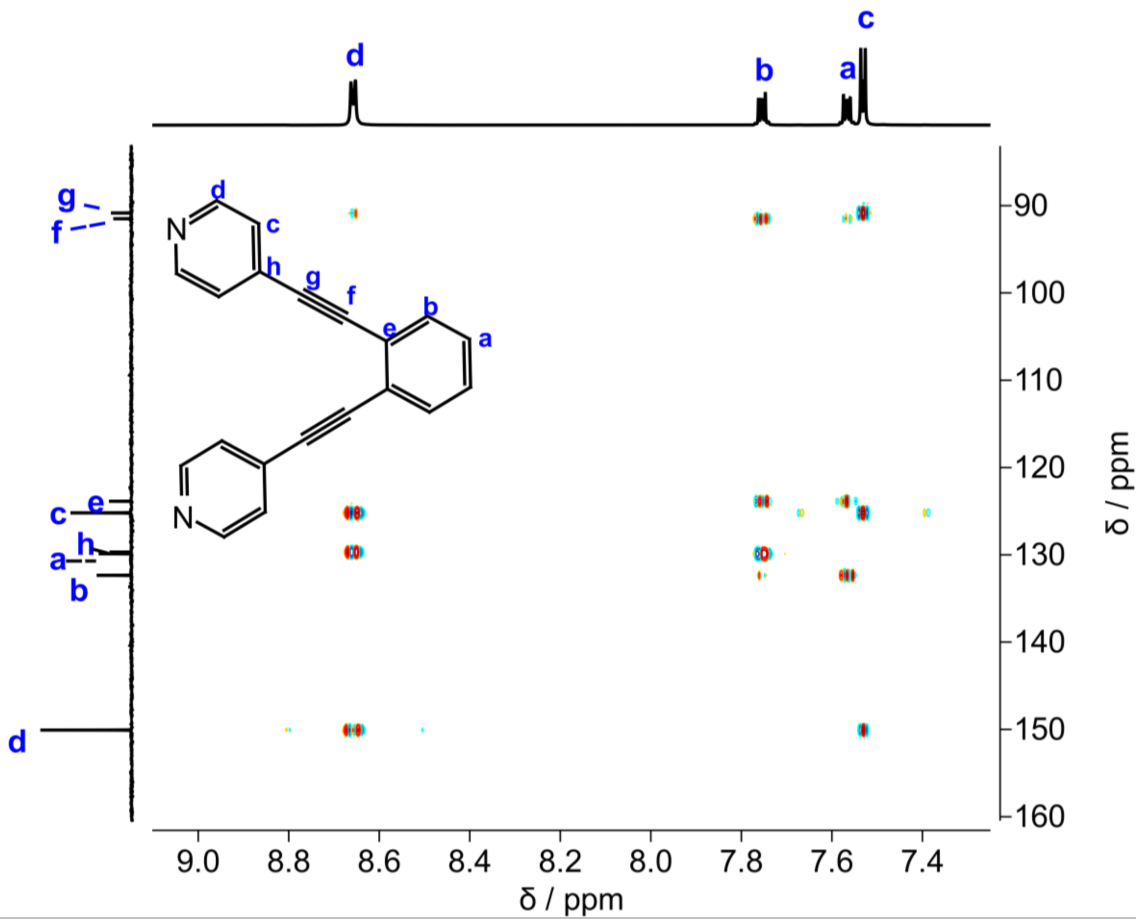

Figure S48. ^{1}H-^{13}C HMBC NMR (600 MHz, 151 MHz, 298 K, DMSO-d_6) spectrum of ligand **2**.

S9. Synthesis and characterization of homoleptic species $[Pd_4(2)_8]^{8+}$ and $[Pd_3(2)_6]^{6+}$

S9.1 Synthesis of $[Pd_4(2)_8](BF_4)_8$ in DMSO

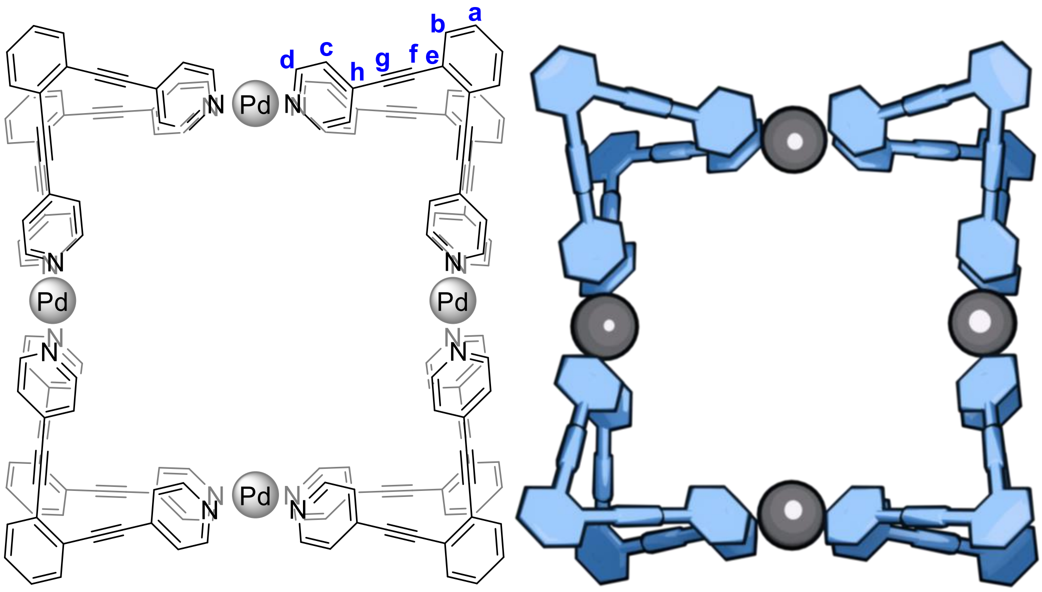

A solution of ligand **2** (1 equiv., 500 µL, 4.9 mM, DMSO-d_6) was prepared. A solution of $[Pd(MeCN)_4](BF_4)_2$ (0.5 equiv., 24 µL, 52 mM, DMSO-d_6) was added. The sample equilibrated by heating in a water bath at 50 °C for 20 min to give near quantitative formation of $[Pd_4(\mathbf{2})_8](BF_4)_8$ ([**2**] = 4.7 mM, [Pd] = 2.3 mM).

^{1}H NMR (600 MHz, DMSO-d_6) δ 9.26 (d, J = 6.4 Hz, 32H, H^d), 7.97 (d, J = 6.7 Hz, 32H, H^c), 7.74 (dd, J = 5.8, 3.3 Hz, 16H, H^b), 7.64 (dd, J = 5.9, 3.3 Hz, 16H, H^a).

^{13}C NMR (151 MHz, DMSO-d_6) δ 150.9 (C^d), 133.9 (C^h), 132.8 (C^b), 131.0 (C^a), 129.0 (C^c), 123.0 (C^e), 95.6 (C^f), 89.1 (C^g).

Table S14. Comparison of NMR environments between ligand **2** and $[Pd_4(\mathbf{2})_8](BF_4)_8$ in DMSO-d_6.

	$^1H^a$		$^{13}C^b$	
Environment	**2**	$[Pd_4(\mathbf{2})_8](BF_4)_8$	**2**	$[Pd_4(\mathbf{2})_8](BF_4)_8$
a	7.57	7.64	129.9	131.0
b	7.75	7.74	132.5	132.8
c	7.53	7.97	125.2	129.0
d	8.66	9.26	150.1	150.9
e	-	-	123.9	123.0
f	-	-	91.5	95.6
g	-	-	90.8	89.1
h	-	-	129.7	133.9

^{a}(600 MHz, 298 K, DMSO-d_6), ^{b}(151 MHz, 298 K, DMSO-d_6),

S9.2 1D and 2D NMR spectra of $[Pd_4(2)_8](BF_4)_8$ in DMSO-d_6

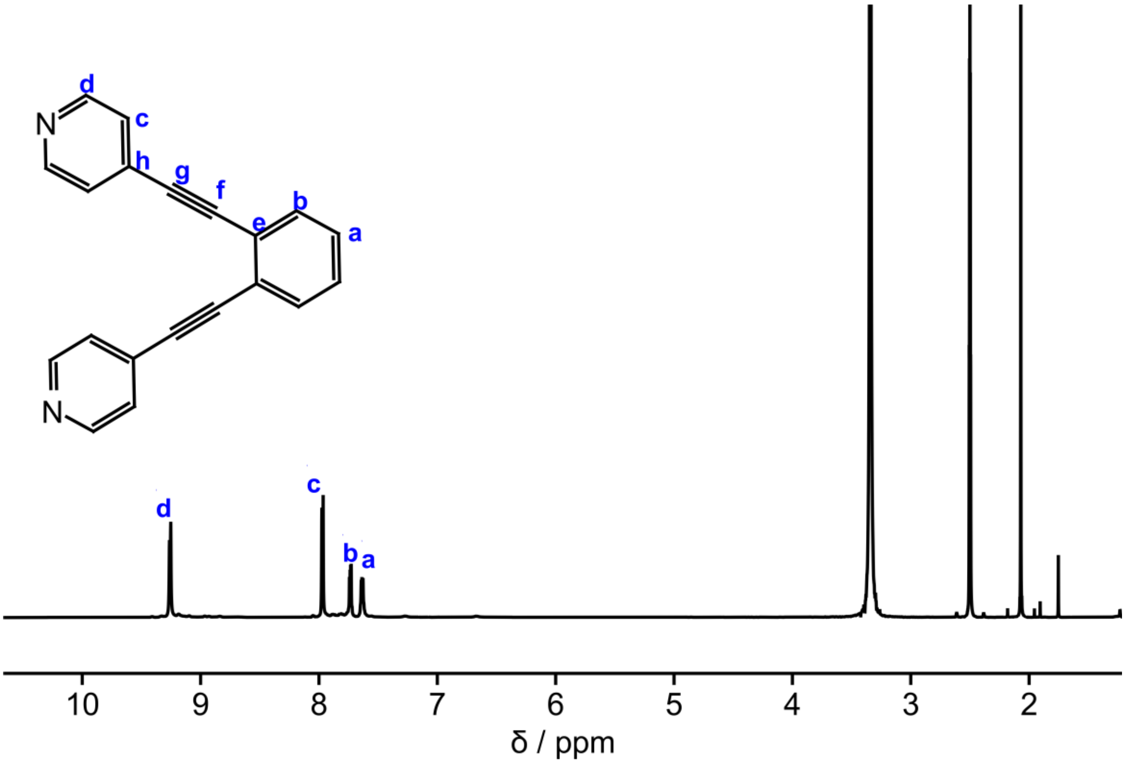

Figure S49. ^{1}H NMR (600 MHz, 298 K, DMSO-d_6) spectrum of $[Pd_4(\mathbf{2})_8](BF_4)_8$ ([Pd] = 2.3 mM).

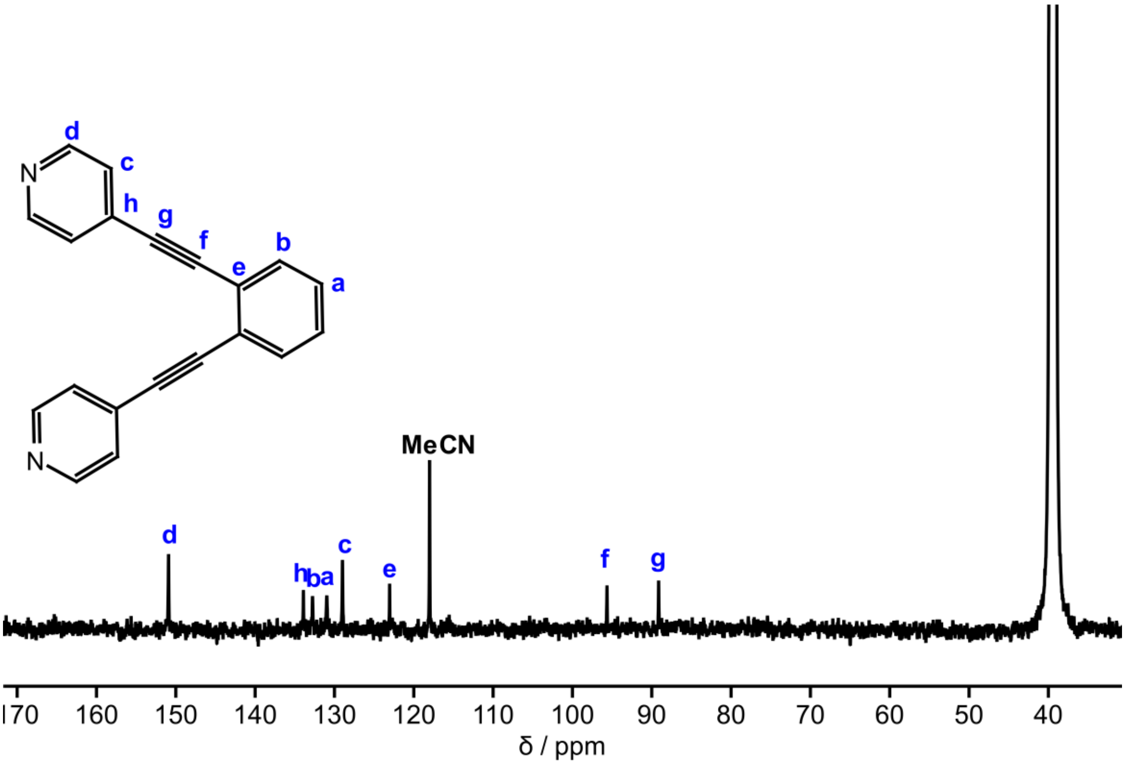

Figure S50. $^{13}C\{^1H\}$ NMR (151 MHz, 298 K, DMSO-d_6) spectrum of $[Pd_4(\mathbf{2})_8](BF_4)_8$ ([Pd] = 2.3 mM).

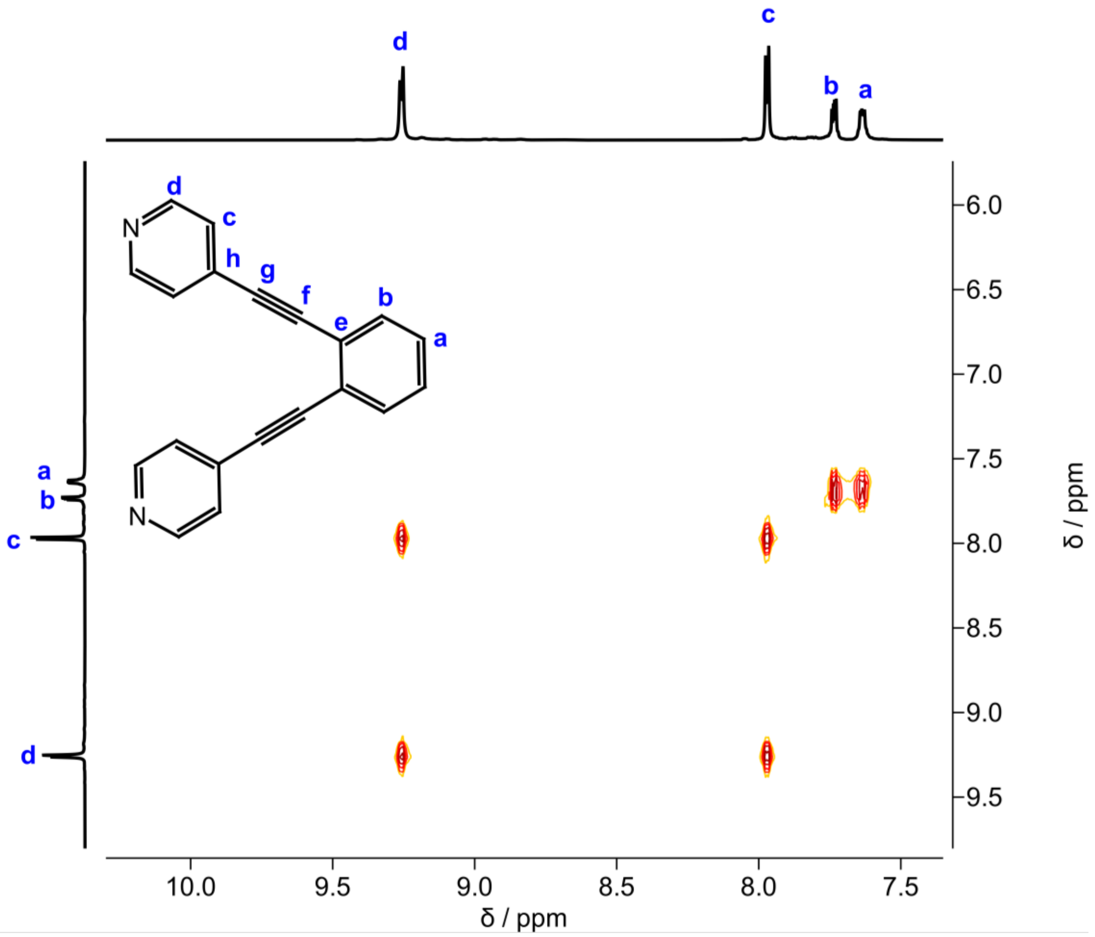

Figure S51. ^1H-^1H COSY NMR (600 MHz, 298 K, DMSO-d_6) spectrum of $[Pd_4(\mathbf{2})_8](BF_4)_8$ ([Pd] = 2.3 mM).

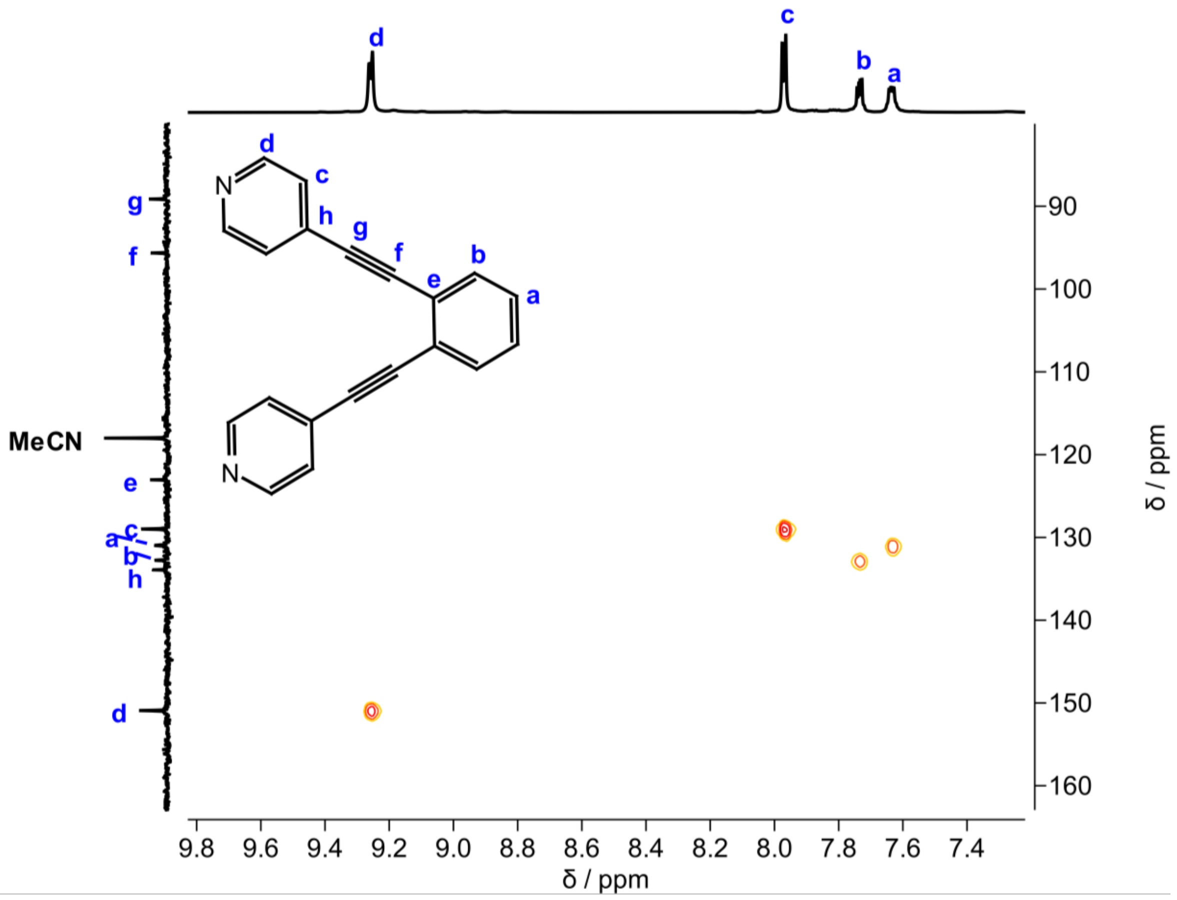

Figure S52. ^1H-^{13}C HSQC NMR (600 MHz, 151 MHz, 298 K, DMSO-d_6) spectrum of $[Pd_4(\mathbf{2})_8](BF_4)_8$ ([Pd] = 2.3 mM).

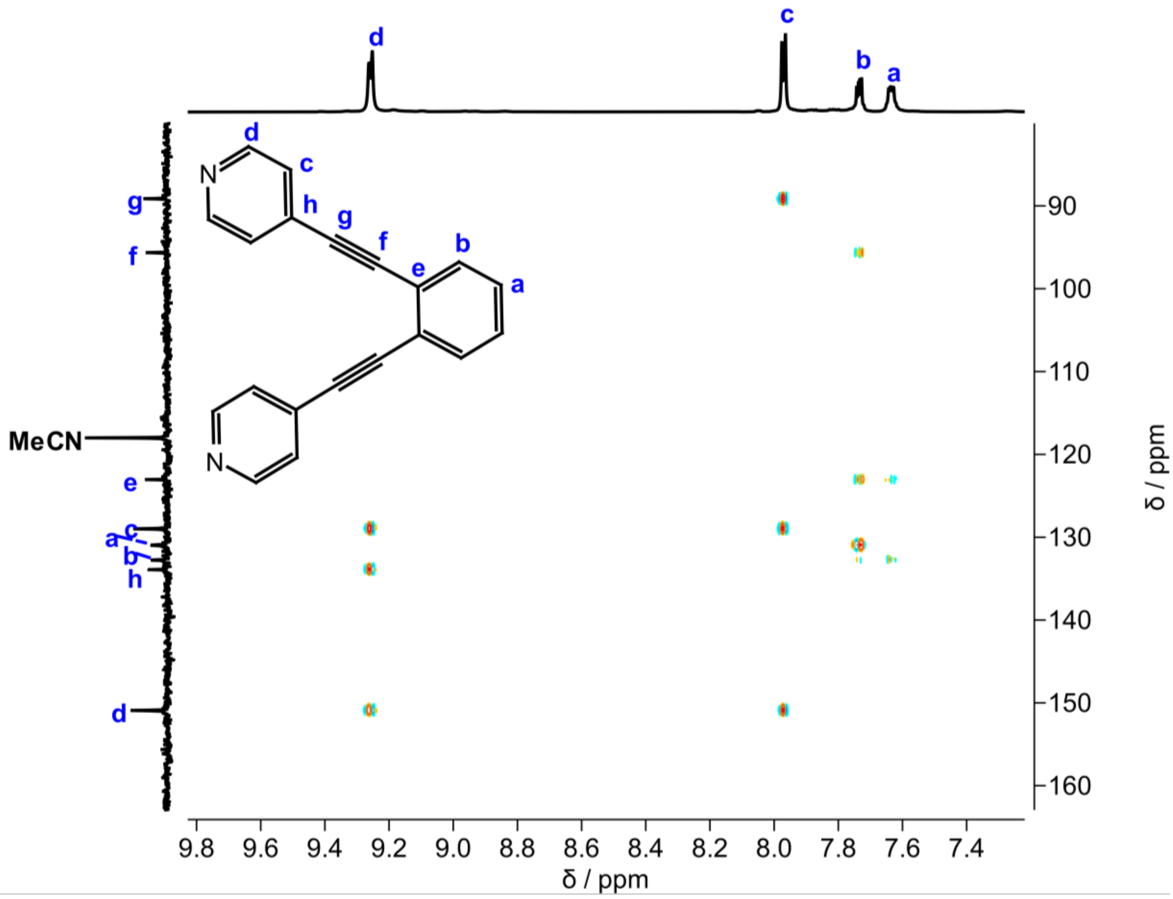

Figure S53. ^{1}H-^{13}C HMBC NMR (600 MHz, 151 MHz, 298 K, DMSO-d_6) spectrum of $[Pd_4(\mathbf{2})_8](BF_4)_8$ ([Pd] = 2.3 mM).

S9.3 ESI-MS spectrum of $[Pd_4(\mathbf{2})_8](BF_4)_8$ in DMSO

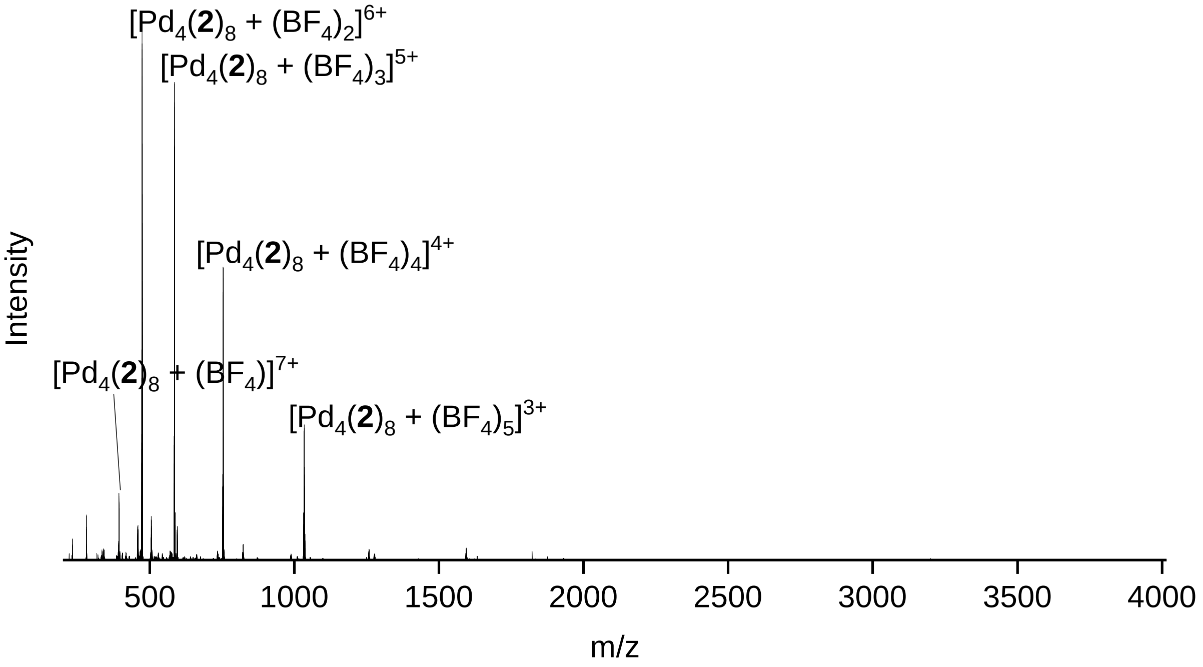

Figure S54. ESI-MS spectrum of $[Pd_4(\mathbf{2})_8](BF_4)_8$ ([Pd] = 2.3 mM) in DMSO.

Table S15. Zoom scans of select ESI-MS peaks, with simulated isotope patterns ESI-MS of $[Pd_4(\mathbf{2})_8](BF_4)_8$ ([Pd] = 2.3 mM) in DMSO-d_6.

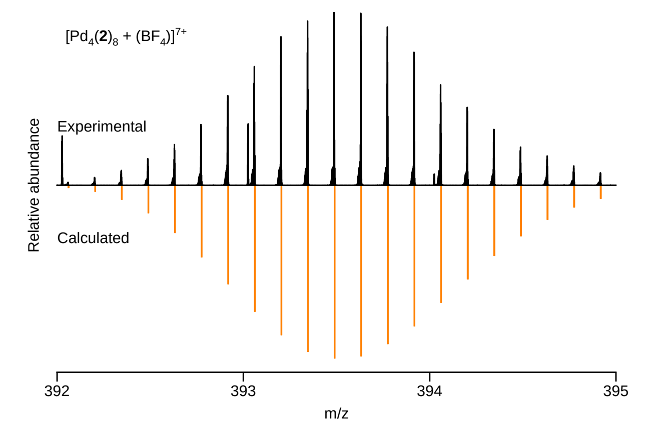

$[Pd_4(\mathbf{2})_8 + (BF_4)]^{7+}$

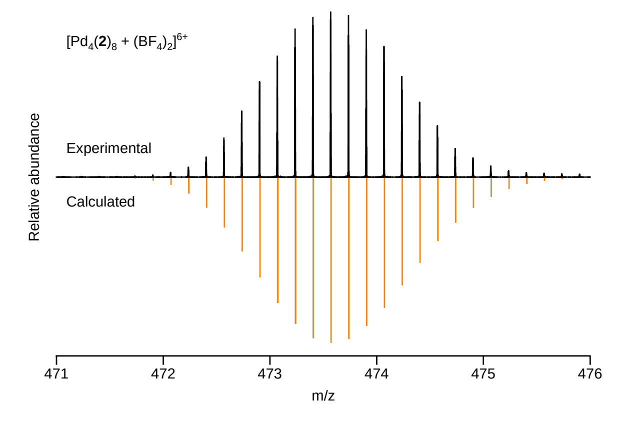

$[Pd_4(\mathbf{2})_8 + (BF_4)_2]^{6+}$

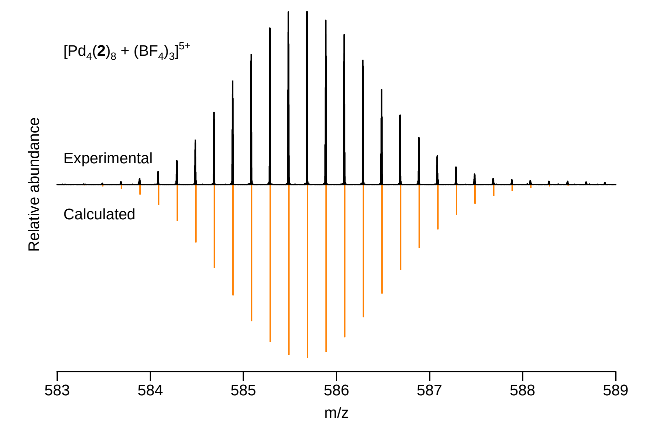

$[Pd_4(\mathbf{2})_8 + (BF_4)_3]^{5+}$

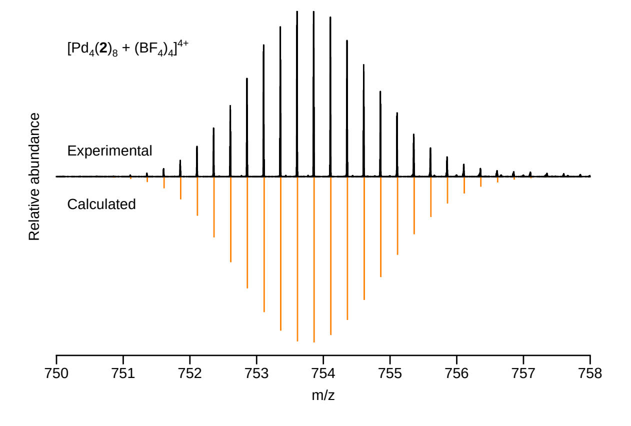

$[Pd_4(\mathbf{2})_8 + (BF_4)_4]^{4+}$

S9.4 Synthesis of $[Pd_3(2)_6](BAr_F)_6$ in MeCN

Ligand **2** (4.03 mg, 14.4 µmol, 2.0 equiv.) was dissolved in MeCN-d_3 (3.4 mL) and a solution of $[Pd(Py^*)_4](BAr_F)_2$ (300 µL, 23.9 mM, 1.0 equiv.) was added. The solution was equilibrated at 50 °C for 30 minutes to afford a stock solution of $[Pd_3(\mathbf{2})_6](BAr_F)_6$ ([Pd] = 2.0 mM) and free Py*. An NMR sample tube was charged with the solution of $[Pd_3(\mathbf{2})_6](BAr_F)_6$ (500 µL) and a ^1H NMR spectrum was collected (Figure S55).

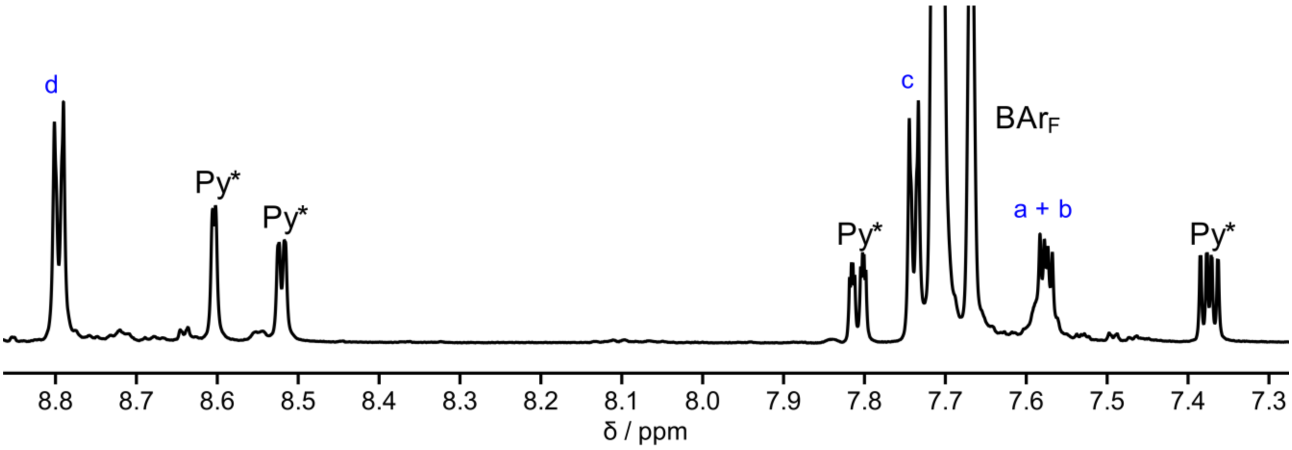

Figure S55. ^1H NMR (600 MHz, 298 K, MeCN-d_3) spectrum of $[Pd_3(\mathbf{2})_6](BAr_F)_6$ and free Py*.

S9.5 ESI-MS spectrum of $[Pd_3(2)_6](BAr_F)_6$ and $[Pd_4(2)_6](BAr_F)_8$ in MeCN

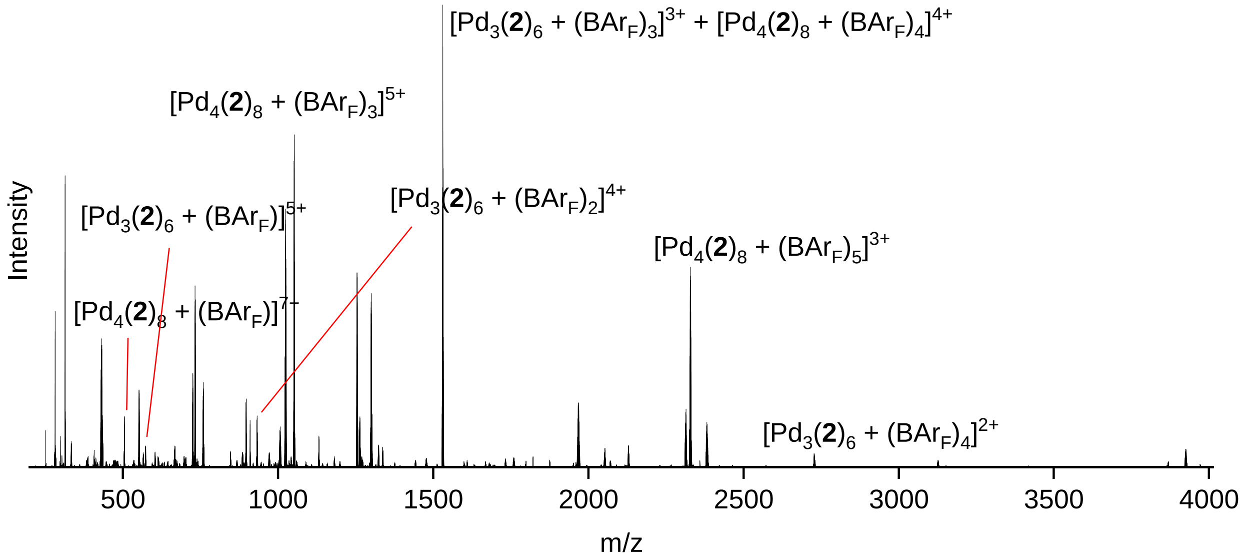

Figure S56. ESI-MS spectrum of a mixture $[Pd_3(\mathbf{2})_6](BAr_F)_8$ and $[Pd_4(\mathbf{2})_8](BAr_F)_8$ ([Pd] = 2.0 mM) in MeCN.

Table S16. Zoom scans of select ESI-MS peaks, with simulated isotope patterns ESI-MS of $[Pd_3(\mathbf{2})_6](BAr_F)_8$ and $[Pd_4(\mathbf{2})_8](BAr_F)_8$ ([Pd] = 2.0 mM) in MeCN.

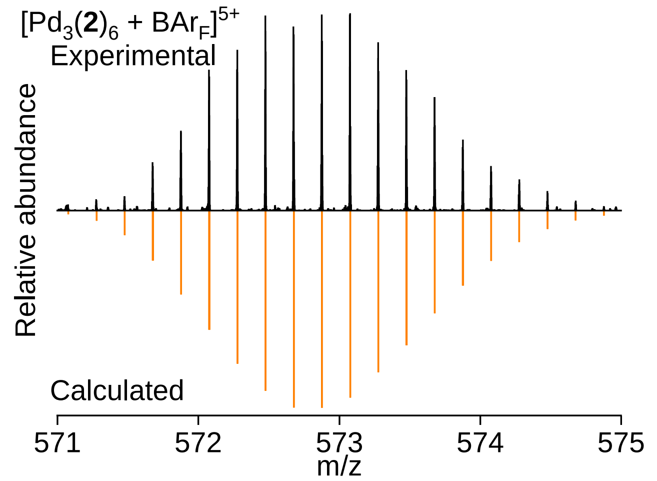

$[Pd_3(\mathbf{2})_6 + BAr_F]^{5+}$

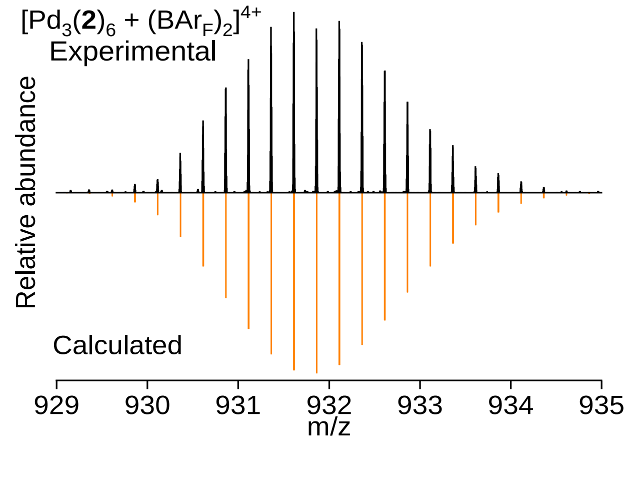

$[Pd_3(\mathbf{2})_6 + (BAr_F)_2]^{4+}$

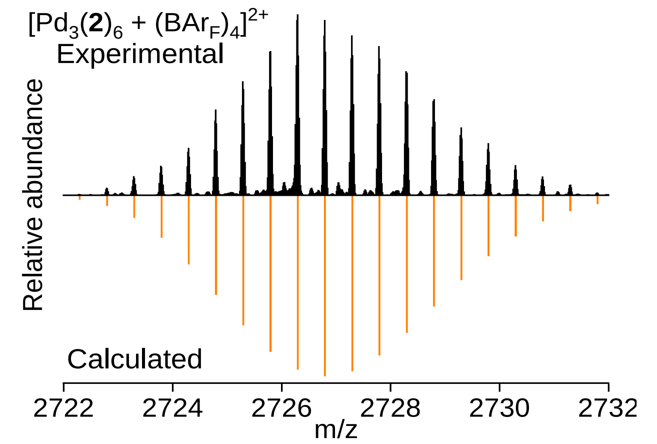

$[Pd_3(\mathbf{2})_6 + (BAr_F)_4]^{2+}$

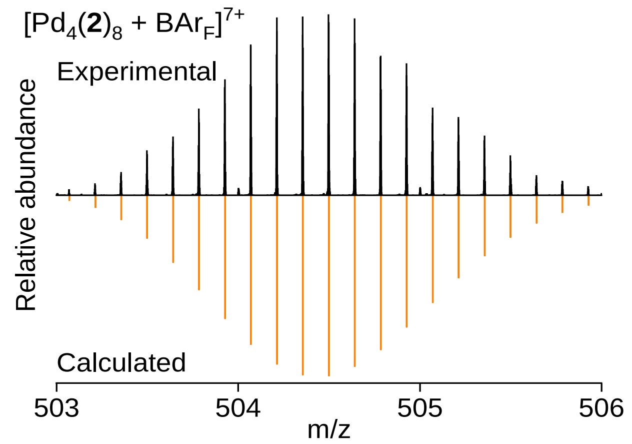

$[Pd_4(\mathbf{2})_8 + (BAr_F)]^{7+}$

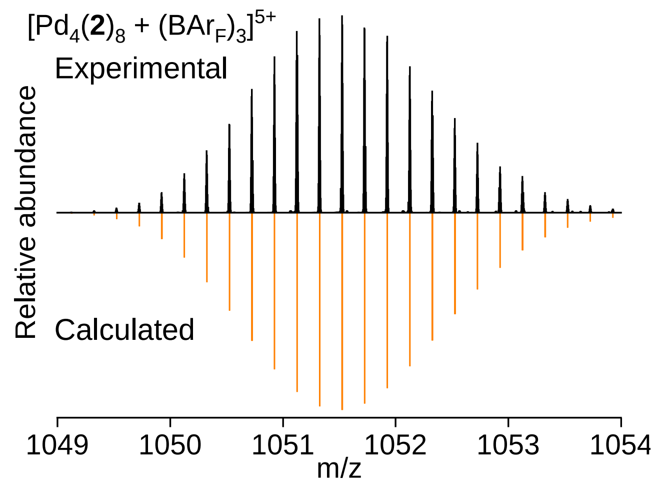

$[Pd_4(\mathbf{2})_8 + (BAr_F)_3]^{5+}$

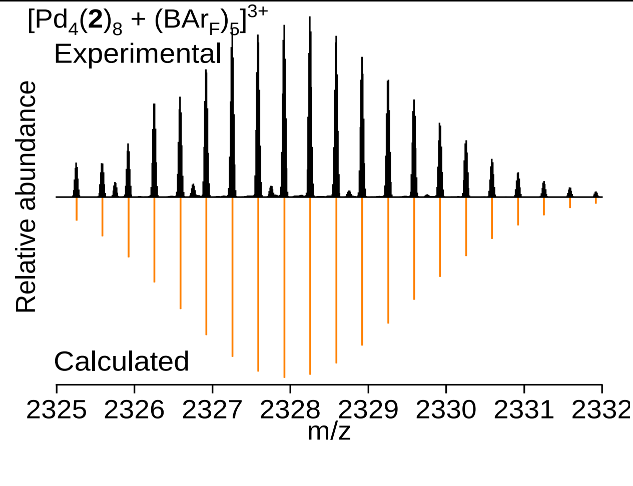

$[Pd_4(\mathbf{2})_8 + (BAr_F)_5]^{3+}$

S10. Synthesis and characterization of heteroleptic cage $[Pd_2(E\text{-}1)_2(2)_2]^{4+}$

S10.1 Synthesis of $[Pd_2(E\text{-}1)_2(2)_2](BF_4)_4$ in DMSO-d_6

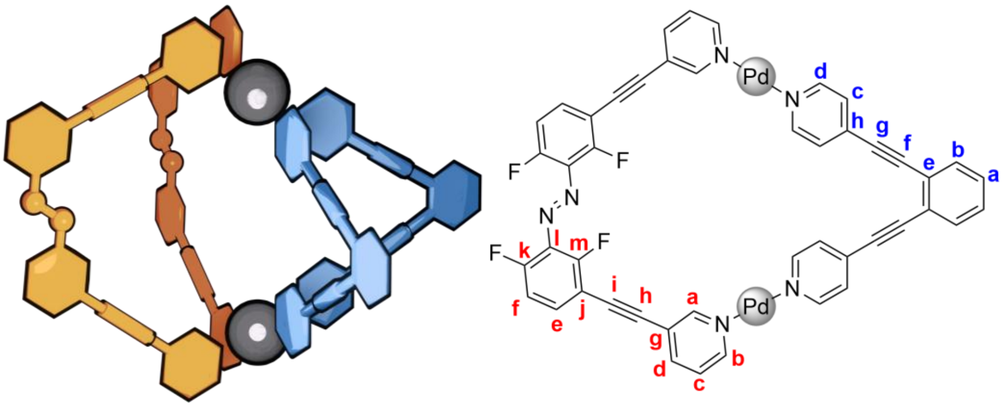

To assemble the heteroleptic cage, a solution of ligand **1** (280 µL, 4.6 mM, 1.0 equiv.) and ligand **2** (270 µL, 4.7 mM, 1.0 equiv.) was prepared in DMSO-d_6. A solution of $[Pd(MeCN)_4](BF_4)_2$ (25 µL, 52 mM, 1 equiv.) in DMSO-d_6 was added. The sample equilibrated within 10 minutes at room temperature. Comparing ^1H NMR spectra of free ligand **1**, $[Pd_2(E\text{-}\mathbf{1})_4]^{4+}$, free ligand **2**, and $[Pd_4(\mathbf{2})_8]^{8+}$ shows the quantitative assembly of a single self-assembled product. A ^1H-^1H ROESY NMR signal between H^{1a} and H^{2d} was observed demonstrating the proximity between the two ligands, consistent with the formation of a heteroleptic product.

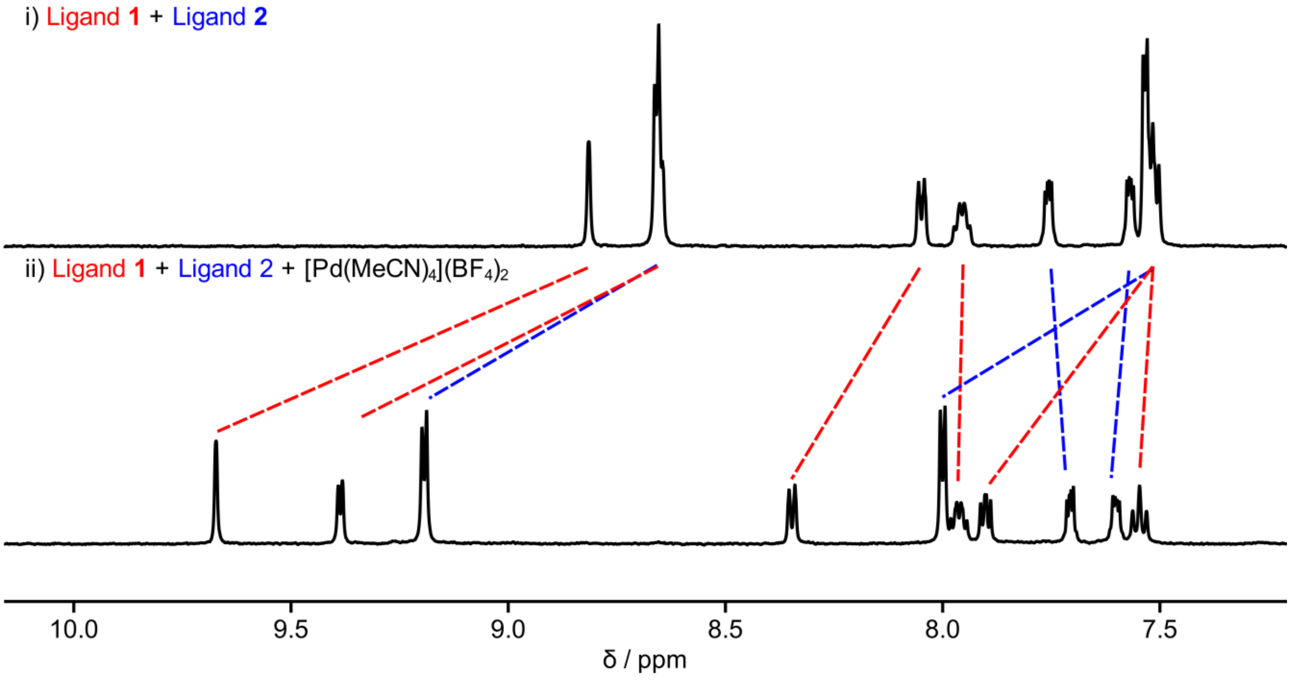

Figure S57. ^1H NMR (600 MHz, 298 K, DMSO-d_6) of i) ligand **1** (4.6 mM, 1.0 equiv.) and ligand **2** (4.6 mM, 1.0 equiv.), and ii) the sample after adding $[Pd(MeCN)_4](BF_4)_2$ (4.6 mM, 1 equiv.).

The heteroleptic cage $[Pd_2(E\text{-}\mathbf{1})_2(\mathbf{2})_2](BF_4)_4$ can also be synthesized by reacting homoleptic $[Pd_2(E\text{-}\mathbf{1})_4](BF_4)_4$ ([Pd] = 2.1 mM, 250 µL in DMSO-d_6, 0.53 µmol, 1.0 equiv.) with $[Pd_4(\mathbf{2})_8](BF_4)_8$ ([Pd] = 2.1 mM, 250 µL in DMSO-d_6, 0.53 µmol, 1.0 equiv.). The sample was prepared in the dark and heated with a heat gun to 150 °C for 5 minutes to thermally equilibrate the sample, quantitatively affording $[Pd_2(E\text{-}\mathbf{1})_2(\mathbf{2})_2](BF_4)_4$.

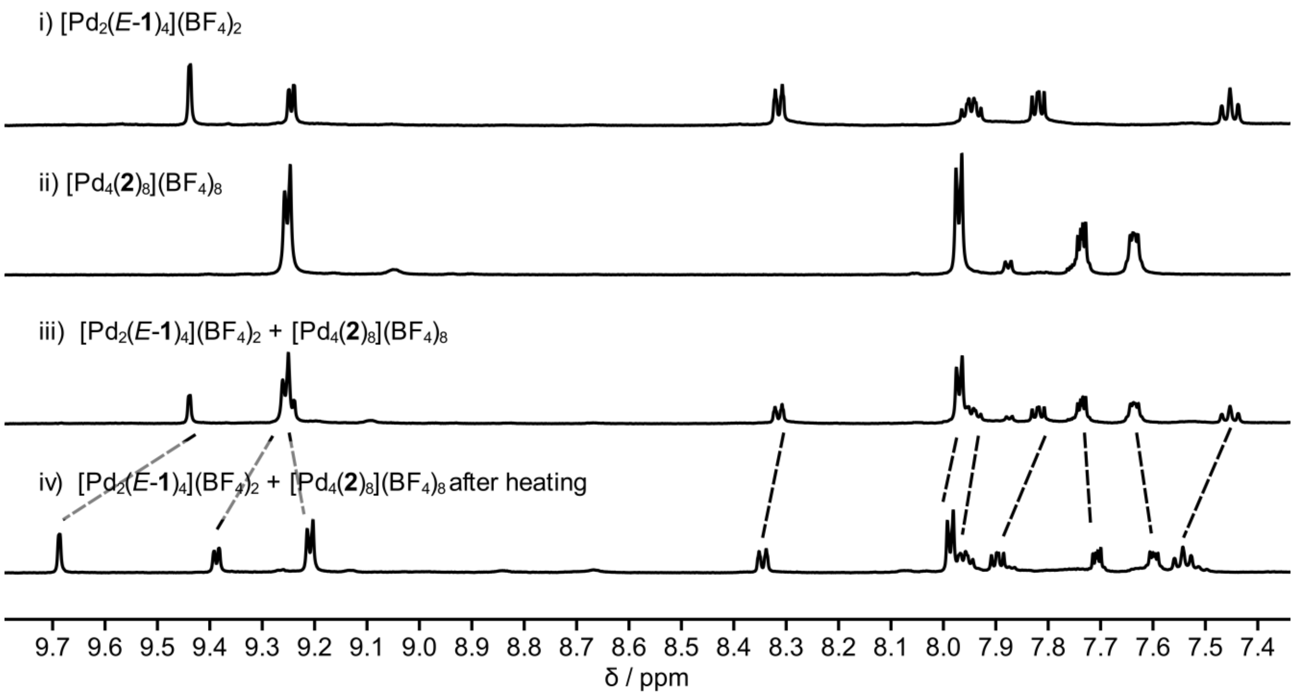

Figure S58. ^{1}H NMR (600 MHz, 298 K, DMSO-d_6) spectra of i) $[Pd_2(E\text{-}\mathbf{1})_4](BF_4)_4$ ([Pd] = 2.1 mM), ii) $[Pd_4(\mathbf{2})_8](BF_4)_8$ ([Pd] = 2.1 mM), iii) a mixture of $[Pd_2(E\text{-}\mathbf{1})_4](BF_4)_4$ ([Pd] = 2.1 mM, 1.0 equiv.) and $[Pd_4(\mathbf{2})_8](BF_4)_8$ ([Pd] = 2.1 mM, 1.0 equiv.), and iv) the previous sample heating at 150°C for 5 minutes.

Table S17. Chemical shift (ppm) of ^{1}H, ^{13}C, and ^{19}F NMR environments for ligand **1** in $[Pd_2(E\text{-}\mathbf{1})_2(\mathbf{2})_2](BF_4)_4$.

	Ligand **1**		
Environment	^{1}H^a	^{19}F^b	^{13}C^c
a	9.68 (d, J = 1.93 Hz)	-	152.5
b	9.39 (dd, J = 5.86, 1.44 Hz)	-	151.0
c	7.90 (dd, J = 8.13, 5.76 Hz)	-	127.5
d	8.35 (dt, J = 8.09, 1.61 Hz)	-	143.0
e	7.96 (td, J = 8.23, 5.65 Hz)	-	136.9
f	7.55 (ddd, J = 10.41, 8.87, 1.46 Hz)	-	129.4
g	-	-	121.4
h	-	-	89.2
i	-	-	86.4
j	-	-	107.5
k	-	−115.82	155.5
l	-	-	130.6
m	-	−115.41	155.3

^{a}(600 MHz, 298 K, DMSO-d_6), ^{b}(565 MHz, 298 K, DMSO-d_6), ^{c}(151 MHz, 298 K, DMSO-d_6).

Table S18. Chemical shift (ppm) of ^{1}H and ^{13}C environments for ligand **2** in $[Pd_2(E\text{-}\mathbf{1})_2(\mathbf{2})_2](BF_4)_4$.

	Ligand **2**	
Environment	$^{1}H^{a}$	$^{13}C^{b}$
a	7.60 (dd, *J* = 5.94, 3.19 Hz)	131.0
b	7.70 (dt, *J* = 7.53, 3.76 Hz)	133.1
c	8.00 (d, *J* = 6.66 Hz)	129.4
d	9.20 (d, *J* = 6.67 Hz)	150.7
e	-	123.1
f	-	95.6
g	-	89.2
h	-	133.7

[a](600 MHz, 298 K, DMSO-*d*6), [b](151 MHz, 298 K, DMSO-*d*6).

S10.2 1D and 2D NMR spectra of $[Pd_2(E\text{-}1)_2(2)_2](BF_4)_4$ in DMSO-d_6

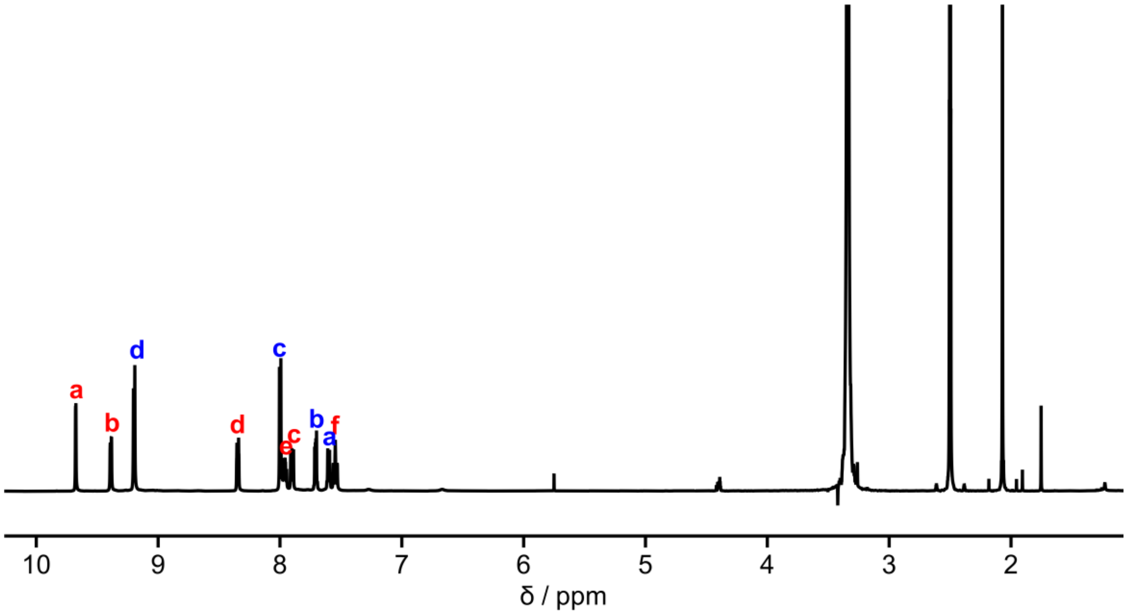

Figure S59. ^{1}H NMR (600 MHz, 298 K, DMSO-*d*6) spectrum of $[Pd_2(E\text{-}\mathbf{1})_2(\mathbf{2})_2](BF_4)_4$ ([Pd] = 2.3 mM).

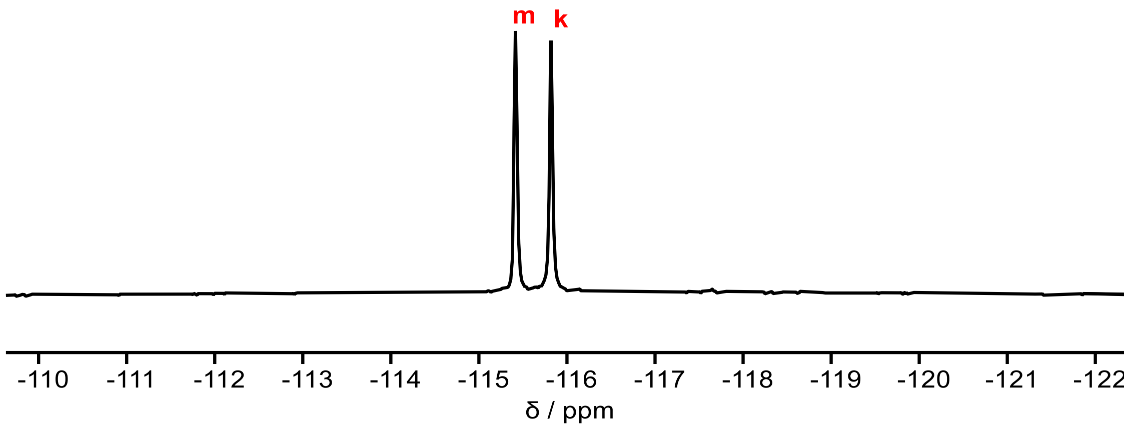

Figure S60. ^{19}F NMR (565 MHz, 298 K, DMSO-*d*6) spectrum of $[Pd_2(E\text{-}\mathbf{1})_2(\mathbf{2})_2](BF_4)_4$ ([Pd] = 2.3 mM).

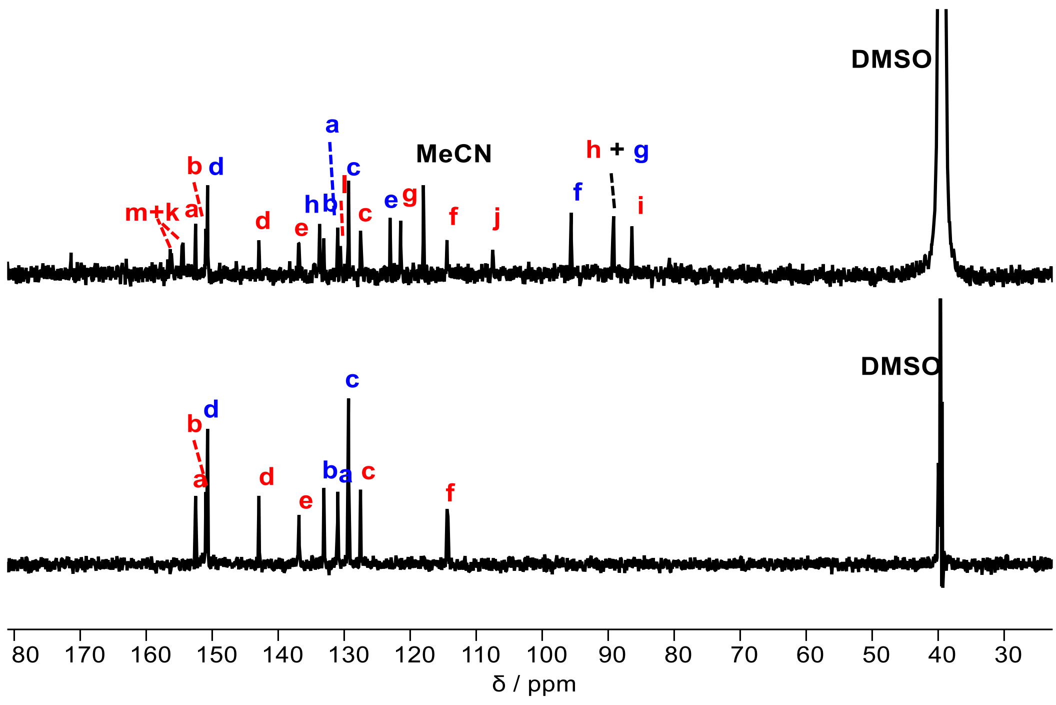

Figure S61. $^{13}C\{^{1}H\}$ (top) and DEPT-135 (bottom) NMR (151 MHz, 298 K, DMSO-d_6) spectra of $[Pd_2(E\text{-}1)_2(2)_2](BF_4)_4$ ([Pd] = 2.3 mM).

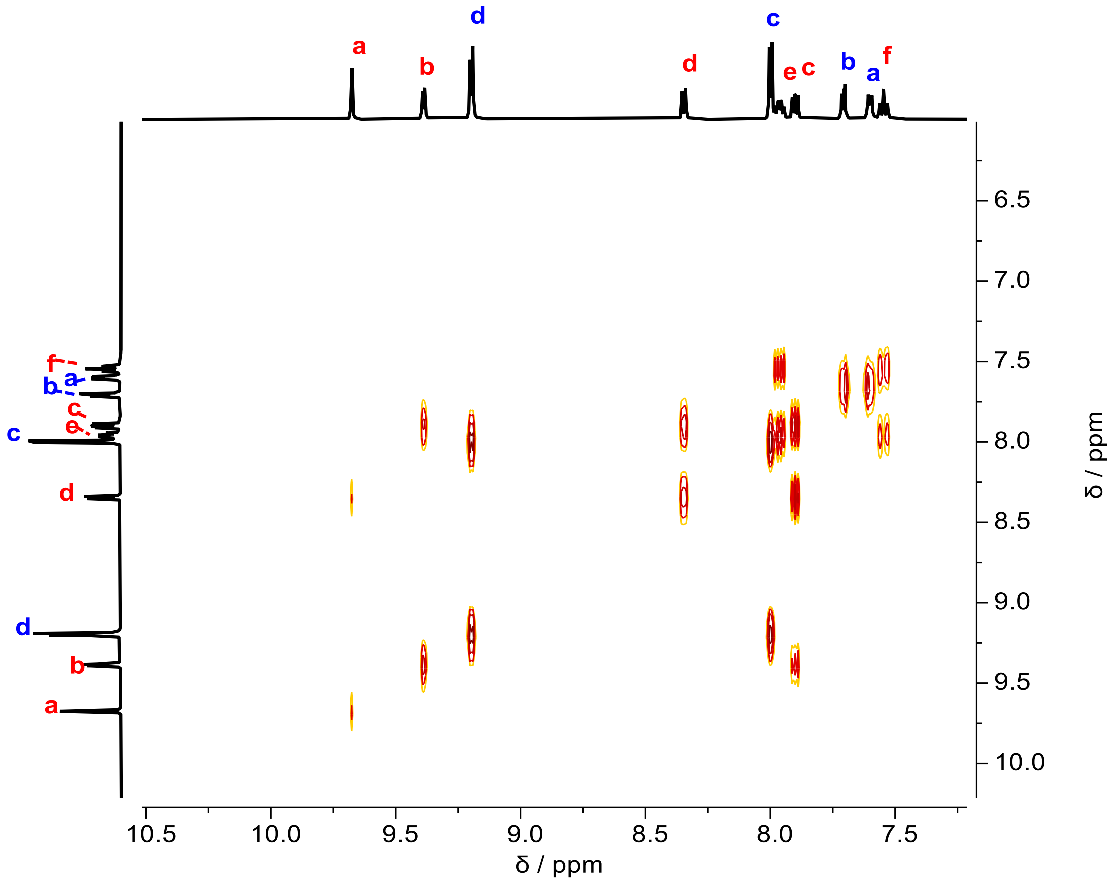

Figure S62. ^{1}H-^{1}H COSY NMR (600 MHz, 298 K, DMSO-d_6) spectrum of $[Pd_2(E\text{-}1)_2(2)_2](BF_4)_4$ ([Pd] = 2.3 mM).

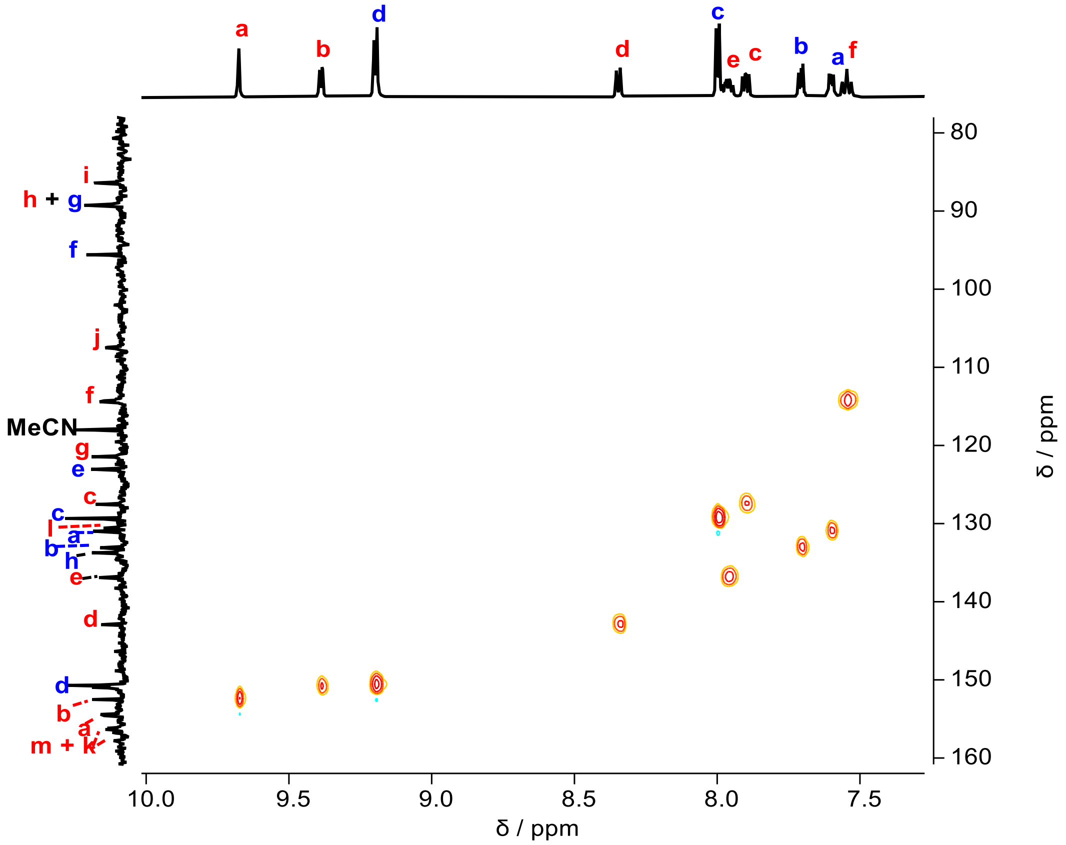

Figure S63. ^{1}H-^{13}C HSQC NMR (600 MHz, 151 MHz, 298 K, DMSO-d_6) spectrum of $[Pd_2(E\text{-}\mathbf{1})_2(\mathbf{2})_2](BF_4)_4$ ([Pd] = 2.3 mM).

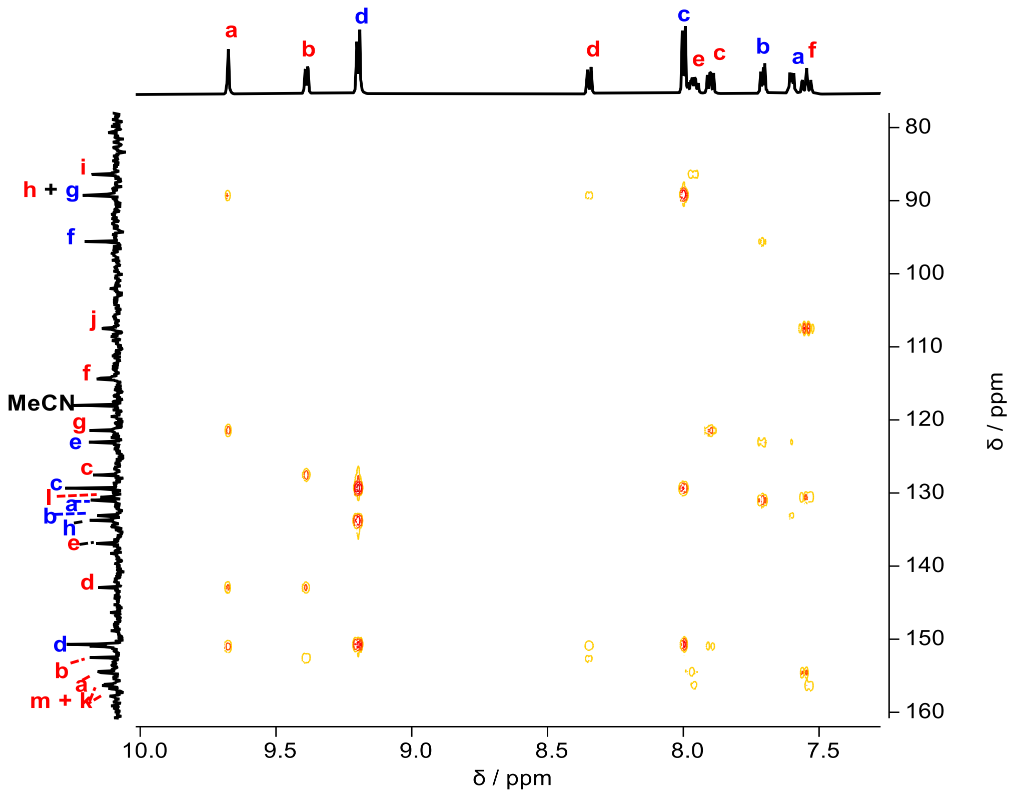

Figure S64. ^{1}H-^{13}C HMBC NMR (600 MHz, 151 MHz, 298 K, DMSO-d_6) spectrum of $[Pd_2(E\text{-}\mathbf{1})_2(\mathbf{2})_2](BF_4)_4$ ([Pd] = 2.3 mM).

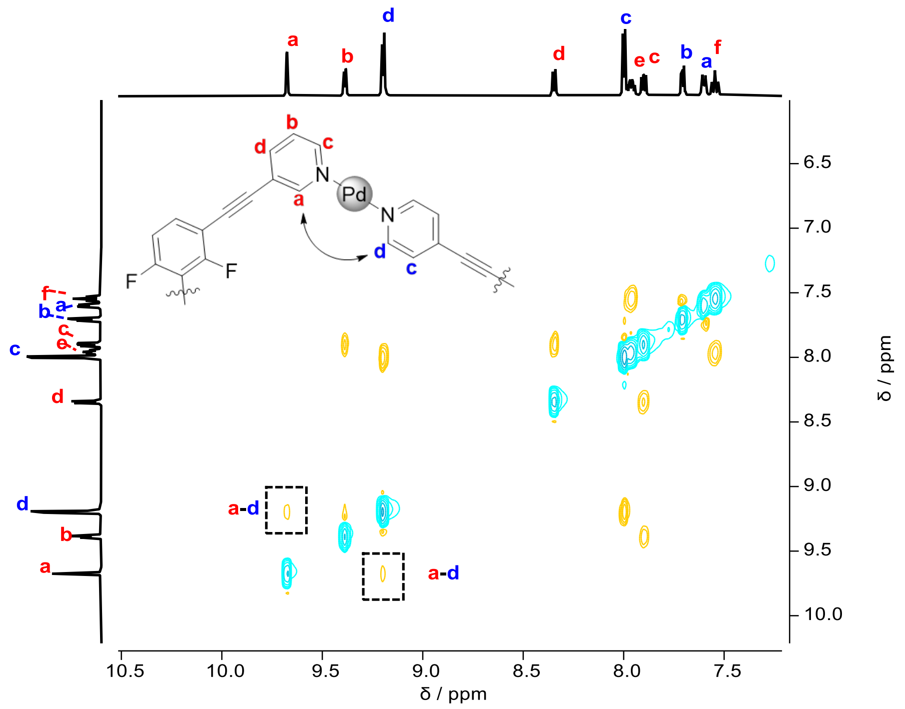

Figure S65. ^{1}H-^{1}H ROESY NMR (600 MHz, 298 K, DMSO-d_6) spectrum of $[Pd_2(E\text{-}\mathbf{1})_2(\mathbf{2})_2](BF_4)_4$ ([Pd] = 2.3 mM).

S10.3 ESI-MS spectra of $[Pd_2(E\text{-}\mathbf{1})_2(\mathbf{2})_2](BF_4)_4$ in DMSO

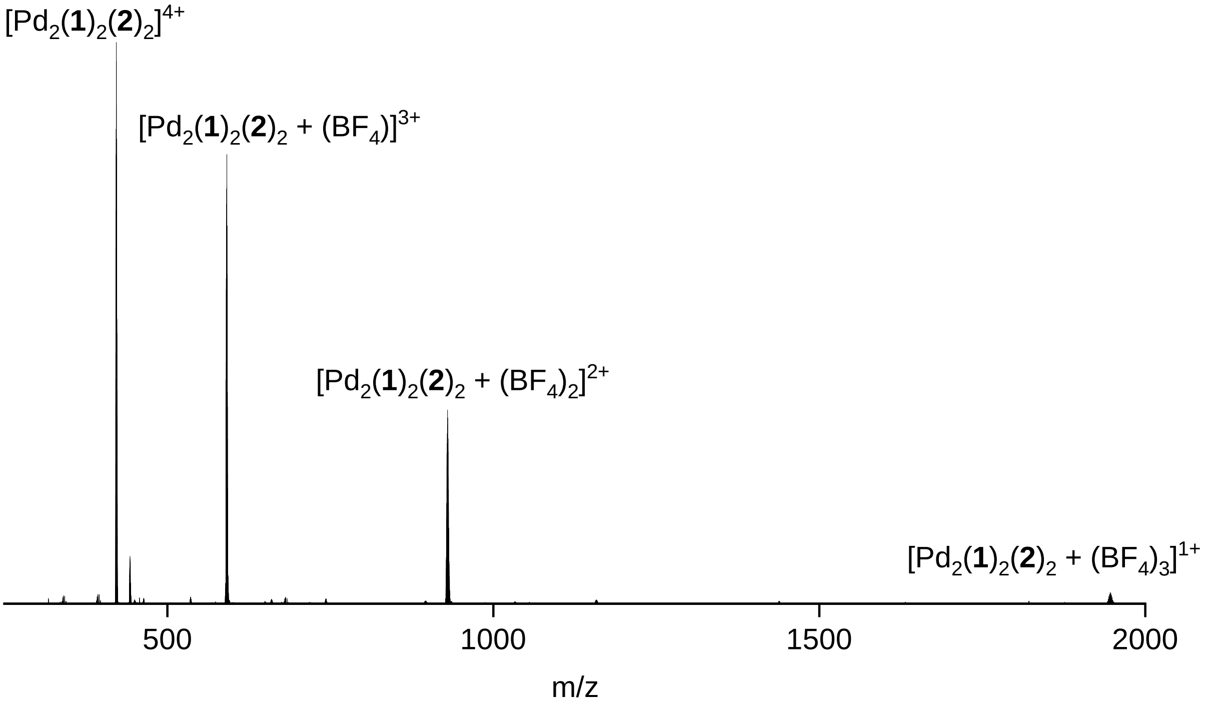

Figure S66. ESI-MS spectrum of $[Pd_2(E\text{-}\mathbf{1})_2(\mathbf{2})_2](BF_4)_4$ ([Pd] = 2.3 mM).

Table S19. Zoom scans of select ESI-MS peaks, with simulated isotope patterns ESI-MS of $[Pd_2(E\text{-}\mathbf{1})_2(\mathbf{2})_2](BF_4)_4$ ([Pd] = 2.3 mM).

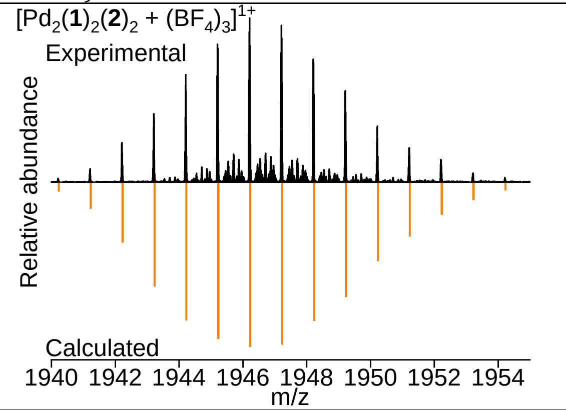	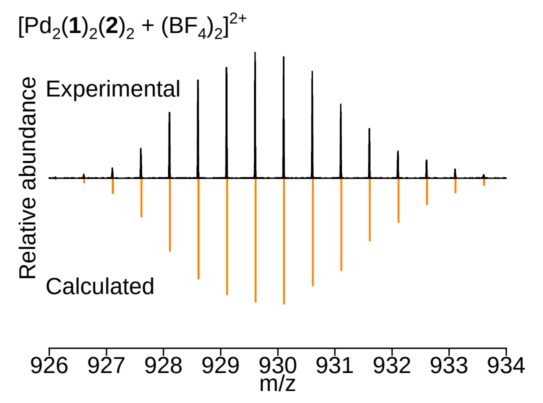
$[Pd_2(E\text{-}\mathbf{1})_2(\mathbf{2})_2 + (BF_4)_3]^{1+}$	$[Pd_2(E\text{-}\mathbf{1})_2(\mathbf{2})_2 + (BF_4)_2]^{2+}$
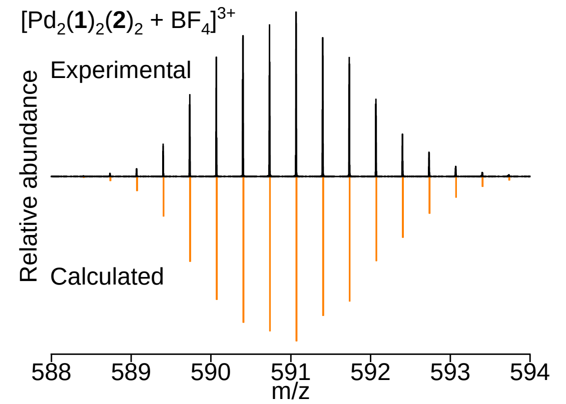	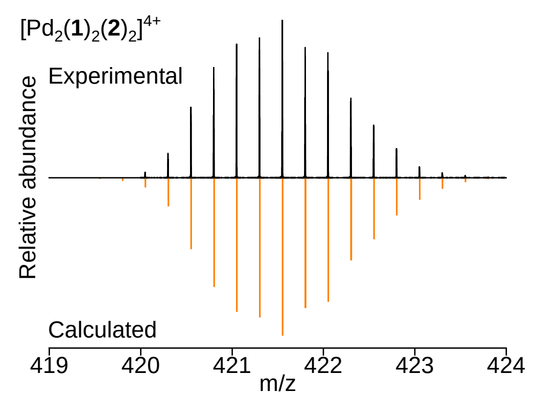
$[Pd_2(E\text{-}\mathbf{1})_2(\mathbf{2})_2 + BF_4]^{1+}$	$[Pd_2(E\text{-}\mathbf{1})_2(\mathbf{2})_2]^{4+}$

S10.4 Single crystal X-ray structure of $[Pd_2(E\text{-}\mathbf{1})_2(\mathbf{2})_2](BF_4)_4$ (CCDC: 2343886)

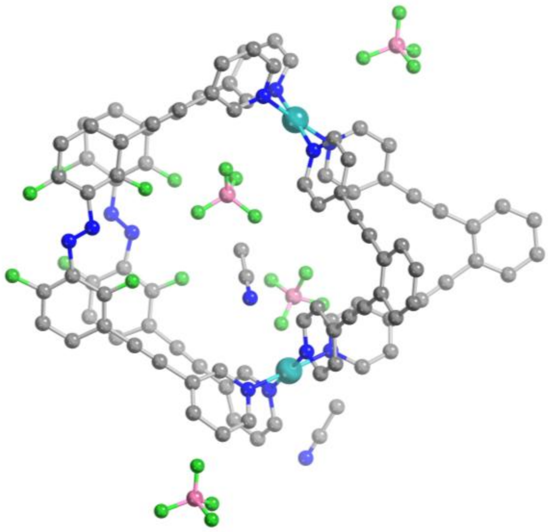

Figure S67. Single crystal X-ray structure of $[Pd_2(E\text{-}\mathbf{1})_2(\mathbf{2})_2](BF_4)_4 \cdot 2CH_3CN$. Color codes: grey: carbon; blue: nitrogen; green: fluorine; turquoise: palladium. Hydrogen atoms omitted for clarity.

Crystals suitable for X-ray diffraction were grown by the vapor diffusion of di-isopropyl ether into a solution of $[Pd_2(E\text{-}\mathbf{1})_2(\mathbf{2})_2](BF_4)_4$ in acetonitrile.

X-ray diffraction data were collected at 100 K on the MX2 Macromolecular Crystallography beamline at the Australian Synchrotron.[2] The data collection and integration were performed within the Blu-Ice[2], and XDS[3] software programs. Structure solutions were obtained by intrinsic phasing methods from SHELXT[4] and were refined by a full-matrix least-squares on all unique F^2 values using SHELXL[5] as implemented within OLEX2-1.5.[6] All non-hydrogen atoms were refined with anisotropic thermal parameters, with hydrogen atoms being added geometrically and refined using riding thermal parameters. The low quality of the crystal collected led to increased atom movement, reflected in the differences in ADPs of the carbon atoms in some phenyl rings and the acetonitrile solvents of crystallisation. Therefore, EADP was used as a constraint on some of the rings to equalise the ADPs. This movement was further reflected in the alkyne and azo linkages, which was restrained using SIMU.

PLAT084_ALERT_3_A, PLAT026_ALERT_3_B, PLAT082_ALERT_2_B, PLAT230_ALERT_3_C, PLAT230_ALERT_2_B:

- The crystal quality was low despite multiple attempts to grow crystals of better quality for SCXRD. The low quality meant that Synchrotron radiation was needed for data collection. As a result, the ratio of observed to unique reflections is quite low, and a high wR2 and R1 value was obtained.

PLAT602_ALERT_2_A, PLAT094_ALERT_2_B:

- Crystal structure of porous coordination polymers and coordination cages often are quite porous and have large solvent accessible voids in the structure. The remaining electron density scattered in the void of the cage, however despite best efforts this was unable to be solved crystallographically besides two acetonitrile solvent molecules assigned.

Table S20. Crystal data and structure refinement for $[Pd_2(E\text{-}\mathbf{1})_2(\mathbf{2})_2](BF_4)_4$.

Crystallographic details	$[Pd_2(E\text{-}\mathbf{1})_2(\mathbf{2})_2](BF_4)_4$
Formula	$C_{96}H_{54}B_4F_{24}N_{14}Pd_2$
Formula weight	2115.57
Temperature	100(2)
Crystal system	Monoclinic
Space group	$P2_1/n$
a (Å)	17.114(3)
b (Å)	37.698(8)
c (Å)	20.238(4)
α (°)	90
β (°)	113.70(3)
γ (°)	90
Volume (Å^3)	11956(5)
Z	1
ρcalc (g cm^{-3})	1.175
μ (mm^{-1})	0.381
F (000)	4224
Reflns unique	32039
No. obs [I≥2σ*I*]	11851
R_{int}	0.0727
GooF	1.294
wR_2 [I≥2σ*I*]	0.4093
R_1 [I≥2σ]	0.1625

Table S21. Angle between planes of adjacent pyridyl rings in $[Pd_2(E\text{-}\mathbf{1})_2(\mathbf{2})_2](BF_4)_4$

Angle between planes of adjacent pyridyl rings	Py-Py/°
Containing N1 and N3	16.6
Containing N2 and N4	16.9
Containing N5 and N7	21.7
Containing N6 and N8	16.5

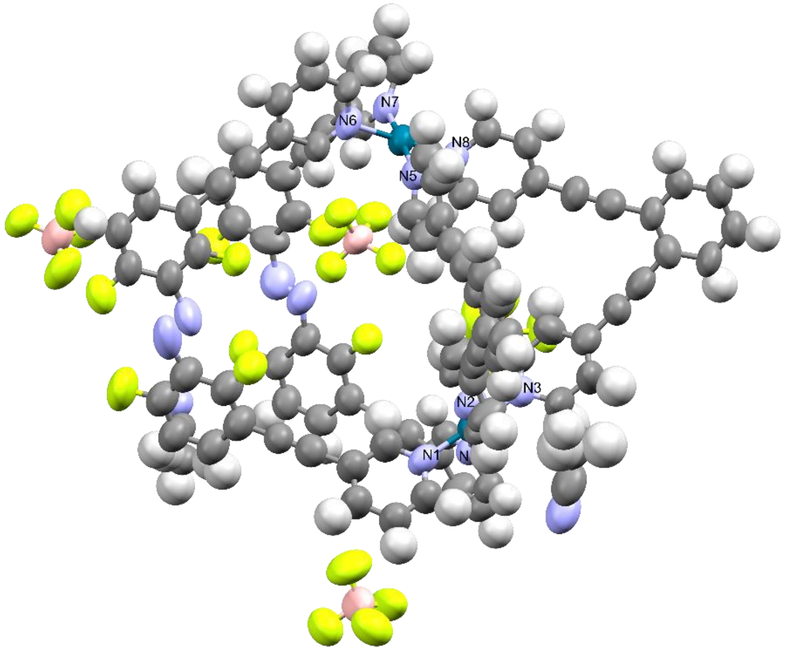

Figure S68. ORTEP representation of ligand $[Pd_2(E\text{-}\mathbf{1})_2(\mathbf{2})_2](BF_4)_4$. Ellipsoids drawn at 50% probability.

S10.5 Synthesis of $[Pd_2(E\text{-}\mathbf{1})_2(\mathbf{2})_2](BAr_F)_4$ in CD_3CN

A sample of $[Pd_2(E\text{-}\mathbf{1})_2(\mathbf{2})_2](BAr_F)_4$ was prepared by reacting a solution of $[Pd_2(E\text{-}\mathbf{1})_4](BAr_F)_4$ ([Pd] = 2.0 mM, 225 µL, 0.44 µmol, 1.0 equiv.) and free Py* with a solution of $[Pd_3(\mathbf{2})_6](BAr_F)_6$ ([Pd] = 2.0 mM, 225 µL, 0.44 µmol l, 1.0 equiv.) and free Py*. The sample was thermally equilibrated by heating at 50 °C for 20 min, affording $[Pd_2(E\text{-}\mathbf{1})_2(\mathbf{2})_2](BAr_F)_4$. ([Pd] = 2.0 mM) and free Py* (Figure S69).

The ESI-MS spectrum for $[Pd_2(E\text{-}\mathbf{1})_2(\mathbf{2})_2](BAr_F)_4$ in MeCN-d_3 corroborates the NMR data, and m/z signals for $[Pd_2(E\text{-}\mathbf{1})_2(\mathbf{2})_2 + (BAr_F)_{4-n}]^{n+}$ (n = 2-4) were observed (Figure S70).

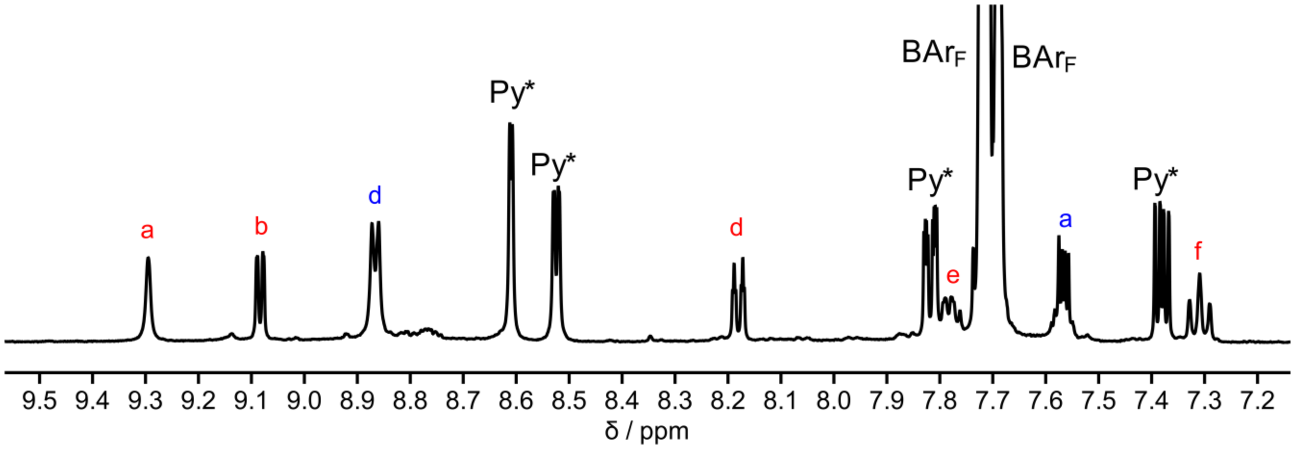

Figure S69. ^1H NMR (500 MHz, 298 K, MeCN-d_3) spectrum of $[Pd_2(E\text{-}\mathbf{1})_2(\mathbf{2})_2](BAr_F)_4$ in ([Pd] = 2.0 mM).

S10.6 ESI-MS spectra of $[Pd_2(E\text{-}\mathbf{1})_2(\mathbf{2})_2](BAr_F)_4$ in MeCN

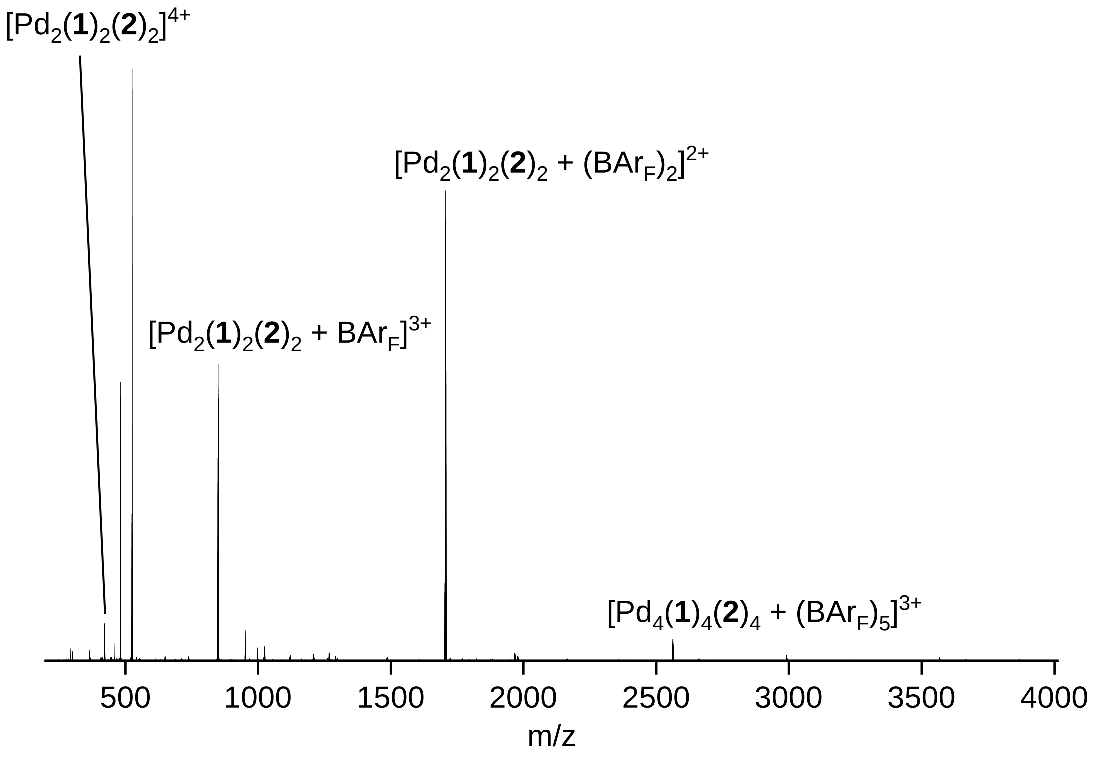

Figure S70. ESI-MS spectrum of $[Pd_2(E\text{-}\mathbf{1})_2(\mathbf{2})_2](BAr_F)_4$ ([Pd] = 2.0 mM) in MeCN.

Table S22. Zoom scans of select ESI-MS peaks, with simulated isotope patterns ESI-MS of $[Pd_2(E\text{-}\mathbf{1})_2(\mathbf{2})_2](BAr_F)_4$ ([Pd] = 2.0 mM).

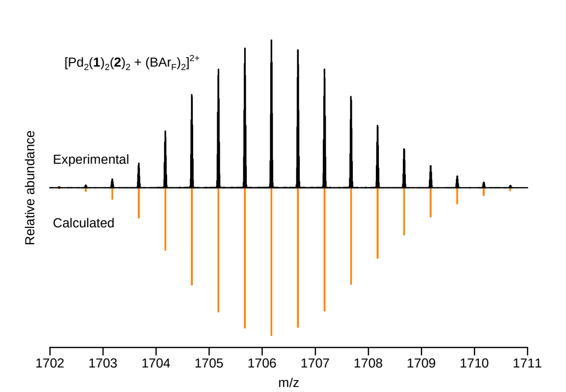	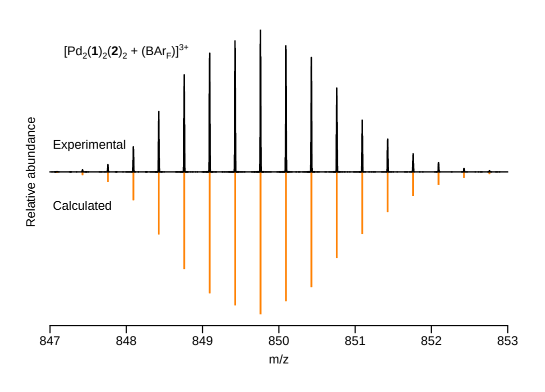
$[Pd_2(\mathbf{1})_2(\mathbf{2})_2 + (BAr_F)_2]^{2+}$	$[Pd_2(\mathbf{1})_2(\mathbf{2})_2 + (BAr_F)]^{3+}$
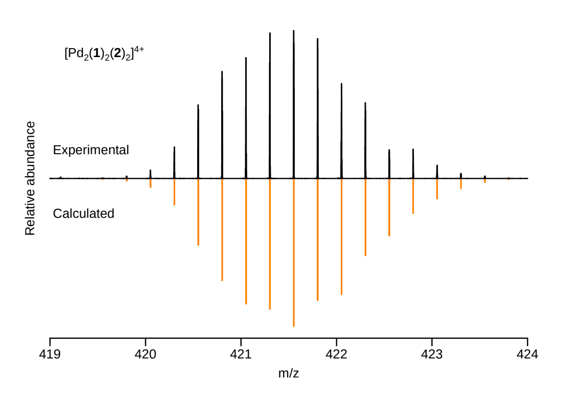	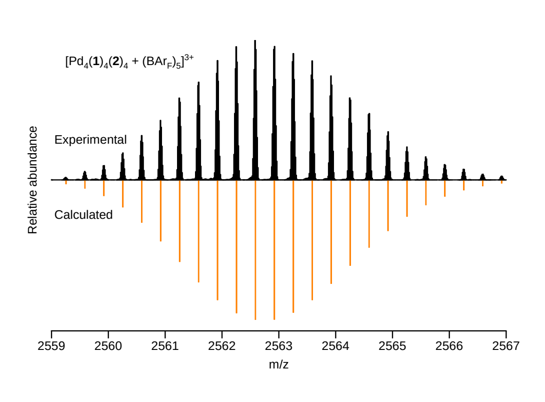
$[Pd_2(\mathbf{1})_2(\mathbf{2})_2]^{4+}$	$[Pd_4(\mathbf{1})_4(\mathbf{2})_4 + (BAr_F)_5]^{3+}$

S11. Photoswitching of $[Pd_2(E\text{-}1)_2(2)_2](BF_4)_4$

S11.1 Irradiating $[Pd_2(E\text{-}1)_2(2)_2](BF_4)_4$ with 530 nm light in DMSO-d_6

The sample of $[Pd_2(E\text{-}\mathbf{1})_2(\mathbf{2})_2](BF_4)_4$ from S10.1 was irradiated with a 530 nm LED for 10 min. This resulted in the disassembly of $[Pd_2(E\text{-}\mathbf{1})_2(\mathbf{2})_2](BF_4)_4$ and the formation of a mixture of self-assembled species. NMR spectroscopy and ESI-MS indicate that homoleptic structures $[Pd(Z\text{-}\mathbf{1})_2](BF_4)_2$ and $[Pd_4(\mathbf{2})_8](BF_4)_8$ are formed, in addition to other minor species. To identify these additional self-assembled species, ^1H (Figure S71) and ^{19}F (Figure S72) NMR spectra of $[Pd_2(E\text{-}\mathbf{1})_2(\mathbf{2})_2](BF_4)_4$ before and after irradiating with 530 nm light were compared to the NMR spectra of the other samples of self-assembled species: $[Pd_2(E\text{-}\mathbf{1})_4](BF_4)_4$, $[Pd(Z\text{-}\mathbf{1})_2](BF_4)_2$, $[Pd_4(\mathbf{2})_8](BF_4)_8$, and $[Pd_2(E\text{-}\mathbf{1})_2(\mathbf{2})_2](BF_4)_4$.

Irradiating $[Pd_2(E\text{-}\mathbf{1})_2(\mathbf{2})_2](BF_4)_4$ with 530 nm light does not result in the complete disassembly of $[Pd_2(E\text{-}\mathbf{1})_2(\mathbf{2})_2](BF_4)_4$ (see Supporting information S11.3 for more details). By comparing the ^1H NMR spectra, it appears that homoleptic species $[Pd_4(\mathbf{2})_8](BF_4)_8$ and $[Pd(Z\text{-}\mathbf{1})_2](BF_4)_2$ are generated, which is corroborated by ESI-MS data (see Supporting information S11.4). However, $[Pd(Z\text{-}\mathbf{1})_2](BF_4)_2$ is not observed in the ^{19}F NMR spectrum after irradiation.

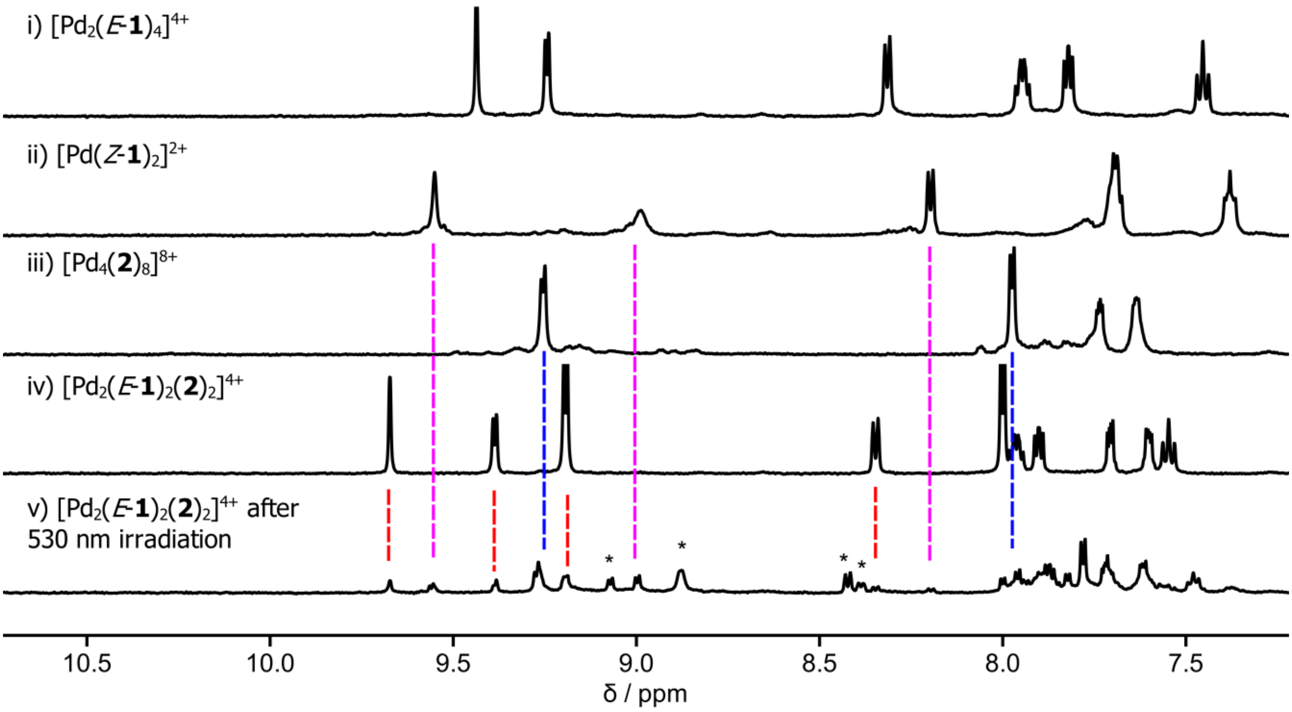

Figure S71. ^1H NMR (600 MHz, 298 K, DMSO-d_6) spectra of i) $[Pd_2(E\text{-}\mathbf{1})_4](BF_4)_4$, ii) $[Pd(Z\text{-}\mathbf{1})_2](BF_4)_2$, iii) $[Pd_4(\mathbf{2})_8](BF_4)_8$, iv) $[Pd_2(E\text{-}\mathbf{1})_2(\mathbf{2})_2](BF_4)_4$, and v) $[Pd_2(E\text{-}\mathbf{1})_2(\mathbf{2})_2](BF_4)_4$ after irradiating with a 530 nm LED for 10 min. For all samples [Pd] = 2.3 mM. New unidentified species are labeled with '*'.

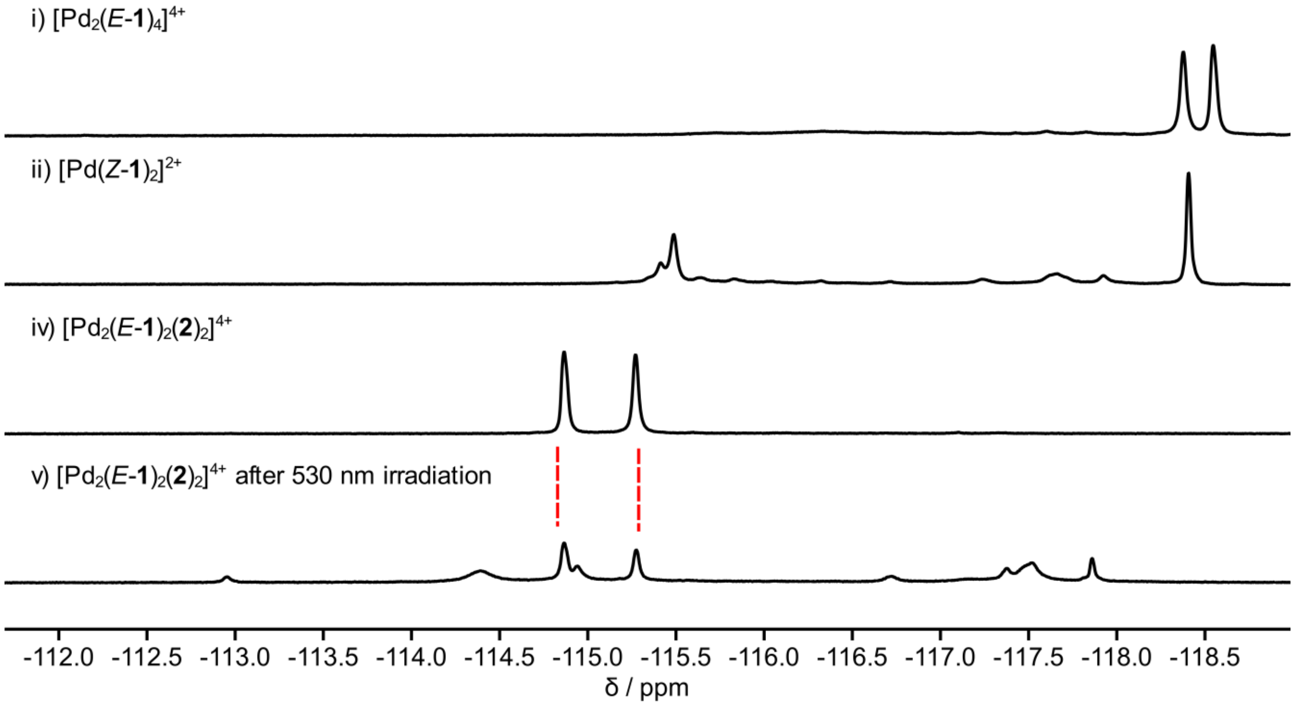

Figure S72. ^{19}F NMR (565 MHz, 298 K, DMSO-d_6) spectra of i) $[Pd_2(E\text{-}\mathbf{1})_4](BF_4)_4$, ii) $[Pd(Z\text{-}\mathbf{1})_2](BF_4)_2$, iii) $[Pd_4(\mathbf{2})_8](BF_4)_8$, iv) $[Pd_2(E\text{-}\mathbf{1})_2(\mathbf{2})_2](BF_4)_4$, and v) $[Pd_2(E\text{-}\mathbf{1})_2(\mathbf{2})_2](BF_4)_4$ after irradiating with a 530 nm LED for 10 min. For all samples [Pd] = 2.3 mM.

S11.2 2D NMR spectra of the heteroleptic mixture in DMSO-d_6 after irradiation with 530 nm light

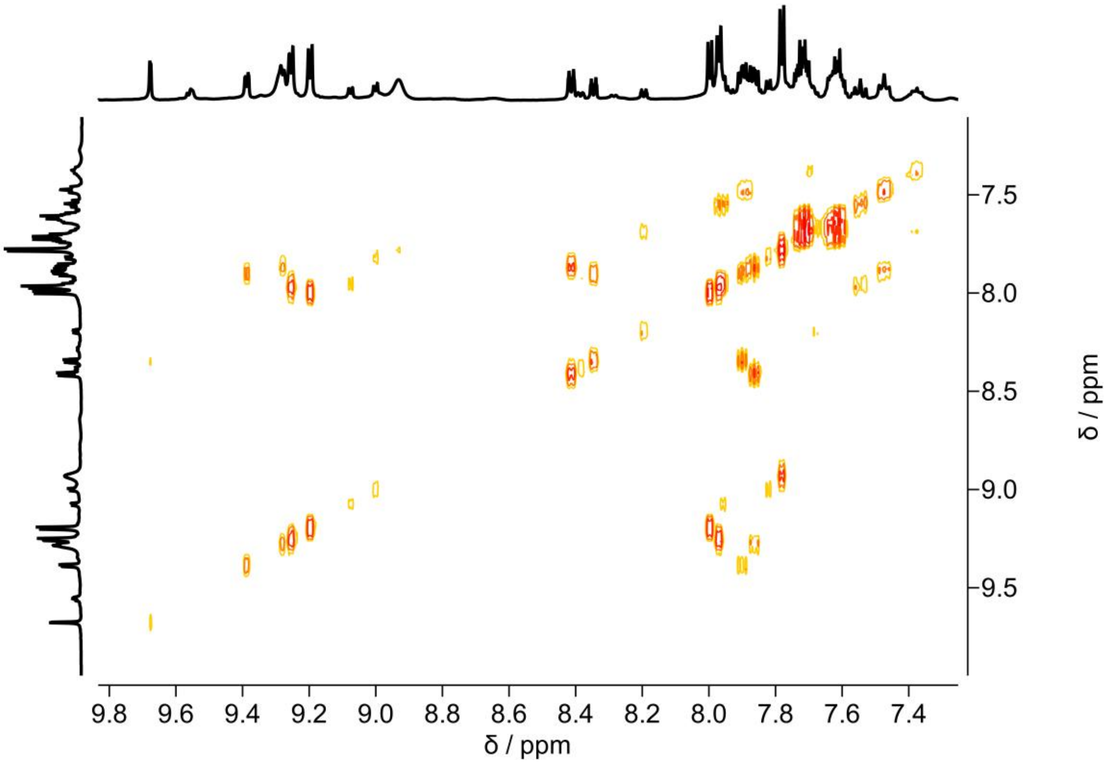

Figure S73. ^{1}H-^{1}H COSY NMR (600 MHz, 298 K, DMSO-d_6) spectrum of $[Pd_2(E\text{-}\mathbf{1})_2(\mathbf{2})_2](BF_4)_4$ ([Pd] = 2.3 mM) after irradiation with a 530 nm LED for 10 min.

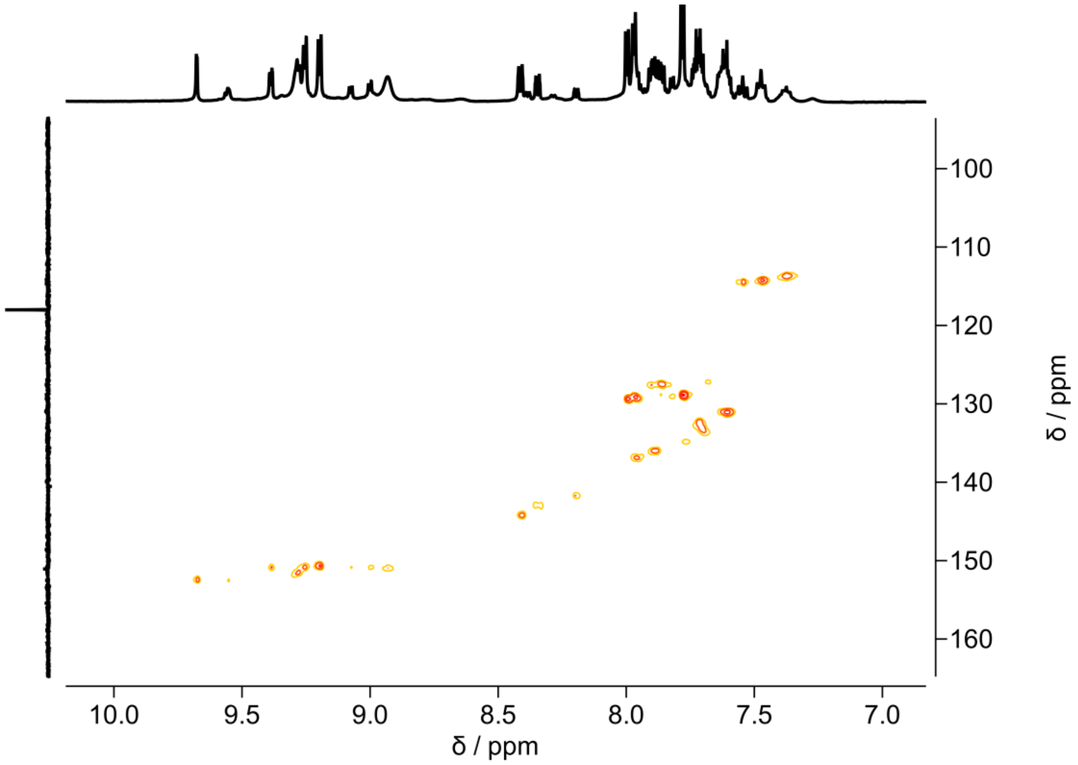

Figure S74. ^{1}H-^{13}C HSQC NMR (600 MHz, 151 MHz, 298 K, DMSO-d_6) spectrum of $[Pd_2(E\text{-}\mathbf{1})_2(\mathbf{2})_2](BF_4)_4$ ([Pd] = 2.3 mM) after irradiation with a 530 nm LED for 10 min.

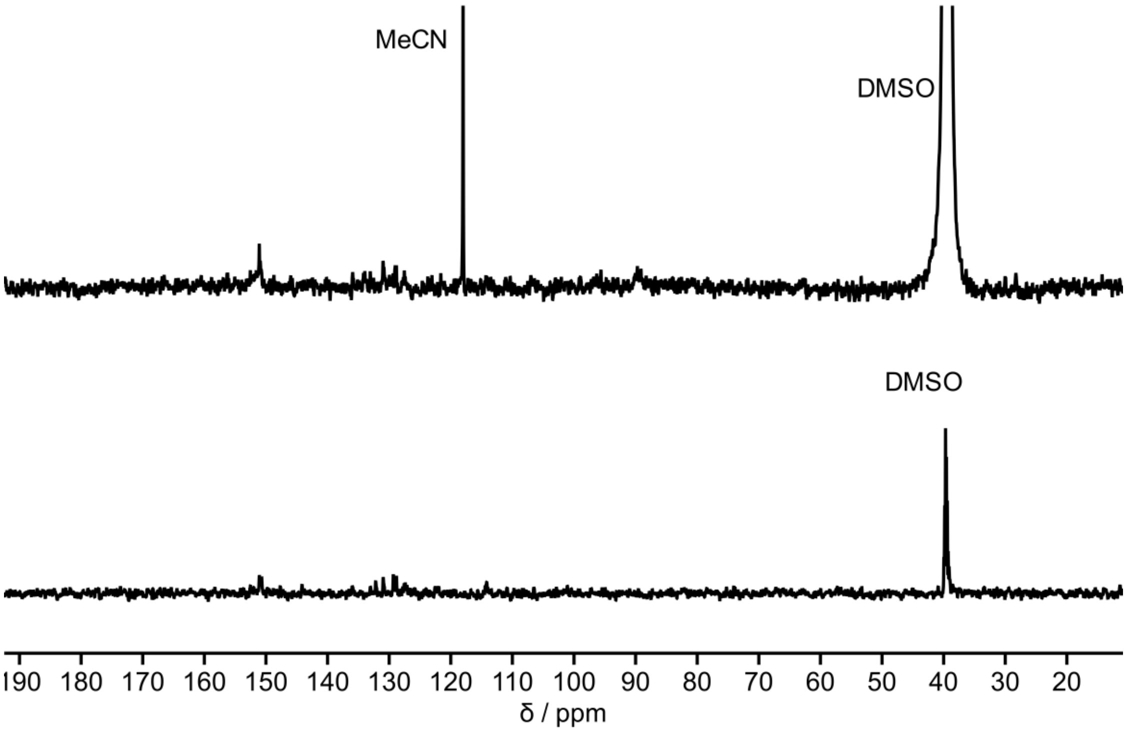

Figure S75. $^{13}C\{^{1}H\}$ (top, ns = 3200) and DEPT-135 (bottom, ns = 1600) NMR (151 MHz, 298 K, DMSO-d_6) spectra of $[Pd_2(E\text{-}\mathbf{1})_2(\mathbf{2})_2](BF_4)_4$ ([Pd] = 2.3 mM) after irradiation with a 530 nm LED for 10 min.

S11.3 Measuring PSS distribution for $[Pd_2(E\text{-}1)_2(2)_2](BF_4)_4$ in DMSO-d_6 after irradiation with 530 nm light

A sample of $[Pd_2(E\text{-}\mathbf{1})_2(\mathbf{2})_2](BF_4)_4$ ([Pd] = 2.1 mM, 500 µL in DMSO-d_6, 1.1 µmol) was prepared by reacting $[Pd_2(E\text{-}\mathbf{1})_4](BF_4)_4$ ([Pd] = 2.1 mM, 250 µL in DMSO-d_6, 0.53 µmol, 1.0 equiv., with $[Pd_4(\mathbf{2})_8](BF_4)_8$ ([Pd] = 2.1 mM, 250 µL in DMSO-d_6, 0.53 µmol, 1.0 equiv.). The sample was prepared in the dark and heated with a heat gun to 150 °C for 5 minutes to thermally equilibrate the sample, quantitatively affording $[Pd_2(E\text{-}\mathbf{1})_2(\mathbf{2})_2](BF_4)_4$.

^{1}H (Figure S76) and ^{19}F NMR (Figure S77) spectroscopy were used to monitor how irradiation affects $[Pd_2(E\text{-}\mathbf{1})_2(\mathbf{2})_2](BF_4)_4$. The sample of $[Pd_2(E\text{-}\mathbf{1})_2(\mathbf{2})_2](BF_4)_4$ (Figure S76i, Figure S77i) was irradiated with 530 nm light for 10 min (Figure S76ii, Figure S77ii) which resulted in the formation of a mixture of self-assembled species. The sample was then irradiated with 405 nm light for 5 min (Figure S76iii, Figure S77iii), which regenerated $[Pd_2(E\text{-}\mathbf{1})_2(\mathbf{2})_2](BF_4)_4$ and demonstrates that $[Pd_2(E\text{-}\mathbf{1})_2(\mathbf{2})_2](BF_4)_4$ can be reversibly disassembled and reassembled with visible light. The sample was irradiated again with 530 nm light for 10 min (Figure S76iv, Figure S77iv) and 4-dimethylaminopyridine (DMAP, 164 mM, 128 µL in DMSO-d_6, 21.0 µmol, 20.0 equiv.) was added to the sample (Figure S76v, Figure S77v). This afforded a solution containing free ligand **1**, free ligand **2**, free DMAP, and $[Pd(DMAP)_4](BF_4)_2$. The ^{1}H NMR spectrum does not have any cleanly resolved signals for the two isomers, so we used ^{19}F NMR to quantify the PSS.

We measured the ^{19}F NMR signal intensities of *E*-**1** and *Z*-**1** after DMAP was added to calculate the distribution of the *E*/*Z* isomers of **1**. Irradiating $[Pd_2(E\text{-}\mathbf{1})_2(\mathbf{2})_2](BF_4)_4$ generated a PSS containing 88% *Z*-**1** and 12% *E*-**1**, which is the same PSS distribution for free ligand **1** when irradiated with 530nm light (S3.1). This indicates that the PSS selectivity of ligand **1** is not affected when assembled in $[Pd_2(E\text{-}\mathbf{1})_2(\mathbf{2})_2](BF_4)_4$, unlike in our previously published example.[9]

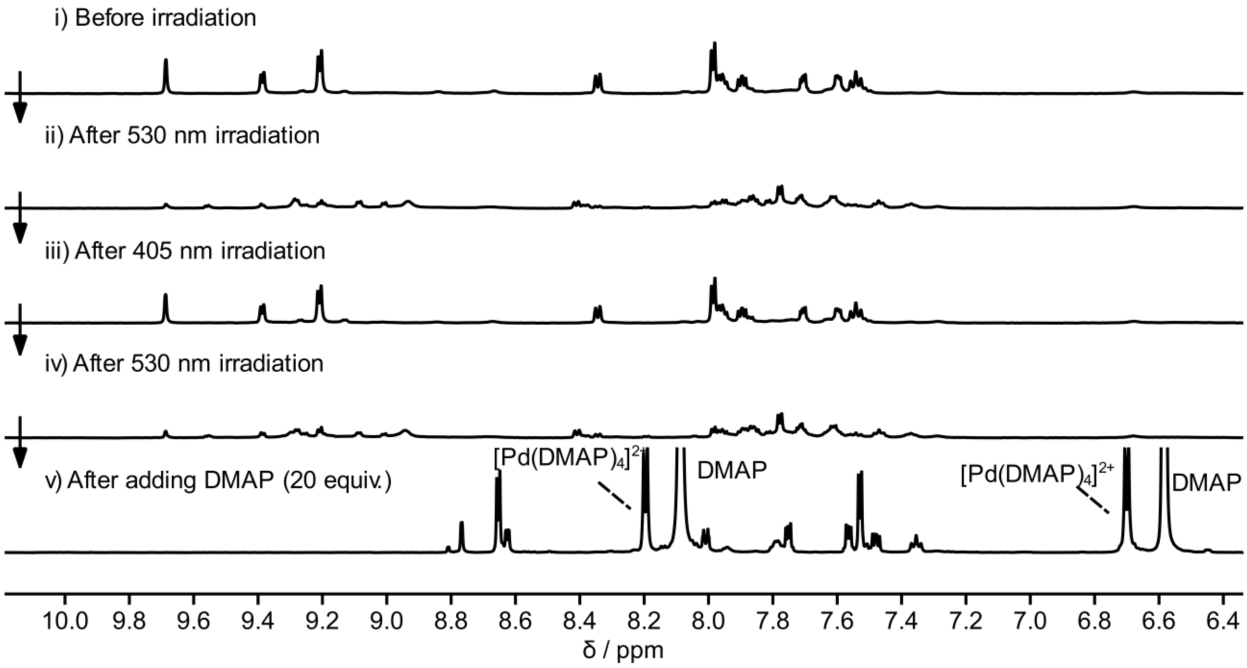

Figure S76. ^{1}H NMR (600 MHz, 298 K, DMSO-d_6) spectra of a sample of $[Pd_2(E\text{-}\mathbf{1})_2(\mathbf{2})_2](BF_4)_4$ ([Pd] = 2.1 mM, 1 equiv.) i) before irradiation, ii) after irradiating with a 530 nm LED for 10 min, iii) after irradiating with a 405 nm LED for 5 min, iv) after irradiating again with a 530 nm LED for 10 min, and v) after adding DMAP (42 mM, 20 equiv.).

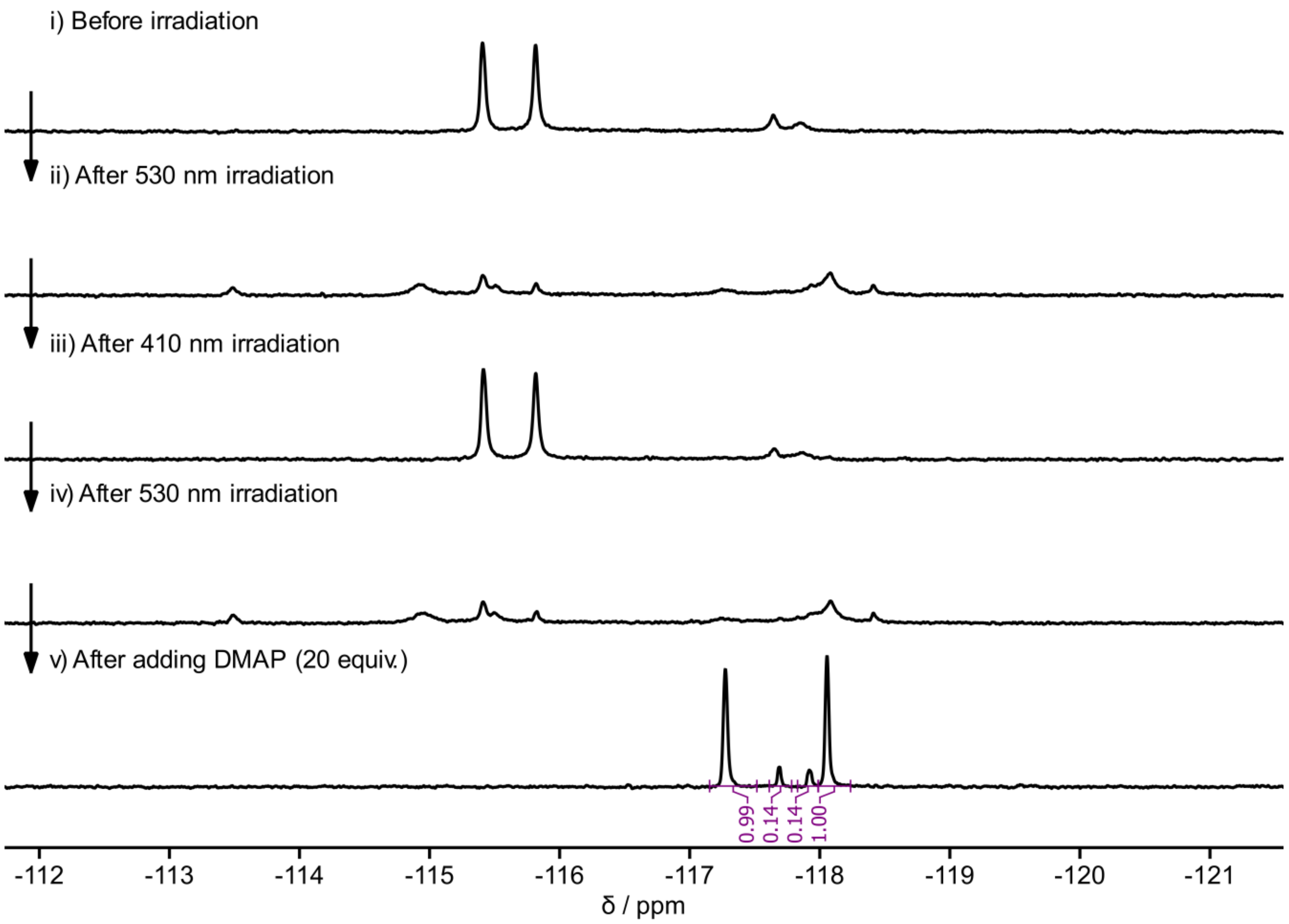

Figure S77. ^{19}F NMR (565 MHz, 298 K, DMSO-d_6) spectra of a sample of $[Pd_2(E\text{-}\mathbf{1})_2(\mathbf{2})_2](BF_4)_4$ ([Pd] = 2.1 mM, 1 equiv.) i) before irradiation, ii) after irradiating with a 530 nm LED for 10 min, iii) after irradiating with a 405 nm LED for 5 min, iv) after irradiating again with a 530 nm LED for 10 min, and v) after adding DMAP (42 mM, 20 equiv.).

S11.4 ESI-MS spectra of $[Pd_2(E\text{-}\mathbf{1})_2(\mathbf{2})_2](BF_4)_4$ in DMSO before and after irradiation with 530 nm light

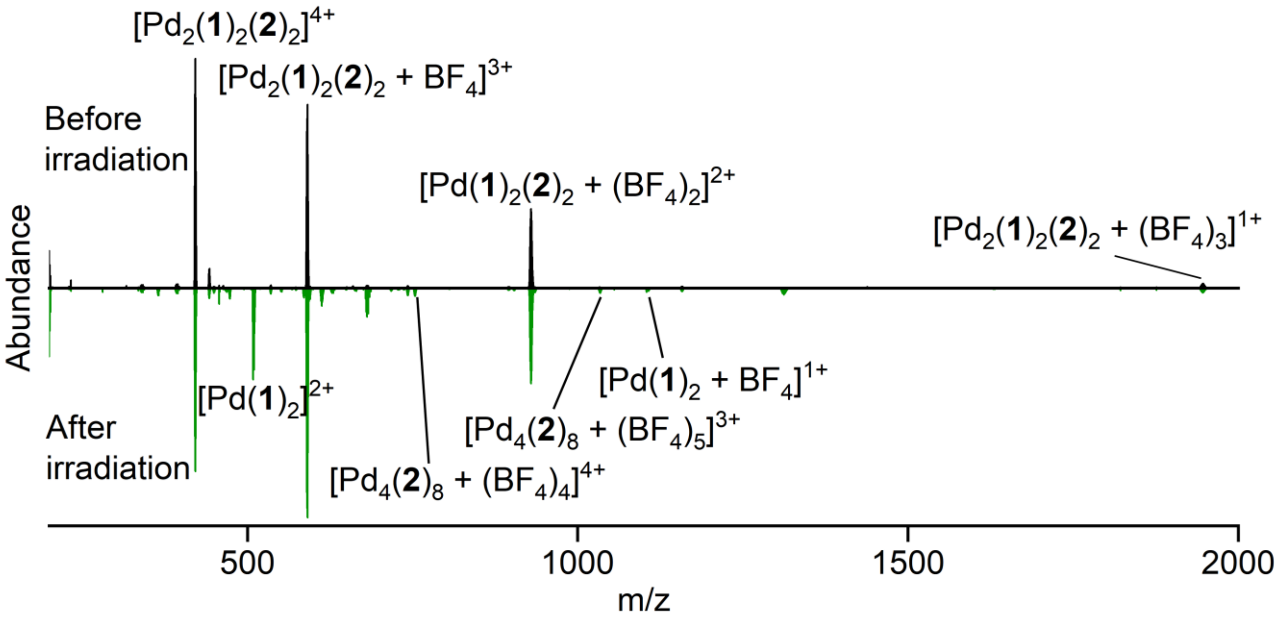

Figure S78. ESI-MS spectrum of $[Pd_2(E\text{-}\mathbf{1})_2(\mathbf{2})_2](BF_4)_4$ ([Pd] = 2.3 mM) before irradiation and after irradiation with a 530 nm LED for 10 min.

Table S23. Select zoom scans of select ESI-MS peaks before and after irradiating $[Pd_2(E\text{-}\mathbf{1})_2(\mathbf{2})_2](BAr_F)_4$ ([Pd] = 2.1 mM) with 530 nm light for 10 min.

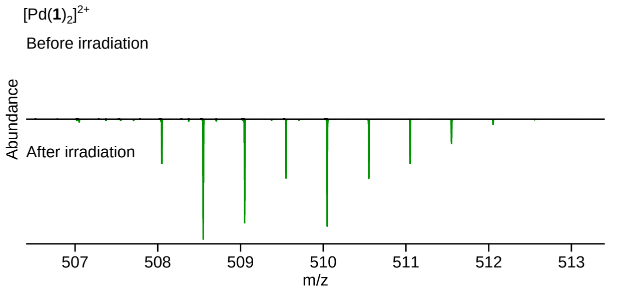	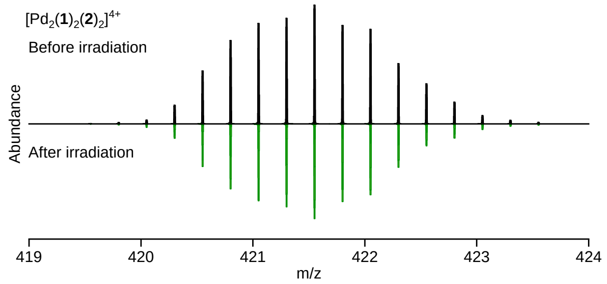
After irradiation: $[Pd(\mathbf{1})_2]^{2+}$	$[Pd_2(\mathbf{1})_2(\mathbf{2})_2]^{4+}$
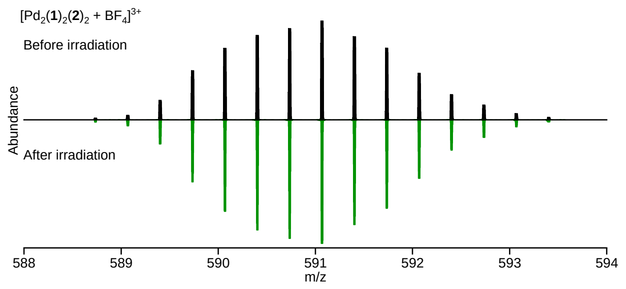	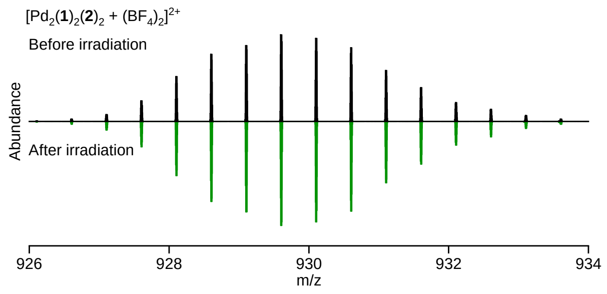
$[Pd_2(\mathbf{1})_2(\mathbf{2})_2 + BF_4]^{3+}$	$[Pd_2(\mathbf{1})_2(\mathbf{2})_2 + (BF_4)_2]^{2+}$

S11.5 ESI-MS spectra of $[Pd_2(E\text{-}\mathbf{1})_2(\mathbf{2})_2](BAr_F)_4$ in MeCN before and after irradiation with 530 nm light

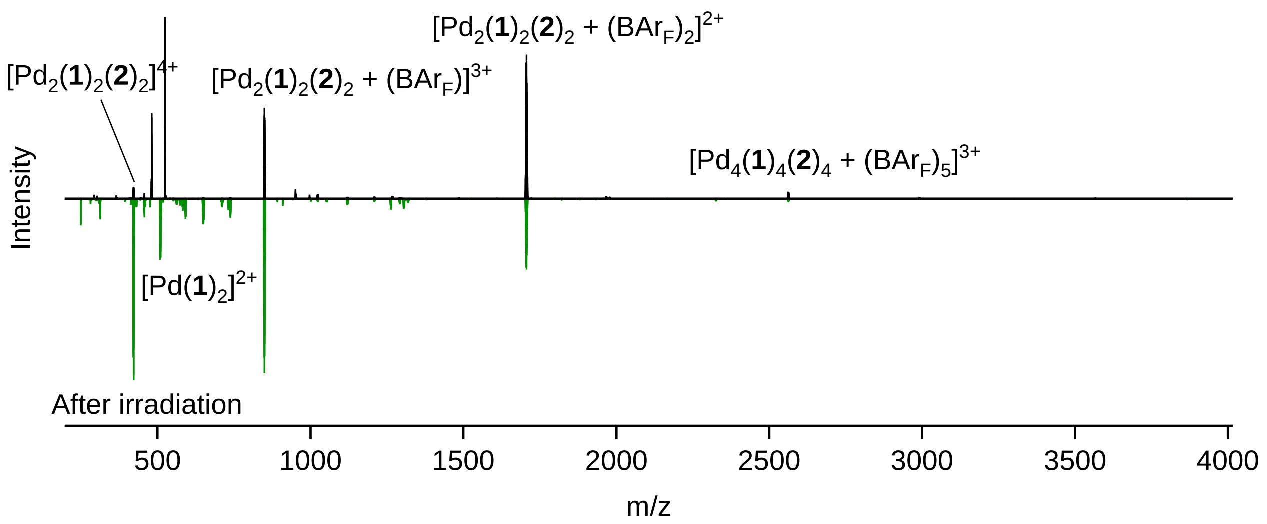

Figure S79. ESI-MS spectrum of $[Pd_2(E\text{-}\mathbf{1})_2(\mathbf{2})_2](BAr_F)_4$ ([Pd] = 2.1 mM) in MeCN-d_3 before irradiation and after irradiation with a 530 nm LED for 10 min.

Table S24. Select zoom scans of select ESI-MS peaks before and after irradiating $[Pd_2(E\text{-}\mathbf{1})_2(\mathbf{2})_2](BAr_F)_4$ ([Pd] = 2.1 mM) in MeCN-d_3 with 530 nm light for 10 min.

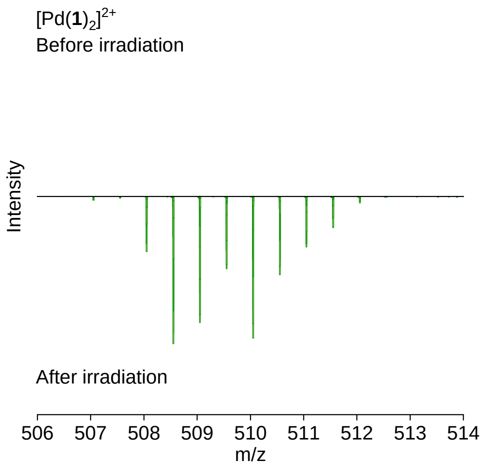	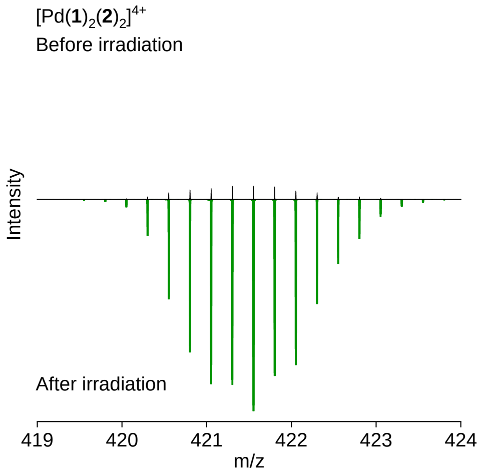
After irradiation: $[Pd(\mathbf{1})_2]^{2+}$	$[Pd_2(\mathbf{1})_2(\mathbf{2})_2]^{4+}$
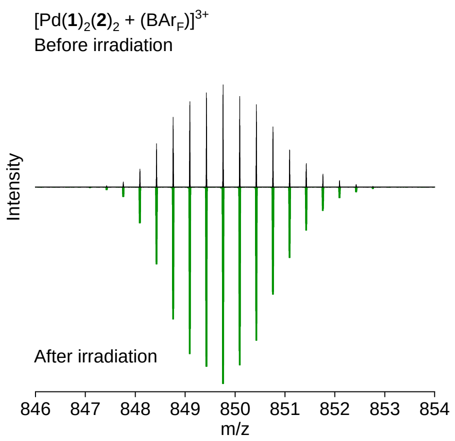	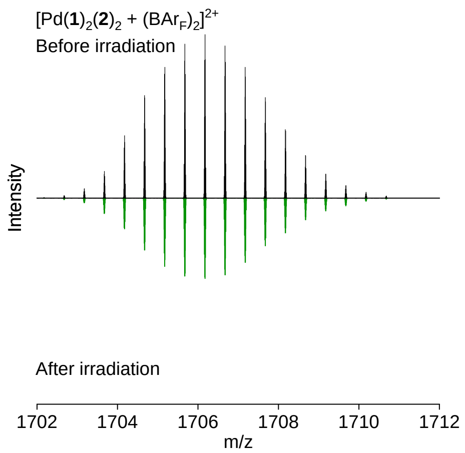
$[Pd_2(\mathbf{1})_2(\mathbf{2})_2 + (BAr_F)]^{3+}$	$[Pd_2(\mathbf{1})_2(\mathbf{2})_2 + (BAr_F)_2]^{2+}$

S12. Spartan models of $[Pd_2(E\text{-}\mathbf{1})_4]^{4+}$, $[Pd_2(E\text{-}1)_2(\mathbf{2})_2]^{4+}$ and $[Pd(Z\text{-}1)_2]^{2+}$

We used Spartan Student v8, Version 8.0.6 to create a plausible model for $[Pd_2(E\text{-}\mathbf{1})_4]^{4+}$ using simple molecular mechanics only. The *cis*-N-Pd-N bond angles were all restricted to 90 degrees and *trans*-N-Pd-N are restricted to 180 degrees as these angles are rarely deviate from ideal. The torsion angles of the C-N=N-C on each side of the azo bonds were restricted to enforce co-planarity of the rings on each side, similar to that observed in the X-ray structure of *E*-**1**. The model has a twisted helicate structure similar to that of the related cage,[9] and suggests a similar twisted structure could be formed with $[Pd_2(E\text{-}\mathbf{1})_4]^{4+}$. A similar model of the heteroleptic cage for $[Pd_2(E\text{-}\mathbf{1})_2(\mathbf{2})_2]^{4+,}$ with the same restrictions applied (no restrictions to ligand **2**), has a structure similar to that of the X-ray structure of $[Pd_2(E\text{-}\mathbf{1})_2(\mathbf{2})_2]^{4+}$, suggesting these simple models could be reasonable approximations for the structures of these complexes.

We also made a similar model of $[Pd(Z\text{-}\mathbf{1})_2]^{2+}$ where the N-Pd-N bonds are restricted as above, and the C-N=N-C torsion angles are restricted to 0 degrees to force the ligand to be in the *Z* configuration. This model suggests the *cis*-$[Pd(Z\text{-}\mathbf{1})_2]^{2+}$ configuration at the metal center is also reasonable.

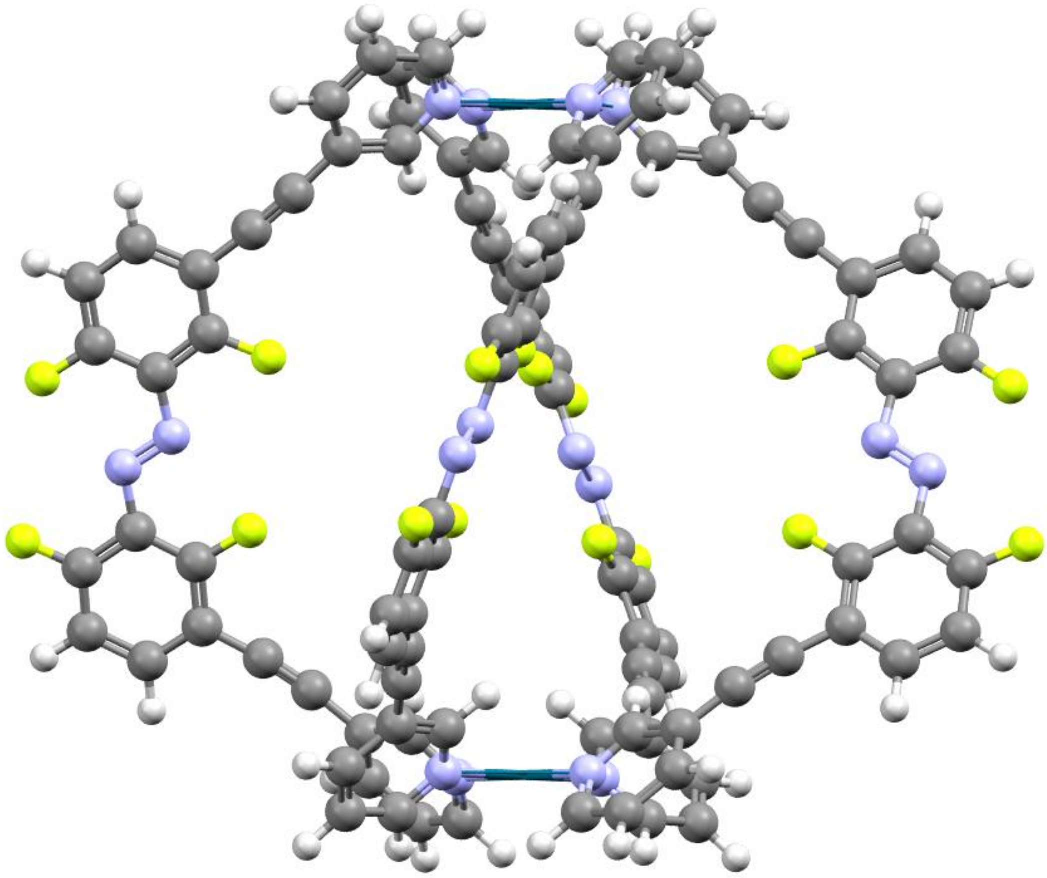

Figure S80. Simple molecular mechanics model of $[Pd_2(E\text{-}\mathbf{1})_4]^{4+}$.

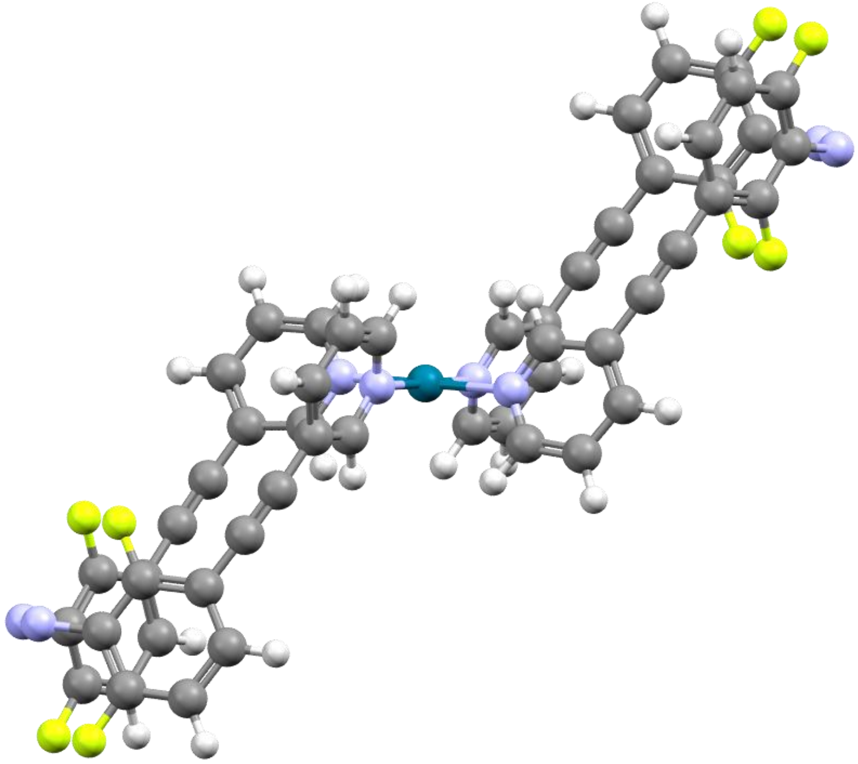

Figure S81. Simple molecular mechanics model of *cis*-[Pd(*Z*-**1**)$_2$]$^{2+}$.

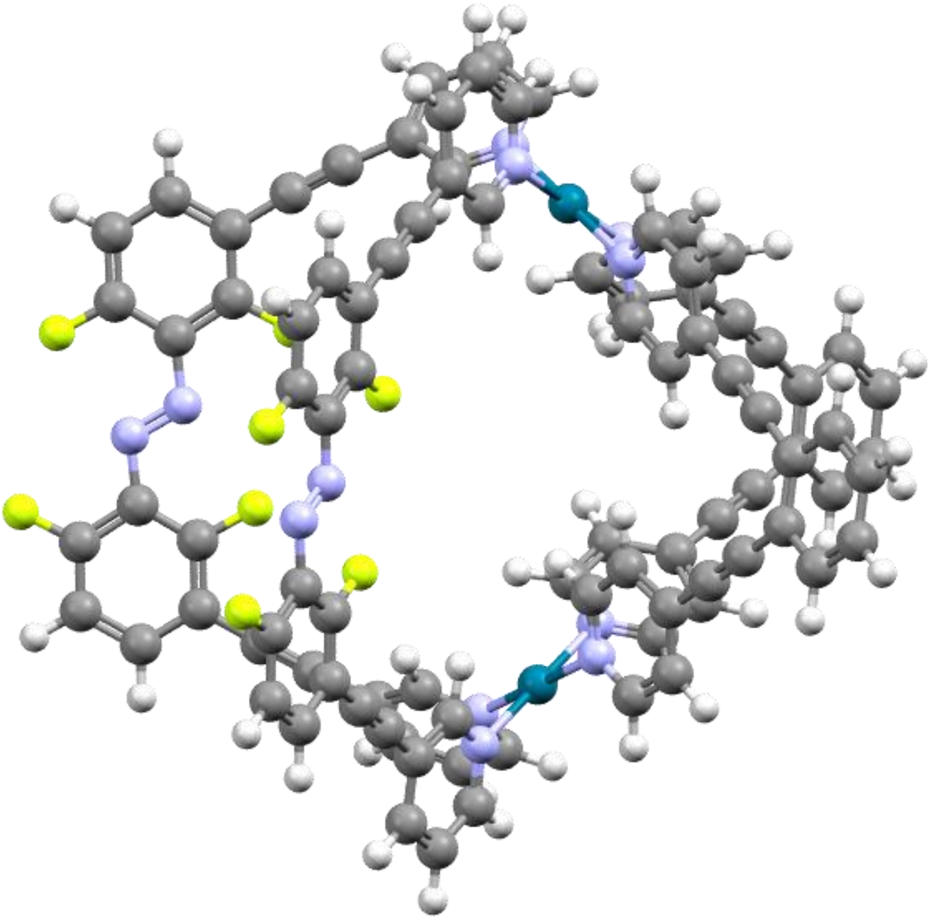

Figure S82. Simple molecular mechanics model of [Pd(*E*-**1**)$_2$(**2**)]$^{4+}$.

S13. Diffusion NMR data for $[Pd_2(E\text{-}1)_4](BF_4)_4$, $[Pd_4(2)_8](BF_4)_8$ and $[Pd_2(E\text{-}1)_2(2)_2](BF_4)_4$ in DMSO-d_6

The gradient strength used for diffusion NMR experiments is dependent on the geometry of the sample, among other things, and needs to be calibrated using the known diffusion coefficient of the residual protons in D_2O at 298 K (1.93×10^{-9} m^2 s^{-1}).[14] The calibration was performed after data collection (prior to analysis) by correcting the nominal *b* values by the resulting calibration factor of 0.928.

The Bruker Avance III 400 MHz instrument with a Prodigy CryoProbe used the diff5 command with the Bruker diffSte pulsed-gradient stimulated echo (PGSTE) pulse sequence, with gradient length δ and echo delay Δ as specified in each experiment, including a spoiler gradient (followed by a 1.5 s recovery) to destroy magnetization remaining from the gradient pulse. Sine-shaped gradient pulses were used, typically ranging from 2-51 G cm^{-1}.

Using the standard Stejskal–Tanner equation:[15]

$$I = I_0 \exp\left[-D\gamma^2 g^2 \delta^2 \left(\Delta - \frac{1}{3}\delta\right)\right] = I_0 \exp\left[-Db\right]$$

where I_0 is the NMR signal intensity in the absence of the applied gradient, γ is the gyromagnetic ratio of the nucleus, g and δ describe the amplitude and duration of the magnetic field gradient pulse, respectively, and the delay Δ defines the timescale over which diffusion is measured.

Estimates of D and I_0 are determined by nonlinear regression of Stejskal–Tanner equation onto the signal integral data.

Samples of the homoleptic switchable cage $[Pd_2(E\text{-}\mathbf{1})_4](BF_4)_4$, homoleptic non-switchable cage $[Pd_4(\mathbf{2})_8](BF_4)_8$ and heteroleptic cage $[Pd_2(E\text{-}\mathbf{1})_2(\mathbf{2})_2](BF_4)_4$, all with the same concentration and volume (2.1 mM, 500 uL) were prepared.

S13.1 Gradient calibration

This will be the diffusion of water (or respectively HDO, values depending on the used NMR machine). A sample of D_2O was used for the gradient calibration with 3 different combinations of δ and Δ:

Table S25. Values of Δ and δ used for gradient calibration of HDO in D_2O.

Experiment number	δ / ms	Δ / ms	Apparent *D* / 10^{-9} m^{2}s^{-1}
1	1.0	78.7	2.05
2	1.5	35.3	2.06
3	2.5	13.4	2.12
Average			2.07±0.04

From this data, the correction factor for the gradient is 0.928, which is applied to all subsequent measurements.

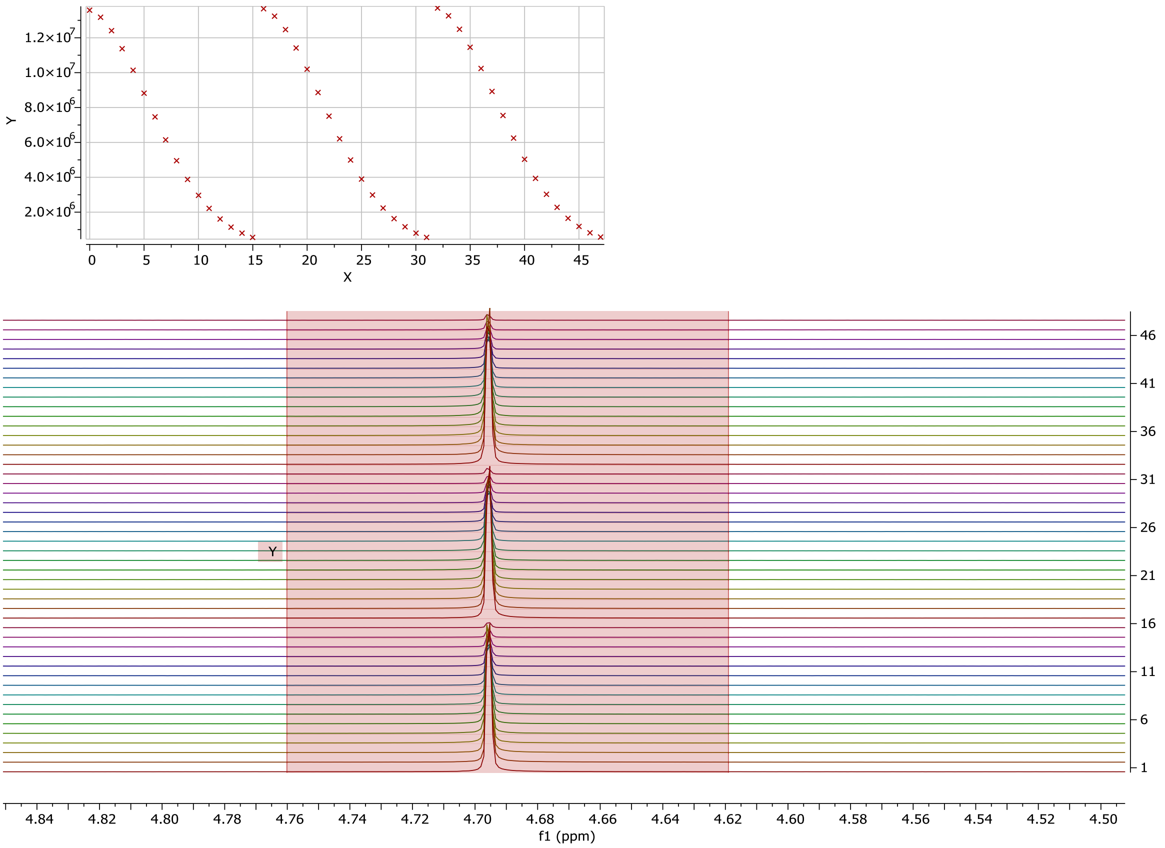

Figure S83. NMR spectra of the DOSY measurement of HDO in D_2O.

The normalised signal intensities were then plotted against the *b* values (from the DiffList file) and fit in OriginPro to the Stejskal–Tanner equation to give apparent *D* values which were used to calibrate the gradient strength.

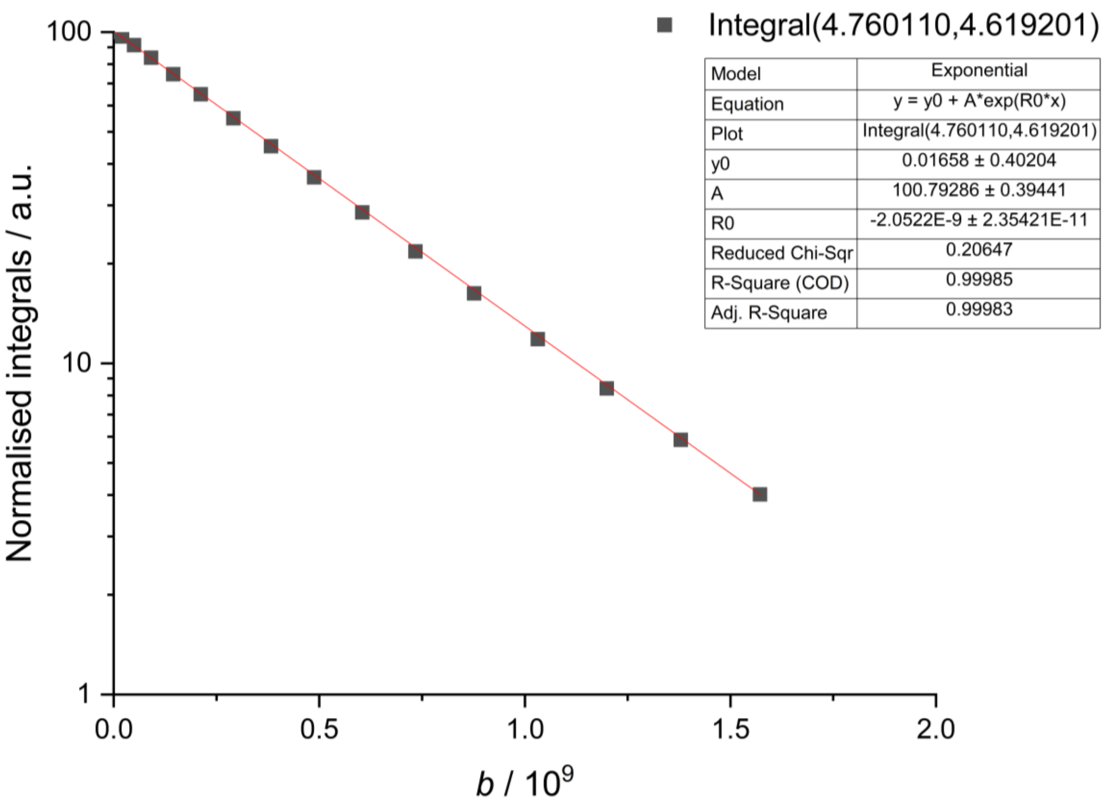

Figure S84. Representative normalized signal intensities of HDO versus *b* values for δ = 1.0 ms, Δ = 78.7 ms.

S13.2 Diffusion coefficient of the homoleptic switchable cage $[Pd_2(E\text{-}\mathbf{1})_4](BF_4)_4$ in DMSO-d_6

A sample of the homoleptic cage $[Pd_2(E\text{-}\mathbf{1})_4](BF_4)_4$ in DMSO-d_6 ([Pd] = 2.1 mM) was measured using the following Δ and δ values:

Table S26. Used Δ and δ values to determine the diffusion coefficient of $[Pd_2(E\text{-}\mathbf{1})_4](BF_4)_4$.

Experiment number	δ / ms	Δ / ms	D / 10^{-10} m^{2}s^{-1}
1	3.0	137.0	1.08
2	4.0	78.0	1.01
3	5.0	50.7	1.05
Average			1.05 ±0.04

Using the Stokes-Einstein equation, this corresponds to a hydrodynamic radius of 10.5 Å.

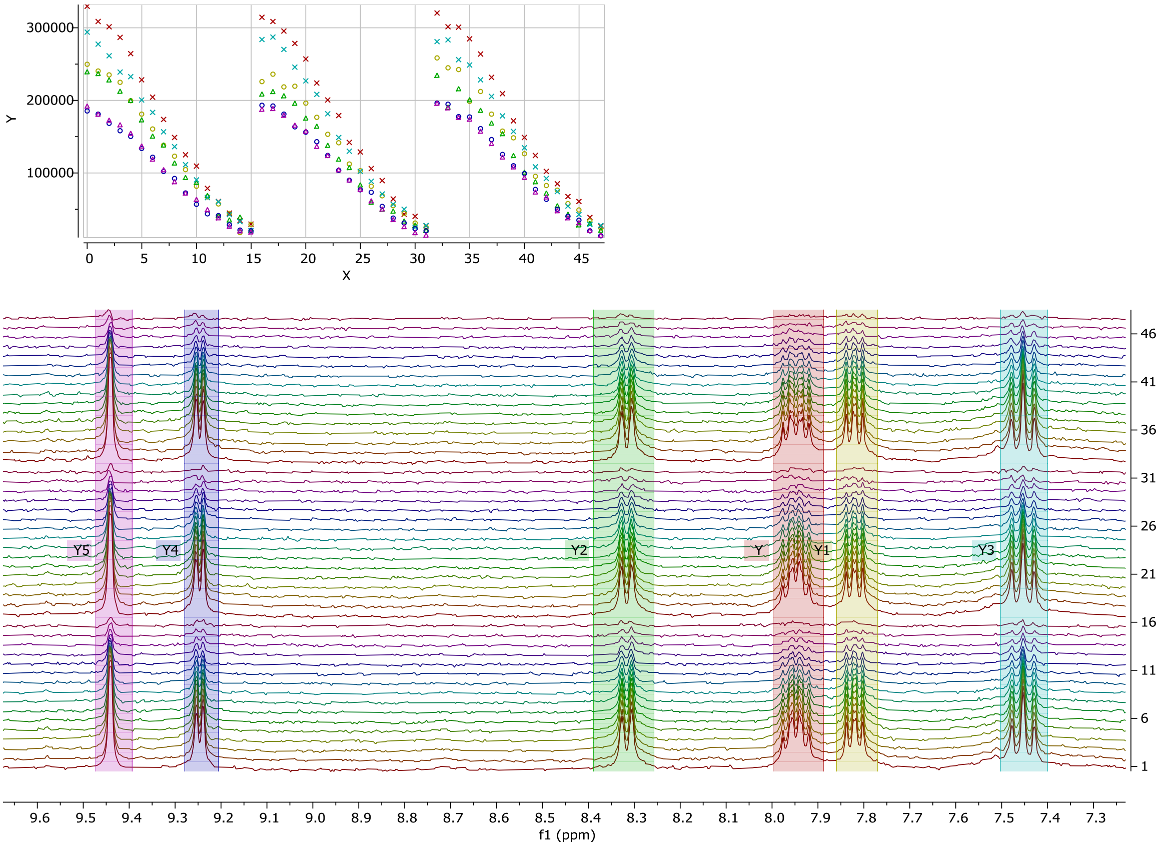

Figure S85. Diffusion NMR spectra for $[Pd_2(E\text{-}\mathbf{1})_4](BF_4)_4$ in DMSO-d_6.

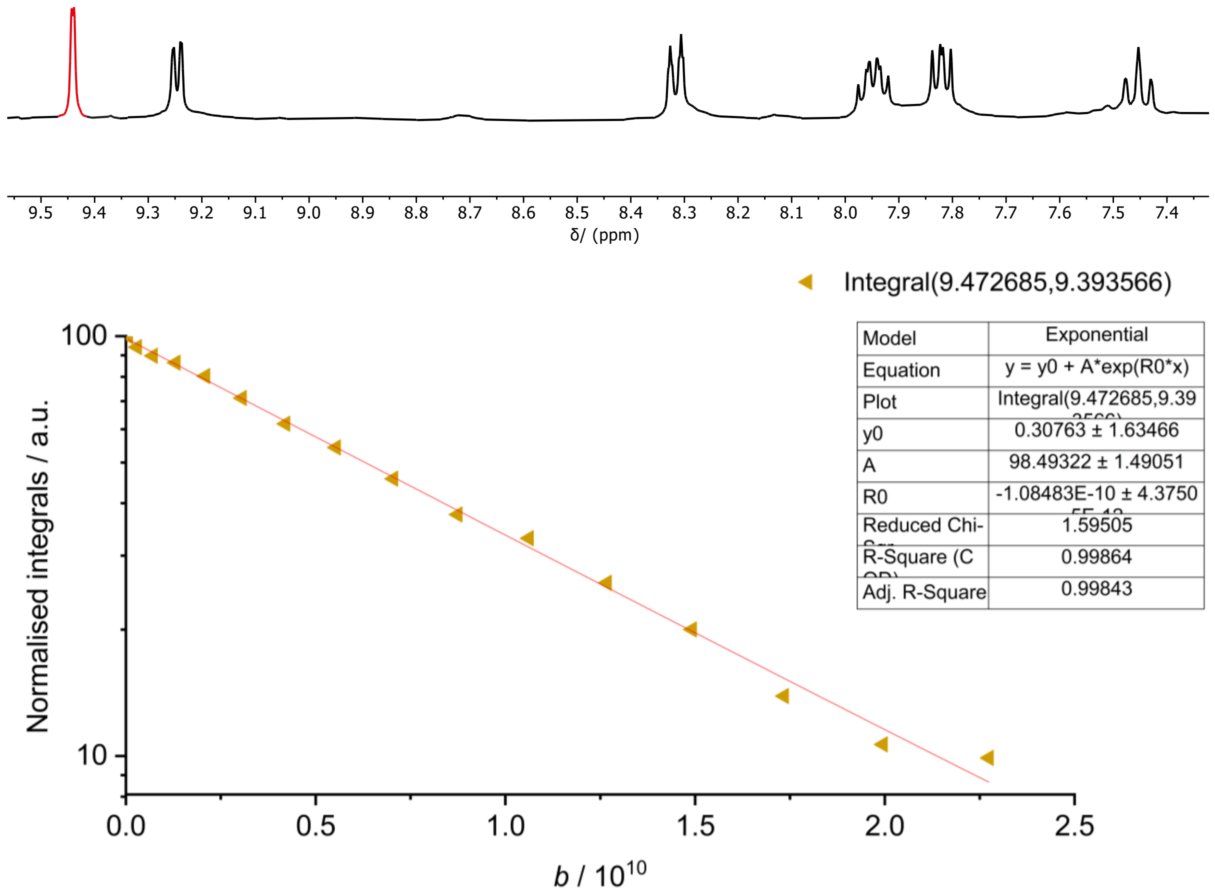

Figure S86. Representative diffusion NMR fitting for the signal at 9.47 ppm of $[Pd_2(E\text{-}\mathbf{1})_4](BF_4)_4$ in DMSO-d_6. Fitting of integrals of $[Pd_2(E\text{-}\mathbf{1})_4](BF_4)_4$ from ^{1}H NMR diffusion experiment: ste, δ = 3.0 ms, Δ = 137.0 ms. Data were fit to a monoexponential using corrected gradient values ($G_{corrected} = G_{app} \times 0.928$).

S13.3 Diffusion coefficient of the heteroleptic cage $[Pd_2(E\text{-}1)_2(2)_2](BF_4)_4$ in DMSO-d_6

A sample of the heteroleptic cage $[Pd_2(E\text{-}\mathbf{1})_2(\mathbf{2})_2](BF_4)_4$ in DMSO-d_6 ([Pd] = 2.1 mM) was measured using the following Δ and δ values:

Table S27. Used Δ and δ values to determine the diffusion coefficient of $[Pd_2(E\text{-}\mathbf{1})_2(\mathbf{2})_2](BF_4)_4$.

Experiment number	δ / ms	Δ / ms	D / 10^{-10} m^2s^{-1}
1	4.0	109.6	1.22
2	5.0	71.0	1.22
3	6.0	50.1	1.18
Average			1.21 ± 0.03

Using the Stokes-Einstein equation, this corresponds to a hydrodynamic radius of 9.1 Å.

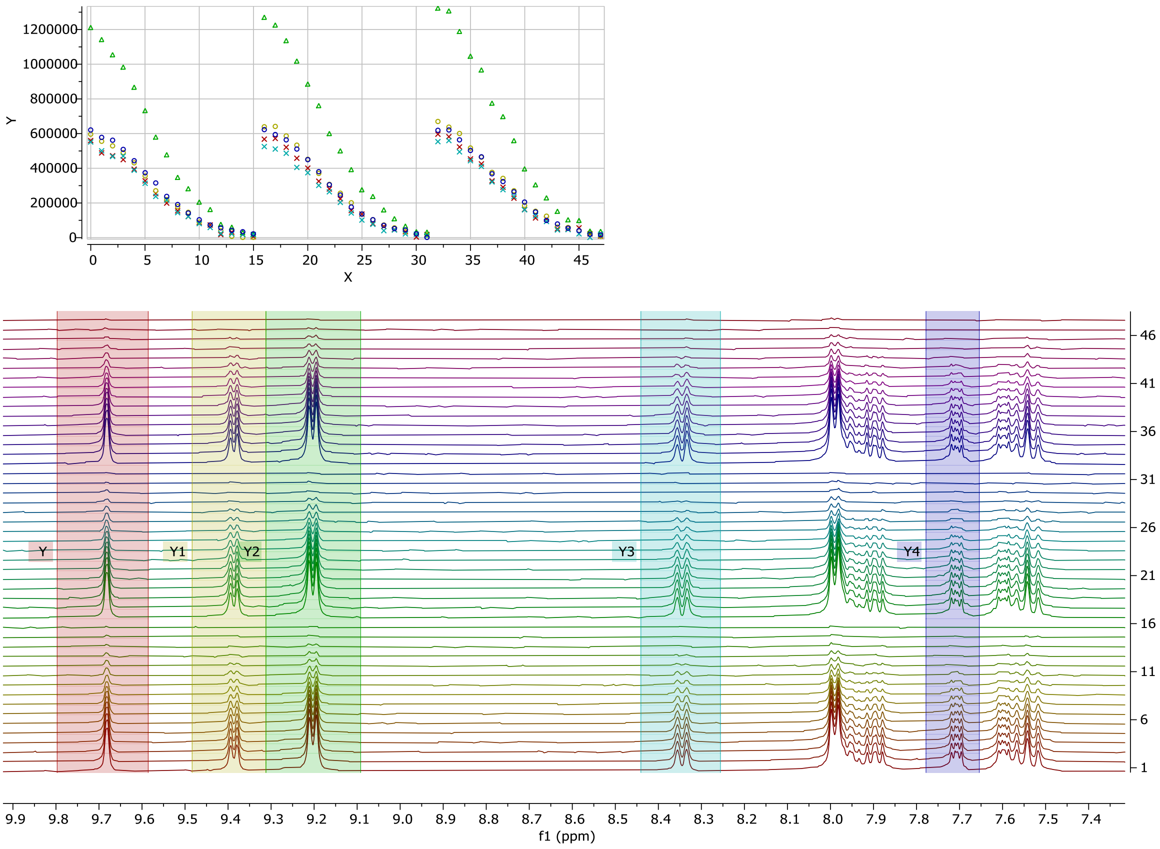

Figure S87. NMR spectra of the diffusion measurement of $[Pd_2(E\text{-}\mathbf{1})_2(\mathbf{2})_2](BF_4)_4$.

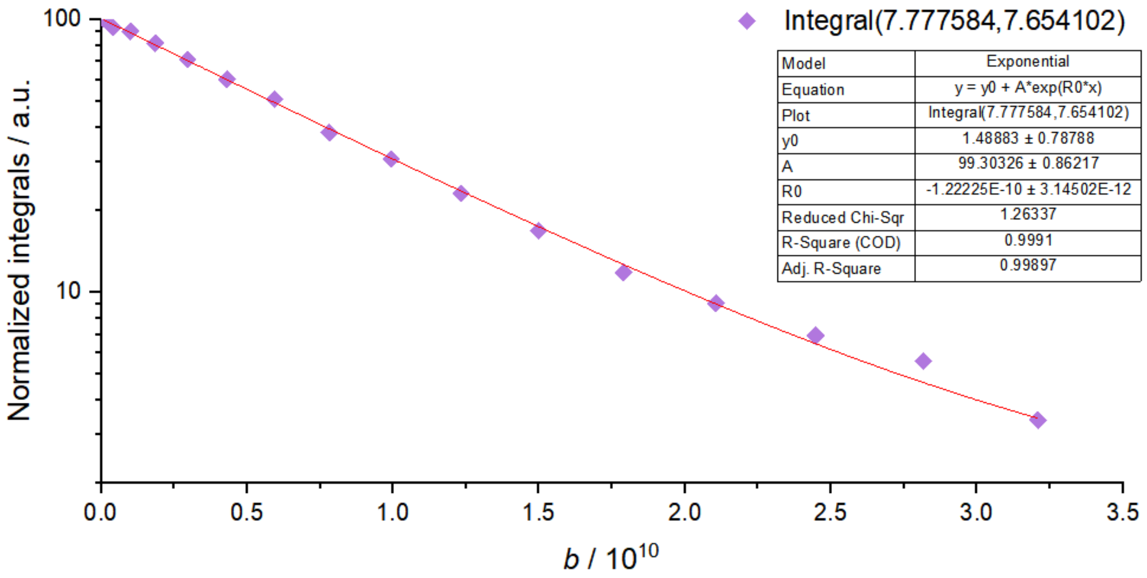

Figure S88 Fitting of integrals of $[Pd_2(E\text{-}\mathbf{1})_2(\mathbf{2})_2](BF_4)_4$ from ^{1}H NMR diffusion experiment a) ste, δ = 4.0 ms, Δ = 109.6 ms,. Data were fit to a monoexponential using corrected gradient values ($G_{corrected} = G_{app} \times 0.928$).

S13.4 Diffusion coefficient of the homoleptic cage $[Pd_4(2)_8](BF_4)_4$ in DMSO-d_6

A sample of the homoleptic cage $[Pd_4(2)_8](BF_4)_8$ in DMSO-d_6 ([Pd] = 2.1 mM) was measured using the following Δ and δ values:

Table S28. Used Δ and δ values to determine the diffusion coefficient of $[Pd_4(\mathbf{2})_8](BF_4)_8$.

Experiment number	δ / ms	Δ / ms	D / 10^{-10} m^2s^{-1}
1	4.0	107.0	1.19
2	5.0	69.0	1.11
3	7.0	37.0	1.00
Average			1.1 ±0.1

Using the Stokes-Einstein equation, this corresponds to a hydrodynamic radius of 9.9 Å.

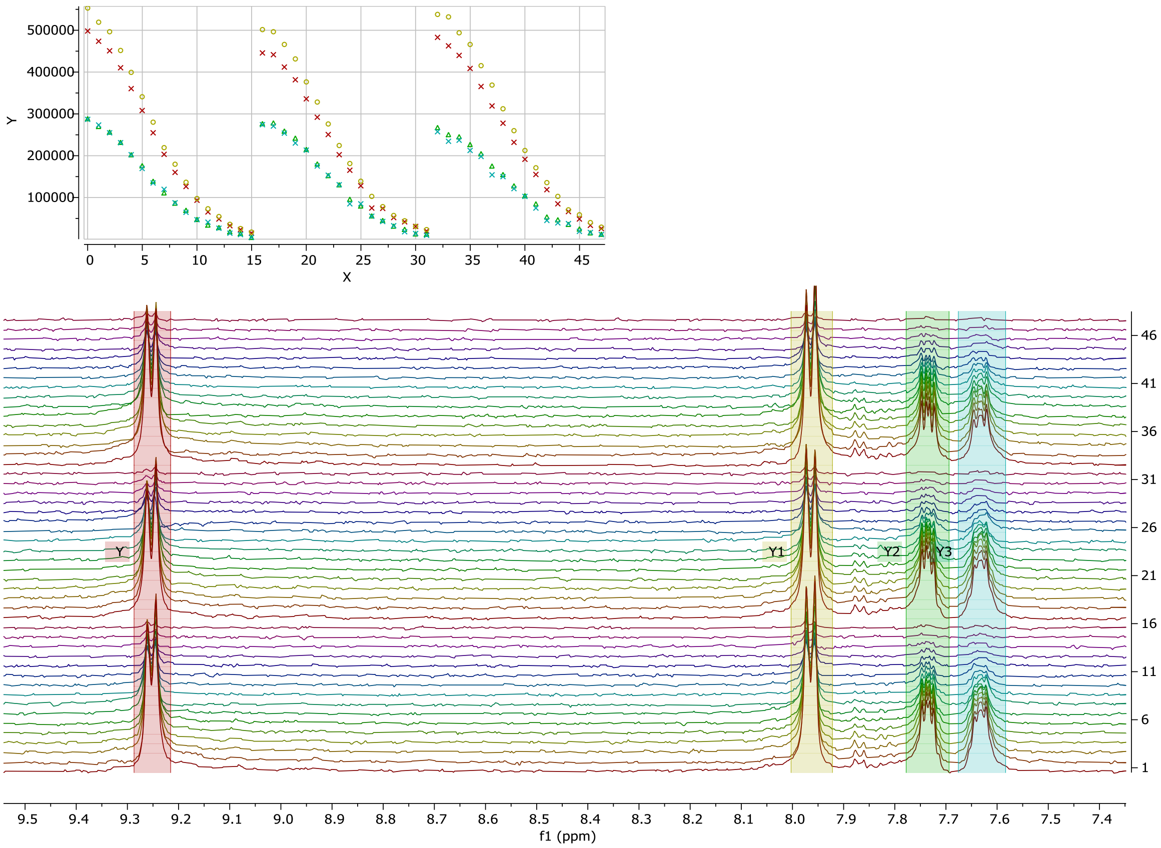

Figure S89. NMR spectra of the diffusion measurement of $[Pd_4(\mathbf{2})_8](BF_4)_8$.

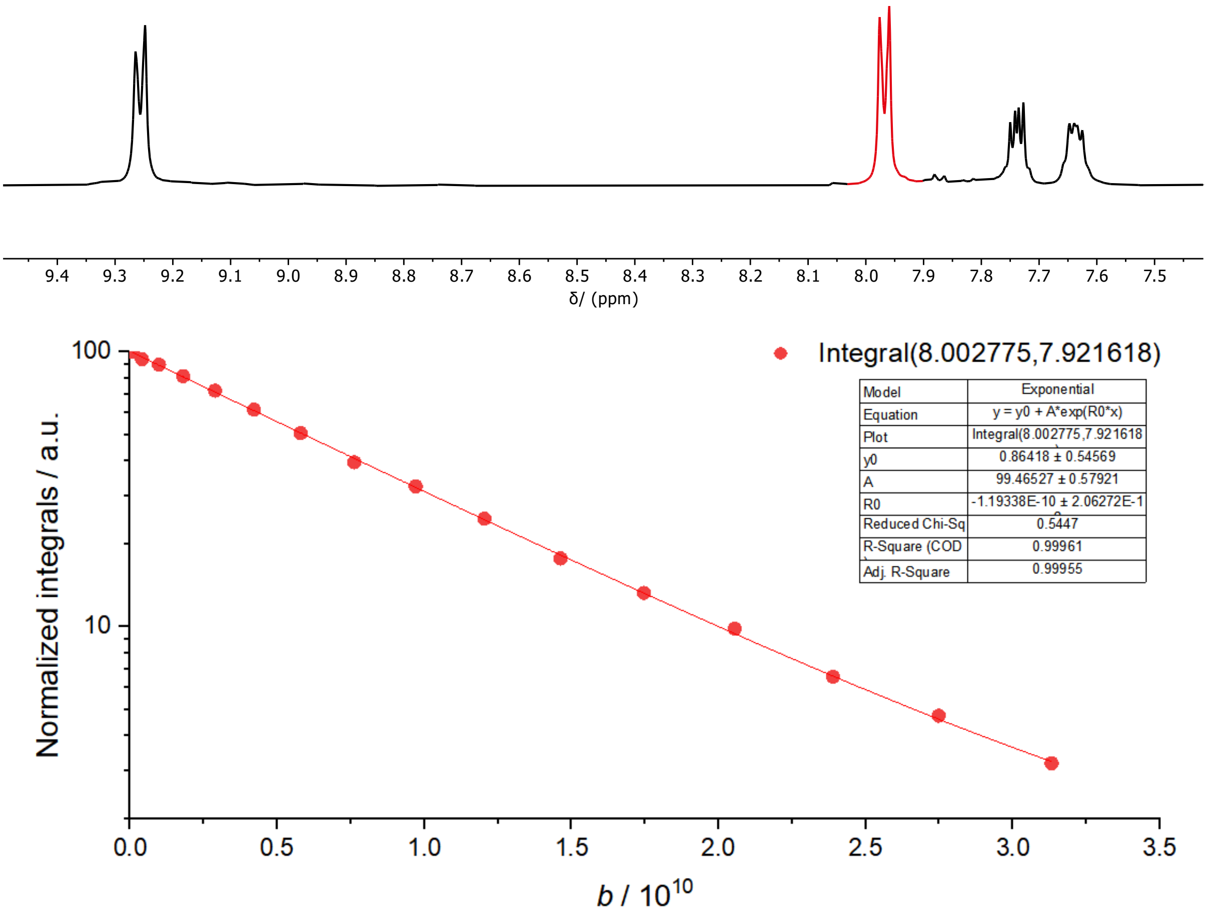

Figure S90 Fitting of integrals of $[Pd_4\ (\mathbf{2})_8](BF_4)_8$ from ^{1}H NMR diffusion. Fitting of integrals of $[Pd_4(\mathbf{2})_8](BF_4)_8$ from ^{1}H NMR diffusion experiment, ste, δ = 4.0 ms, Δ = 107.0 ms.. Data were fit to a monoexponential using corrected gradient values ($G_{corrected} = G_{app} \times 0.928$).

S14. Controlling catalysis with light using $[Pd_2(E\text{-}\mathbf{1})_2(\mathbf{2})_2](BAr_F)_4$ in $DCM/MeNO_2$ (9:1)

We tested whether we could use light to control the catalysis of the Michael's addition reaction (Scheme S3). A sample of $[Pd_2(E\text{-}\mathbf{1})_2(\mathbf{2})_2](BAr_F)_4$ was prepared by reacting homoleptic *E*-**1** cage $[Pd_2(E\text{-}\mathbf{1})_4](BAr_F)_4$ (1 equiv.) in acetonitrile with a mixture of homoleptic ligand **2** cages $[Pd_3(\mathbf{2})_6](BAr_F)_6$ and $[Pd_4(\mathbf{2})_8](BAr_F)_8$ (1 equiv.) in acetonitrile. The sample was heated to 50 °C and N_2 was blown over the sample to remove the solvent. The sample was redissolved in acetonitrile and the solvent was removed two additional times to remove Py*. The residue was redissolved in dichloromethane-d_2 (450 µL) nitromethane-d_3 (50 µL) to afford a sample of $[Pd_2(E\text{-}\mathbf{1})_2(\mathbf{2})_2](BAr_F)_4$ ([Pd] = 2.2 mM, 1 equiv.) for catalysis. The nitromethane was required to solubilize $[Pd_2(E\text{-}\mathbf{1})_2(\mathbf{2})_2](BAr_F)_4$ in dichloromethane. Solutions of methyl vinyl ketone (2.5 equiv.), benzoyl nitromethane (16.5 equiv.), and 18-crown-6 (2.5 equiv.) in dichlromethane-d_2 were added to the solution of $[Pd_2(E\text{-}\mathbf{1})_2(\mathbf{2})_2](BAr_F)_4$ ([Pd] = 2.1 mM) (Figure S91i). The reaction sample was kept in the dark at 25 °C (298 K) and monitored using ^1H NMR spectroscopy with a ^1H NMR spectrum collected every 5 min. After 17 hours, the sample was removed from the NMR instrument and irradiated with an LED centred at 530 nm for 10 min (Figure S91ii). The sample was monitored for an additional 4 hours then removed from the NMR instrument again and irradiated with 405 nm light for 10 min (Figure S91iii). The sample was then monitored for an additional 18 hours. The generation of the Michael addition product were determined by integrating the ^1H signal product signal at 2.66 ppm in each NMR spectrum. The signal intensities were plot against time to determine whether the rate of product formation was affected upon irradiation with 530 nm and 405 nm light (Figure S92).

Irradiating the sample with 530 nm light immediately reduced the rate of product formation, demonstrating that catalysis can be 'switched off' at will with visible light. After the sample was irradiated with 405 nm light the rate of product formation slowly started to increase over the 18 hours. The slow increase in rate of formation after irradiation suggests that the self-assembled system is slow to reach equilibrium resulting in the slow reformation of the catalytic species $[Pd_2(E\text{-}\mathbf{1})_2(\mathbf{2})_2](BAr_F)_4$. The reformation of $[Pd_2(E\text{-}\mathbf{1})_2(\mathbf{2})_2](BAr_F)_4$ might be improved by adding species that promote ligand exchange around palladium(II), which would increase the rate at which the self-assembled system equilibrates.

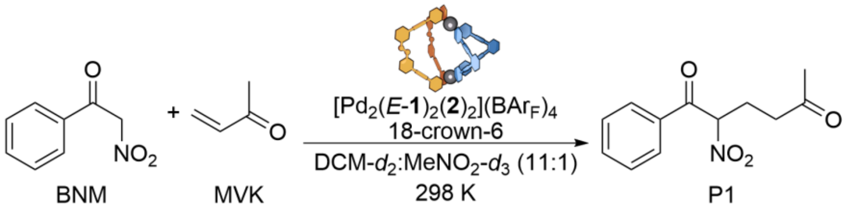

Scheme S3. Michael's addition between methyl vinyl ketone and benzoyl nitromethane using coordination cages as catalysts.

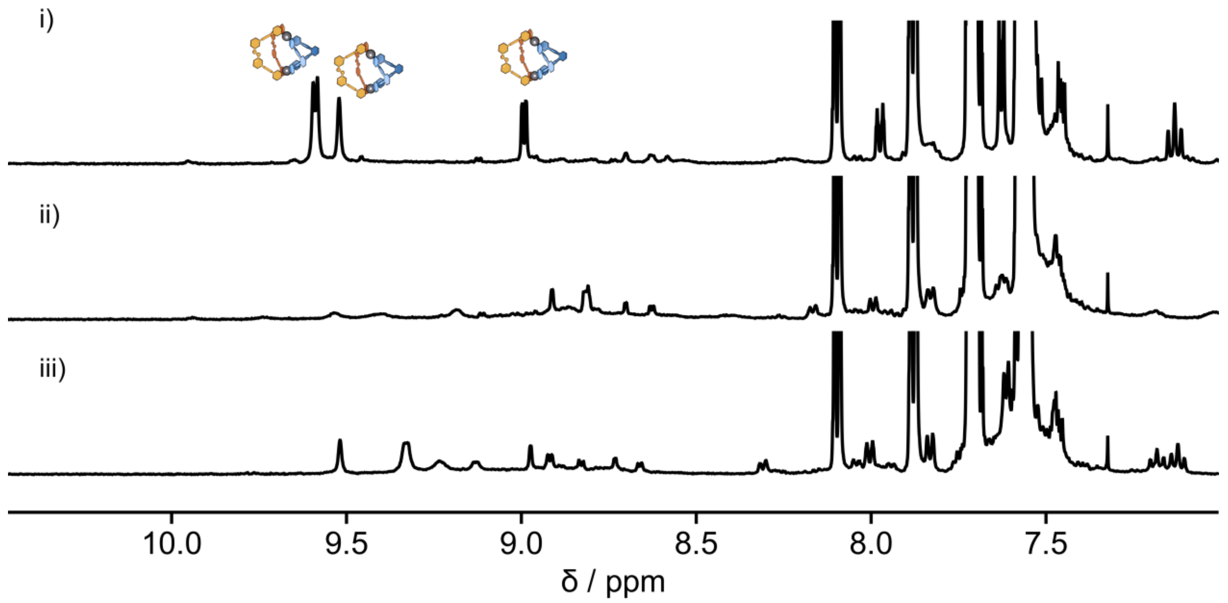

Figure S91. ^{1}H NMR spectra (500 MHz, DCM-d_2:MeNO$_2$ (9:1), 298 K) of i) [Pd$_2$(E-**1**)$_2$(**2**)$_2$](BAr$_F$)$_4$ ([Pd] = 2.1 mM, 1 equiv.) with methyl vinyl ketone (2.5 equiv.), benzoyl nitromethane (16.5 equiv.), and 18-crown-6 (2.5 mM, 2.5 equiv.), ii) the previous sample after being irradiated with 530 nm light for 10 min iii) the previous sample after being irradiated with 405 nm light for 10 min.

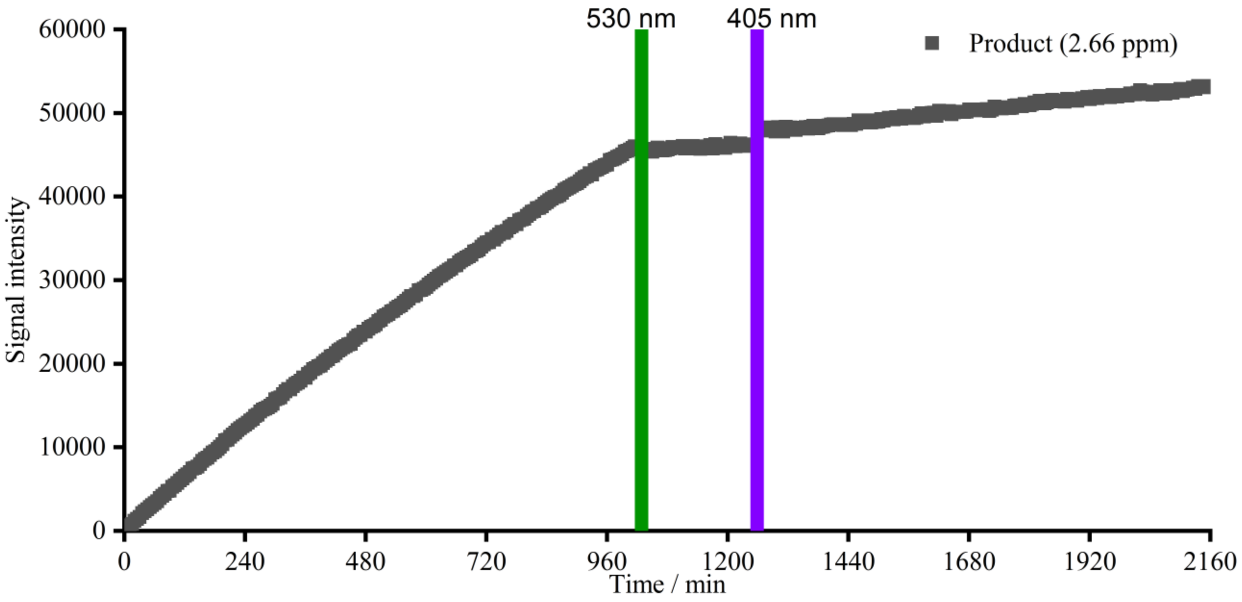

Figure S92. ^{1}H NMR (500 MHz, DCM-d_2:MeNO$_2$ (9:1), 298 K) integrals monitoring the formation of the Michael addition product in the presence of self-assembled product [Pd$_2$(E-**1**)$_2$(**2**)$_2$](BAr$_F$)$_4$ ([Pd] = 1.9 mM). Signal intensities were determined by integrating the product peak at 2.66 ppm. Vertical green line indicates when sample was irradiated with 530 nm light and vertical violet line indicates when sample was irradiated with 405 nm light.

S15. The effect of solvent on self-assembly

S15.1 The effects of solvents on the assembly of [Pd_2(E-**1**)$_2$(**2**)$_2$](BAr_F)$_4$

[Pd_2(E-**1**)$_4$](BAr_F)$_4$ ([Pd] = 1.96 mM, 270 µL, 0.53 µmol, 1.0 equiv.) and free Py* (4 equiv, 7.8 mM) was reacted with [Pd_3(**2**)$_6$](BAr_F)$_6$ ([Pd] = 1.96 mM, 270 µL, 0.53 µmol, 1.0 equiv.) and free Py* (4 equiv, 7.8 mM) in MeCN-d_3. The sample was heated to 50 °C for 30 min to thermally equilibrate and then argon was blown over the sample to remove the solvent. The residue was redissolved in MeCN-d_3 (500 µL) to afford a sample of [Pd_2(E-**1**)$_2$(**2**)$_2$](BAr_F)$_4$ ([Pd] = 2.12 mM, 500 µL, 1.06 µmol, 1.0 equiv.) with free Py* (4 equiv, 7.8 mM).^{1}H and ^{19}F NMR spectroscopy data was collected for this sample (Figure S93i), before it was heated to 50 °C and argon was blown over the sample to remove the solvent. The residue was redissolved in MeCN-d_3 (42 µL) and DCM-d_2 (458 µL) and ^{1}H and ^{19}F NMR spectroscopy data was collected again (Figure S93ii). The sample was heated a third time to 50 °C and argon was blown over it to remove the solvent. The residue was redissolved in DMSO-d_6 (500 µL) and ^{1}H and ^{19}F NMR spectroscopy data was collected (Figure S93iii).

The reaction between ligand **1**, ligand **2**, and [Pd(Py*)$_4$](BAr_F)$_2$ in MeCN-d_3, MeCN-d_3:DCM-d_2 (1:11) and DMSO-d_6 formed [Pd_2(E-**1**)$_2$(**2**)$_2$](BAr_F)$_4$. The ^{1}H and ^{19}F NMR spectroscopy of [Pd_2(E-**1**)$_2$(**2**)$_2$](BAr_F)$_4$ in MeCN-d_3, MeCN-d_3:DCM-d_2 (1:11) and DMSO-d_6 (Figure S93) demonstrate the self-assembled structure has one ligand environment for ligand **1** and ligand **2**, which is the same for [Pd_2(E-**1**)$_2$(**2**)$_2$](BF_4)$_4$ in DMSO-d_6 (Figure S59).

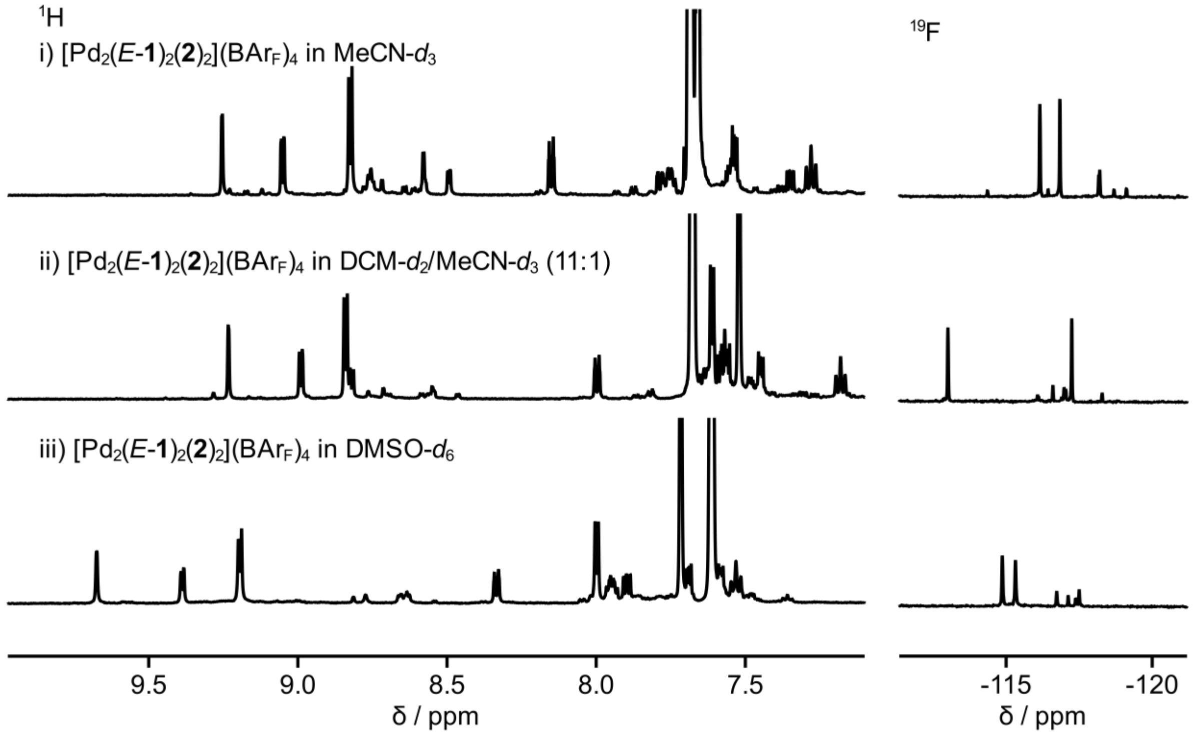

Figure S93.^{1}H (600 MHz, 298 K) and ^{19}F (565 MHz, 298 K) NMR spectra of [Pd_2(E-**1**)$_2$(**2**)$_2$](BAr_F)$_4$ ([Pd] = 2.12 mM) in i) MeCN-d_3, ii) DCM-d_2:MeCN-d_3 (11:1) and in iii) DMSO-d_6.

S15.2 The effects of solvents on the assembly of $[Pd_2(\textit{E}\text{-}\mathbf{1})_4](BArF)_4$

A sample of $[Pd_2(\textit{E}\text{-}\mathbf{1})_4](BAr_F)_4$ ([Pd] = 1.96 mM, 500 µL, 0.98 µmol, 1.0 equiv.) and free Py* (4 equiv, 7.8 mM) was prepared in in MeCN-d_3. The sample was heated to 50 °C for 30 min to thermally equilibrate and ^{1}H NMR spectroscopy data was collected for this sample (Figure S94i). Then it was heated to 50 °C and argon was blown over the sample to remove the solvent. The residue was redissolved in MeCN-d_3 (42 µL) and DCM-d_2 (458 µL) and ^{1}H NMR spectroscopy data was collected again (Figure S94ii) showing that the ligand environment in $[Pd_2(\textit{E}\text{-}\mathbf{1})_4](BAr_F)_4$ is the same in MeCN-d_3 as in MeCN-d_3:DCM-d_2 (1:11).

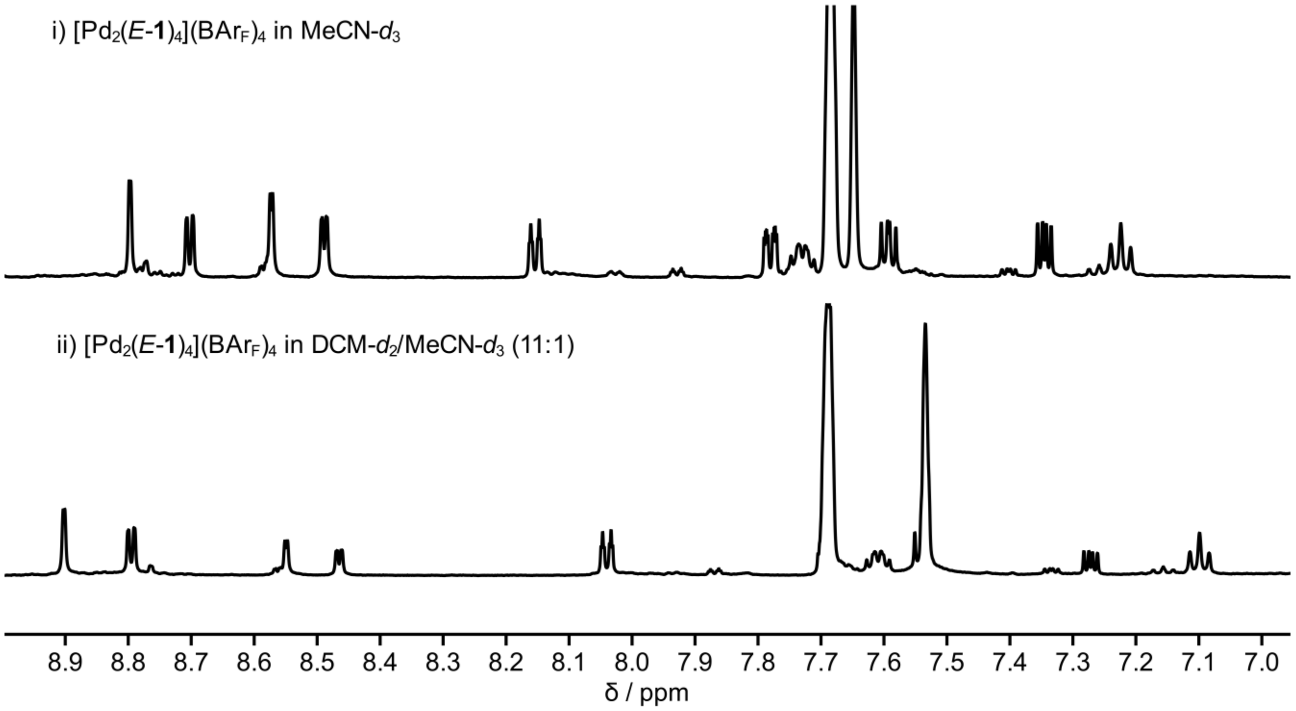

Figure S94. ^{1}H (600 MHz, 298 K) spectra of $[Pd_2(\textit{E}\text{-}\mathbf{1})_4](BAr_F)_4$ ([Pd] = 1.96 mM) in i) MeCN-d_3 and in ii) DCM-d_2:MeCN-d_3 (11:1).

S15.3 The effects of solvents on the assembly of $[Pd_3(\mathbf{2})_6](BAr_F)_6$

A sample of $[Pd_3(\mathbf{2})_6](BAr_F)_6$ ([Pd] = 1.96 mM, 500 µL, 0.98 µmol, 1.0 equiv.) and free Py* (4 equiv, 7.8 mM) was prepared in in MeCN-d_3. The sample was heated to 50 °C for 30 min to thermally equilibrate and ^{1}H NMR spectroscopy data was collected for this sample (Figure S95i). Then it was heated to 50 °C and argon was blown over the sample to remove the solvent. The residue was redissolved in MeCN-d_3 (42 µL) and DCM-d_2 (458 µL) and ^{1}H NMR spectroscopy data was collected again (Figure S95ii) showing that the ligand environment in $[Pd_3(\mathbf{2})_6](BAr_F)_6$ is the same in MeCN-d_3 as in MeCN-d_3:DCM-d_2 (1:11).

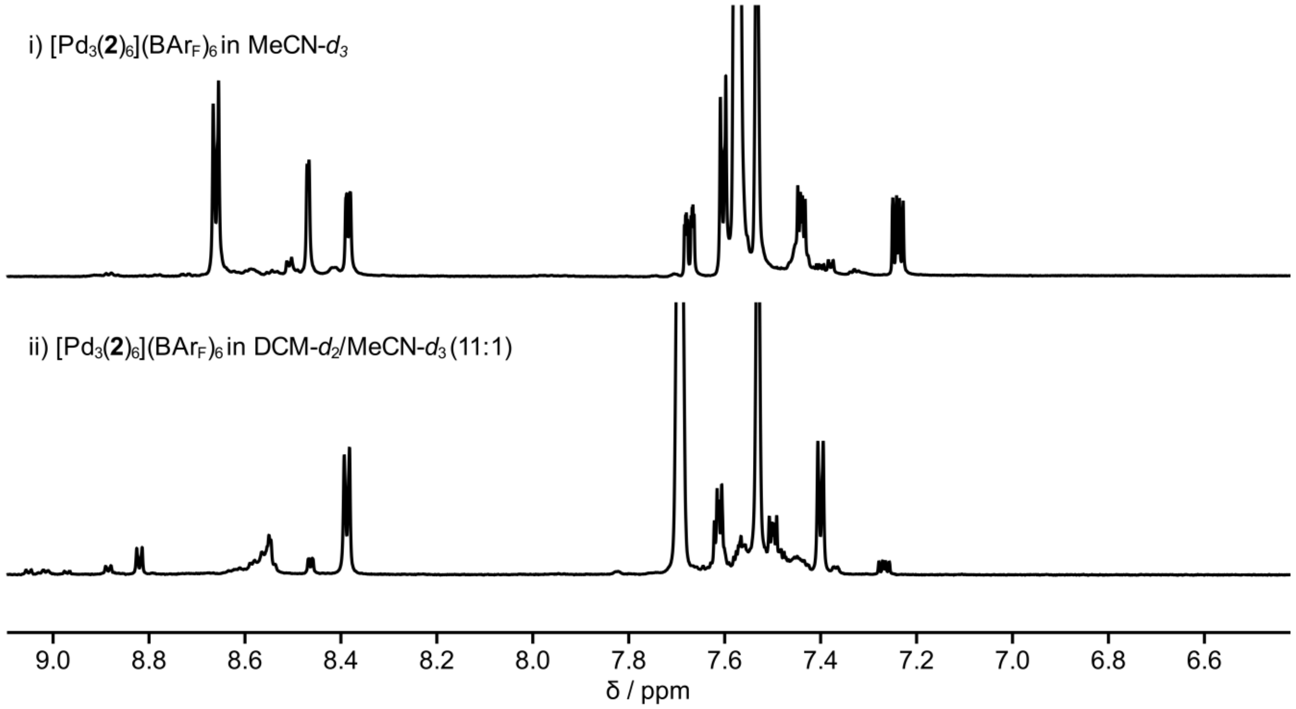

Figure S95. ^{1}H (600 MHz, 298 K) spectra of $[Pd_3(\mathbf{2})_6](BAr_F)_6$ ([Pd] = 1.96 mM) in i) MeCN-d_3 and in ii) DCM-d_2:MeCN-d_3 (11:1).

S16. The effect of 3-chloropyridine (Py*) on the self-assembly of $[Pd_2(E\text{-}\mathbf{1})_2(\mathbf{2})_2](BAr_F)_4$ in DCM:MeCN (10:1)

We used $[Pd(3\text{-chloropyridine})_4](BAr_F)_2$ as the source of the BAr_F anion.[11] Self-assembly between ligand *E*-**1**, ligand **2** and palladium(II) generates free Py*. Py* weakly coordinates to palladium(II)[11], and has been demonstrated to increase the rate of self-assembly.[16] We wanted to investigate whether Py* affects the self-assembly of $[Pd_2(E\text{-}\mathbf{1})_2(\mathbf{2})_2](BAr_F)_4$ in DCM:MeCN (10:1) after irradiation with 530 nm and 405 nm light.

S16.1 Photoswitching of $[Pd_2(E\text{-}\mathbf{1})_2(\mathbf{2})_2](BAr_F)_4$ in the presence of 3-chloropyridine in DCM:MeCN (10:1)

To evaluate the influence of Py* on the photoswitching process of $[Pd_2(E\text{-}\mathbf{1})_2(\mathbf{2})_2](BAr_F)_4$ a sample was prepared according to following procedure:

$[Pd_2(E\text{-}\mathbf{1})_4](BAr_F)_4$ ([Pd] = 1.96 mM, 270 µL, 0.53 µmol, 1.0 equiv.) and free Py* (4 equiv, 7.8 mM) was reacted with $[Pd_3(\mathbf{2})_6](BAr_F)_6$ ([Pd] = 1.96 mM, 270 µL, 0.53 µmol, 1.0 equiv.) and free Py* (4 equiv, 7.8 mM) in MeCN-d_3. The sample was heated to 50 °C and argon was blown over the sample to remove the solvent. The residue was redissolved in MeCN-d_3 (45 µL) and DCM-d_2 (455 µL) to afford a sample of $[Pd_2(E\text{-}\mathbf{1})_2(\mathbf{2})_2](BAr_F)_4$ ([Pd] = 2.12 mM, 500 µL, 1.06 µmol, 1.0 equiv.) with free Py* (4 equiv, 7.8 mM).

A ^{1}H NMR spectrum was collected (Figure S96i) before the sample was irradiated with an LED centred at 530 nm for 10 minutes (Figure S96ii). The sample was then removed from the NMR instrument, irradiated with 405 nm light for 5 min and immediately placed back into the NMR instrument (Figure S96iii). The ^{1}H NMR spectra demonstrate that $[Pd_2(E\text{-}\mathbf{1})_2(\mathbf{2})_2](BAr_F)_4$ can be reversibly disassembled and reassembled in DCM-d_2:MeCN-d_3 (10:1) in the presence of Py* (4 equiv., 7.8 mM).

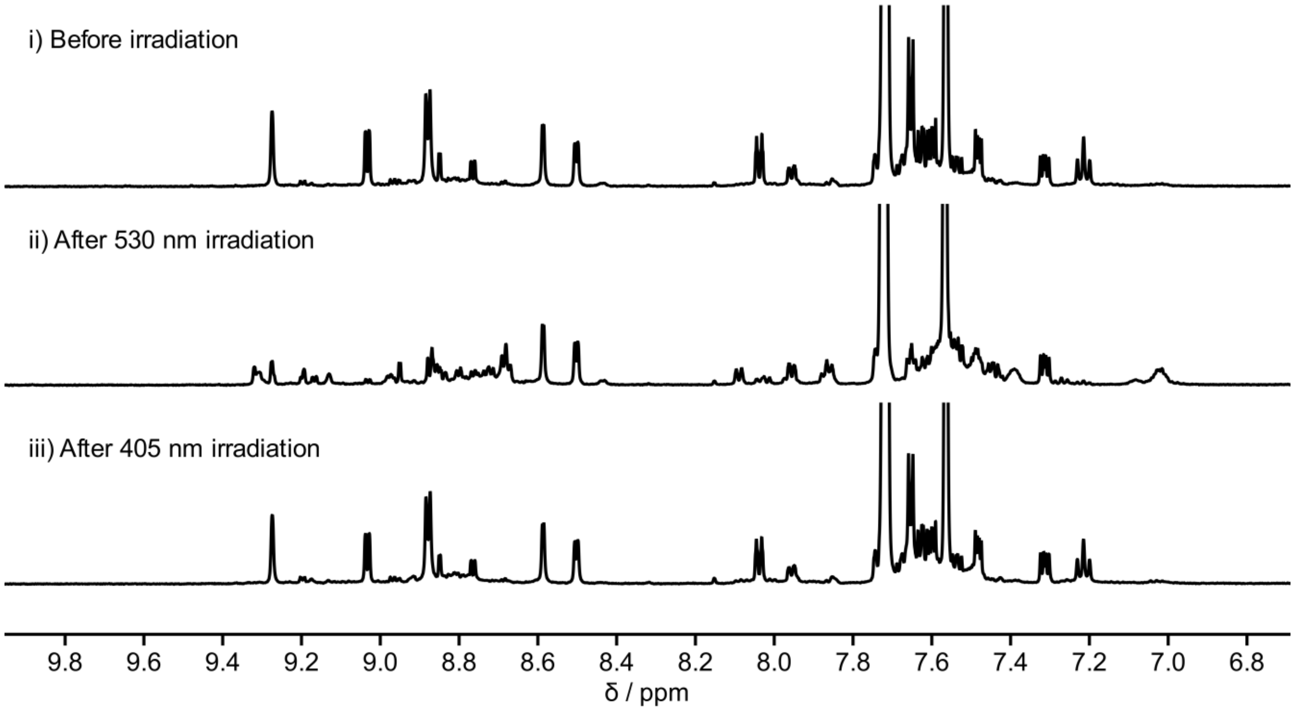

Figure S96. ^{1}H NMR spectra (600 MHz, 298 K, DCM-d_2:MeCN-d_3 (10:1)) of i) $[Pd_2(E\text{-}\mathbf{1})_2(\mathbf{2})_2](BAr_F)_4$ ([Pd] = 2.12 mM) in the presence of Py*, ii) the previous sample after being irradiated with 530 nm light for 10 min, iii) the previous sample after being irradiated with 405 nm light for 5 min.

S16.2 Photoswitching of $[Pd_2(E\text{-}1)_2(2)_2](BAr_F)_4$ without Py* in DCM:MeCN (10:1)

A sample of $[Pd_2(E\text{-}\mathbf{1})_2(\mathbf{2})_2](BAr_F)_4$ without 3-chloropyridine was prepared by reacting a sample of $[Pd_2(E\text{-}\mathbf{1})_4](BAr_F)_4$ ([Pd] = 1.96 mM, 270 µL, 0.53 µmol, 1.0 equiv.) + free Py* (4 equiv, 7.8 mM) with $[Pd_3(\mathbf{2})_6](BAr_F)_6$ ([Pd] = 1.96 mM, 270 µL, 0.53 µmol, 1.0 equiv.) + free Py* (4 equiv, 7.8 mM). The solvent was removed under reduced pressure at 50 °C. Acetonitrile (4 mL) was added to the residue and the solvent was removed again. This procedure was repeated in total three times to remove all the Py*. The residue was redissolved in MeCN-d_3 (45 µL) and DCM-d_2 (455 µL) to afford a sample of $[Pd_2(E\text{-}\mathbf{1})_2(\mathbf{2})_2](BAr_F)_4$ ([Pd] = 2.12 mM, 500 µL, 1.06 µmol, 1.0 equiv.) (Figure S97i).

This sample was analysed using ^{1}H NMR spectroscopy (Figure S97i) before being irradiated with a LED centred at 530 nm for 10 minutes (Figure S97ii). The sample was then removed from the NMR instrument, irradiated with 405 nm light for 5 min and immediately placed back into the NMR instrument (Figure S97iii).

The ^{1}H NMR spectra demonstrate that $[Pd_2(E\text{-}\mathbf{1})_2(\mathbf{2})_2](BAr_F)_4$ can be reversibly disassembled and reassembled in DCM-d_2:MeCN-d_3 (10:1) in the absence of Py*. This indicates that Py* has no significant effect on the self-assembly of $[Pd_2(E\text{-}\mathbf{1})_2(\mathbf{2})_2](BAr_F)_4$ in DCM-d_2:MeCN-d_3 after irradiation with 405 nm and 530 nm light.

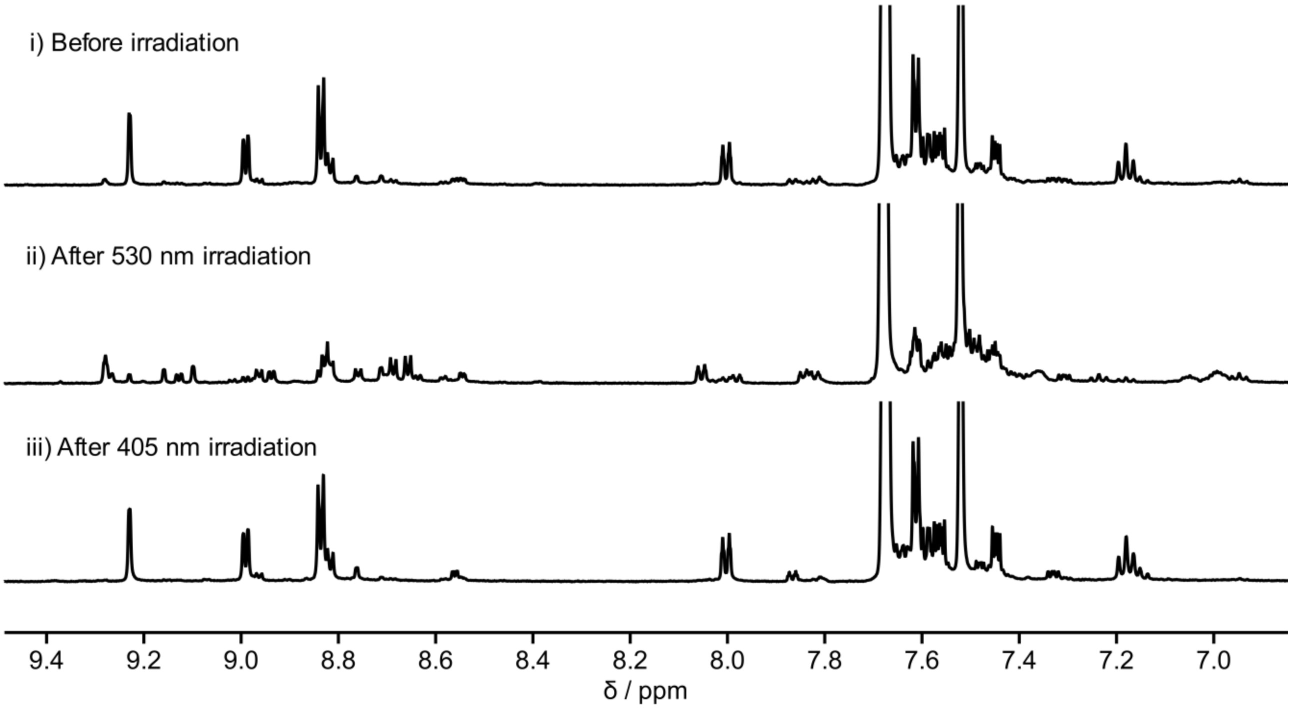

Figure S97. ^{1}H NMR spectra (600 MHz, 298 K, DCM-d_2:MeCN-d_3 (10:1)) of i) $[Pd_2(E\text{-}\mathbf{1})_2(\mathbf{2})_2](BAr_F)_4$ ([Pd] = 2.12 mM) in the absence of Py*, ii) the previous sample after being irradiated with 530 nm light for 10 min, iii) the previous sample after being irradiated with 405 nm light for 5 min.

S17. Guest binding studies in heteroleptic cage [Pd_2(*E*-1)$_2$(2)$_2$](BAr_F)$_4$ in 11:1 DCM:MeCN

Preparation of the [Pd_2(*E*-1)$_2$(2)$_2$](BAr_F)$_4$ stock solution:
A stock solution of [Pd_2(*E*-**1**)$_2$(**2**)$_2$](BAr_F)$_4$ was prepared by reacting a sample of ligand *E*-**1** (4.4 mM, 1.0 mL, DCM-d_2:MeCN-d_3 4:6, 1.0 equiv.), ligand **2** (4.4 mM, 1.0 mL, MeCN-d_3, 1.0 equiv.) and [Pd(Py*)$_4$](BAr_F)$_2$ (22.0 mM, 200 μL, MeCN-d_3, 1.0 equiv.). The sample was heated to 50 °C for 1 hr to thermally equilibrate and then argon was blown over the sample to remove the solvent. The residue was redissolved in DCM-d_2:MeCN-d_3 (11:1, 2.0 mL) to afford a sample of [Pd_2(*E*-**1**)$_2$(**2**)$_2$](BAr_F)$_4$ ([Pd] = 2.2 mM, 2.0 mL).

Preparation of the methyl vinyl ketone stock solution:
Methyl vinyl ketone (9.6 μL, 7.7 mg, 110 μmol) was dissolved in DCM-d_2:MeCN-d_3 (11:1, 200 μL) to afford a stock solution of methyl vinyl ketone (551 mM).

Preparation of the benzoyl nitromethane stock solution:
Benzoyl nitromethane (18.2 mg, 110 μmol) was dissolved in DCM-d_2:MeCN-d_3 (11:1, 200 μL) to afford a stock solution of benzoyl nitromethane (551 mM).

Preparation of the 18-crown-6 stock solution:
18-crown-6 (29.1 mg, 110 μmol) was dissolved in DCM-d_2:MeCN-d_3 (11:1, 200 μL) to afford a stock solution of 18-crown-6 (551 mM).

Preparation of the tetrabutylammonium triflate stock solution:
Tetrabutylammonium triflate (4.3 mg, 11 μmol) was dissolved in DCM-d_2:MeCN-d_3 (11:1, 200 μL) to afford a stock solution of 18-crown-6 (55 mM).

We investigated ^{1}H NMR peak shifts on addition of each of the substrates methyl vinyl ketone and benzoyl nitromethane by the heteroleptic cage Pd_2(*E*-**1**)$_2$(**2**)$_2$](BAr_F)$_4$ in the same solvent mixture used for the catalysis experiments. We found that the addition of methyl vinyl ketone or 18-crown-6 did not result in significant peak shifts, consistent with weak binding interactions. Similarly, the addition of 18-crown-6 to a sample of the cage with 40 equiv. of methyl vinyl ketone did not result in any significant changes.

By contrast, the addition of benzoyl nitromethane resulted in the formation of a new cage species in slow exchange, which corresponds to the binding of the deprotonated benzoyl nitromethane. The presence of 3-chloropyridine likely deprotonates some of the benzoyl nitromethane to lead to binding of the anion. When 18-crown-6 is added to the sample the signals of the cage with bound deprotonated benzoyl nitromethane increase as the signals of the free cage decrease. This is consistent with the cage being close to saturated with bound deprotonated benzoyl nitromethane.

S17.1 Binding of methyl vinyl ketone by $[Pd_2(E\text{-}\mathbf{1})_2(\mathbf{2})_2](BAr_F)_4$

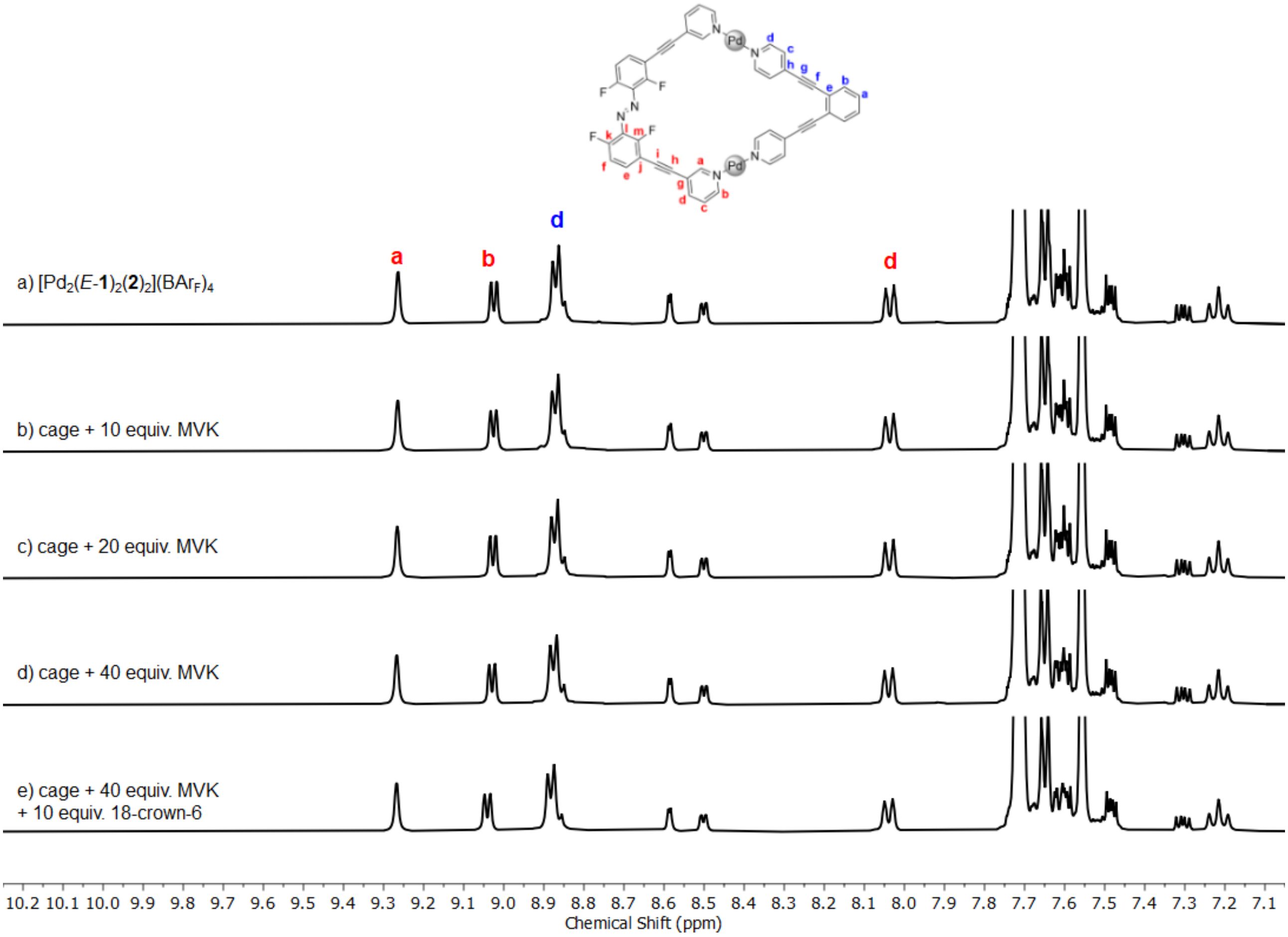

Figure S98. ^{1}H NMR spectra (400 MHz, 298 K, DCM-d_2:MeCN-d_3 (11:1)) of a) $[Pd_2(E\text{-}\mathbf{1})_2(\mathbf{2})_2](BAr_F)_4$ ([Pd] = 2.2 mM, 2.0 equiv.) and with b) 10 equiv. methyl vinyl ketone (10 µL), c) 20 equiv. methyl vinyl ketone (20 µL), d) 40 equiv. methyl vinyl ketone (40 µL), and e) 40 equiv. methyl vinyl ketone (40 µL) and 10 equiv. of 18-crown-6 (10 µL). No significant peak shifts were observed. Concentration of the methyl vinyl ketone stock solution added = 551 mM in DCM-d_2:MeCN-d_3 (11:1).

S17.2 Binding of benzoyl nitromethane by $[Pd_2(E\text{-}\mathbf{1})_2(\mathbf{2})_2](BAr_F)_4$

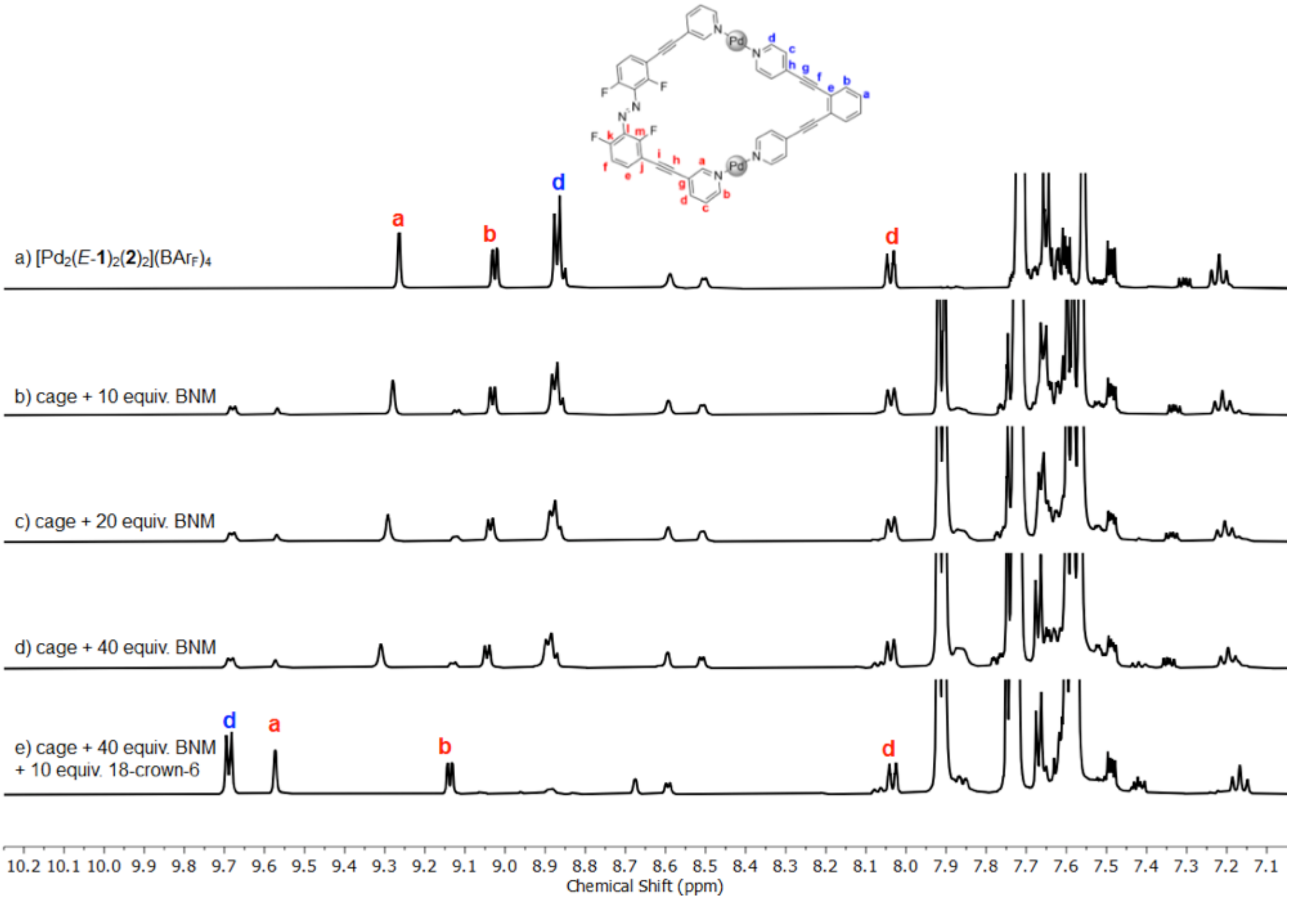

Figure S99. ^1H NMR spectra (500 MHz, 298 K, DCM-d_2:MeCN-d_3 (11:1)) of a) $[Pd_2(E\text{-}\mathbf{1})_2(\mathbf{2})_2](BAr_F)_4$ ([Pd] = 2.2 mM, 2.0 equiv.) and with b) 10 equiv. benzoyl nitromethane (10 µL), c) 20 equiv. benzoyl nitromethane (20 µL), d) 40 equiv. benzoyl nitromethane (40 µL), and e) 40 equiv. benzoyl nitromethane (40 µL) and 10 equiv. of 18-crown-6 (10 µL). The formation of a new species corresponding to the bound deprotonated benzoyl nitromethane is observed, where deprotonation is promoted by the addition of 18-crown-6. Concentration of the benzoyl nitromethane stock solution added = 551 mM in DCM-d_2:MeCN-d_3 (11:1)

S17.3 Binding of 18-crown-6 by [Pd_2(*E*-1)$_2$(2)$_2$](BAr_F)$_4$

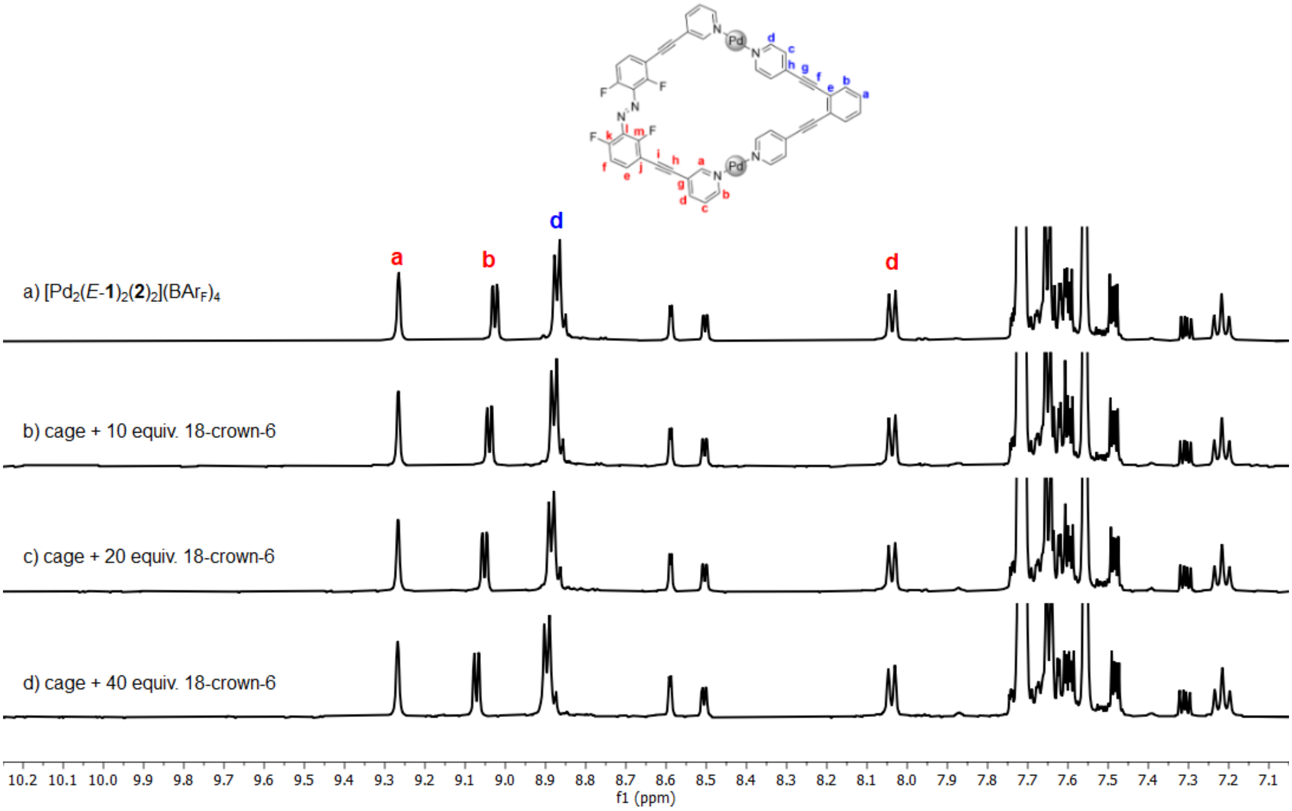

Figure S100. ^{1}H NMR spectra (500 MHz, 298 K, DCM-d_2:MeCN-d_3 (11:1)) of a) [Pd_2(*E*-**1**)$_2$(**2**)$_2$](BAr_F)$_4$ ([Pd] = 2.2 mM, 2.0 equiv.) and with b) 10 equiv. 18-crown-6 (10 µL), c) 20 equiv. 18-crown-6 (20 µL), and d) 40 equiv. 18-crown-6 (40 µL). The signal most significantly shifted is at 9.0 ppm, corresponding to H^b which is outside the cage. Concentration of the 18-crown-6 stock solution added = 551 mM in DCM-d_2:MeCN-d_3 (11:1).

S17.4 Binding of triflate (OTf^-) by $[Pd_2(E\text{-}\mathbf{1})_2(\mathbf{2})_2](BAr_F)_4$

Tetrabutylammonium triflate (TBAOTf) was added to a sample of the free cage in 11:1 DCM:MeCN. At least two separate binding events are occurring. Firstly, a triflate anion is bound within the cage, to give new series of peaks from the cage in slow exchange on the NMR timescale with the free cage. The signals most affected are H^a and H^d which would be directed inside the cage. The signal for H^b, which must be directed outside the cage, has only a minor shift.

When more than 1 equiv. of TBAOTf is added a new binding event occurs on the outside of the cage, which is a fast exchange process on the NMR timescale. As more triflate is added the average species is a bound OTf within the cage and the outside of the cage mostly bound by OTf anions. The signals most affected by this binding event on the outside of the cage are H^b and H^d, as expected for external binding.

As there are multiple binding events (ultimately all four BArF anions will be replaced with OTf), we have not attempted to determine relative binding constants.

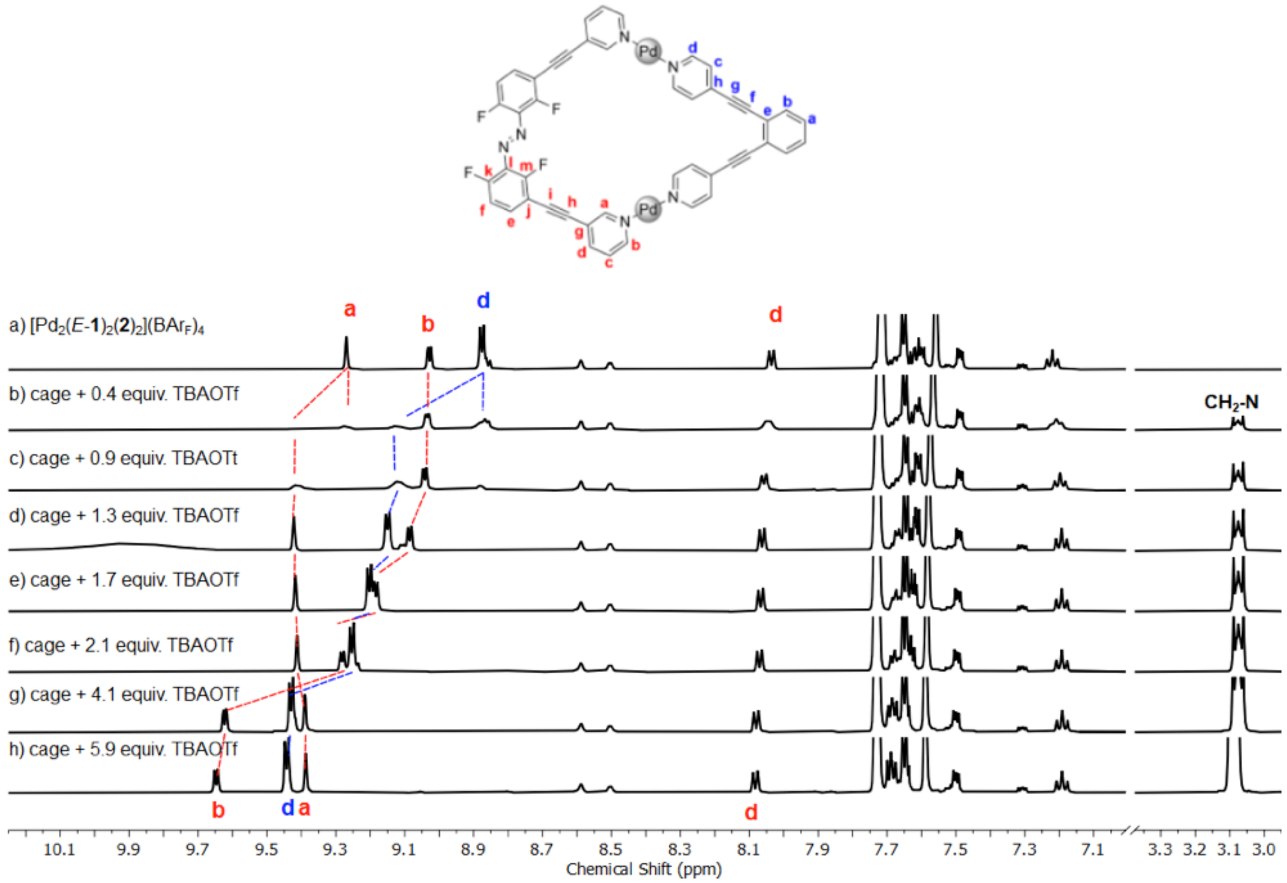

Figure S101. ^1H NMR spectra (500 MHz, 298 K, DCM-d_2:MeCN-d_3 (11:1)) of a) $[Pd_2(E\text{-}\mathbf{1})_2(\mathbf{2})_2](BAr_F)_4$ ([Pd] = 2.2 mM, 2.0 equiv.) and with b) 0.4 equiv. tetrabutylammonium triflate (TBAOTf), c) 0.9 equiv. TBAOTf, d) 1.3 equiv. TBAOTf, e) 1.7 equiv. TBAOTf, f) 2.1 equiv. TBAOTf, g) 4.1 equiv. TBAOTf and h) 5.9 equiv. TBAOTf.

S18. Catalyzing the Michael addition between methyl vinyl ketone and benzoyl nitromethane

S18.1 Preparation of stock solutions for catalysis samples

Preparation of the [Pd(Py*)$_4$](BAr$_F$)$_2$ stock solution:
[Pd(Py*)$_4$](BArF)$_2$ (54.6 mg, 23.9 µmol) was dissolved in DCM-*d*$_2$ (1.0 mL) to afford a stock solution of [Pd(Py*)$_4$](BAr$_F$)$_2$ ([Pd] = 23.9 mM).

Preparation of the [Pd$_2$(*E*-1)$_4$](BAr$_F$)$_4$ stock solution:
To prepare an isomerically pure sample of *E*-**1**, a sample containing a mixture of *E*-**1** and *Z*-**1** was dissolved in anhydrous acetonitrile. The solution was heated in a microwave reactor at 130 °C for 15 min, then the solvent was removed under reduced pressure. Ligand *E*-**1** (6.25 mg, 13.7 µmol, 2.0 equiv.) was dissolved in MeCN-*d*$_3$ (3.2 mL) and a stock solution of [Pd(Py*)$_4$](BAr$_F$)$_2$ (280 µL, 23.9 mM, 1.0 equiv.) was added. The solution was prepared in the dark and was left to equilibrate at room temperature for 30 minutes to afford a stock solution of [Pd$_2$(*E*-**1**)$_4$](BAr$_F$)$_4$ ([Pd] = 1.96 mM) and free Py*.

Preparation of the [Pd$_3$(2)$_6$](BAr$_F$)$_6$ stock solution:
Ligand **2** (4.03 mg, 14.4 µmol, 2.0 equiv.) was dissolved in MeCN-*d*$_3$ (3.4 mL) and a stock solution of [Pd(Py*)$_4$](BAr$_F$)$_2$ (300 µL, 23.9 mM, 1.0 equiv.) was added. The solution was equilibrated at 50 °C for 30 minutes to afford a stock solution of [Pd$_3$(**2**)$_6$](BAr$_F$)$_6$ ([Pd] = 1.96 mM) and free Py*.

Preparation of the methyl vinyl ketone stock solution:
Methyl vinyl ketone (10 µL, 8.41 mg, 120 µmol) was dissolved in DCM-*d*$_2$ (190 µL) to afford a stock solution of methyl vinyl ketone (600 mM).

Preparation of the benzoyl nitromethane stock solution:
Benzoyl nitromethane (21.8 mg, 132 µmol) was dissolved in DCM-*d*$_2$ (500 µL) to afford a stock solution of benzoyl nitromethane (264 mM).

Preparation of the 18-crown-6 stock solution:
18-crown-6 (10.2 mg, 38.6 µmol) was dissolved in DCM-*d*$_2$ (300 µL) to afford a stock solution of 18-crown-6 (129 mM).

S18.2 Preparation of self-assembled species samples for catalysis

Preparation of the [Pd$_2$(*E*-1)$_4$](BAr$_F$)$_4$ sample:
[Pd$_2$(*E*-**1**)$_4$](BAr$_F$)$_4$ stock solution ([Pd] = 1.96 mM, 450 µL, 0.88 µmol, 1.0 equiv.) was transferred into a 4 mL vial. The sample was heated to 50 °C under an argon flow to remove the solvent. The residue was redissolved in MeCN-*d*$_3$ (45 µL) and DCM-*d*$_2$ (405 µL) to afford a sample of [Pd$_2$(*E*-**1**)$_4$](BAr$_F$)$_4$ ([Pd] = 1.96 mM, 450 µL, 0.88 µmol, 1.0 equiv.) (Figure S102) for catalysis.

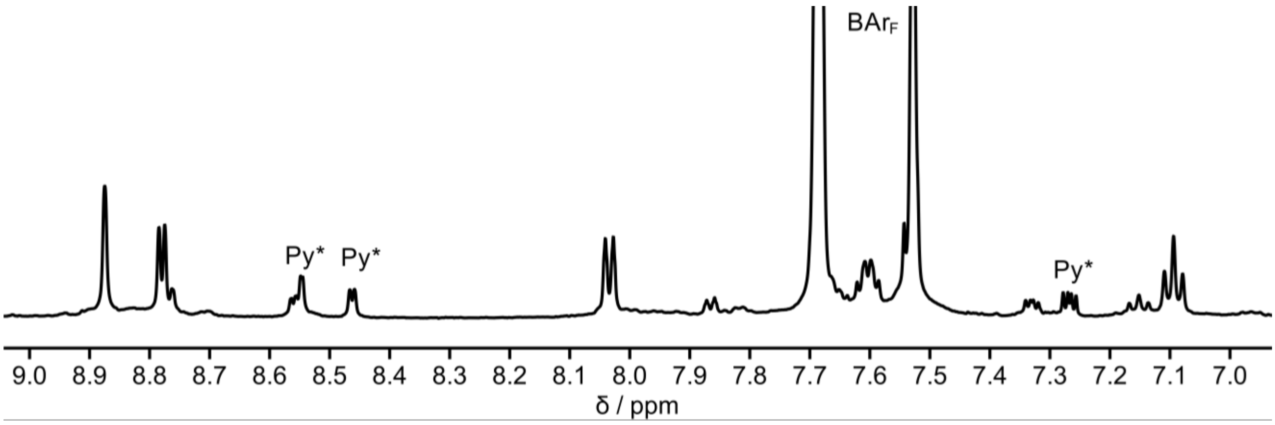

Figure S102. ^{1}H NMR (600 MHz, 298 K, DCM-d_2/MeCN-d_3 (9:1)) spectrum of $[Pd_2(E\text{-}\mathbf{1})_4](BAr_F)_4$.

Preparation of the $[Pd_3(2)_6](BAr_F)_6$ stock solution:

$[Pd_3(\mathbf{2})_6](BAr_F)_6$ stock solution ([Pd] = 1.96 mM, 450 µL, 0.88 µmol, 1.0 equiv.) was transferred into a 4 mL vial. The sample was heated to 50 °C under an argon flow to remove the solvent. The residue was redissolved in MeCN-d_3 (45 µL) and DCM-d_2 (405 µL) to afford a sample of $[Pd_3(\mathbf{2})_6](BAr_F)_6$ ([Pd] = 1.96 mM, 450 µL, 0.88 µmol, 1.0 equiv.) (Figure S103) for catalysis.

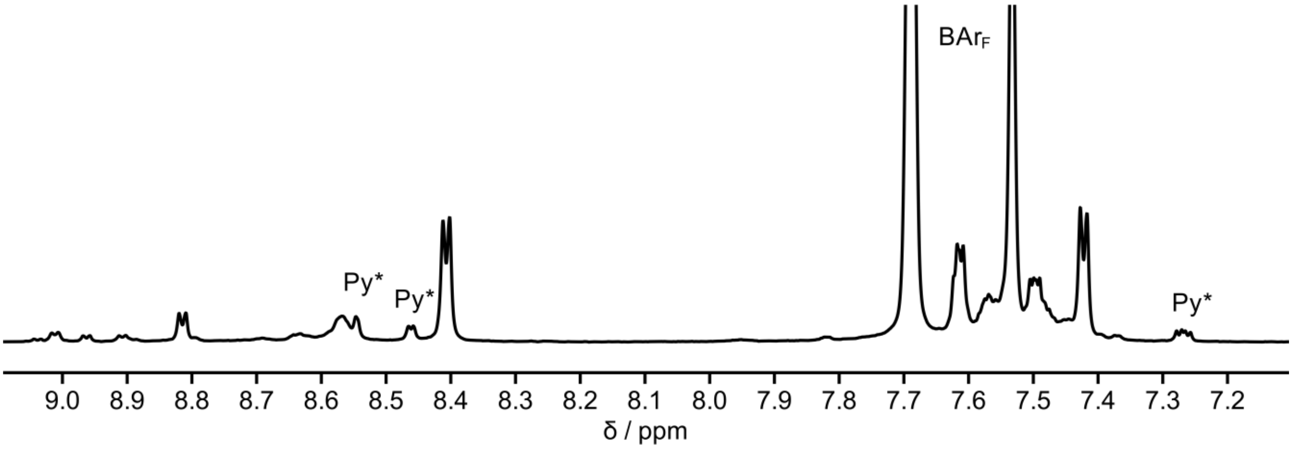

Figure S103. ^{1}H NMR (600 MHz, 298 K, DCM-d_2/MeCN-d_3 (9:1)) spectrum of $[Pd_3(\mathbf{2})_6](BAr_F)_6$.

Preparation of the $[Pd_2(E\text{-}1)_2(2)_2](BAr_F)_4$ stock solution:

A sample of $[Pd_2(E\text{-}\mathbf{1})_2(\mathbf{2})_2](BAr_F)_4$ was prepared by reacting $[Pd_2(E\text{-}\mathbf{1})_4](BAr_F)_4$ from the stock solution ([Pd] = 1.96 mM, 225 µL, 0.44 µmol, 1.0 equiv.) with the stock solution of $[Pd_3(\mathbf{2})_6](BAr_F)_6$ ([Pd] = 1.96 mM, 225 µL, 0.44 µmol l, 1.0 equiv.). The sample was heated to 50 °C and argon was blown over the sample to remove the solvent. The residue was redissolved in MeCN-d_3 (45 µL) and DCM-d_2 (405 µL) to afford a sample of $[Pd_2(E\text{-}\mathbf{1})_2(\mathbf{2})_2](BAr_F)_4$ ([Pd] = 1.96 mM, 450 µL, 0.88 µmol, 1.0 equiv.) (Figure S104) for catalysis.

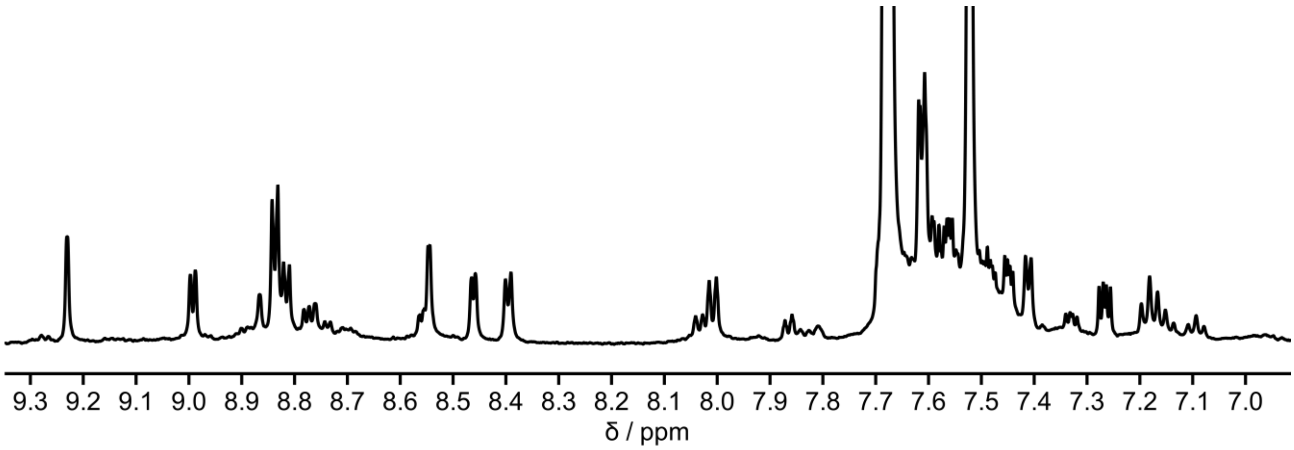

Figure S104. ^{1}H NMR (600 MHz, 298 K, DCM-d_2/MeCN-d_3 (9:1)) spectrum of $[Pd_2(E\text{-}\mathbf{1})_2(\mathbf{2})_2](BAr_F)_4$.

S18.3 Monitoring conversion of benzoyl nitromethane to the Michael addition product using ^{1}H NMR spectroscopy

S18.3.1 Determining T_1 values for benzoyl nitromethane and the Michael addition product in DCM:MeCN (11:1)

The T_1 relaxation values for the benzoyl nitromethane ^{1}H NMR signal at 5.97 ppm and the Michael addition product signal at 2.66 ppm (Figure S105) were measured using ^{1}H NMR spectroscopy so that the relaxation delay (Bruker setting D1) can be set appropriately to measure quantitative signal intensities. These signals were selected as they do not overlap with other signals.

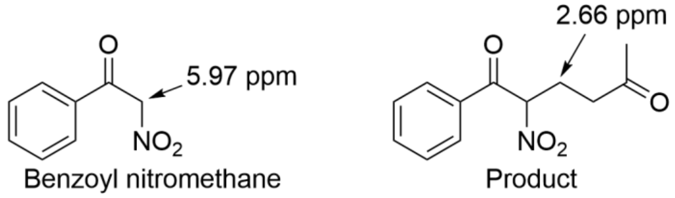

Figure S105. Benzoyl nitromethane and Michael addition product ^{1}H environments used to monitor the Michael addition between benzoyl nitromethane and methyl vinyl ketone.

To measure T_1 for each signal, a catalysis sample was prepared by charging an NMR tube with $[Pd_2(E\text{-}\mathbf{1})_2(\mathbf{2})_2](BAr_F)_4$ ([Pd] = 1.96 mM, 450 µL, 0.88 µmol, 1.0 equiv.) in DCM-d_2/MeCN-d_3 (9:1) and adding solutions of methyl vinyl ketone (600 mM, 25 µL, 15.0 µmol, 17.0 equiv.), benzoyl nitromethane (264 mM, 30 µL, 7.92 µmol, 9.0 equiv.), and 18-crown-6 (129 mM, 45 µL, 5.79 µmol, 6.6 equiv.) in DCM-d_2. This afforded a sample containing $[Pd_2(E\text{-}\mathbf{1})_2(\mathbf{2})_2](BAr_F)_4$ ([Pd] = 1.60 mM, 1.0 equiv.), benzoyl nitromethane (14.4 mM, 9.0 equiv.), methyl vinyl ketone (27.3 mM, 17 equiv.) and 18-crown-6 (10.5 mM, 6.6 equiv.) in DCM-d_2/MeCN-d_3 (11:1). The sample was kept at room temperature for 16 hours to produce the Michael addition product, then the sample was irradiated with 530 nm light for 10 min to disassemble $[Pd_2(E\text{-}\mathbf{1})_2(\mathbf{2})_2](BAr_F)_4$ and stop the Michael addition reaction where the ratio of benzoyl nitromethane to product was approximately 10:1. ^{1}H NMR spectra of the sample were collected with tau set to 0.001, 0.1, 0.25, 0.5, 1, 2, 4, 8, 16 sec. The signal intensities for both signals were measured in each spectrum and plotted against tau (Figure S106 and Figure S107) and the data were fit to the Bruker defined monoexponential:

$$I[t] = I[0] + P * exp(-t/T1)$$

For the benzoyl nitromethane product signal at 2.66 ppm, the T_1 was 2.32 s and for the benzoyl nitromethane signal at 5.97 ppm the signal was determined to be 4.42 s.

The relaxation experiment was repeated with optimised tau values for the benzoyl nitromethane signal at 5.97 ppm as this signal had a longer T_1 value than the signal at 2.66 ppm. ^{1}H NMR spectra were collected with tau values set to 0.001, 0.5, 1.5, 2, 3, 4, 6, 8, 12, 16 sec. The signal intensity was measured in each spectrum and plot against tau (Figure S108). The data were fit to a mono-exponential and T_1 was determined to be 4.39 s.

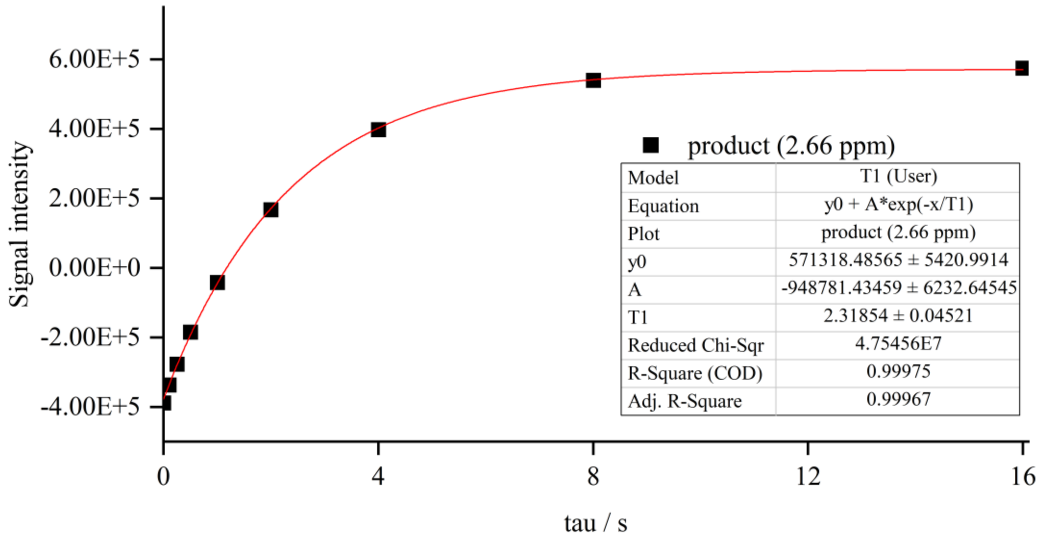

Figure S106. ^{1}H NMR (600 MHz, 298 K, DCM-*d*$_2$:MeCN-*d*$_3$ (11:1)) relaxation experiment to determine T_1 for the Michael addition product signal at 2.66 ppm.

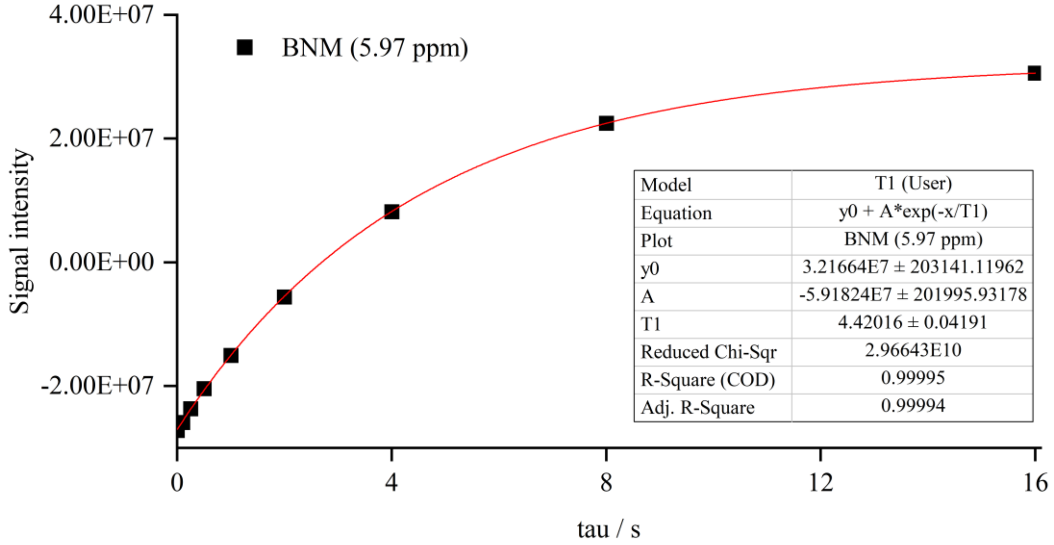

Figure S107. ^{1}H NMR (600 MHz, 298 K, DCM-*d*$_2$:MeCN-*d*$_3$ (11:1)) relaxation experiment to determine T_1 for the benzoyl nitromethane signal at 5.97 ppm.

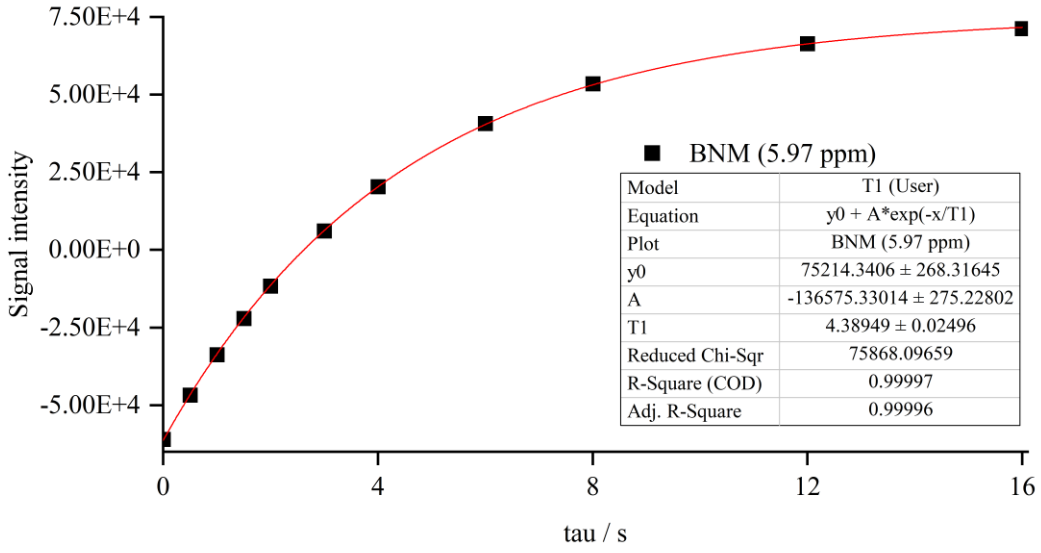

Figure S108. ^{1}H NMR (600 MHz, 298 K, DCM-d_2:MeCN-d_3 (11:1)) relaxation experiment to determine T_1 for the benzoyl nitromethane signal at 5.97 ppm.

S18.4 Monitoring the catalysis of the Michael addition between methyl vinyl ketone and benzoyl nitromethane in DCM:MeCN (11:1) using ^{1}H NMR spectroscopy

S18.4.1 Calculating conversion of benzoyl nitromethane to Michael addition product

Samples containing benzoyl nitromethane (14.4 mM, 9.0 equiv.), methyl vinyl ketone (27.3 mM, 17 equiv.), 18-crown-6 (10.5 mM, 6.6 equiv.) were monitored using ^{1}H NMR spectroscopy, with a spectrum collected every 5 min. The signal intensity for the benzoyl nitromethane signal at 5.97 ppm and the Michael addition product signal at 2.66 ppm were selected as they do not overlap with other signals and they both have the same number of protons in each environment, so a decrease in benzoyl nitromethane signal intensity results in the concomitant increase in the product signal intensity. Representative ^{1}H NMR spectra monitoring the Michael addition reaction are shown in Figure S109.

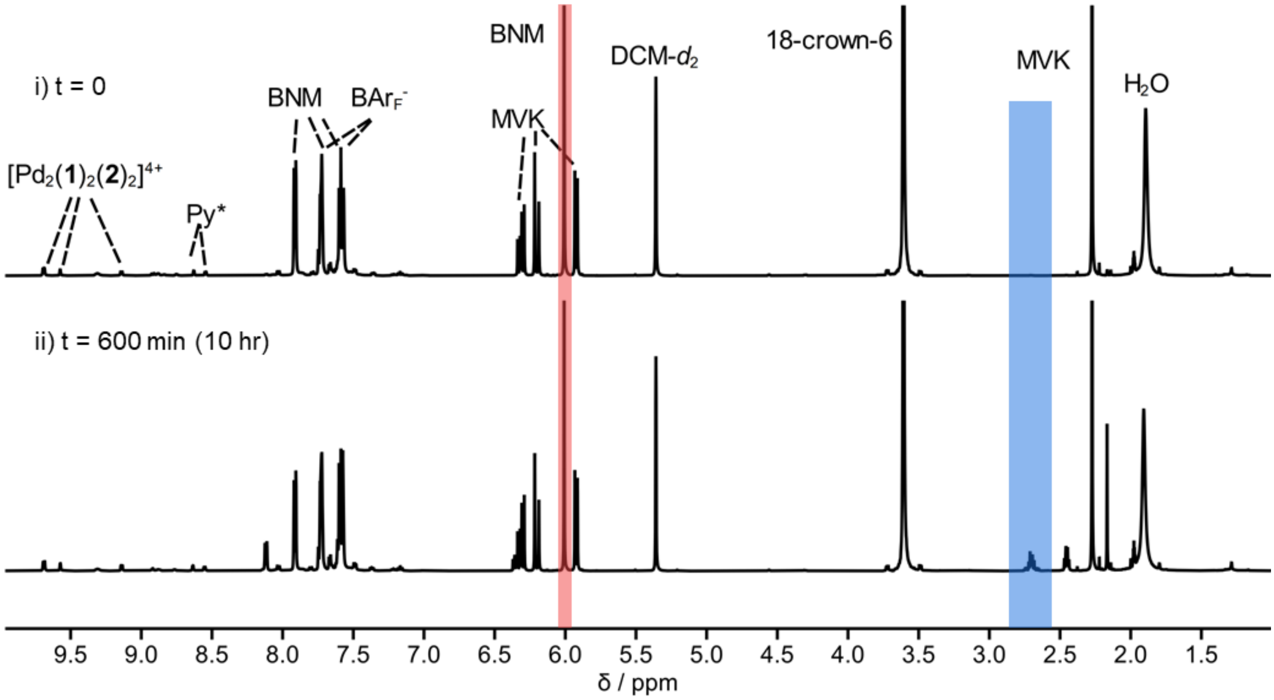

Figure S109. ^{1}H NMR (600 MHz, 298 K, DCM-d_2:MeCN-d_3 (11:1)) spectra of benzoyl nitromethane (14.4 mM, 9.0 equiv.), methyl vinyl ketone (27.3 mM, 17 equiv.), 18-crown-6 (10.5 mM, 6.6 equiv.) and $[Pd_2(E\text{-}\mathbf{1})_2(\mathbf{2})_2](BAr_F)_4$ ([Pd] = 1.6 mM, 1.0 equiv.) i) at the start of the reaction (t = 0) and ii) after 600 min (10 hr). The vertical red line highlights the signal at 5.97 ppm which was used to monitor the abundance of benzoyl nitromethane and the vertical blue line highlights the signal at 2.66 ppm which was used to monitor the abundance of the Michael addition product. Relaxation delay (Bruker setting D1) was set to 5 seconds.

The mol% of benzoyl nitromethane and the Michael addition product relative to each other were determined by dividing all signal intensities by the sum of the signal intensities for benzoyl nitromethane and the product at the start of the reaction (i.e. t = 0):

$$mol\% = \left(\frac{SI}{BNM_0 + P_0}\right) \times 100$$

Mol% = mol% of either benzoyl nitromethane or product

SI = signal intensity of either benzoyl nitromethane or product

BNM_0 = signal intensity of benzoyl nitromethane at t = 0

P_0 = signal intensity of the Michael addition product at t = 0

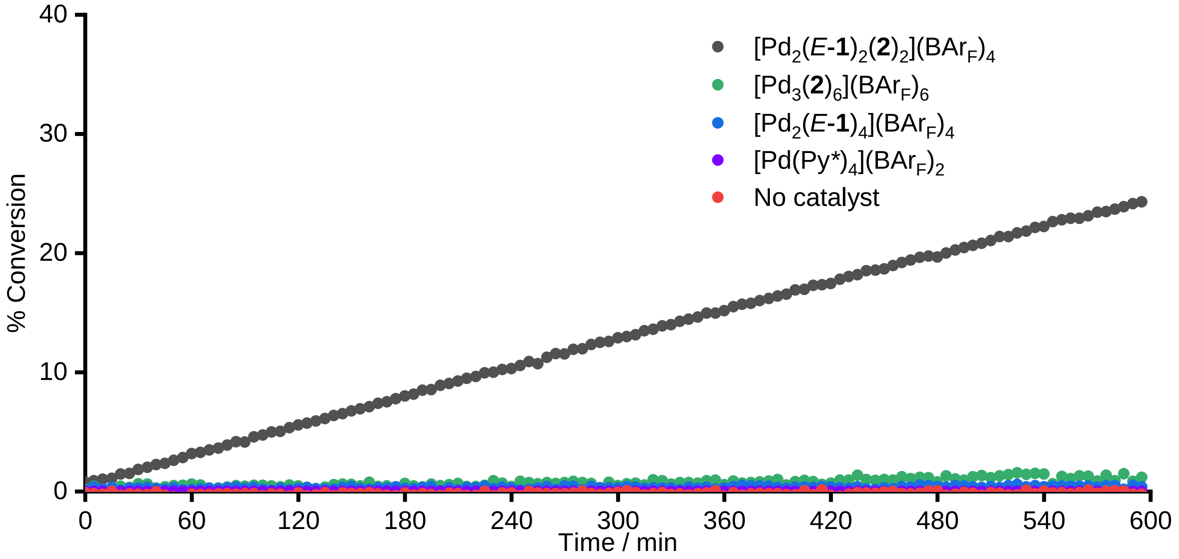

Figure S110. ^{1}H NMR (600 MHz, 298 K, DCM-d_2:MeCN-d_3 (11:1)) signal intensities of Michael addition product following the reaction between benzoyl nitromethane (14.4 mM, 9.0 equiv.) and methyl vinyl ketone (27.3 mM, 17 equiv.) in the presence of 18-crown-6 (10.5 mM, 6.6 equiv.) using different self-assembled species as catalysts ([Pd] = 1.6 mM, 1.0 equiv.).

S18.4.2 Using $[Pd_2(E\text{-}\mathbf{1})_2(\mathbf{2})_2](BAr_F)_4$ to catalyse the Michael addition between methyl vinyl ketone and benzoyl nitromethane

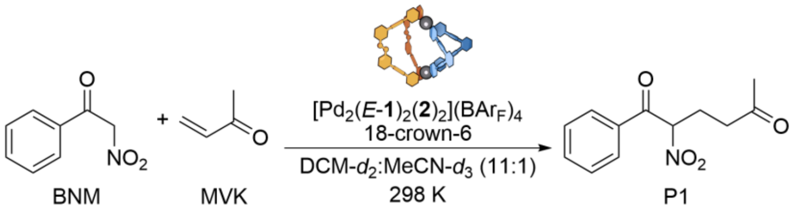

An NMR tube was charged with $[Pd_2(E\text{-}\mathbf{1})_2(\mathbf{2})_2](BAr_F)_4$ ([Pd] = 1.96 mM, 450 µL, 0.88 µmol, 1.0 equiv.) in DCM-d_2:MeCN-d_3 (9:1) (Figure S111i). Then the stock solutions of methyl vinyl ketone (600 mM, 25 µL, 15.0 µmol, 17.0 equiv.) and benzoyl nitromethane (264 mM, 30 µL, 7.92 µmol, 9.0 equiv.) in DCM-d_2 were added. The Michael addition was initiated by the addition of 18-crown-6 stock solution (129 mM, 45 µL, 5.79 µmol, 6.6 equiv.) in DCM-d_2. This afforded a sample containing $[Pd_2(E\text{-}\mathbf{1})_2(\mathbf{2})_2](BAr_F)_4$ ([Pd] = 1.60 mM, 1.0 equiv.), benzoyl nitromethane (14.4 mM, 9.0 equiv.), methyl vinyl ketone (27.3 mM, 17 equiv.) and 18-crown-6 (10.5 mM, 6.6 equiv.) in DCM-d_2:MeCN-d_3 (11:1) (Figure S111ii). The mol% of benzoyl nitromethane and the product were plot against time (Figure S112, see Supporting Information section S18.4.1 for details) to determine whether $[Pd_2(E\text{-}\mathbf{1})_2(\mathbf{2})_2](BAr_F)_4$ can catalyse the reaction. Over 10 hours, 24% of the benzoyl nitromethane was converted into the product.

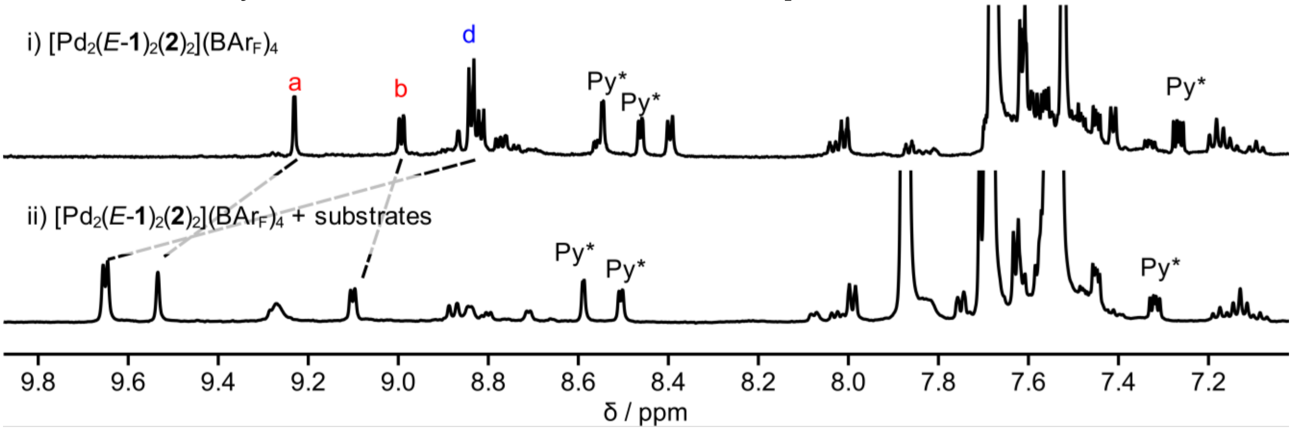

Figure S111. ^{1}H NMR (600 MHz, 298 K) of i) $[Pd_2(E\text{-}\mathbf{1})_2(\mathbf{2})_2](BAr_F)_4$ ([Pd] = 1.96 mM) in DCM-d_2/MeCN-d_3 (9:1), and ii) the same sample after adding Michael addition substrates, affording a sample of $[Pd_2(E\text{-}\mathbf{1})_2(\mathbf{2})_2](BAr_F)_4$ ([Pd] = 1.60 mM, 1.0 equiv.), benzoyl nitromethane (14.4 mM, 9.0 equiv.), methyl vinyl ketone (27.3 mM, 17 equiv.) and 18-crown-6 (10.5 mM, 6.6 equiv.) in DCM-d_2:MeCN-d_3 (11:1).

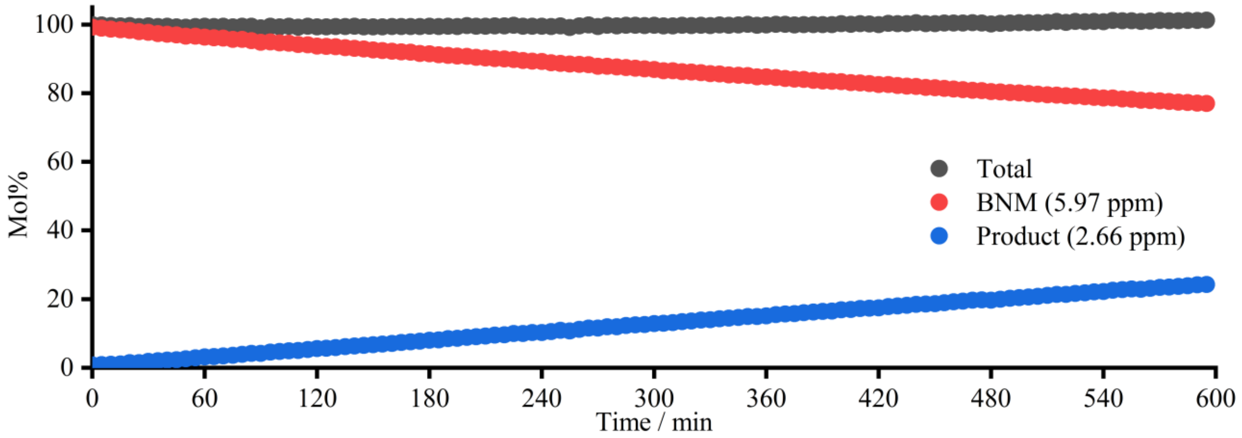

Figure S112. Monitoring catalysis within heteroleptic cage $[Pd_2(E\text{-}\mathbf{1})_2(\mathbf{2})_2](BAr_F)_4$. ^1H NMR (600 MHz, 298 K, DCM-d_2:MeCN-d_3 (11:1)) signal intensities of benzoyl nitromethane and Michael addition product following the reaction between benzoyl nitromethane (14.4 mM, 9.0 equiv.) and methyl vinyl ketone (27.3 mM, 17 equiv.) in the presence of 18-crown-6 (10.5 mM, 6.6 equiv.) and $[Pd_2(E\text{-}\mathbf{1})_2(\mathbf{2})_2](BAr_F)_4$ ([Pd] = 1.6 mM, 1.0 equiv.). The integral of the signal at 5.97 ppm was used to monitor the abundance of benzoyl nitromethane and the integral of the signal at 2.66 ppm was used to monitor the abundance of the product Michael addition product. Relaxation delay (Bruker setting D1) was set to 5 seconds.

S18.4.3 Using $[Pd_2(E\text{-}1)_4](BAr_F)_4$ to catalyse the Michael addition between methyl vinyl ketone and benzoyl nitromethane

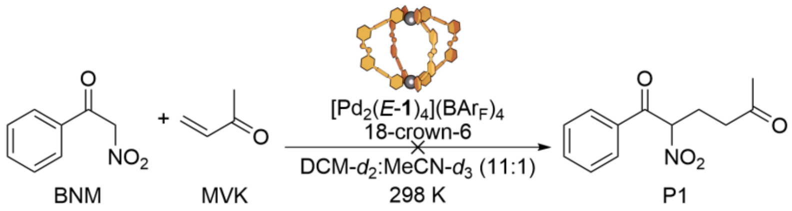

An NMR tube was charged with $[Pd_2(E\text{-}\mathbf{1})_4](BAr_F)_4$ ([Pd] = 1.96 mM, 450 µL, 0.88 µmol, 1.0 equiv.) in DCM-d_2:MeCN-d_3 (9:1) (Figure S113i). Then the stock solutions of methyl vinyl ketone (600 mM, 25 µL, 15.0 µmol, 17.0 equiv.) and benzoyl nitromethane (264 mM, 30 µL, 7.92 µmol, 9.0 equiv.) in DCM-d_2 were added. The 18-crown-6 stock solution (129 mM, 45 µL, 5.79 µmol, 6.6 equiv.) in DCM-d_2 was added last. This afforded a sample containing $[Pd_2(E\text{-}\mathbf{1})_4](BAr_F)_4$ ([Pd] = 1.60 mM, 1.0 equiv.), benzoyl nitromethane (14.4 mM, 9.0 equiv.), methyl vinyl ketone (27.3 mM, 17 equiv.) and 18-crown-6 (10.5 mM, 6.6 equiv.) in DCM-d_2:MeCN-d_3 (11:1) (Figure S113ii). The mol% of benzoyl nitromethane and the product were plot against time (Figure S114, see Supporting Information section S18.4.1 for details) to determine whether $[Pd_2(E\text{-}\mathbf{1})_4](BAr_F)_4$ can catalyse the reaction. No product formation was observed after 10 hours.

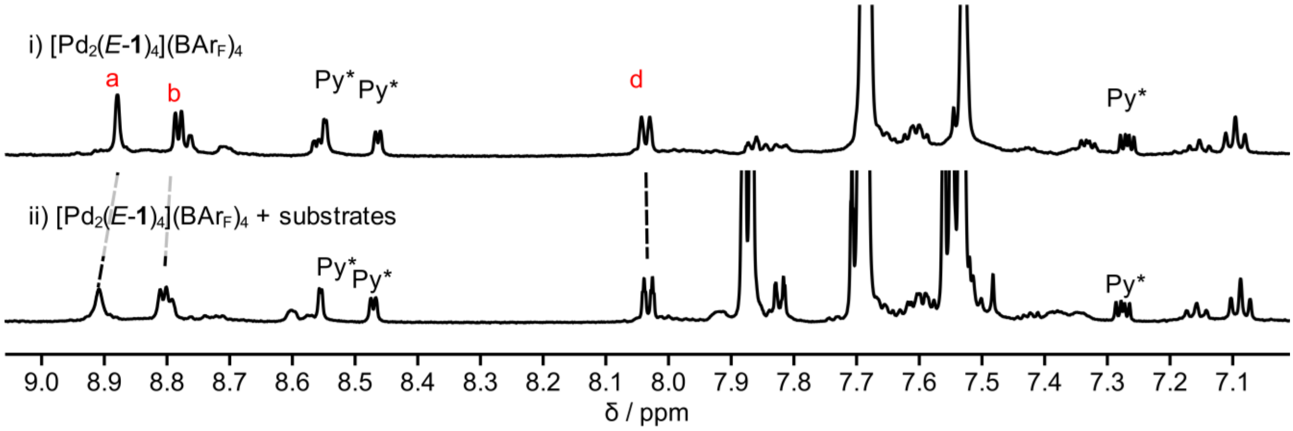

Figure S113. ^1H NMR (600 MHz, 298 K) of i) $[Pd_2(E\text{-}\mathbf{1})_4](BAr_F)_4$ ([Pd] = 1.96 mM) in DCM-d_2/MeCN-d_3 (9:1), and ii) the same sample after adding Michael addition substrates, affording a sample of $[Pd_2(E\text{-}\mathbf{1})_2(\mathbf{2})_2](BAr_F)_4$ ([Pd] = 1.60 mM, 1.0 equiv.), benzoyl nitromethane (14.4 mM, 9.0 equiv.), methyl vinyl ketone (27.3 mM, 17 equiv.) and 18-crown-6 (10.5 mM, 6.6 equiv.) in DCM-d_2:MeCN-d_3 (11:1).

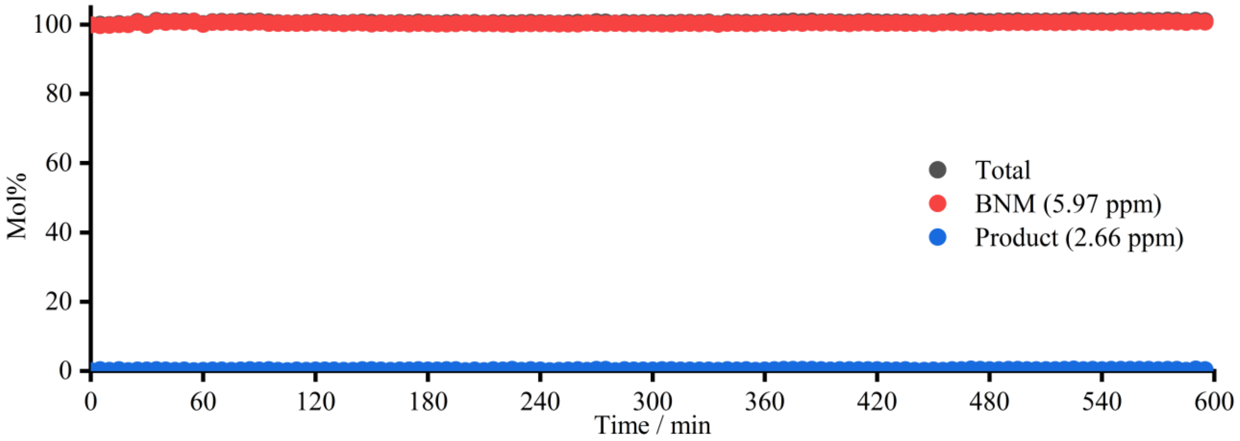

Figure S114. ^{1}H NMR (600 MHz, 298 K, DCM-d_2:MeCN-d_3 (11:1)) signal intensities of benzoyl nitromethane and the Michael addition product following the reaction between benzoyl nitromethane (14.4 mM, 9.0 equiv.) and methyl vinyl ketone (27.3 mM, 17 equiv.) in the presence of 18-crown-6 (10.5 mM, 6.6 equiv.) and $[Pd_2(E\text{-}\mathbf{1})_4](BAr_F)_4$ ([Pd] = 1.6 mM, 1.0 equiv.). The integral of the signal at 5.97 ppm was used to monitor the abundance of benzoyl nitromethane and the integral of the signal at 2.66 ppm was used to monitor the abundance of the Michael addition product. Relaxation delay (Bruker setting D1) was set to 5 seconds.

S18.4.4 Using $[Pd_3(\mathbf{2})_6](BAr_F)_6$ to catalyse the Michael addition between methyl vinyl ketone and benzoyl nitromethane

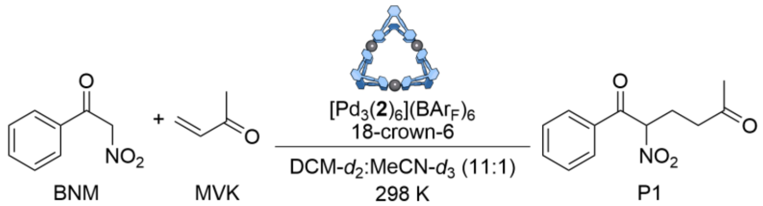

An NMR tube was charged with $[Pd_3(\mathbf{2})_6](BAr_F)_6$ ([Pd] = 1.96 mM, 450 µL, 0.88 µmol, 1.0 equiv.) in DCM-d_2:MeCN-d_3 (9:1) (Figure S115i). Then the stock solutions of methyl vinyl ketone (600 mM, 25 µL, 15.0 µmol, 17.0 equiv.) and benzoyl nitromethane (264 mM, 30 µL, 7.92 µmol, 9.0 equiv.) in DCM-d_2 were added. The Michael addition was initiated by the addition of 18-crown-6 stock solution (129 mM, 45 µL, 5.79 µmol, 6.6 equiv.) in DCM-d_2. This afforded a sample containing $[Pd_3(\mathbf{2})_6](BAr_F)_6$ ([Pd] = 1.60 mM, 1.0 equiv.), benzoyl nitromethane (14.4 mM, 9.0 equiv.), methyl vinyl ketone (27.3 mM, 17 equiv.) and 18-crown-6 (10.5 mM, 6.6 equiv.) in DCM-d_2:MeCN-d_3 (11:1) (Figure S115ii). The mol% of benzoyl nitromethane and the product were plot against time (Figure S116, see Supporting Information section S18.4.1 for details) to determine whether $[Pd_3(\mathbf{2})_6](BAr_F)_6$ can catalyse the reaction. Over 10 hours, less than 1% benzoyl nitromethane was converted to the Michael addition product.

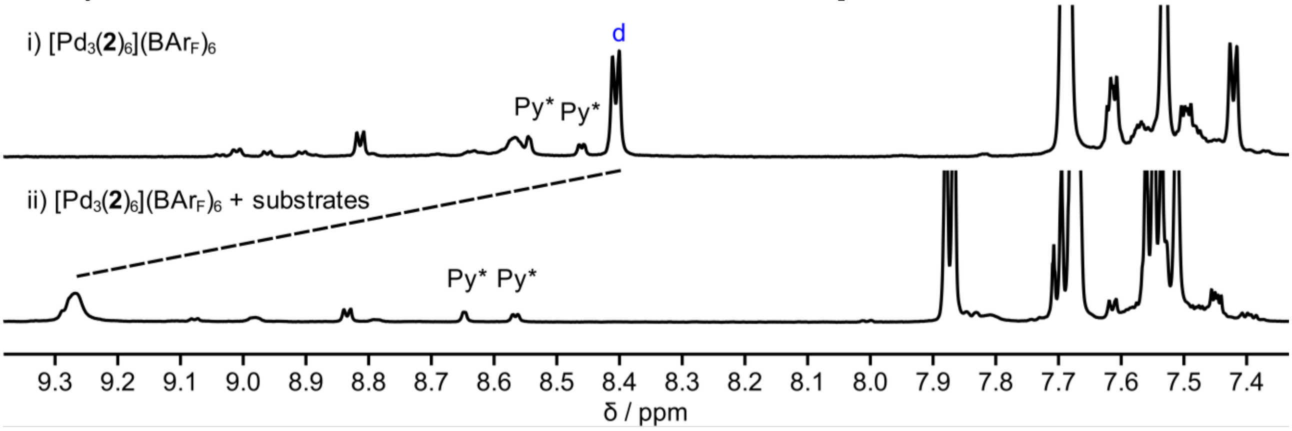

Figure S115. ^{1}H NMR (600 MHz, 298 K) of i) $[Pd_2(E\text{-}\mathbf{1})_4](BAr_F)_4$ ([Pd] = 1.96 mM) in DCM-d_2/MeCN-d_3 (9:1), and ii) the same sample after adding Michael addition substrates, affording a sample of $[Pd_2(E\text{-}\mathbf{1})_2(\mathbf{2})_2](BAr_F)_4$ ([Pd] = 1.60 mM, 1.0 equiv.), benzoyl nitromethane (14.4 mM, 9.0 equiv.), methyl vinyl ketone (27.3 mM, 17 equiv.) and 18-crown-6 (10.5 mM, 6.6 equiv.) in DCM-d_2:MeCN-d_3 (11:1).

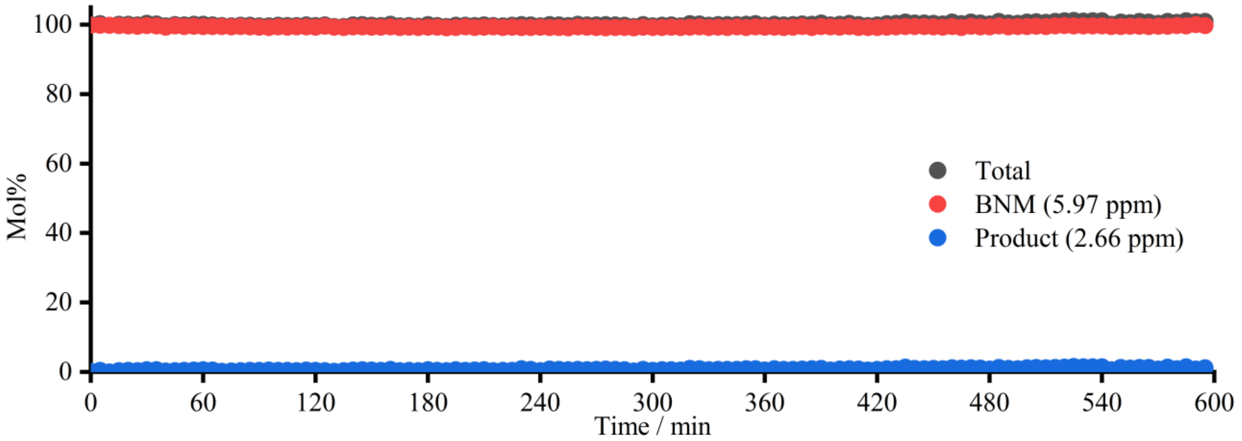

Figure S116. ^{1}H NMR (600 MHz, 298 K, DCM-d_2:MeCN-d_3 (11:1)) signal intensities of benzoyl nitromethane and the Michael addition product following the reaction between benzoyl nitromethane (14.4 mM, 9.0 equiv.) and methyl vinyl ketone (27.3 mM, 17 equiv.) in the presence of 18-crown-6 (10.5 mM, 6.6 equiv.) and $[Pd_3(\mathbf{2})_6](BAr_F)_6$ ([Pd] = 1.6 mM, 1.0 equiv.). The integral of the signal at 5.97 ppm was used to monitor the abundance of benzoyl nitromethane and the integral of the signal at 2.66 ppm was used to monitor the abundance of the Michael addition product. Relaxation delay (Bruker setting d1) was set to 5 seconds.

S18.4.5 Using [Pd(Py*)$_4$](BAr$_F$)$_4$ to catalyse the Michael addition between methyl vinyl ketone and benzoyl nitromethane

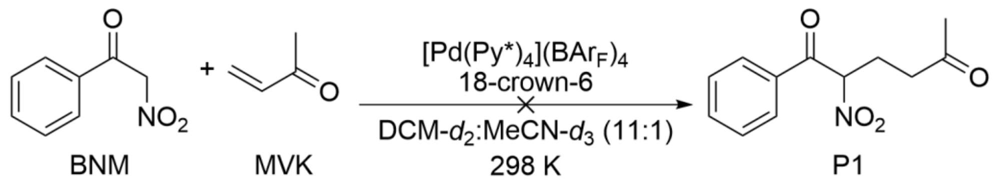

An NMR tube was charged with [Pd(Py*)$_4$](BAr$_F$)$_2$ stock solution (23.9 mM, 37 µL, 0.88 µmol, 1.0 equiv.) in DCM-d_2:MeCN-d_3 (9:1, 413 µL). Then the stock solutions of methyl vinyl ketone (600 mM, 25 µL, 15.0 µmol, 17.0 equiv.) and benzoyl nitromethane (264 mM, 30 µL, 7.92 µmol, 9.0 equiv.) were added. The 18-crown-6 stock solution (129 mM, 45 µL, 5.79 µmol, 6.6 equiv.) in DCM-d_2 was added last. This afforded a sample containing [Pd(Py*)$_4$](BAr$_F$)$_2$ ([Pd] = 1.60 mM, 1.0 equiv.), benzoyl nitromethane (14.4 mM, 9.0 equiv.), methyl vinyl ketone (27.3 mM, 17 equiv.) and 18-crown-6 (10.5 mM, 6.6 equiv.) in DCM-d_2:MeCN-d_3 (11:1). The mol% of benzoyl nitromethane and the product were plot against time (see Supporting Information section S18.4.1 for details) to determine whether [Pd(Py*)$_4$](BAr$_F$)$_2$ can catalyse the reaction. No product formation was observed after 10 hours, demonstrating that palladium(II) was not responsible for the catalysis observed in the sample containing [Pd$_2$(*E*-**1**)$_2$(**2**)$_2$](BAr$_F$)$_4$ (Figure S112).

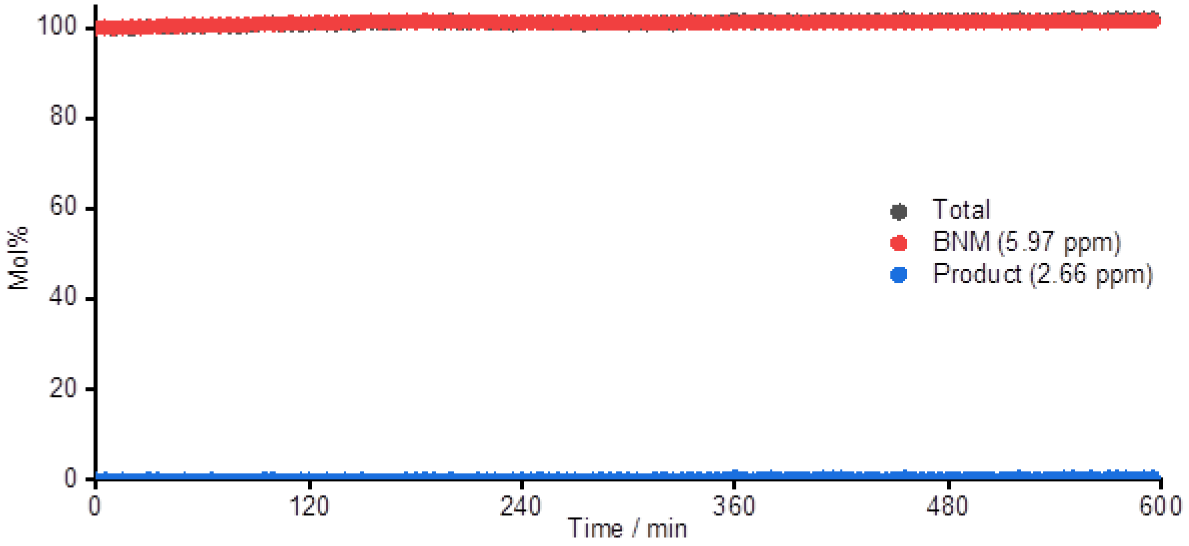

Figure S117. ^{1}H NMR (600 MHz, 298 K, DCM-d_2:MeCN-d_3 (11:1)) signal intensities of benzoyl nitromethane and the Michael addition product following the reaction between benzoyl nitromethane (14.4 mM, 9.0 equiv.) and methyl vinyl ketone (27.3 mM, 17 equiv.) in the presence of 18-crown-6 (10.5 mM, 6.6 equiv.) and [Pd(Py*)$_4$](BAr$_F$)$_6$ ([Pd] = 1.6 mM, 1.0 equiv.). The integral of the signal at 5.97 ppm was used to monitor the abundance of benzoyl nitromethane and the integral of the signal at 2.66 ppm was used to monitor the abundance of the Michael addition product. Relaxation delay (Bruker setting D1) was set to 5 seconds.

S18.4.6 Michael addition between methyl vinyl ketone and benzoyl nitromethane with no catalyst

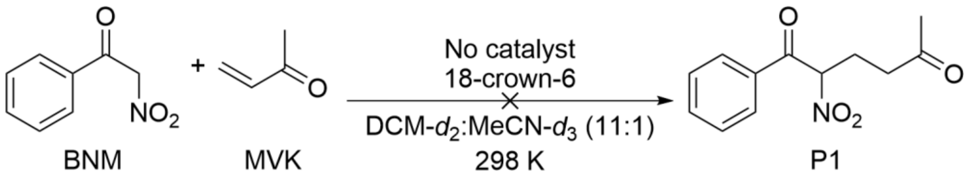

An NMR tube was charged with a solution of DCM-d_2:MeCN-d_3 (9:1, 450 µL). Then the stock solutions of methyl vinyl ketone (600 mM, 25 µL, 15.0 µmol, 17.0 equiv.) and benzoyl nitromethane (264 mM, 30 µL, 7.92 µmol, 9.0 equiv.) were added in DCM-d_2. The 18-crown-6 stock solution (129 mM, 45 µL, 5.79 µmol, 6.6 equiv.) in DCM-d_2 was added last. This afforded a sample containing benzoyl nitromethane (14.4 mM, 9.0 equiv.), methyl vinyl ketone (27.3 mM, 17 equiv.) and 18-crown-6 (10.5 mM, 6.6 equiv.) in DCM-d_2:MeCN-d_3 (11: 1). The mol% of benzoyl nitromethane and the product were plot against time (see Supporting Information section S18.4.1 for details) to determine whether the Michael addition reaction can proceed without a catalyst. No product formation was observed after 10 hours, demonstrating that the Michael addition reaction does not proceed without a catalyst (Figure S112).

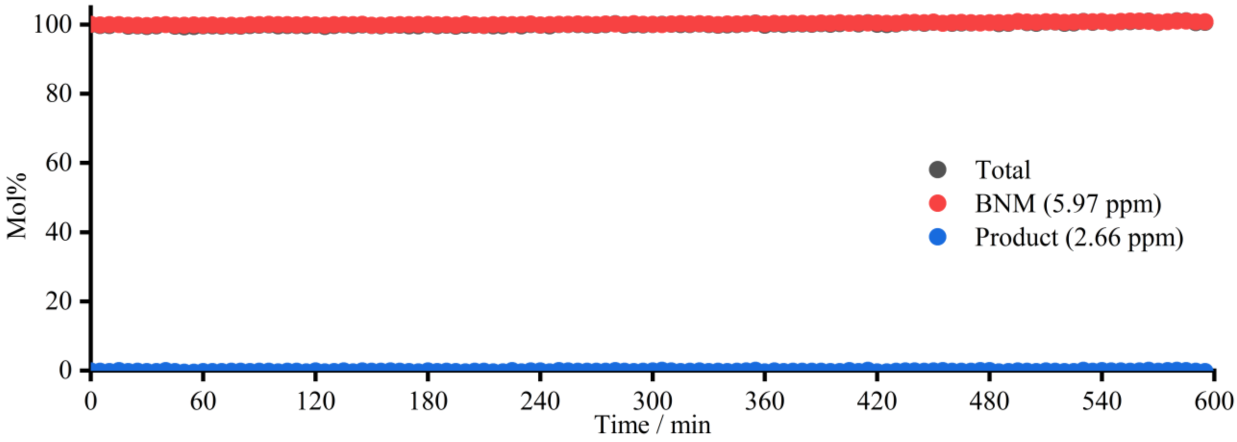

Figure S118. ^{1}H NMR (600 MHz, 298 K, DCM-d_2:MeCN-d_3 (11:1)) signal intensities of benzoyl nitromethane and the Michael addition product following the reaction between benzoyl nitromethane (14.4 mM, 9.0 equiv.) and methyl vinyl ketone (27.3 mM, 17 equiv.) in the presence of 18-crown-6 (10.5 mM, 6.6 equiv.). The integral of the signal at 5.97 ppm was used to monitor the abundance of benzoyl nitromethane and the integral of the signal at 2.66 ppm was used to monitor the abundance of the Michael addition product. Relaxation delay (Bruker setting D1) was set to 5 seconds.

S19. Controlling catalysis with light using $[Pd_2(E\text{-}\mathbf{1})_2(\mathbf{2})_2](BAr_F)_4$ in DCM/MeCN (11:1)

S19.1 Preparation of stock solutions for catalysis

Preparation of $[Pd_2(E\text{-}\mathbf{1})_2(\mathbf{2})_2](BAr_F)_4$:

A sample of $[Pd_2(E\text{-}\mathbf{1})_2(\mathbf{2})_2](BAr_F)_4$ was prepared by reacting $[Pd_2(E\text{-}\mathbf{1})_4](BAr_F)_4$ from the stock solution ([Pd] = 1.96 mM, 270 µL, 0.53 µmol, 1.0 equiv.) with the stock solution of $[Pd_3(\mathbf{2})_6](BAr_F)_6$ ([Pd] = 1.96 mM, 270 µL, 0.53 µmol l, 1.0 equiv.). The sample was heated to 50 °C and argon was blown over the sample to remove the solvent. The residue was redissolved in MeCN-d_3 (45 uL) and DCM-d_2 (455 µL) to afford a sample of $[Pd_2(E\text{-}\mathbf{1})_2(\mathbf{2})_2](BAr_F)_4$ ([Pd] = 2.12 mM, 500 µL, 1.06 µmol, 1.0 equiv.) for catalysis.

Preparation of the methyl vinyl ketone stock solution:

Methyl vinyl ketone (10 µL, 8.41 mg, 120 µmol) was dissolved in DCM-d_2 (90 µL) to afford a stock solution of methyl vinyl ketone (1200 mM).

Preparation of the benzoyl nitromethane stock solution:

Benzoyl nitromethane (10.0 mg, 60.6 µmol) was dissolved in DCM-d_2 (100 µL) to afford a stock solution of benzoyl nitromethane (606 mM).

Preparation of the 18-crown-6 stock solution:

18-crown-6 (17.5 mg, 6.63 µmol) was dissolved in DCM-d_2 (100 µL) to afford a stock solution of 18-crown-6 (663 mM).

S19.2 Deactivating catalysis using 530 nm light

An NMR tube was charged with $[Pd_2(E\text{-}\mathbf{1})_2(\mathbf{2})_2](BAr_F)_4$ ([Pd] = 2.12 mM, 500 µL, 1.06 µmol, 1.0 equiv.) in DCM-d_2:MeCN-d_3 (10:1) (Figure S119i). To ensure $[Pd_2(E\text{-}\mathbf{1})_2(\mathbf{2})_2]^{4+}$ could be reversibly disassembled and reassembled with light, the sample was irradiated with an LED centred at 530 nm for 10 min (Figure S119ii), then irradiated with an LED centred at 405 nm for 5 min (Figure S119iii). Irradiating with the 530 nm light resulted in the disassembly of the $[Pd_2(E\text{-}\mathbf{1})_2(\mathbf{2})_2]^{4+}$ and irradiation with 405 nm light regenerated $[Pd_2(E\text{-}\mathbf{1})_2(\mathbf{2})_2]^{4+}$ near quantitatively, as was observed for the sample of $[Pd_2(E\text{-}\mathbf{1})_2(\mathbf{2})_2](BAr_F)_4$ in 10:1 DCM-d_2:MeCN-d_3 (S11.3).

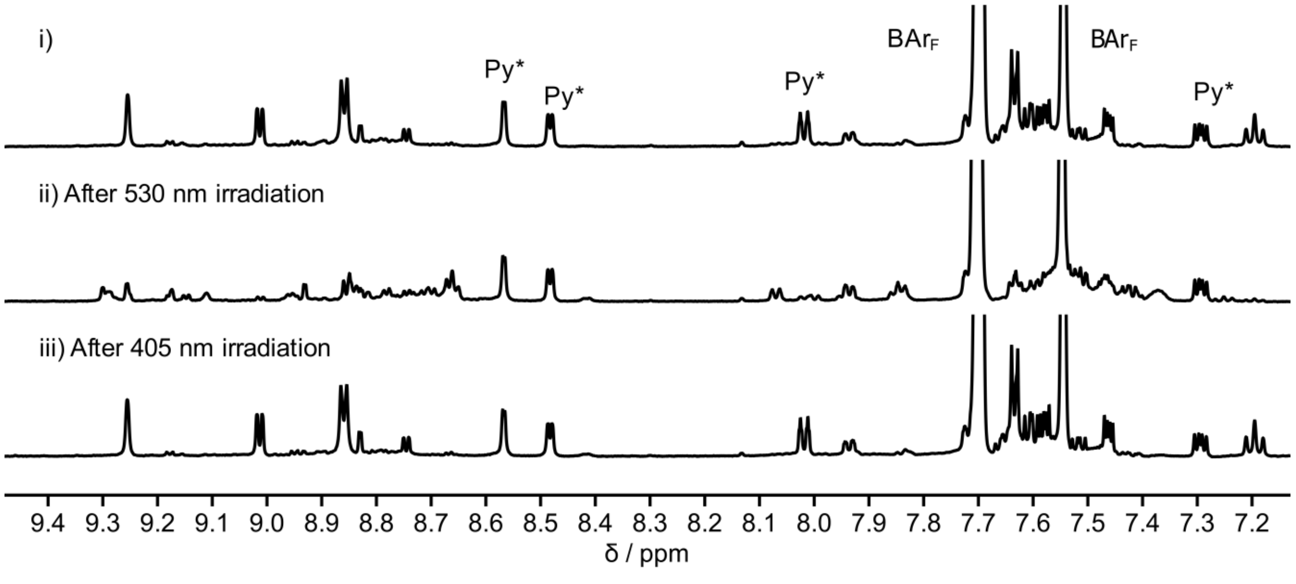

Figure S119. ^{1}H NMR spectra (600 MHz, 298 K, DCM-d_2) of i) $[Pd_2(E\text{-}\mathbf{1})_2(\mathbf{2})_2](BAr_F)_4$ ([Pd] = 2.1 mM), ii) the previous sample after irradiation with 530 nm light for 10 min, and iii) the previous sample after irradiation with 405 nm light to 3 min.

Stock solutions of methyl vinyl ketone (1.20 M, 15 µL, 18 µmol, 17 equiv.) and benzoyl nitromethane (606 mM, 15 µL, 9.1 µmol, 8.5 equiv.) were added to the sample of $[Pd_2(E\text{-}\mathbf{1})_2(\mathbf{2})_2](BAr_F)_4$ ([Pd] = 2.12 mM, 500 µL, 1.06 µmol, 1.0 equiv.) in DCM-d_2:MeCN-d_3 (10:1). The Michael addition was initiated by the addition of 18-crown-6 stock solution (660 mM, 10 µL, 6.6 µmol, 6.2 equiv.). This afforded a catalysis sample containing $[Pd_2(E\text{-}\mathbf{1})_2(\mathbf{2})_2](BAr_F)_4$ ([Pd] = 1.96 mM, 1.0 equiv.), benzoyl nitromethane (16.8 mM, 8.5 equiv.), methyl vinyl ketone (33 mM, 17 equiv.) and 18-crown-6 (12.3 mM, 6.2 equiv.) in DCM-d_2:MeCN-d_3 (11:1).

The catalysis sample was monitored using ^{1}H NMR spectroscopy, with a spectrum collected every 5 min. After 3 hours the sample was removed from the NMR instrument and irradiated with an LED centred at 530 nm for 10 min. The sample was immediately placed back in the NMR instrument to avoid exposure to ambient light and was monitored for additional 3 hours. The sample was then removed from the NMR instrument again, irradiated with 405 nm light for 5 min, immediately placed back in the NMR instrument and monitored for an additional 4 hours. The generation of the Michael addition product was determined by measuring the signal intensity for the benzoyl nitromethane signal at 5.97 ppm and the Michael addition product signal at 2.66 ppm. The signal intensities were plot against time to estimate how the rate of product formation was affected upon irradiation with 530 nm and 405 nm light.

To qualitatively determine how the rate of product formation changes after irradiation, the signal intensity data was fit to a pseudo zeroth order linear function. At the start of the reaction, the pseudo zeroth order rate constant for the conversion of benzoyl nitromethane was 1.2×10^{-3} min^{-1}. Irradiating the sample with a 530 nm for 10 min reduced the pseudo zeroth order rate constant to 1.2×10^{-4} min^{-1}, a ~10-fold decrease compared to before the sample was irradiated. The rate of product formation increased after irradiation with 405 nm light for 5 min, with a pseudo zeroth order rate constant of 8.4×10^{-4} min^{-1}.

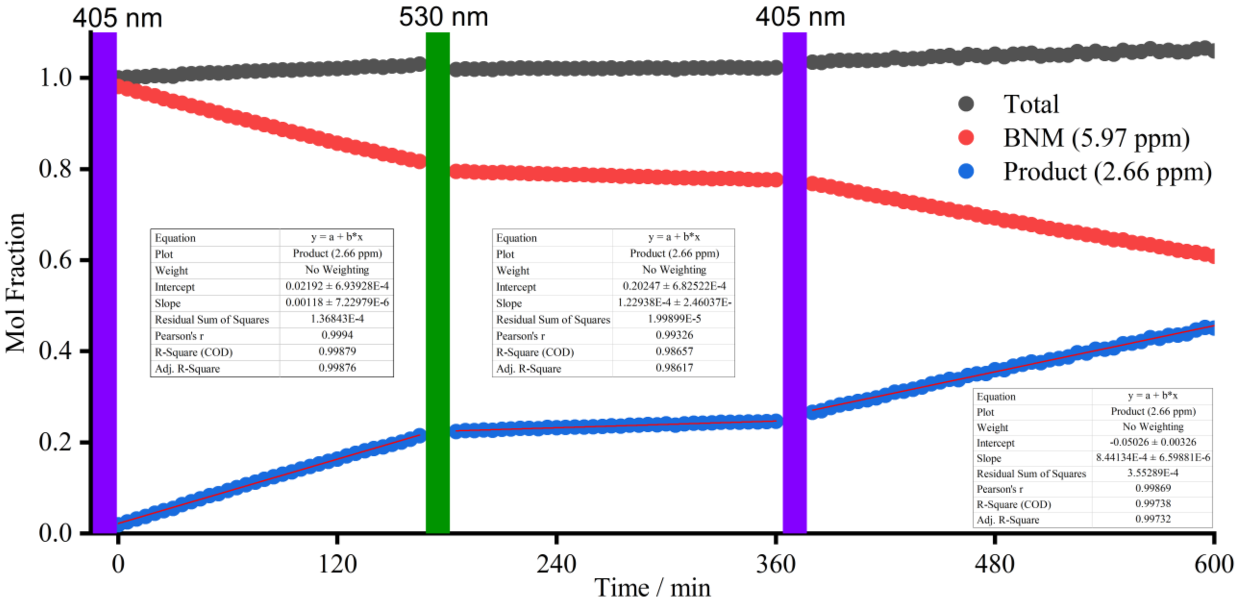

Figure S120. ^{1}H NMR (600 MHz, DCM-d_2/MeCN-d_3 (11:1), 298 K) integrals monitoring the formation of the Michael addition product in the presence of self-assembled product $[Pd_2(E\text{-}\mathbf{1})_2(\mathbf{2})_2](BAr_F)_4$. Sample concentrations: $[Pd_2(E\text{-}\mathbf{1})_2(\mathbf{2})_2](BAr_F)_4$ ([Pd] = 1.96 mM, 1.0 equiv.), benzoyl nitromethane (16.8 mM, 8.5 equiv.), methyl vinyl ketone (33.3 mM, 17 equiv.) and 18-crown-6 (12.3 mM, 6.2 equiv.). The integral of the signal at 5.97 ppm was used to monitor the abundance of benzoyl nitromethane and the integral of the signal at 2.66 ppm was used to monitor the abundance of the Michael addition product. The green vertical line indicates when the sample was irradiated with 530 nm light. The violet vertical line indicates when the sample was irradiated with 405 nm light.

S19.3 Activating catalysis using 405 nm light

An NMR tube was charged with [Pd_2(*E*-**1**)$_2$(**2**)$_2$](BAr_F)$_4$ ([Pd] = 2.12 mM, 500 µL, 1.06 µmol, 1.0 equiv.) in DCM-d_2:MeCN-d_3 (10:1). The sample was irradiated with an LED centred at 530 nm for 10 min. Then the stock solutions of methyl vinyl ketone (1200 mM, 15 µL, 18 µmol, 17 equiv.) and benzoyl nitromethane (606 mM, 15 µL, 9.1 µmol, 8.5 equiv.) were added. The Michael addition was initiated by the addition of 18-crown-6 stock solution (660 mM, 10 µL, 6.6 µmol, 6.2 equiv.). This afforded a sample containing [Pd_2(*E*-**1**)$_2$(**2**)$_2$](BAr_F)$_4$ ([Pd] = 1.96 mM, 1.0 equiv.), benzoyl nitromethane (16.8 mM, 8.5 equiv.), methyl vinyl ketone (33.3 mM, 17 equiv.) and 18-crown-6 (12.3 mM, 6.2 equiv.) in DCM-d_2:MeCN-d_3 (11:1).

The sample was monitored using ^{1}H NMR spectroscopy, with a spectrum collected every 5 min. After 3 hours the sample was removed from the NMR instrument and irradiated with an LED centred at 405 nm for 5 min. The sample was immediately placed back in the NMR instrument to avoid exposure to ambient light and was monitored for additional 3 hour. The sample was then removed from the NMR instrument again, irradiated with 530 nm light for 10 min, immediately placed back in the NMR instrument and monitored for an additional 4 hours. The generation of the Michael addition product was determined by measuring the signal intensity for the benzoyl nitromethane signal at 5.97 ppm and the Michael addition product signal at 2.66 ppm. The signal intensities were plot against time to determine how the rate of product formation was affected upon irradiation with 530 nm and 405 nm light.

To qualitatively determine how the rate of product formation changes after irradiation, the signal intensity data was fit to a pseudo zeroth order linear function. At the start of the experiment, the pseudo zeroth order rate constant for the conversion of benzoyl nitromethane was 7.9×10^{-5} min^{-1}. Irradiating the sample with a 405 nm for 5 min reduced the pseudo zeroth order rate constant to 6.8×10^{-4} min^{-1}, a ~9-fold decrease compared to before the sample was irradiated. The rate of product formation was then reduced by irradiating with 530 nm light for 10 min, with a pseudo zeroth order rate constant of 2.8×10^{-5} min^{-1}.

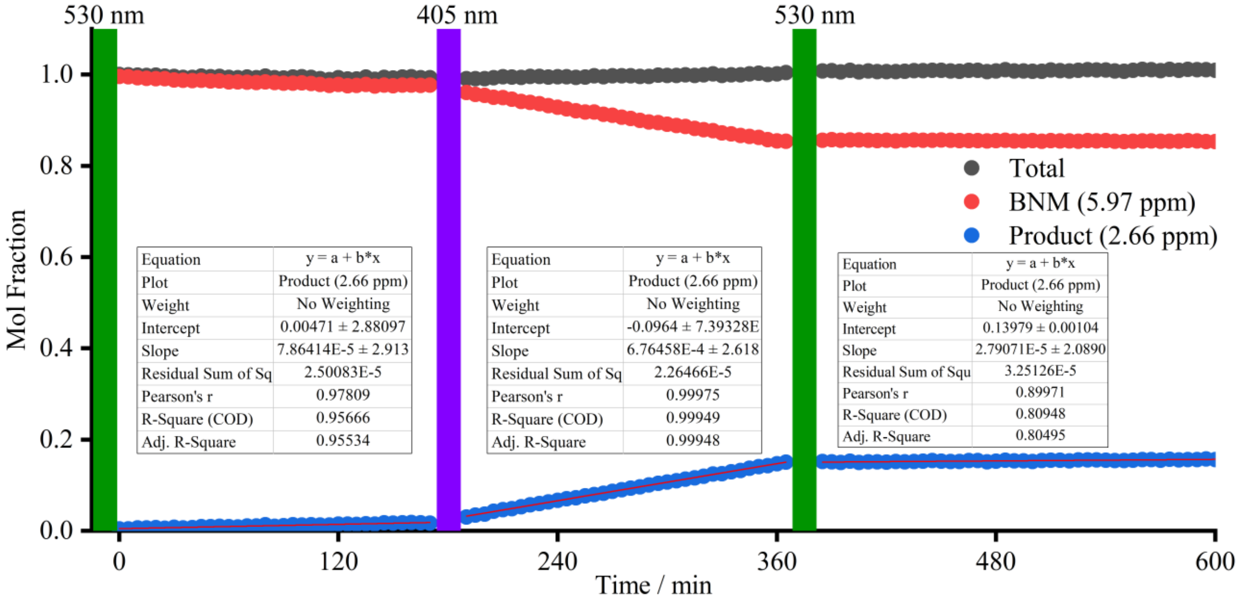

Figure S121. ^{1}H NMR (600 MHz, DCM-d_2/MeCN-d_3 (11:1), 298 K) integrals monitoring the formation of the Michael addition product in the presence of self-assembled product [Pd_2(*E*-**1**)$_2$(**2**)$_2$](BAr_F)$_4$. Sample concentrations: [Pd_2(*E*-**1**)$_2$(**2**)$_2$](BAr_F)$_4$ ([Pd] = 1.96 mM, 1.0 equiv.), benzoyl nitromethane (16.8 mM, 8.5 equiv.), methyl vinyl ketone (33.3 mM, 17 equiv.) and 18-crown-6 (12.3 mM, 6.2 equiv.). The integral of the signal at 5.97 ppm was used to monitor the abundance of benzoyl nitromethane and the integral of the signal at 2.66 ppm was used to monitor the abundance of the Michael addition product. The green vertical line indicates when the sample was irradiated with 530 nm light. The violet vertical line indicates when the sample was irradiated with 405 nm light.

S19.4 Multiple switching cycles, followed by catalysis

To verify the cage retains its catalytic activity after multiple switching cycles we subjected a sample of $[Pd_2(E\text{-}\mathbf{1})_2(\mathbf{2})_2](BAr_F)_4$ ([Pd] = 1.96 mM) in 9:1 DCM-d_2:MeCN-d_3 to five switching cycles of 530 nm (10 min) and 405 nm (5 min) irradiation, followed by addition of substrates and 18-crown-6 in DCM-d_2 to give final sample concentrations: $[Pd_2(E\text{-}\mathbf{1})_2(\mathbf{2})_2](BAr_F)_4$ ([Pd] = 1.96 mM, 1.0 equiv.), benzoyl nitromethane (16.8 mM, 8.5 equiv.), methyl vinyl ketone (33.3 mM, 17 equiv.) and 18-crown-6 (12.3 mM, 6.2 equiv.) in 11:1 DCM-d_2:MeCN-d_3.

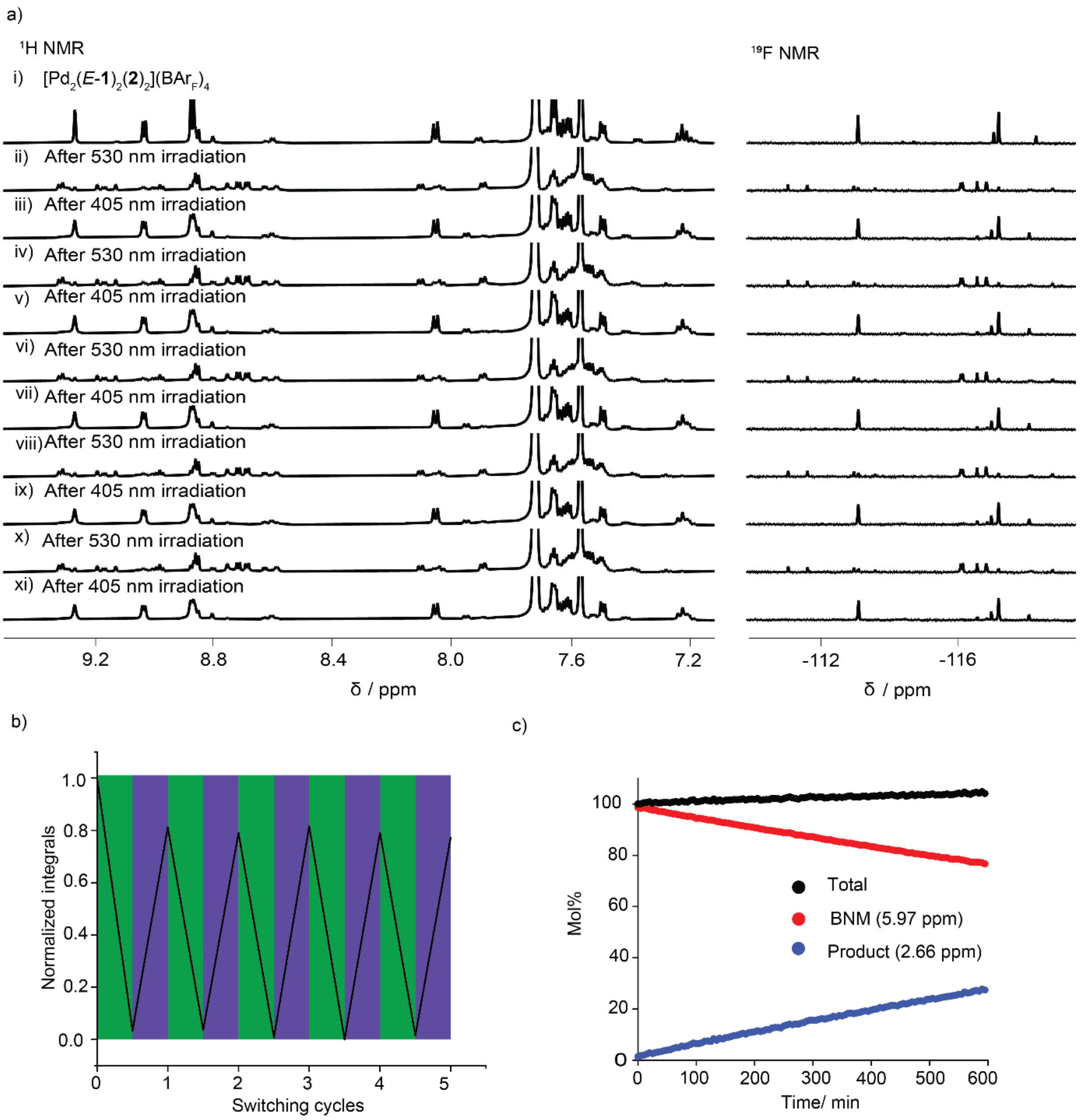

Figure S122. a) ^{1}H NMR and ^{19}F NMR (600 MHz, 298 K) of i) $[Pd_2(E\text{-}\mathbf{1})_2(\mathbf{2})_2](BAr_F)_4$ ([Pd] = 1.96 mM) in DCM-d_2/MeCN-d_3 (9:1), ii) the same sample after 530 nm irradiation for 10 min, iii) after 405 nm irradiation for 5 min, iv) after 530 nm irradiation for 10 min, v) after 405 nm irradiation for 5 min, vi) after 530 nm irradiation for 10 min, vii) after 405 nm irradiation for 5 min, viii) after 530 nm irradiation for 10 min, ix) after 405 nm irradiation for 5 min, x) after 530 nm irradiation for 10 min, and xi) after 405 nm irradiation for 5 min. b) Normalized integrals from ^{19}F NMR (-133.1 ppm) vs number of switching cycles. c) Monitoring catalysis within the same cage sample after five switching cycles. ^{1}H NMR (600 MHz, 298 K, DCM-d_2:MeCN-d_3 (11:1)) signal intensities of benzoyl nitromethane and Michael addition product following the reaction between benzoyl nitromethane (14.4 mM, 9.0 equiv.) and methyl vinyl ketone (27.3 mM, 17 equiv.) in the presence of 18-crown-6 (10.5 mM, 6.6 equiv.) and $[Pd_2(E\text{-}\mathbf{1})_2(\mathbf{2})_2](BAr_F)_4$ ([Pd] = 1.96 mM, 1.0 equiv.). The integral of the signal at 5.97 ppm was used to monitor the abundance of benzoyl nitromethane and the integral of the signal at 2.66 ppm was used to monitor the abundance of the product Michael addition product. Relaxation delay (Bruker setting D1) was set to 5 seconds.

S19.5 Deactivating catalysis by binding OTf in the cavity of the cage

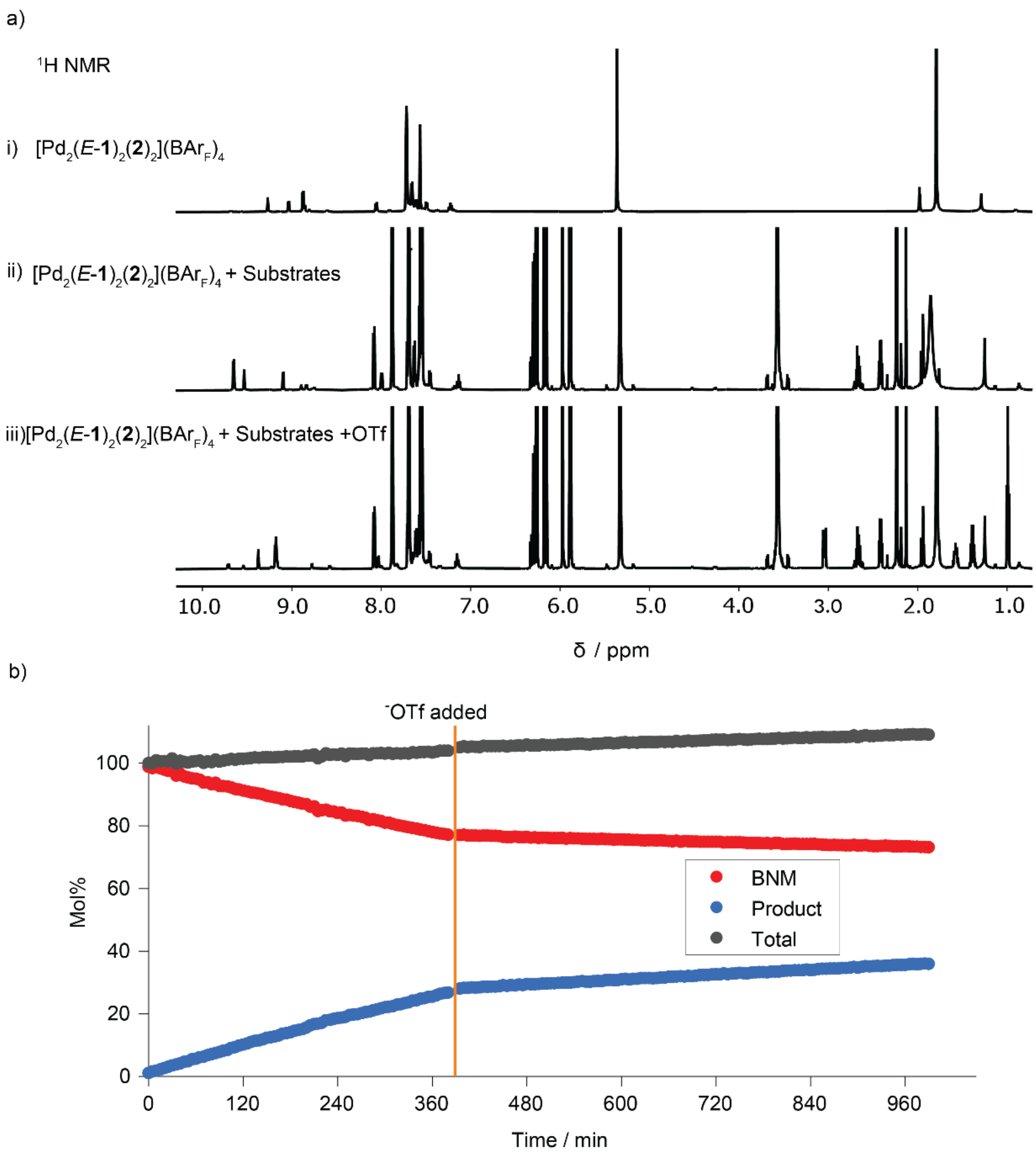

Figure S123. a) ^{1}H NMR (600 MHz, 298 K) of i) [Pd_2(E-**1**)$_2$(**2**)$_2$](BAr$_F$)$_4$ ([Pd] = 1.96 mM) in DCM-d_2/MeCN-d_3 (9:1), ii) the same sample after adding substrates (benzoyl nitromethane (14.4 mM, 9.0 equiv.) and methyl vinyl ketone (27.3 mM, 17 equiv.) in the presence of 18-crown-6 (10.5 mM, 6.6 equiv.) shows binding iii) same sample with substrates after adding TBA OTf (88 mM in DCM-d_2, 5 µL, 0.5 equiv.) inhibits the catalysis b) Monitoring catalysis within heteroleptic cage [Pd_2(E-**1**)$_2$(**2**)$_2$](BAr$_F$)$_4$. ^{1}H NMR (600 MHz, 298 K, DCM-d_2:MeCN-d_3 (11:1)) signal intensities of benzoyl nitromethane and Michael addition product following the reaction between benzoyl nitromethane (14.4 mM, 9.0 equiv.) and methyl vinyl ketone (27.3 mM, 17 equiv.) in the presence of 18-crown-6 (10.5 mM, 6.6 equiv.) and [Pd_2(E-**1**)$_2$(**2**)$_2$](BAr$_F$)$_4$ ([Pd] = 1.96 mM, 1.0 equiv.). The integral of the signal at 5.97 ppm was used to monitor the abundance of benzoyl nitromethane and the integral of the signal at 2.66 ppm was used to monitor the abundance of the product Michael addition product. After 6 hours TBA OTf (88 mM in DCM-d_2, 5 µL, 0.5 equiv.) was added and monitored the catalysis for 10 hours. Relaxation delay (Bruker setting D1) was set to 5 seconds.

S20. References

(1) Antoine John, A.; Lin, Q. Synthesis of Azobenzenes Using N-Chlorosuccinimide and 1,8-Diazabicyclo[5.4.0]undec-7-ene (DBU). *J. Org. Chem.* **2017**, *82* (18), 9873-9876. DOI: 10.1021/acs.joc.7b01530.

(2) Mcphillips, T. M.; Mcphillips, S. E.; Chiu, H.-J.; Cohen, A. E.; Deacon, A. M.; Ellis, P. J.; Garman, E.; Gonzalez, A.; Sauter, N. K.; Phizackerley, R. P.; et al. Blu-Ice and the Distributed Control System: software for data acquisition and instrument control at macromolecular crystallography beamlines. *J. Synchrotron Rad.* **2002**, *9* (6), 401-406. DOI: 10.1107/s0909049502015170.

(3) Kabsch, W. XDS. *Acta Crystallogr. Sect. D. Biol. Crystallogr.* **2010**, *66* (2), 125-132. DOI: 10.1107/s0907444909047337.

(4) Sheldrick, G. M. A short history of SHELX. *Acta Crystallograph. Sect. A* **2008**, *64* (1), 112-122. DOI: 10.1107/s0108767307043930.

(5) Sheldrick, G. M. Crystal structure refinement with *SHELXL*. *Acta Cryst. C* **2015**, *71* (1), 3-8. DOI: 10.1107/s2053229614024218.

(6) Dolomanov, O. V.; Bourhis, L. J.; Gildea, R. J.; Howard, J. A. K.; Puschmann, H. OLEX2: a complete structure solution, refinement and analysis program. *J. Appl. Crystallogr.* **2009**, *42*, 339-341. DOI: 10.1107/s0021889808042726.

(7) Spek, A. L. checkCIF validation ALERTS: what they mean and how to respond. *Acta Crystallogr E* **2020**, *76*, 1-11. DOI: 10.1107/S2056989019016244. Spek, A. checkCIF validation ALERTS: what they mean and how to respond. *Acta Crystallograph. Sect. E* **2020**, *76* (1), 1-11. DOI: doi:10.1107/S2056989019016244.

(8) Kennedy, A. D. W.; DiNardi, R. G.; Fillbrook, L. L.; Donald, W. A.; Beves, J. E. Visible-Light Switching of Metallosupramolecular Assemblies. *Chem.– Eur. J.* **2022**, *28* (16), e202104461. DOI: 10.1002/chem.202104461.

(9) DiNardi, R. G.; Douglas, A. O.; Tian, R.; Price, J. R.; Tajik, M.; Donald, W. A.; Beves, J. E. Visible-Light-Responsive Self-Assembled Complexes: Improved Photoswitching Properties by Metal Ion Coordination. *Angew. Chem. Int. Ed.* **2022**, *61* (38), e202205701. DOI: 10.1002/anie.202205701.

(10) Kai, S.; Sakuma, Y.; Mashiko, T.; Kojima, T.; Tachikawa, M.; Hiraoka, S. The Effect of Solvent and Coordination Environment of Metal Source on the Self-Assembly Pathway of a Pd(II)-Mediated Coordination Capsule. *Inorg. Chem.* **2017**, *56* (20), 12652-12663. DOI: 10.1021/acs.inorgchem.7b02152.

(11) Tateishi, T.; Takahashi, S.; Okazawa, A.; Martí-Centelles, V.; Wang, J.; Kojima, T.; Lusby, P. J.; Sato, H.; Hiraoka, S. Navigated Self-Assembly of a Pd_2L_4 Cage by Modulation of an Energy Landscape under Kinetic Control. *J. Am. Chem. Soc.* **2019**, *141* (50), 19669-19676. DOI: 10.1021/jacs.9b07779.

(12) Martínez-Martínez, A. J.; Weller, A. S. Solvent-free anhydrous Li^+, Na^+ and K^+ salts of $[B(3,5\text{-}(CF_3)_2C_6H_3)_4]^-$, $[BArF_4]^-$. Improved synthesis and solid-state structures. *Dalton Trans.* **2019**, *48* (11), 3551-3554. DOI: 10.1039/C9DT00235A.

(13) Suzuki, K.; Kawano, M.; Fujita, M. Solvato-Controlled Assembly of Pd_3L_6 and Pd_4L_8 Coordination "Boxes". *Angew. Chem. Int. Ed.* **2007**, *46* (16), 2819-2822. DOI: 10.1002/anie.200605084.

(14) Price, W. S. *NMR Studies of Translational Motion: Principles and Applications*; Cambridge University Press, 2009. DOI: DOI: 10.1017/CBO9780511770487.

(15) Stejskal, E. O.; Tanner, J. E. Spin Diffusion Measurements: Spin Echoes in the Presence of a Time - Dependent Field Gradient. *J. Chem. Phys.* **1965**, *42* (1), 288-292. DOI: 10.1063/1.1695690.

(16) Poole, D. A., III; Bobylev, E. O.; de Bruin, B.; Mathew, S.; Reek, J. N. H. Exposing Mechanisms for Defect Clearance in Supramolecular Self-Assembly: Palladium–Pyridine Coordination Revisited. *Inorg. Chem.* **2023**, *62* (14), 5458-5467. DOI: 10.1021/acs.inorgchem.2c04404.
